# Comparative effects and dose–response relationships of exercise for finger dexterity and upper-limb function in Parkinson’s disease: a Bayesian network and model-based dose–response meta-analysis

**DOI:** 10.3389/fneur.2026.1964034

**Published:** 2026-10-05

**Authors:** Bu-Yu Liu, Yu-Xin Zhuang, Chang-Kuan Zhuang, Hui Ruan

**Affiliations:** 1 School of Physical Education, Hainan Normal University, Haikou, Hainan, China; 2 School of Acupuncture-Moxibustion and Tuina, Beijing University of Chinese Medicine, Beijing, China

**Keywords:** dose–response relationship, exercise therapy, finger dexterity, network meta-analysis, Parkinson’s disease, rehabilitation, upper-limb function

## Abstract

**Background:**

Upper-limb impairment limits independence in Parkinson’s disease (PD), but the comparative effects and dose–response profiles of exercise modalities remain uncertain.

**Methods:**

PubMed, Embase, CENTRAL, Web of Science, Scopus, and SPORTDiscus were searched from inception to June 20, 2026. Studies that qualified were randomized controlled trials of structured exercise or rehabilitation in adults with PD. Bayesian random-effects network meta-analysis and model-based dose–response network meta-analysis were conducted separately for finger dexterity and upper-limb function. Weekly dose was expressed as metabolic equivalent of task minutes per week (MET·min/week), and evidence certainty was assessed using CINeMA.

**Results:**

Thirty-six reports comprising 1,575 participants were included. Aerobic exercise training (Hedges’ *g* 1.46, 95% CrI 0.26–2.69) and task-oriented finger-dexterity training (0.96, 0.23–1.67) improved finger dexterity versus usual care. Low-certainty evidence suggested that mind–body exercise may improve upper-limb function, although the unusually large estimate (Hedges’ *g* = 6.90, 95% CrI 4.28–9.43) was based on only two small trials involving 53 participants in the relevant comparison arms. Given the sparse evidence and local inconsistency in the modality-specific dose–response analysis, this result should be regarded as exploratory and interpreted cautiously. Models fitted to mapped dose nodes suggested an approximately linear association for finger dexterity and diminishing incremental gains for upper-limb function. Predictions toward 1,800 MET·min/week partly extended beyond the highest representative dose nodes and remained uncertain.

**Conclusion:**

Exercise effects were outcome specific. The findings can inform exercise-modality selection and future dose-ranging trials, but they do not establish an optimal dose or justify a uniform clinical prescription.

**Systematic review registration:**

https://www.crd.york.ac.uk/PROSPERO/view/CRD420261463560, identifier: CRD420261463560.

## 1 Background

Parkinson’s disease (PD) is a progressive neurodegenerative disorder that can impair everyday use of the hand (1). More than 8.5 million people worldwide were afflicted with PD in 2019 (2), and PD caused substantial disability and mortality in 2021 (3, 4). Retaining independence as the disease progresses is therefore a clinical priority (1, 2).

Bradykinesia, rigidity, tremor, and impaired scaling of repetitive movement can affect hand and upper-limb control in PD (5). These problems disrupt reaching, grasp and release, bimanual coordination, and handwriting (6, 7). They can restrict eating, dressing, writing, and object handling. Finger dexterity refers to rapid, precise, and coordinated finger or hand manipulation. Upper-limb function encompasses reaching, grasping, transferring, and manipulating objects in daily tasks. These are related but distinct functional constructs (7–9). Therefore, this study analyzed them in separate evidence networks.

Pharmacological and surgical treatments alleviate PD symptoms but do not fully prevent functional decline (1, 10). Exercise-based rehabilitation is therefore an important component of long-term PD management (11, 12). Exercise modalities differ in their therapeutic targets, ranging from task-oriented hand practice to technology-assisted, resistance, aerobic, mind–body, and multicomponent training (8, 13–18). Previous exercise syntheses have emphasized overall motor outcomes, gait, or balance, whereas finger dexterity and broader upper-limb function have received less focused synthesis (8, 15).

Exercise prescriptions are described by frequency, intensity, time and type (FITT) (19), yet PD trials vary in weekly dose and intervention length, even within the same modality. Studies also use different measures of dexterity and upper-limb function (7, 8, 20). Three questions remain: which exercise modalities are most effective; whether their effects differ between finger dexterity and broader upper-limb function; and whether weekly dose is associated with the magnitude of benefit. Network meta-analysis compares modalities using direct and indirect evidence, but pooling different doses may obscure dose–response relationships and contribute to heterogeneity (21–23). To address sparse and inconsistently reported dose data, weekly doses estimated from metabolic equivalents (METs), session duration, and frequency can be mapped to prespecified, clinically interpretable nodes that preserve comparability and network connectivity. Modelling representative node values can then estimate trends within the observed dose range (22–24). Evidence applying this approach to both outcomes in PD remains limited (25).

Therefore, this study combined Bayesian network meta-analysis with model-based network meta-analysis of dose–response relationships. Separate networks compared exercise modalities for finger dexterity and upper-limb function, allowing outcome-specific patterns to be examined. Overall and modality-specific dose–response trends were modelled, identifying nodes with stronger support for benefit and the maximum predicted effect within the observed evidence range. Where data, network connectivity and reporting permitted, modification by intervention length, mean age, and outcome-assessment types were explored. These analyses were intended to inform clinical interpretation and generate testable hypotheses for future upper-limb rehabilitation trials in PD.

## 2 Methods

This systematic review was registered prospectively with PROSPERO (CRD420261463560) and is reported in accordance with the PRISMA 2020 statement and the PRISMA extension for network meta-analyses.

### 2.1 Search strategy and search terms

PubMed, Embase, the Cochrane Central Register of Controlled Trials (CENTRAL), Web of Science, Scopus, and SPORTDiscus were systematically searched from inception to June 20, 2026. The search strategy combined three concept blocks: PD, exercise or rehabilitation, and randomized controlled trials. Controlled vocabulary was combined with free-text terms in title, abstract, or keyword fields, with syntax adapted for each database. No language restrictions were applied, and complete line-by-line strategies and search yields are provided in Supplementary File 2.

To minimize missed studies, the reference lists of included studies and relevant systematic reviews were screened. In addition, ClinicalTrials.gov and the WHO International Clinical Trials Registry Platform were searched for potentially eligible completed, unpublished, or ongoing trials.

### 2.2 Eligibility criteria

Randomized controlled trials enrolling adults with clinically diagnosed PD were included. Eligible trials compared a structured exercise or rehabilitation program assignable to a prespecified intervention node with usual care, no structured exercise, another eligible intervention, or a different dose or regimen. They also reported finger dexterity, hand function or upper-limb function outcomes from which an effect size could be extracted or calculated. Dose–response analyses additionally required sufficient arm-level information to estimate weekly exercise dose. Trials lacking these data were retained only in the conventional network meta-analysis by exercise modality.

Non-randomized or single-arm studies and mixed-population trials without separately extractable data for participants with PD were excluded. Also, multimodal interventions that could not be assigned to a prespecified node or isolate the independent effect of the target exercise were excluded. Studies with ineligible comparators, outcomes, or data were also excluded. Multiple reports from the same trial were linked at the trial level. The most complete report for the target outcome and time point was extracted. Detailed eligibility criteria, decision rules, and the rationale and operational definitions for intervention nodes are provided in Supplementary File 3.

### 2.3 Data extraction and effect-size calculation

Two reviewers independently screened records and extracted data according to prespecified criteria. Disagreements were resolved through discussion, with adjudication by a third reviewer when required. A standardized form captured first author, publication year, sample size, age, sex, completion rate, intervention length, frequency, session duration, exercise intensity, and intervention details.

Outcome measures were classified according to the construct assessed rather than the instrument name. This construct-based framework was informed by previous reviews of upper-limb measurement and exercise rehabilitation in PD (7, 8). Finger dexterity was defined as rapid, precise, and coordinated finger or hand manipulation, whereas upper-limb function was defined more broadly as the capacity to generate force, reach, grasp, transfer and manipulate objects, and perform upper-limb-related daily activities. Accordingly, the BBT, HGS, FMA-UE, JTHFT and DASH were treated as indicators of different components or measurement levels within the broader upper-limb-function domain. Finger-dexterity measures were analyzed in a separate network. Only one measure per study within each outcome domain and post-intervention time point was included to avoid double counting.

For both outcome domains, assessments conducted at the end of the intervention were used. The interval from baseline to assessment therefore varied with intervention duration, rather than representing a common follow-up interval across studies.

For continuous outcomes, the mean and standard deviation (SD) of the change from baseline were preferentially extracted. Values reported as medians with interquartile ranges, or means with standard errors (SE or SEM), were converted to means and SDs using Cochrane Handbook methods. Details are provided in Supplementary Table 4.1. When only baseline and endpoint values were reported, mean change was calculated as the difference between the two time points. The SD of the change score was calculated as follows:


$${\mathrm{SD}}_{\text{change}}=\sqrt{{{\mathrm{SD}}_{\text{baseline}}}^{2}+{{\mathrm{SD}}_{\text{endpoint}}}^{2}-2R\times {\mathrm{SD}}_{\text{baseline}}\times {\mathrm{SD}}_{\text{endpoint}}}$$

Where *R* denotes the correlation between baseline and endpoint measurements. When a change-score SD could not be obtained from another statistic, *R* was back-calculated from trials reporting all three SDs for the same outcome. The mean of the derived correlations was used in the primary analysis. Full derivations are reported in Supplementary Tables 4.2A–4.2C. Sensitivity analyses set *R* to 0.50 and 0.90 to assess the influence of this assumption.

All change scores were defined as endpoint values minus baseline values. For scales on which lower scores indicated better function, change scores were multiplied by −1 so that positive effects consistently favored exercise. Because studies used different scales, the small-sample-corrected standardized mean difference, Hedges’ *g*, was used for the conventional network meta-analysis.

### 2.4 Exercise-dose estimation and node mapping

Weekly exercise dose was estimated at the study-arm level from the planned prescription and expressed as metabolic equivalent minutes per week (MET·min/week):


$$\begin{array}{l} \text{Weekly exercise dose}=\mathrm{MET}\times \text{session duration}\;(\min)\\ \times \text{weekly frequency}\;(\text{sessions}/\text{week}) \end{array}$$

For multicomponent or staged interventions, component- or stage-specific doses were calculated separately and then summed or weighted by duration. Active-control doses were estimated from the actual prescription, whereas usual care or no structured exercise was assigned 0 MET·min/week. Because completed dose was inconsistently reported, the primary analysis used planned prescription dose (Supplementary File 5, Section S5.1).

The standard definition of 1 MET as 3.5 mL O₂·kg^−1^·min^−1^ was used. MET values were assigned using a prespecified evidence hierarchy. Priority was given to values directly reported as METs or oxygen consumption, followed by estimates derived from exercise parameters using American College of Sports Medicine metabolic equations. The closest activity in the 2024 Adult Compendium of Physical Activities was used, followed by activity analogies, interpolation, or component-specific time weighting when necessary (Supplementary File 5, Section S5.2). Assignments reflected activity type, body-region involvement, active movement, task demands and training composition, rather than equipment name alone (Supplementary File 5, Section S5.3). For each arm, the original prescription, MET rationale, exact weekly dose, and final node were retained. Complete conversion rules and audit results are reported in Supplementary Tables 5.6A–5.7B.

Exact weekly doses were mapped to prespecified outcome-specific discrete nodes using the midpoints between adjacent nodes. These nodes were defined before model estimation to maintain network connectivity while reflecting the observed distribution of exact weekly doses. Because the exact doses were reconstructed from heterogeneously reported prescriptions and were sparsely distributed across study arms, mapping clinically comparable exposures reduced unwarranted precision and improved cross-study comparability and model stability.

For finger dexterity, nodes were 0, 180, 450, 700, and 1,600 MET·min/week, with boundaries of 90, 315, 575, and 1,150 MET·min/week. For upper-limb function, nodes were 0, 180, 450, 700, and 1,575 MET·min/week. Boundaries were 90, 315, 575, and 1,137.5 MET·min/week (Supplementary File 5, Section S5.4). The discrete node values served as inputs to the primary dose–response models. Exact doses were retained only to describe the original distribution and support auditing. If a non-zero active intervention mapped to the 0-dose node, it was reassigned to the lowest non-zero node in a sensitivity analysis. These doses are standardized cross-study exposure estimates, not direct measurements of energy expenditure or deterministic clinical prescriptions.

### 2.5 Risk-of-bias assessment

Two reviewers independently assessed risk of bias using the Cochrane Risk of Bias 2 tool (RoB 2) (26). Assessments were performed separately for finger dexterity and upper-limb function, focusing on the corresponding outcome at the end of intervention. Domains covered the randomization process, deviations from intended interventions, missing outcome data, outcome measurement and selection of the reported result. Responses to signalling questions informed domain-level and overall judgements of low risk, some concerns or high risk of bias. Disagreements were resolved by discussion, with adjudication by a third reviewer when necessary.

### 2.6 Bayesian network meta-analysis

Separate evidence networks for finger dexterity and upper-limb function were constructed, with exercise modalities represented as nodes. Usual care or no structured exercise (UC) was the reference. Hedges’ *g* provided a common effect scale, with positive values favoring exercise. Before modelling, distributions of age, disease severity, disease duration, medication status, intervention length, and exercise dose across direct comparisons were compared. These comparisons were used to assess transitivity.

The study fitted Bayesian random-effects network meta-analysis models using a normal likelihood and identity link. Relative treatment effects were assigned N(0, 5^2^) priors, and the between-study heterogeneity SD, τ, was assigned a U(0, 2) prior. Multi-arm trials were analyzed jointly within each model to preserve correlations induced by shared comparators. Posterior median estimates of Hedges’ *g*, 95% credible intervals (CrIs), and *P*(*g* > 0) were reported.

Each model used four Markov chains with 10,000 adaptation iterations. A further 20,000 iterations were discarded as burn-in, after which 50,000 iterations were sampled, retaining every fifth draw. Convergence was assessed using trace plots, the potential scale reduction factor (*R̂*) and effective sample size (ESS). Convergence was considered adequate when *R̂* ≤ 1.05 and ESS ≥ 200. Heterogeneity was summarized using the posterior median and 95% CrI for τ. Global inconsistency was assessed by comparing deviance information criterion (DIC) and residual deviance between consistency and unrelated mean effects (UME) models. Local inconsistency was evaluated using node splitting for estimable comparisons.

Intervention rankings were described using the surface under the cumulative ranking curve (SUCRA), mean rank and rank probabilities. Rankings were interpreted alongside relative effects, 95% CrIs, heterogeneity and network structure. For outcomes including at least 10 studies, comparison-adjusted funnel plots and Egger regression were used to explore small-study effects. Sensitivity analyses set the baseline-to-endpoint correlation to *R* = 0.50 or 0.90. Further analyses excluded studies at high risk of bias or with total sample sizes above the outcome-specific 90th percentile.

### 2.7 Dose–response network meta-analysis

MBNMAdose was used to perform Bayesian model-based network meta-analysis of dose–response relationships separately for each outcome. Model inputs were representative values of the mapped discrete dose nodes for each study arm, with UC assigned a dose of 0. Overall-exercise and modality-specific models were fitted. Doses were scaled by 1,000 MET·min/week during modeling, although results were reported in the original units. Exact weekly doses were used only to describe the original distribution, define the data-supported range and support auditing. Predictions between nodes represent model interpolation. Predictions above the highest node but within the exact-dose range represent limited extrapolation. Neither represents direct modeling of the original continuous doses.

Models used a normal likelihood and identity link, with Hedges’ *g* as the effect measure and random effects for the primary analyses. Candidate dose functions were linear, log-linear, exponential, quadratic, Emax, and natural cubic spline models. Spline knots were placed at the 25th, 50th, and 75th percentiles of the non-zero dose distribution. Overall-effect parameters were assigned N(0, 100^2^) priors, and dose–function parameters were assigned N(0, 2^2^) priors. Parameters constrained to be positive received corresponding truncated normal priors, and τ was assigned a U(0, 2) prior.

Each model used three Markov chains with 5,000 adaptation iterations. This study discarded 15,000 iterations as burn-in and sampled 30,000 iterations, retaining every tenth draw. Convergence was assessed using trace plots, posterior density plots, R̂ and ESS. Models were compared only when *R̂* ≤ 1.05 and ESS ≥ 200. Model selection considered DIC, residual deviance, predictive stability and parsimony. When ΔDIC ≤ 2, the model with fewer parameters and stable predictions was preferred. Node splitting assessed local inconsistency only for identifiable comparisons with both direct and indirect evidence.

At representative doses, posterior median estimates of Hedges’ *g*, 95% CrIs, and *P*(*g* > 0) are reported. The threshold dose was the first dose at which the interpolated mean curve reached the prespecified Hedges’ *g* threshold of 0.20 within the observed range. The minimum statistically supported dose was the first dose whose 95% CrI lower bound exceeded 0. The threshold-supported range satisfied both criteria. Also, *P*(*g* ≥ 0.20) at the threshold dose was reported. The maximum predicted effect, doses within 0.01 of this maximum and dose-specific rankings within the observed range were considered exploratory. Range-restricted maxima and near-maximum ranges do not identify an internal optimum or biological plateau. Rankings included only modalities whose observed dose ranges covered the target dose.

Dose–response sensitivity analyses set the baseline-to-endpoint correlation to *R* = 0.50 or 0.90. Additional analyses excluded studies at high risk of bias or with total sample sizes above the outcome-specific 90th percentile.

### 2.8 Subgroup analyses

This study conducted prespecified exploratory subgroup analyses at the study level for both outcomes. Stratification factors were intervention length (<8 versus ≥8 weeks), mean age (<65 versus ≥65 years), and outcome-assessment type. Finger-dexterity measures were classified as pegboard or non-pegboard tests. Upper-limb function measures were classified as objective or clinician-assessed versus patient- or goal-reported outcomes.

Conventional analyses fitted separate Bayesian random-effects network meta-analysis models within each subgroup, using UC as the reference. Between-subgroup differences were formally tested using treatment-by-subgroup interaction models in which all non-UC interventions shared one interaction coefficient. Overall dose–response analyses tested interactions between each subgroup variable and the selected dose–function parameter. The interaction involved the linear slope for finger dexterity and the exponential amplitude parameter for upper-limb function. Inference was restricted to the observed dose range shared by both subgroups.

Analyses were performed only when networks were connected, data were sufficient and models were identifiable. Conventional interaction coefficients were assigned Student-*t* priors with 1 degree of freedom and scale 2. Dose-interaction parameters were assigned N(0, 2^2^) priors, and τ was assigned a U(0, 2) prior. All subgroup models used four chains. Convergence required *R̂* ≤ 1.05, ESS ≥ 200 and a Monte Carlo standard error to posterior SD ratio ≤ 0.10. Between-subgroup differences were evaluated using posterior medians, 95% CrIs and directional posterior probabilities for the interaction parameters. This study did not infer interactions by comparing statistical significance within individual subgroups. All subgroup analyses were exploratory and study-level, and were not interpreted causally at the individual level.

All analyses were performed in R version 4.6.1. Bayesian network meta-analysis and dose–response network meta-analysis used gemtc version 1.0.2 and MBNMAdose version 0.5.0, respectively. Markov chain Monte Carlo sampling was performed using JAGS version 4.3.2.

### 2.9 CINeMA assessment of evidence certainty

This study assessed evidence certainty for the principal comparisons using the Confidence in Network Meta-Analysis (CINeMA) framework. Domains comprised within-study bias, reporting bias, indirectness, imprecision, heterogeneity and incoherence. Evidence certainty was rated as high, moderate, low, or very low. Conventional network meta-analysis assessed principal comparisons between exercise modalities. Dose–response analyses assessed only prespecified dose-node comparisons with UC within the observed dose range.

Following Cohen’s effect-size conventions and Cochrane methodological guidance, a favorable Hedges’ *g* of 0.20 was prespecified as an exploratory threshold for clinical importance (27, 28). This common threshold informed clinical interpretation and CINeMA assessments of imprecision and heterogeneity in both analyses. In dose–response analyses, it also defined the exploratory threshold dose and threshold-supported range. This value does not replace an outcome- and population-specific minimal clinically important difference and was not used to define a recommended clinical dose (29, 30).

## 3 Results

### 3.1 Study selection

Database searches identified a total of 12,147 records for screening. After removing 7,909 duplicates, 4,238 records underwent title and abstract screening. Of these, 4,052 were excluded because of ineligible publication type (*n* = 904), non-randomized design (*n* = 807), ineligible population (*n* = 696) or ineligible exercise intervention (*n* = 1,645). Full texts were retrieved for all remaining 186 reports and assessed for eligibility.

After full-text assessment, 150 reports were excluded for the following reasons. These comprised ineligible comparators (*n* = 43), no eligible upper-limb or hand-function outcome (*n* = 61), and duplicate publication or overlapping populations (*n* = 8). Insufficient or non-extractable outcome data accounted for the remaining 38 reports.

The 36 included reports comprised 75 study arms, including 39 exercise arms and 36 control or comparator arms. Three reports described three-arm trials, and 20 included direct comparisons between exercise modalities or doses. The study-selection process and reasons for full-text exclusion are shown in Figure 1.

**Figure 1:**
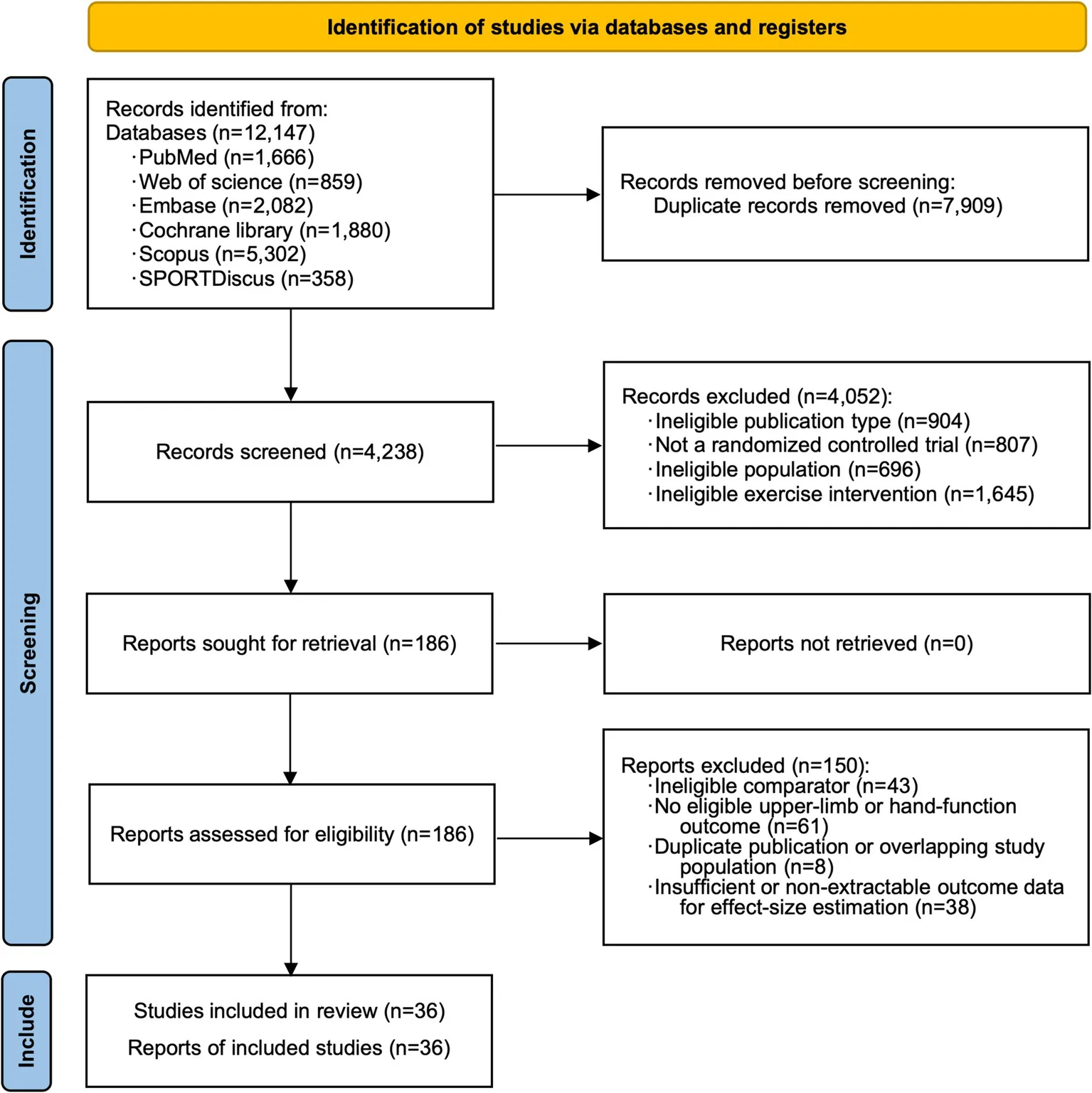
PRISMA flow diagram of study selection.

### 3.2 Study characteristics

The 36 reports, published between 2005 and 2026, included 1,575 participants with PD, with sample sizes ranging from 6 to 130. Mean participant age was 65.1 years, with study means ranging from 58.6 to 72.9 years. Twenty-seven reports provided sex distributions (*n* = 1,314), of whom 59.5% were men. Table 1 summarizes the principal characteristics of the included studies.

**Table 1:** Characteristics of the included randomized controlled trials.

Study	Mean age (years)	Male, *n* (%)	Total sample size (*n*)	Completion rate (%)	Intervention duration	Intervention frequency	Session duration	Intervention-group treatment	Comparator-group treatment
Allen 2017 (42)	67.95	23 (60.5%)	38	97.4%	12 weeks	3 sessions/week	45 min	UE exergames	Usual care and continued with their usual activities
Cabrera-Martos 2018 (43)	70.62	31 (62.0%)	50	100.0%	4 weeks	2 sessions/week	45 min	Intervention focused on task components that involved goals proposed by participants	Standard intervention focused on impairments in range of motion, grasp, and manipulation
Choi 2022 (44)	71.22	9 (64.3%)	14	100.0%	4 weeks	5 sessions/week	40 min	Task-based LSVT-BIG program	Relaxation and stretching
Fernández-González 2019 (45)	66.65	11 (47.8%)	23	100.0%	6 weeks	2 sessions/week	30 min	treatment based on serious games designed by the research team, using the LMC system for the UL	A specific intervention for the UL
Hashemi 2022 (46)	63.64	29 (64.4%)	45	100.0%	8 weeks	3 sessions/week	90 min	Supervised ULVRE or non-supervised ULVRE	Conventional rehabilitation exercises
Horin 2019 (47)	64.15	26 (65.0%)	40	92.5%	12 weeks	7 sessions/week	30 min	mHealth application	Maintained their normal routine
Park 2021 (48)	62.35	NR	16	75.0%	4 weeks	3 sessions/week	50 min	Drum playing intervention with rhythmic cueing	Regular programs
Taghizadeh 2017 (49)	60.10	35 (87.5%)	40	100.0%	2 weeks	5 sessions/week	120 min	Sensory-motor training (SMT)	Common rehabilitation therapies
Zare 2022 (50)	64.45	20 (50.0%)	40	82.5%	6 weeks	5 sessions/week	120 min	Activity-based training	Strengthening exercises
Vorasoot 2019 (51)	68.13	25 (54.3%)	46	100.0%	4 weeks	7 sessions/week	45 min	Handwriting exercise using a newly developed handwriting practice book	Control group
Vanbellingen 2017 (52)	67.65	63 (61.2%)	103	89.3%	4 weeks	5 sessions/week	30 min	Home-based dexterity program (HOMEDEXT)	Thera-band program
Raciti 2022 (53)	64.58	NR	30	80.0%	8 weeks	6 sessions/week	90 min	Armeo®Spring (a gravity-supporting device)	Conventional physical therapy (CPT)
Collett 2017 (54)	66.49	61 (58.1%)	105	84.8%	24 weeks	2 sessions/week	60 min	Self-managed community exercise program	Handwriting
van der Kolk 2019 (32)	64.35	80 (61.5%)	130	96.2%	24 weeks	3 sessions/week	30 min	aerobic exercise done on a stationary home-trainer	Stretching (active control group)
Çetin 2024 (55)	67.50	13 (65.0%)	20	87.0%	8 weeks	3 sessions/week	50 min	Exergames Program (EP)	Conventional Exercise Program (CP)
Nackaerts 2017 (56)	63.13	23 (60.5%)	38	100.0%	6 weeks	5 sessions/week	30 min	Training focused at improving writing size	Stretch and relaxation exercises
Kadkhodaie 2020 (57)	67.62	NR	27	77.8%	6 weeks	3 sessions/week	40 min	Upper limb pure eccentric training	No additional exercise
Duncan 2012 (58)	69.15	35 (56.5%)	62	83.9%	48 weeks	2 sessions/week	60 min	Community-based Argentine Tango program	Control group (no intervention)
Robles-García 2016 (59)	66.67	NR	16	93.8%	4 weeks	3 sessions/week	30 min	Movement imitation training	Self-regulated duty cycle
van der Kolk 2017 (35)	63.88	24 (64.9%)	37	94.6%	24 weeks	3 sessions/week	30 min	Aerobic exercise	No intervention (control)
Marusiak 2019 (60)	72.85	9 (45.0%)	20	100.0%	8 weeks	3 sessions/week	40 min	8-week moderate intensity AIT program	Usual care
Schaeffer 2019 (61)	62.97	21 (52.5%)	40	100.0%	10 weeks	1 session/week	60 min	Occupational therapy	Control: no intervention
Demirgöz Demir 2025 (62)	65.70	25 (55.6%)	45	100.0%	4 weeks	5 sessions/week	120 min	conventional rehabilitation program + robot-assisted arm training; conventional rehabilitation + robot-assisted arm training + recreational activities	Conventional rehabilitation program
Heinzle 2026 (63)	69.00	39 (67.2%)	58	98.3%	4 weeks	4 sessions/week	30 min	Immersive virtual reality upper-limb training	Conventional upper-limb training
Raciti 2025 (64)	60.80	19 (47.5%)	40	100.0%	8 weeks	6 sessions/week	90 min	Robotic-assisted therapy (RAT)	Conventional physical therapy (CPT)
Li 2024 (65)	63.04	NR	60	90.0%	4 weeks	5 sessions/week	30 min	Baduanjin exercise	Usual physical activities
Caglar 2005 (66)	65.85	21 (70.0%)	30	100.0%	8 weeks	7 sessions/week	60 min	A schedule of exercises to undertake at home	Did not receive this instruction
Chen 2023 (67)	68.33	NR	39	93.5%	14 weeks	1 session/week	120 min	Archery exercises	Standard care
Eldemir 2023 (68)	64.64	19 (63.3%)	30	93.8%	6 weeks	3 sessions/week	60 min	Task-oriented circuit training-based telerehabilitation (TOCT-TR)	Home exercises aimed at improving balance, gait, and mobility
Moraes 2024 (69)	66.35	36 (51.4%)	70	100.0%	24 weeks	2 sessions/week	60 min	Global exercise training	Handwriting upper limb exercise training
Mishra 2022 (70)	64.62	NR	6	100.0%	3 weeks	3 sessions/week	50 min	Motor imagery combined with conventional treatment	Conventional treatment consisting of flexibility and strength training
Lorek 2025 (71)	65.61	NR	30	93.3%	12 weeks	3 sessions/week	40 min	Moderate-intensity AIT program	Usual care, including conventional physical therapy
Pazzaglia 2020 (72)	71.00	35 (68.6%)	51	100.0%	6 weeks	3 sessions/week	40 min	Virtual reality rehabilitation program	Conventional rehabilitation program
Leal 2019 (73)	65.05	27 (50.0%)	54	100.0%	24 weeks	2 sessions/week	40 min	Resistance training	Maintained their pharmacological treatments
Zhang 2026 (74)	63.50	23 (51.1%)	45	100.0%	14 weeks	4 sessions/week	60 min	Tai Chi group; combined resistance-stretching group	Normal care; health education sessions
Martinazzo 2025 (75)	61.70	NR	34	67.6%	12 weeks	2 sessions/week	60 min	Mat Pilates	Control group (GC)

Conventional network meta-analyses included 30 reports on finger dexterity (60 arms; *n* = 1,233) and 23 on upper-limb function (46 arms; *n* = 842). Further details are provided in Supplementary Tables 5.6A, 5.6B. Both connected networks contained seven active interventions (AET, CULR, MBE, MCT, RT, TAULHT, and TOFDT) and UC, forming eight nodes. Together, the networks comprised 12 and 10 direct comparisons, respectively. CULR versus TAULHT was the most frequently studied direct comparison, with seven reports in each network (Figure 2).

**Figure 2:**
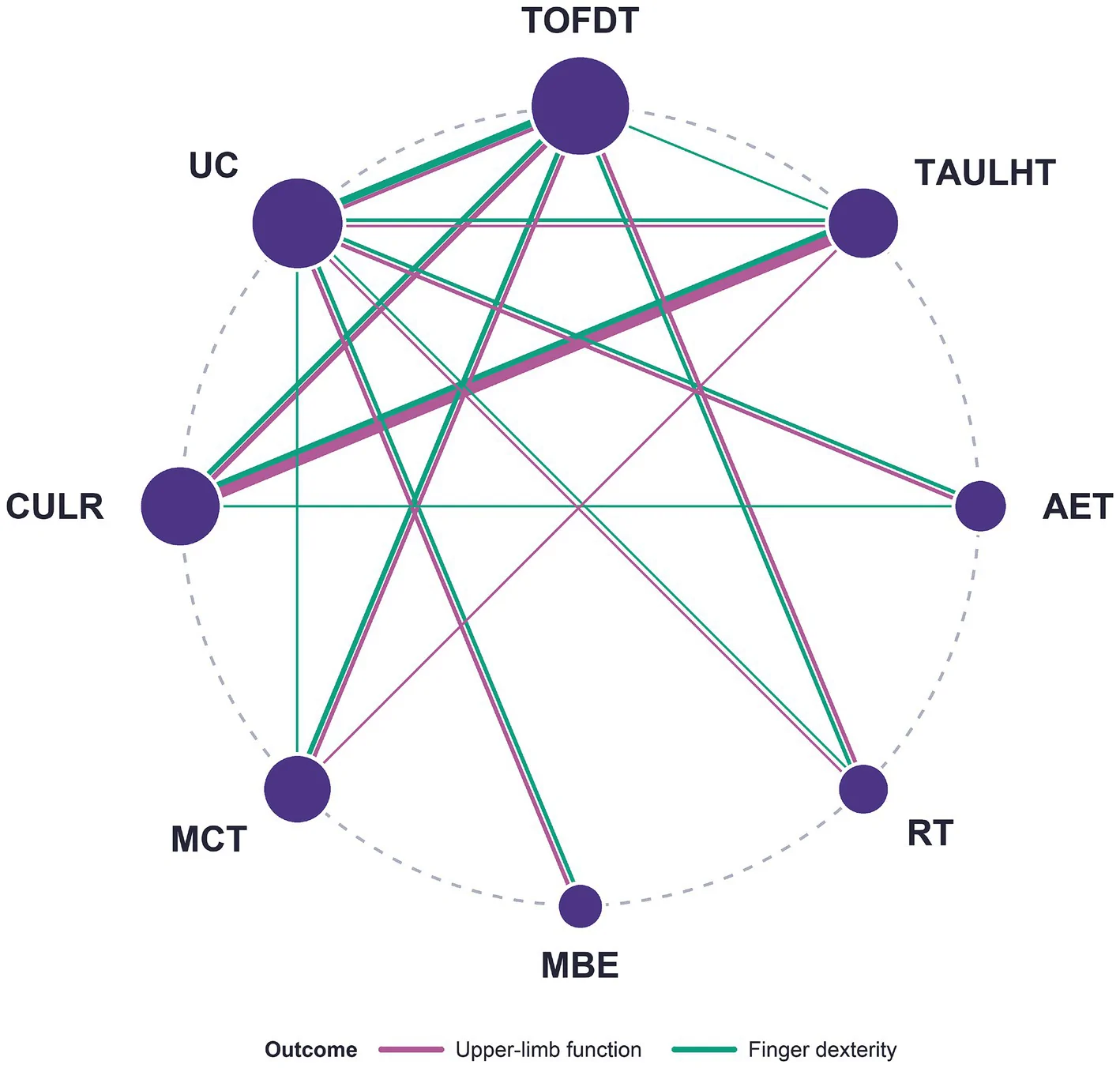
Evidence networks of exercise interventions for finger dexterity and upper-limb function. Green edges denote finger dexterity and magenta edges upper-limb function. Node size and edge width indicate participant numbers and direct comparisons, respectively. AET, aerobic exercise training; CULR, conventional upper-limb rehabilitation; MBE, mind–body exercise; MCT, multicomponent training; RT, resistance training. TAULHT, technology-assisted upper-limb or hand training; TOFDT, task-oriented finger dexterity training; UC, usual care or no structured exercise.

Exercise prescriptions varied substantially across studies, intervention types, and assessed outcomes. Intervention length ranged from 2 to 48 weeks, frequency from 1 to 7 sessions per week and session duration from 30 to 120 min. Most non-zero-dose arms prescribed low-to-moderate-intensity exercise in the included studies. Exact weekly doses ranged from 120 to 1,800 MET·min/week for both outcomes. Median doses were 460.0 MET·min/week for finger dexterity and 466.3 MET·min/week for upper-limb function.

For modeling, exact doses were mapped to outcome-specific discrete nodes. Finger-dexterity nodes were 0, 180, 450, 700, and 1,600 MET·min/week. Upper-limb-function nodes were 0, 180, 450, 700, and 1,575 MET·min/week. Supplementary File 5 provides complete dose assignments, conversions, node mapping, and arm-level data.

Finger dexterity was most often assessed using the Nine-Hole Peg Test (9HPT; 16 reports) and Purdue Pegboard Test (PPT; 5 reports). Upper-limb function was most often assessed using the Box and Block Test (BBT; 5 reports) and handgrip strength (HGS; 3 reports). Other measures included FMA-UE, JTHFT, DASH, other pegboard tests, kinematic measures, and patient-reported outcomes. All outcomes were assessed at the end of intervention, which occurred 2–48 weeks after baseline for finger dexterity and 2–24 weeks for upper-limb function. These ranges reflected differences in intervention duration across studies (Supplementary Tables 5.6A, 5.6B).

### 3.3 Risk-of-bias assessment

RoB 2 assessments classified 21 of the 36 included reports (58.3%) as low overall risk, 10 (27.8%) as having some concerns and 5 (13.9%) as high risk. Overall, low-risk judgements predominated across all assessed RoB 2 domains. The outcome-measurement domain had the highest proportion of low-risk judgements (32 reports, 88.9%).

Risk of bias arose mainly from deviations from intended interventions and missing outcome data. For deviations from intended interventions, 9 reports (25.0%) had some concerns and 1 (2.8%) was at high risk. For missing outcome data, 5 reports (13.9%) had some concerns and 4 (11.1%) were at high risk. Domain-level judgements and their distributions are shown in Supplementary Figures 6A–C.

### 3.4 Relative effects of exercise modalities

#### 3.4.1 Finger dexterity

In the finger-dexterity network of 30 reports, conventional Bayesian network meta-analysis compared seven active exercise or rehabilitation interventions with UC (Figure 2). High-certainty evidence showed that AET improved finger dexterity relative to UC (Hedges’ *g* = 1.460, 95% CrI 0.260–2.685; *P*(*g* > 0) = 0.991). TOFDT also improved finger dexterity (Hedges’ *g* = 0.956, 95% CrI 0.233–1.671; *P*(*g* > 0) = 0.994). Estimates for the other five interventions generally favored benefit, but all 95% CrIs included 0 (Figure 3; Supplementary Tables 7.1A, 7.2A).

**Figure 3:**
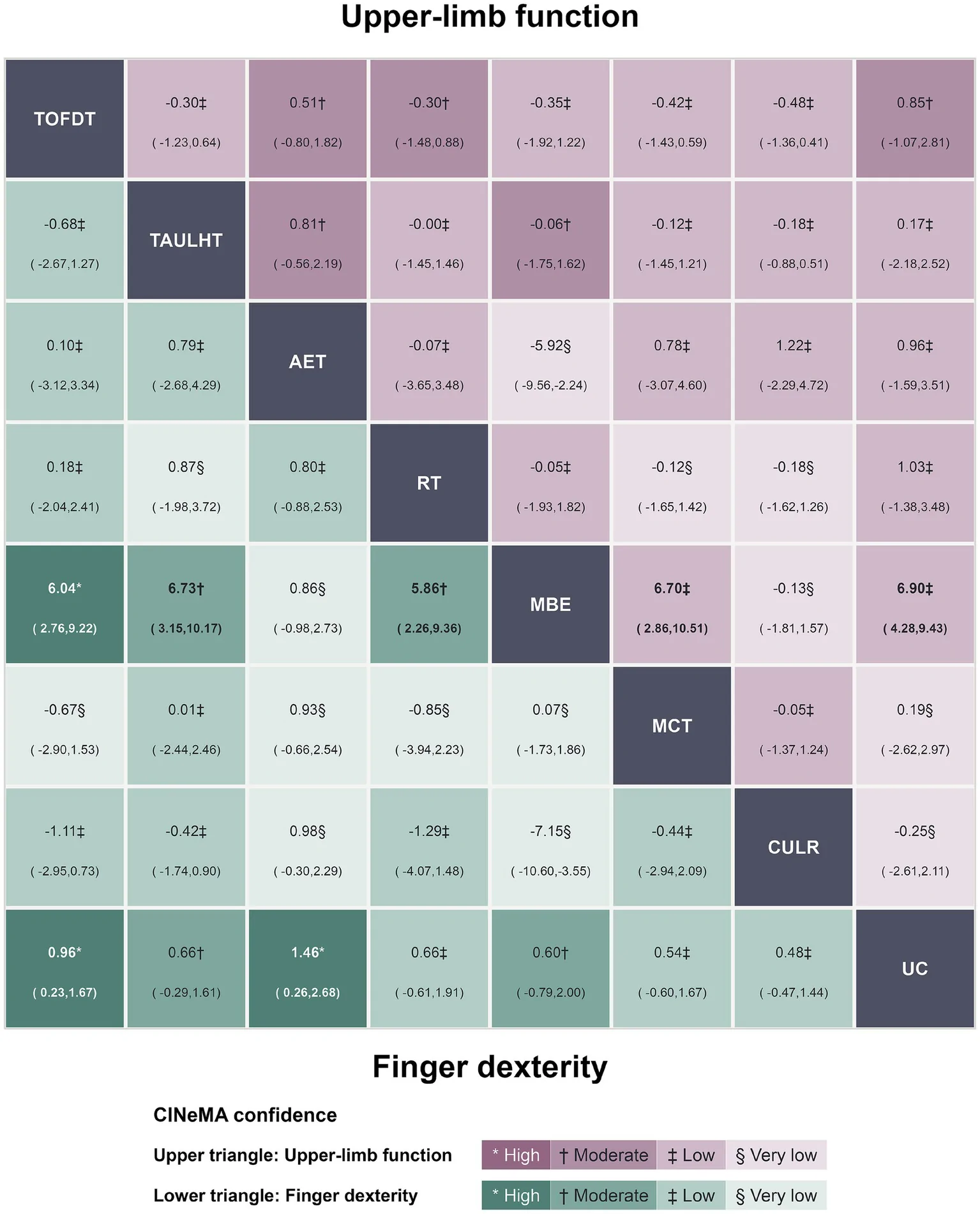
League table of relative effects and CINeMA confidence. The upper triangle (purple shades) presents upper-limb function, and the lower triangle (green shades) presents finger dexterity. Values are posterior median Hedges’ *g* (95% CrI); bold indicates CrIs excluding zero. Positive values favor the row intervention in the upper triangle and the column intervention in the lower triangle. Within each triangle, color intensity and symbols indicate CINeMA confidence: * high, † moderate, ‡ low, and § very low. Darker shades indicate higher confidence. Intervention abbreviations are defined in Figure 2; CrI, credible interval.

Posterior rankings placed AET first (SUCRA = 0.876; mean rank = 1.87; probability of being best = 0.616). This ranking does not establish that AET was more effective than other active interventions. Complete rankings are provided in Supplementary Table 7.1A and Supplementary Figure 7.1A.

Estimated between-study heterogeneity was τ = 0.931 (95% CrI 0.673–1.341; Supplementary Table 7.3). All fitted models met the prespecified criteria for satisfactory convergence. The unrelated mean effects model did not materially improve fit over the consistency model (DIC, 118.354 versus 117.603; ΔDIC = 0.752). Model-fit results are provided in Supplementary Tables 7.4A, 7.4B and Supplementary Figure 7.4A. Among 11 estimable node-split comparisons, the minimum *p* value was 0.171, providing no clear evidence of local inconsistency. Detailed results are shown in Supplementary Table 7.5A and Supplementary Figure 7.5A.

#### 3.4.2 Upper-limb function

In the upper-limb-function network of 23 reports, conventional Bayesian network meta-analysis compared seven active exercise or rehabilitation interventions with UC (Figure 2). Low-certainty evidence suggested that MBE may improve upper-limb function relative to UC (Hedges’ *g* = 6.897, 95% CrI 4.284–9.426; *P*(*g* > 0) > 0.999). The 95% CrIs for the other six interventions included 0 (Figure 3; Supplementary Tables 7.1B, 7.2B). This comparison was informed by only two small studies involving 53 participants in the relevant comparison arms, indicating that the evidence base was limited.

Posterior rankings placed MBE first (SUCRA = 1.000; mean rank = 1.00; probability of being best = 0.998). This ranking does not establish that MBE was more effective than other active interventions. Complete rankings are provided in Supplementary Table 7.1B and Supplementary Figure 7.1B.

Estimated between-study heterogeneity was τ = 1.901 (95% CrI 1.620–1.996; Supplementary Table 7.3). All fitted models met the prespecified criteria for satisfactory convergence. The unrelated mean effects model did not materially improve fit over the consistency model (DIC, 92.339 versus 92.196; ΔDIC = 0.143). Model-fit results are provided in Supplementary Tables 7.4A, 7.4B and Supplementary Figure 7.4B. Among eight estimable node-split comparisons, the minimum *p* value was 0.848, providing no clear evidence of local inconsistency. Detailed results are shown in Supplementary Table 7.5B and Supplementary Figure 7.5B.

### 3.5 Overall exercise dose–response relationships

#### 3.5.1 Finger dexterity

The overall dose–response analysis for finger dexterity included 23 reports and 29 distinct non-zero exact weekly doses from the original prescriptions. Observed doses ranged from 120 to 1,800 MET·min/week. Exact doses were mapped to prespecified nodes, whose representative values entered the models (Supplementary Table 8.1A and Supplementary Figure 8.1A). All candidate models considered in the comparison met the prespecified convergence criteria.

The random-effects Emax model had the lowest DIC (38.22), whereas the linear random-effects model had a DIC of 38.48. The two models therefore provided similarly close fits to the data (ΔDIC = 0.26). Considering model fit, parameter count, and predictive stability, the linear random-effects model as the primary model (τ = 0.865) was selected. Further details are provided in Supplementary Table 8.2A and Supplementary Figure 8.2A. Dose-node contributions to overall model deviance are shown in Supplementary Figure 8.2C.

The primary model showed an approximately linear increase in finger-dexterity benefit with increasing overall exercise dose. At 600 MET·min/week, the predicted effect was Hedges’ *g* = 0.578 (95% CrI 0.195–0.965). At 1,200 MET·min/week, the corresponding predicted effect was Hedges’ *g* = 1.157 (95% CrI 0.391–1.931). At 1,800 MET·min/week, the predicted effect was Hedges’ *g* = 1.735 (95% CrI 0.586–2.896). All three doses had *P*(*g* > 0) = 0.997 (Figure 4; Supplementary Tables 8.3A, 8.3B).

**Figure 4:**
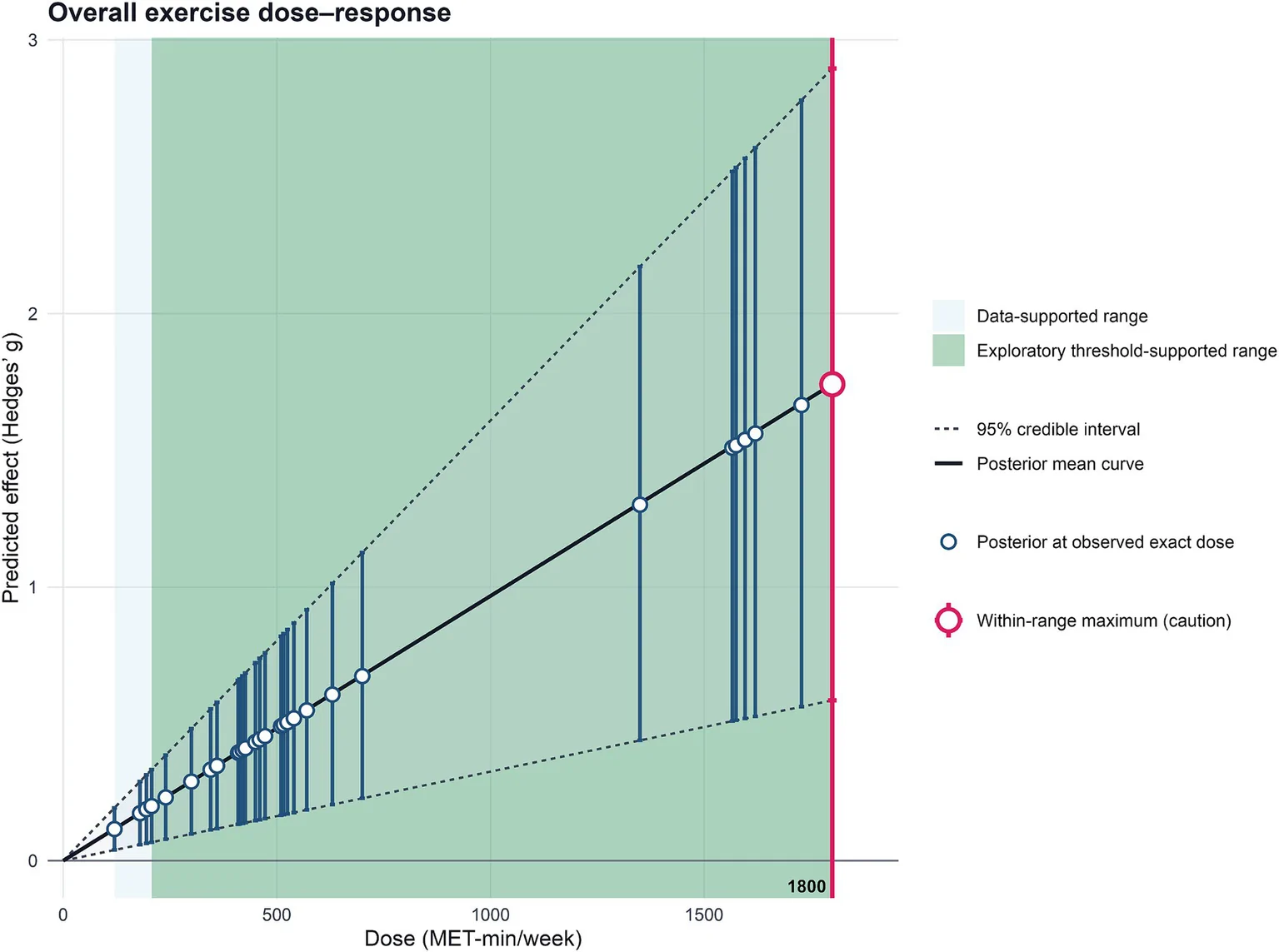
Model-based overall exercise dose–response relationship for finger dexterity. The black curve and dotted bounds show posterior mean Hedges’ *g* estimates and 95% CrIs. Blue points show estimates at observed doses; blue and green shading mark the data-supported and threshold-supported ranges. Magenta marks the within-range maximum, not an optimal dose. CrI, credible interval; MET, metabolic equivalent of task.

The maximum predicted effect within the observed dose range occurred at its upper boundary. The near-maximum range was 1,790–1,800 MET·min/week, with substantial uncertainty at the high-dose end. These estimates should not be interpreted as identifying an optimal dose or recommended prescription. CINeMA certainty for prespecified dose-node comparisons with UC ranged from low to moderate (Supplementary Table 8.6A).

#### 3.5.2 Upper-limb function

The overall dose–response analysis for upper-limb function included 15 reports and 20 distinct non-zero exact weekly doses from the original prescriptions. Observed doses ranged from 120 to 1,800 MET·min/week. Exact doses were mapped to prespecified nodes, whose representative values entered the models (Supplementary Table 8.1B and Supplementary Figure 8.1B). All candidate models considered in the comparison met the prespecified convergence criteria.

The random-effects Emax model had the lowest DIC (27.94), whereas the exponential random-effects model had a DIC of 28.50. The two models therefore provided similarly close fits to the data (ΔDIC = 0.56). Considering model fit, parameter count, and predictive stability, the exponential random-effects model as the primary model (τ = 1.951) was selected. Further details are provided in Supplementary Table 8.2C and Supplementary Figure 8.2F. Dose-node contributions to overall model deviance are shown in Supplementary Figure 8.2H.

The primary model showed increasing upper-limb-function benefit with higher overall exercise doses, although the incremental gain progressively diminished. At 600 MET·min/week, the predicted effect was Hedges’ *g* = 1.722 (95% CrI 0.658–2.795). At 1,200 MET·min/week, the corresponding predicted effect was Hedges’ *g* = 2.667 (95% CrI 1.020–4.329). At 1,800 MET·min/week, the predicted effect was Hedges’ *g* = 3.186 (95% CrI 1.218–5.171). All three doses had *P*(*g* > 0) > 0.999 (Figure 5; Supplementary Tables 8.3A, 8.3B).

**Figure 5:**
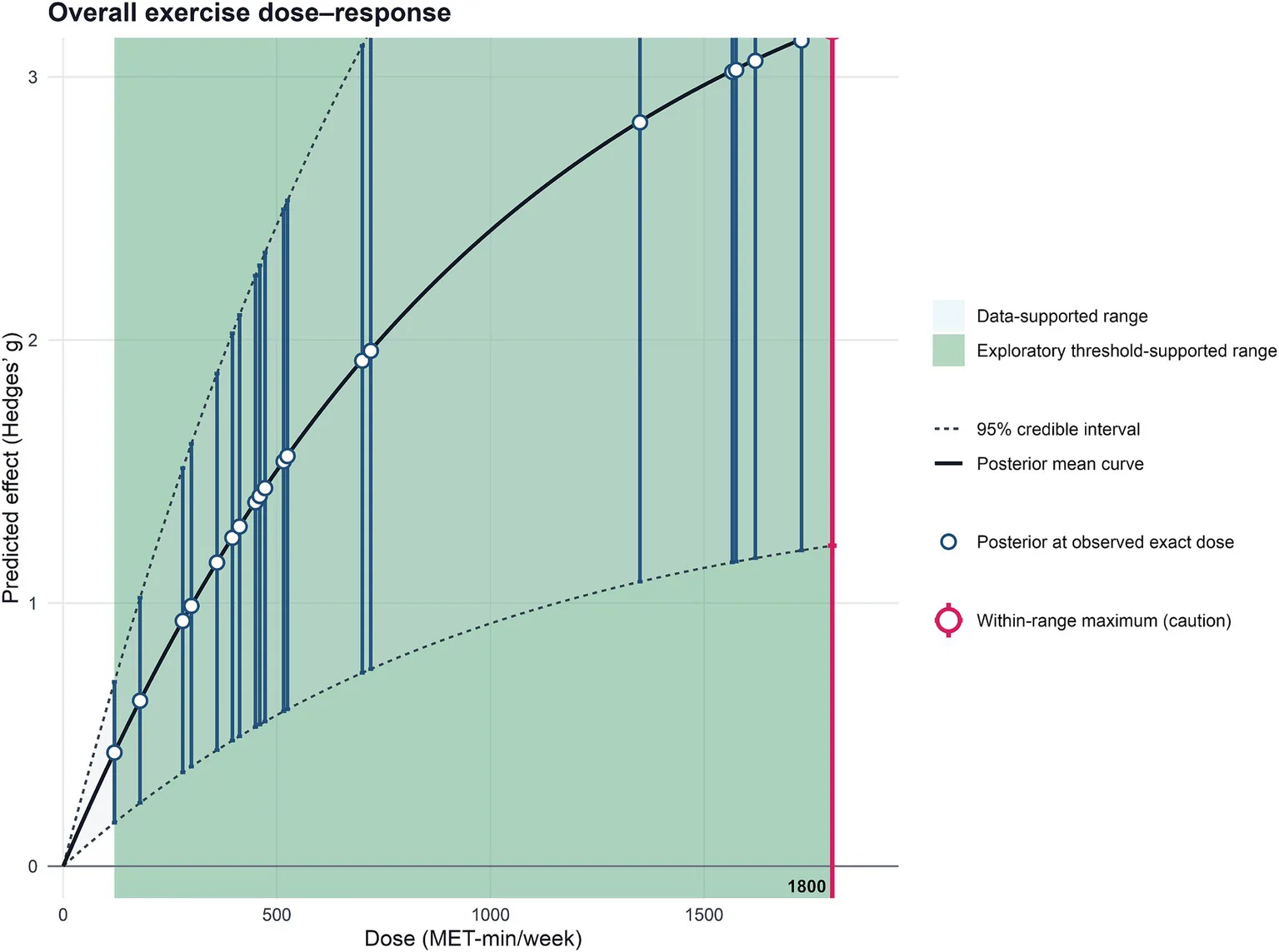
Model-based overall exercise dose–response relationship for upper-limb function. The black curve and dotted bounds show posterior mean Hedges’ *g* estimates and 95% CrIs. Blue points show estimates at observed doses; blue and green shading mark the data-supported and threshold-supported ranges. Magenta marks the within-range maximum, not an optimal dose. CrI, credible interval; MET, metabolic equivalent of task.

The maximum predicted effect within the observed dose range occurred at its upper boundary. The near-maximum range was 1,790–1,800 MET·min/week, with substantial uncertainty at the high-dose end. These estimates should not be interpreted as identifying an optimal dose or recommended prescription. CINeMA certainty for prespecified dose-node comparisons with UC ranged from low to moderate (Supplementary Table 8.6B).

### 3.6 Modality-specific exercise dose–response relationships

#### 3.6.1 Finger dexterity

The modality-specific analysis used a linear random-effects model (τ = 0.955; Supplementary Table 8.2B and Supplementary Figure 8.2A). Correspondence between observed and posterior-predicted effects and modality-specific deviance contributions are shown in Supplementary Figures 8.2B, 8.2D. TOFDT showed the clearest positive dose–response relationship among the examined modalities. At 1,800 MET·min/week, its predicted effect was Hedges’ *g* = 1.863 (95% CrI 0.316–3.466; *P*(*g* > 0) = 0.991). The 95% CrIs for all other modalities included 0 in this analysis (Figure 6; Supplementary Table 8.4A).

**Figure 6:**
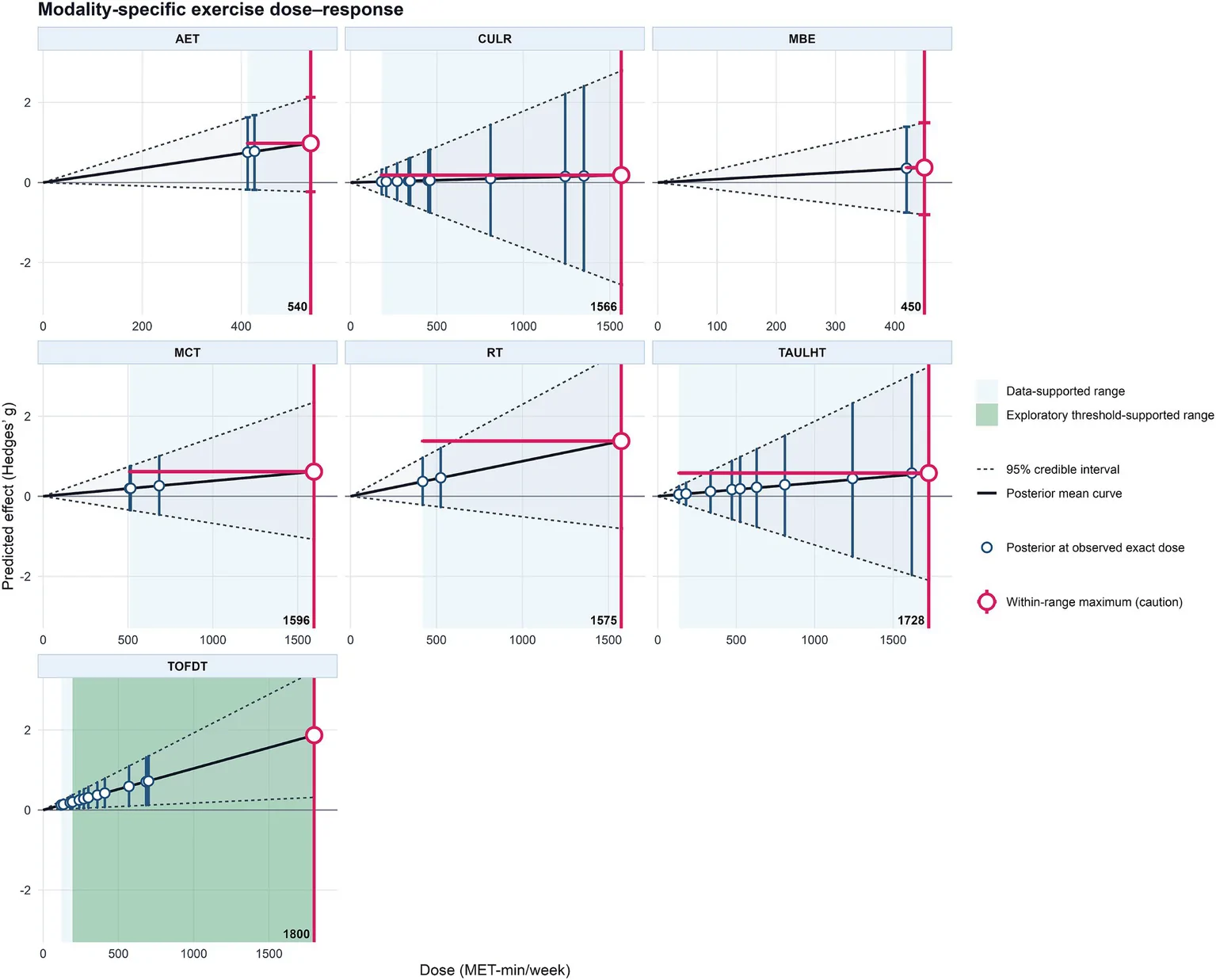
Model-based modality-specific exercise dose–response relationships for finger dexterity. Black curves and dotted bounds show posterior mean Hedges’ *g* estimates and 95% CrIs. Blue points show estimates at observed doses. Blue and green shading mark data-supported and threshold-supported ranges; magenta marks within-range maxima, not optimal doses. Intervention abbreviations are defined in Figure 2. CrI, credible interval; MET, metabolic equivalent of task.

The maximum predicted TOFDT effect occurred at the upper observed-dose boundary and did not identify an optimal dose or recommended prescription. Node splitting identified nominal local inconsistency only for AET at the 450 MET·min/week node versus UC (*p* = 0.002). The original exact dose for this node was 412.8 MET·min/week (Supplementary Table 8.1A and Supplementary Figure 8.2E).

At data-supported doses of 300, 600, 900, and 1,200 MET·min/week, TOFDT had the highest dose-specific SUCRA (0.78–0.83). However, different modalities contributed to the rankings at each dose, so these results were interpreted descriptively (Supplementary Figure 8.5A). CINeMA certainty for modality-specific dose comparisons ranged from very low to moderate (Supplementary Table 8.6A).

#### 3.6.2 Upper-limb function

The modality-specific analysis used a linear random-effects model (τ = 1.884; Supplementary Table 8.2D and Supplementary Figure 8.2F). Correspondence between observed and posterior-predicted effects and modality-specific deviance contributions are shown in Supplementary Figures 8.2G, 8.2I. MBE showed the clearest positive dose–response relationship among the examined modalities. At 720 MET·min/week, its predicted effect was Hedges’ *g* = 4.912 (95% CrI 2.595–7.209; *P*(*g* > 0) > 0.999). The 95% CrIs for all other modalities included 0 in this analysis (Figure 7; Supplementary Table 8.4B).

**Figure 7:**
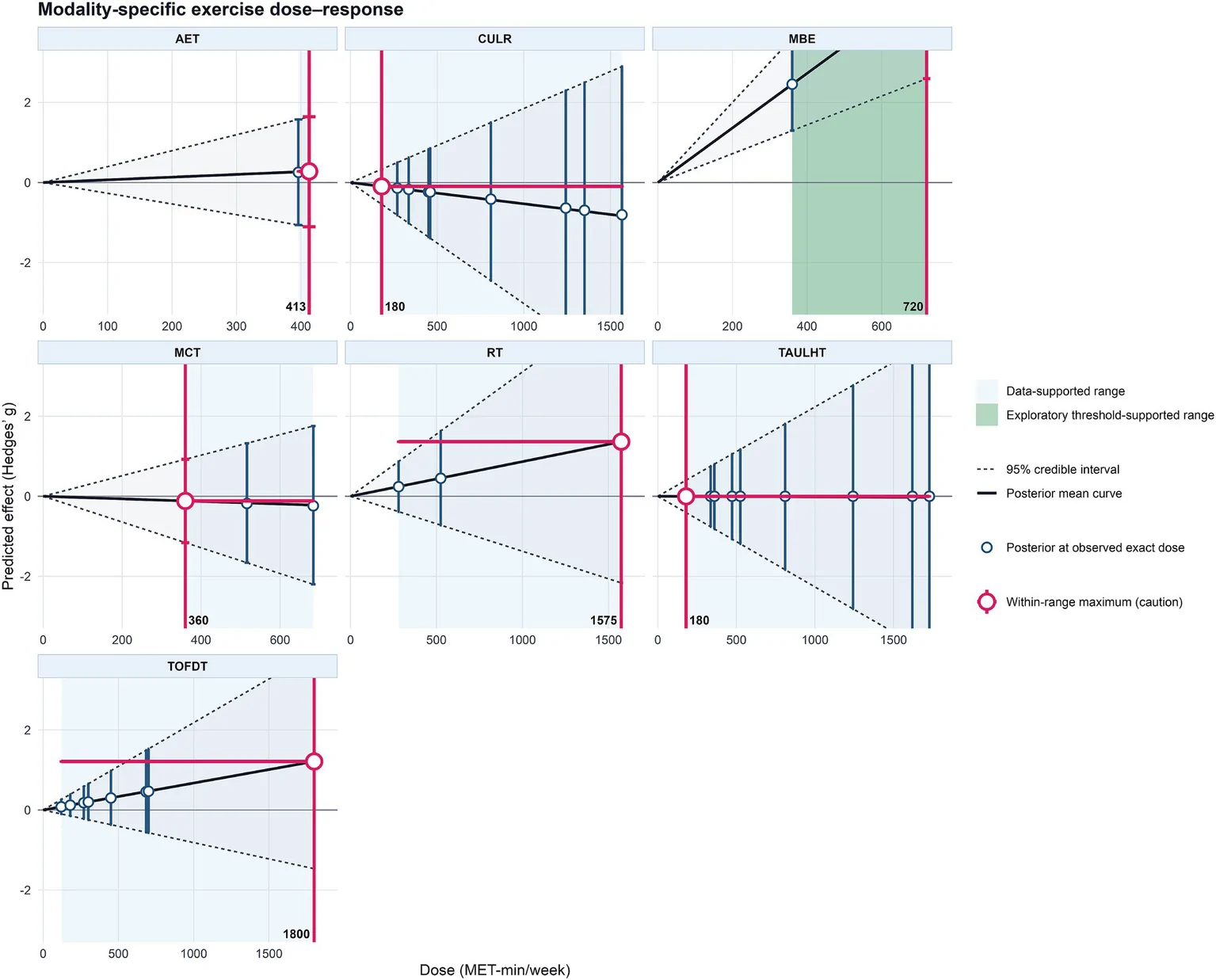
Model-based modality-specific exercise dose–response relationships for upper-limb function. Black curves and dotted bounds show posterior mean Hedges’ *g* estimates and 95% CrIs. Blue points show estimates at observed doses. Blue and green shading mark data-supported and threshold-supported ranges; magenta marks within-range maxima, not optimal doses. Intervention abbreviations are defined in Figure 2. CrI, credible interval; MET, metabolic equivalent of task.

Only two studies supported the MBE estimate, whose maximum predicted effect occurred at the upper observed-dose boundary. This range-restricted maximum did not identify an optimal dose or recommended prescription. Node splitting indicated local inconsistency for MBE at the 700 MET·min/week node versus UC (*p* < 0.001). The original exact dose for this node was 720 MET·min/week (Supplementary Table 8.1B and Supplementary Figure 8.2J).

At the data-supported dose of 600 MET·min/week, MBE had the highest dose-specific SUCRA (1.000). However, different modalities contributed to rankings at different doses, so this result was interpreted descriptively (Supplementary Figure 8.5B). CINeMA certainty for modality-specific dose comparisons ranged from very low to moderate (Supplementary Table 8.6B).

### 3.7 Exercise dose and the exploratory Hedges’ *g* threshold of 0.20

#### 3.7.1 Finger dexterity

The overall dose–response curve became statistically supported from 120 MET·min/week and reached the exploratory Hedges’ *g* threshold of 0.20 at 207 MET·min/week. At this dose, the predicted effect was Hedges’ *g* = 0.200 (95% CrI 0.067–0.333), with *P*(*g* > 0) = 0.997. The corresponding *P*(*g* ≥ 0.20) was 0.496, and the threshold-supported range was 207–1,800 MET·min/week (Supplementary Table 8.7 and Supplementary Figure 8.7A).

In modality-specific analyses, only TOFDT met both statistical support and the Hedges’ *g* threshold within its observed dose range. Its minimum statistically supported dose was 120 MET·min/week, and the curve reached the threshold at 195 MET·min/week. At this threshold, the predicted effect was Hedges’ *g* = 0.202 (95% CrI 0.034–0.375), with *P*(*g* > 0) = 0.991 and *P*(*g* ≥ 0.20) = 0.510. The threshold-supported range was 195–1,800 MET·min/week. No other modality met both criteria within its observed dose range (Supplementary Table 8.7).

#### 3.7.2 Upper-limb function

The overall dose–response curve was statistically supported and exceeded the exploratory Hedges’ *g* threshold at the lowest data-supported dose of 120 MET·min/week. At this dose, the predicted effect was Hedges’ *g* = 0.432 (95% CrI 0.165–0.700), with *P*(*g* > 0) > 0.999. The corresponding *P*(*g* ≥ 0.20) was 0.955, and the threshold-supported range was 120–1,800 MET·min/week (Supplementary Table 8.7 and Supplementary Figure 8.7B).

In modality-specific analyses, only MBE met both statistical support and the Hedges’ *g* threshold. Its minimum statistically supported dose and threshold dose were both 360 MET·min/week. At this dose, the predicted effect was Hedges’ *g* = 2.456 (95% CrI 1.298–3.604), with *P*(*g* > 0) and *P*(*g* ≥ 0.20) both exceeding 0.999. The threshold-supported range for MBE was 360–720 MET·min/week. No other modality met both criteria within its observed dose range (Supplementary Table 8.7).

Dose thresholds were defined by where the posterior mean curve reached a Hedges’ *g* of 0.20, whereas effects and 95% CrIs were reported as posterior medians. A Hedges’ *g* of 0.20 was an exploratory effect threshold, not a validated minimal clinically important difference. These estimates locate where the dose–response curves reached a prespecified effect level and should not be interpreted as recommended clinical doses.

### 3.8 Exploratory subgroup analyses

In conventional Bayesian network meta-analysis, subgroup-specific treatment rankings for finger dexterity varied by intervention length, mean age, and outcome-assessment type. Formal interaction analysis identified a positive interaction only for assessment type. Treatment effects were larger for non-pegboard than pegboard tests (β = 1.343, 95% CrI 0.058–2.680; *P*(β > 0) = 0.980). Intervention length and mean age showed no clear effect modification. For upper-limb function, all interaction estimates for the three subgroup factors included 0, indicating no clear effect modification. Complete subgroup rankings and interaction results are provided in Supplementary Tables 7.6A–7.6D.

Bayesian dose–response network meta-analysis showed no clear interaction of intervention length or mean age with the finger-dexterity dose–response relationship. Within the shared data-supported range of 180–516 MET·min/week, dose-related effects were larger for non-pegboard than pegboard tests. The interaction estimate was β = 2.84 Hedges’ *g* per 1,000 MET·min/week (95% CrI 0.99–4.48; *P*(β > 0) = 0.998).

For upper-limb function, interventions lasting ≥8 weeks showed a positive dose-by-subgroup interaction signal relative to interventions lasting <8 weeks. The interaction estimate was β = 3.56 (95% CrI 0.42–6.66; *P*(β > 0) = 0.987). The mean age and outcome-assessment type showed no clear interactions in this analysis. These estimates remained affected by few studies in some subgroups and substantial heterogeneity, and were interpreted as exploratory. Complete results and interaction curves are provided in Supplementary Tables 8.8A, 8.8B and Supplementary Figures 8.8A, 8.8B.

### 3.9 Sensitivity analyses

In conventional Bayesian network meta-analysis, the principal findings remained stable across all sensitivity analyses. These analyses set the baseline-to-endpoint correlation to *R* = 0.50 or 0.90, excluded high-risk studies, or excluded large studies. For finger dexterity, the effects of AET and TOFDT versus UC remained statistically supported. For upper-limb function, the MBE effect versus UC persisted across all analyses, whereas the 95% CrIs for other comparisons continued to include 0 (Supplementary Figures 7.7A–7.7H).

In Bayesian dose–response network meta-analysis, overall curves for both outcomes retained positive trends across all sensitivity analyses. Effect directions and statistical support at prespecified dose nodes were consistent with the primary analyses. Modality-specific findings were generally robust, and whether 95% CrIs included 0 was unchanged at all comparable doses. Only CULR for finger dexterity and TAULHT for upper-limb function changed direction in some scenarios. Neither change received clear statistical support across the sensitivity scenarios.

Overall, sensitivity analyses did not alter the principal dose–response conclusions. However, estimated between-study heterogeneity for upper-limb-function models approached the prespecified upper bound of the prior, warranting cautious interpretation. Detailed results are provided in Supplementary Tables 8.9A–8.9E and Supplementary Figures 8.9A–8.9D.

### 3.10 Assessment of small-study effects and publication bias

Comparison-adjusted funnel plots showed no clear systematic asymmetry for either outcome. Egger regression found no clear small-study effects for finger dexterity (coefficient = −1.278, SE = 1.670, *p* = 0.450). No clear effect was detected for upper-limb function (coefficient = −0.313, SE = 5.142, *p* = 0.952). Because some direct comparisons contained few studies, power was limited and publication bias could not be excluded (Supplementary Table 7.8 and Supplementary Figures 7.8A, 7.8B).

## 4 Discussion

### 4.1 Principal findings

The conventional analyses indicated that exercise effects were outcome specific. High-certainty evidence supported improvements in finger dexterity with AET and TOFDT. In contrast, although MBE produced a statistically supported estimate for upper-limb function, this unusually large effect was based on only two small studies and low-certainty evidence and should therefore be regarded as exploratory.

Across the observed dose range, the modelled benefit increased approximately linearly for finger dexterity, whereas incremental gains in upper-limb function diminished with increasing dose. Statistically supported modality-specific curves were observed primarily for TOFDT and MBE. However, the predicted maxima occurred at the upper boundaries of the observed dose ranges and did not establish an optimal dose or support a specific clinical prescription.

### 4.2 Comparison with previous evidence

The findings of this study extend previous evidence that exercise benefits overall motor symptoms and manual dexterity in PD (8, 15, 31). The AET finding is consistent with current recommendations for aerobic exercise (11, 32) and with proposed effects on corticostriatal and frontoparietal connectivity, dopamine release and neuroplasticity (17, 33, 34). The TOFDT finding similarly accords with task-specific practice and previous hand-training trials (11, 35), potentially through motor-skill acquisition and experience-dependent neuroplasticity (17, 36). These mechanisms were not directly examined in the included trials.

The estimated upper-limb-function benefit of MBE extends previous evidence on overall motor function, balance, and quality of life (37–39). Re-examination confirmed that the extracted data matched the published reports and that repeated calculations reproduced the large estimate (Hedges’ *g* = 6.897, 95% CrI 4.28–9.43). However, this estimate was based on only two small trials involving 53 participants, which assessed handgrip strength but tested different practices (Tai Chi and Pilates); large between-group differences relative to the reported dispersion, possible small-study effects and substantial between-study heterogeneity across the broader upper-limb network limit confidence in its magnitude. Proposed mechanisms involving multijoint coordination and sensory feedback remain indirect. Given local inconsistency in the MBE-specific dose–response analysis, this finding should be regarded as exploratory.

The main advance of this study is the extension from modality comparisons to model-based dose–response relationships. Earlier reviews highlighted dose heterogeneity but could not identify effective components or appropriate doses for hand-focused exercise (8, 31). Existing dose studies have focused on overall motor symptoms or home exercise rather than finger dexterity or upper-limb function (25, 40). Different modeled dose–response shapes were identified, but the boundary maxima did not establish an optimal or recommended dose.

Findings remained stable across sensitivity analyses, although assessment type influenced finger-dexterity estimates. Larger effects with non-pegboard measures may reflect differences in task demands, constructs, and responsiveness rather than biological effect modification (7, 41). Interventions lasting at least 8 weeks showed a stronger dose-related association with upper-limb function. This direction accords with evidence from shorter home-exercise programs that did not improve overall motor symptoms (40). Nevertheless, these study-level analyses were exploratory and cannot define a duration threshold or support individualized prescribing.

### 4.3 Clinical and research implications

The two analytical layers provide complementary information for exercise planning. Conventional network meta-analysis informs modality selection, whereas model-based dose–response analysis provides population-level reference points for weekly dose. AET or TOFDT may be considered when impaired finger dexterity is the principal target. Evidence for MBE in broader upper-limb function remains preliminary and is currently insufficient to establish its superiority over other exercise modalities. Selection should also reflect functional goals, disease stage, patient preference, access, and safety.

Dose expressed as MET·min/week integrates exercise intensity, session duration, and weekly frequency. For aerobic programs, 450–700 MET·min/week may provide provisional escalation anchors from the overall finger-dexterity curve. These values are not AET-specific efficacy thresholds. TOFDT may begin near 180 MET·min/week and increase toward 450–700 MET·min/week as tolerated. Because the MBE dose–response curve was supported by only two small studies, the estimated range of 450–700 MET·min/week should be considered hypothesis-generating rather than a basis for clinical prescription.

Implementation should progressively adjust training time, frequency, or task difficulty while monitoring movement quality, adherence, fatigue, and adverse events. All proposed dose anchors derive from population-level models and should not be treated as individual thresholds. Because maximum predicted benefits occurred at observed-range boundaries, the present evidence cannot define an optimal or recommended dose.

Future trials should directly compare multiple doses within the same exercise modality and extend the studied dose range. Such designs are needed to determine whether benefits plateau or reverse. Trials should report planned and completed dose, intensity, frequency, session duration, progression, adherence, and adverse events. Standardized finger-dexterity and upper-limb-function outcomes would further strengthen evidence for individualized exercise prescription.

### 4.4 Strengths and limitations

This study integrates comparative exercise effects with model-based dose–response estimates. RoB 2 and CINeMA assessments, model comparisons, inconsistency checks, and sensitivity and subgroup analyses supported evaluation of evidence certainty and robustness.

Several limitations constrain interpretation. Five of 36 studies had high risk of bias and 10 raised some concerns, mainly regarding intervention deviations and missing data. Sparse direct comparisons and modality-specific dose data limited precision and stability. Differences in interventions, participants, and outcome measures contributed to heterogeneity. Standardization addressed scale differences but did not eliminate differences in constructs or responsiveness across strength-based, performance-based, and patient-reported measures.

The unusually large MBE estimate was supported by only two small trials involving 53 participants in the relevant comparison arms. Rechecking sample sizes, means, and dispersion measures confirmed agreement with the published reports, and repeated calculations reproduced the estimate. Nevertheless, sparse evidence and substantial upper-limb heterogeneity limit confidence in its magnitude; this finding remains exploratory.

Exercise doses were estimated from prescribed frequency, session duration, and assigned MET values, with limited information on adherence, progression, and individual exercise capacity. Estimated doses may therefore differ from completed exposure. Node mapping also removed within-node dose variation. Predicted maxima occurred at range boundaries and did not establish an optimal or recommended dose.

Differences in intervention duration may have influenced end-of-intervention effects through cumulative training exposure. Duration subgroup analyses showed no clear effect modification in conventional models, but longer interventions were associated with greater dose-related benefits for upper-limb function. These study-level analyses included sparse subgroups and cannot exclude residual confounding or establish causal duration effects. End-of-intervention assessments also cannot establish persistence of benefit. Larger trials with standardized outcomes, multiple exercise doses, and post-intervention follow-up are needed.

## 5 Conclusion

High-certainty evidence supported improvements in finger dexterity with AET and TOFDT. MBE produced an unusually large but low-certainty estimate for upper-limb function; because this estimate was based on only two small studies, it should be regarded as exploratory and does not establish either the true magnitude of effect or the superiority of MBE. Within observed dose ranges, modeled finger-dexterity benefit increased approximately linearly with overall exercise dose. Modeled upper-limb-function benefit also increased, although incremental gains diminished at higher doses. Because maximum predicted benefits occurred at observed-range boundaries, these findings do not imply that more exercise is always better. They also do not define an optimal dose. The results provide a framework for exercise planning and future dose-ranging trials, but current estimates cannot yet support a uniform clinical prescription.

## Data Availability

The original contributions presented in the study are included in the article/Supplementary material, further inquiries can be directed to the corresponding author.

## Glossary

AET: aerobic exercise training
CINeMA: Confidence in Network Meta-Analysis
CrI: credible interval
CULR: conventional upper-limb rehabilitation
DIC: deviance information criterion
ESS: effective sample size
MBE: mind–body exercise
MBNMA: model-based network meta-analysis
MCT: multicomponent training
MET: metabolic equivalent of task
NMA: network meta-analysis
PD: Parkinson’s disease
PRISMA: Preferred Reporting Items for Systematic Reviews and Meta-Analyses
RoB 2: Risk of Bias 2
RT: resistance training
SUCRA: surface under the cumulative ranking curve
TAULHT: technology-assisted upper-limb or hand training
TOFDT: task-oriented finger-dexterity training
UC: usual care or no structured exercise
UME: unrelated mean effects
