# Supplementary material for: Comparative effects and dose–response relationships of exercise for finger dexterity and upper-limb function in Parkinson’s disease: a Bayesian network and model-based dose–response meta-analysis
Source: Front Neurol. 2026 Oct 5;17:1964034. doi: 10.3389/fneur.2026.1964034 (PMC13681302; doi:10.3389/fneur.2026.1964034)
Supplement: Supplementary file 1 [file Data_Sheet_1.PDF]

Supplementary Materials for

Comparative Effects and Dose – Response Relationships of Exercise for Finger Dexterity and Upper-Limb Function in Parkinson's Disease: A Bayesian Network and Model-Based Dose – Response Meta-Analysis

Bu-Yu Liu[1†], Yu-Xin Zhuang[2†], Chang-Kuan Zhuang[1]*, Hui Ruan[1]

[1] School of Physical Education, Hainan Normal University, Haikou 571158, Hainan, China

[2] School of Acupuncture-Moxibustion and Tuina, Beijing University of Chinese Medicine, Beijing 100000, China

***Corresponding author:** Chang-Kuan Zhuang

Email: 090061@hainnu.edu.cn

Supplementary File 1: PRISMA NMA reporting checklist 4

Supplementary File 2: Literature Search Strategy 8

Supplementary File 3: Detailed eligibility criteria and theory-based definitions of intervention nodes 20

S3.1 Detailed eligibility criteria 20

S3.2 Theoretical basis and principles for intervention-node construction 21

S3.3 Operational definitions of intervention nodes 22

Supplementary File 4: Conversion of reported summary statistics and estimation of change-score standard deviations 23

S4.1 Conversion of reported summary statistics to means and standard deviations 23

S4.2 Estimation of change-score standard deviations 23

Supplementary File 5: Exercise dose estimation, MET assignment, dose node mapping, and arm-level data preparation for dose–response analyses 25

S5.1 Definition and calculation of weekly exercise dose 25

S5.2 Hierarchical assignment of MET values 25

S5.3 Handling of exercise and rehabilitation modalities 26

S5.4 Mapping exact weekly doses to prespecified dose nodes 26

S5.5 Traceability and interpretation 27

S5.6 Arm-level dose and outcome data for dose–response analyses 28

S5.7 Study-arm-level exercise prescriptions and MET derivations 32

Supplementary File 6: Risk-of-bias assessment 41

Supplementary File 7: Conventional Bayesian network meta-analysis 43

S7.1 Relative effects versus usual care and posterior intervention rankings 43

S7.2 CINeMA assessment of certainty of evidence for pairwise intervention comparisons 45

S7.3 Posterior between-study heterogeneity 48

S7.4 Global inconsistency, model-fit, and convergence assessment 49

S7.5 Local inconsistency assessed using node splitting 52

S7.6 Exploratory subgroup and treatment-by-subgroup interaction analyses 54

S7.7 Sensitivity analyses of relative treatment effects in the conventional Bayesian network meta-analysis 56

S7.8 Small-study effects and publication-bias diagnostics 61

Supplementary File 8: Bayesian dose–response network meta-analysis 63

S8.1 Observed dose distribution and data support 63
S8.2 Dose–response model selection, fit, convergence, and consistency 65
S8.3 Overall exercise dose–response associations 73
S8.4 Modality-specific dose–response associations 74
S8.5 Dose-specific treatment rankings 75
S8.6 CINeMA assessment of certainty of evidence in the Bayesian dose–response network meta-analysis 77
S8.7 Exploratory dose thresholds and data-supported effective ranges 79
S8.8 Exploratory dose-by-subgroup interaction analyses 81
S8.9 Sensitivity analyses of the dose–response network meta-analysis 83
References 91

Supplementary File 1: PRISMA NMA reporting checklist

PRISMA NMA Checklist of Items to Include When Reporting a Systematic Review Involving a Network Meta-analysis.

Section/Topic	Item	Checklist Item	Reported on Page
**Title**	**1**	**Comparative Effects and Dose–Response Relationships of Exercise for Finger Dexterity and Upper-Limb Function in Parkinson's Disease: A Bayesian Network and Model-Based Dose–Response Meta-Analysis**	**Main p. 1**
**ABSTRACT**			
**Structured summary**	**2**	**Structured abstract summarising the background, methods, results, conclusions and PROSPERO registration.**	**Main pp. 2 – 3**
**INTRODUCTION**			
**Rationale**	**3**	**Upper-limb impairment limits independence in Parkinson's disease; comparative exercise effects and dose – response relationships remain uncertain.**	**Main pp. 3 – 4**
**Objectives**	**4**	**To compare exercise modalities for finger dexterity and upper-limb function and model overall and modality-specific dose – response relationships.**	**Main p. 4**
**METHODS**			
**Protocol and registration**	**5**	**Prospectively registered in PROSPERO (CRD420261463560) and reported according to PRISMA 2020 and the PRISMA extension for network meta-analyses.**	**Main p. 5**
**Eligibility criteria**	**6**	**RCTs in adults with clinically diagnosed Parkinson's disease evaluating eligible exercise or rehabilitation interventions and reporting finger-dexterity or upper-limb-function outcomes.**	**Main pp. 5–6; Supp. pp. 20–23**
**Information sources**	**7**	**Six databases were searched from inception to 20 June 2026; reference lists, ClinicalTrials.gov and the WHO ICTRP were also searched.**	**Main p. 5**

Section/Topic	Item	Checklist Item	Reported on Page
**Search**	**8**	**Searches combined Parkinson' s disease, exercise or rehabilitation, and RCT terms; full strategies and yields are reported in Supplementary File 2.**	**Main pp. 5; Supp. pp. 8 – 19**
**Study selection**	**9**	**Two reviewers independently screened records and full texts; disagreements were resolved by discussion or third-reviewer adjudication.**	**Main p. 6**
**Data collection process**	**10**	**Two reviewers independently extracted data using a standardised form, with third-reviewer adjudication when required.**	**Main p. 6**
**Data items**	**11**	**Extracted items included study and participant characteristics, intervention prescription and dose, outcomes, and change-score statistics.**	**Main pp. 6–8; Supp. pp. 24–41**
**Geometry of the network**	**12**	**Separate networks were constructed for finger dexterity and upper-limb function using prespecified exercise-modality and dose nodes.**	**Main pp. 7–10, 13; Fig. 2 (p. 39)**
**Risk of bias within individual studies**	**13**	**Two reviewers independently assessed outcome-specific risk of bias using the Cochrane Risk of Bias 2 tool.**	**Main p. 8; Supp. pp. 42–43**
**Summary measures**	**14**	**Effects were expressed as Hedges' g with 95% credible intervals; rankings used SUCRA, mean rank and rank probabilities.**	**Main pp. 7 and 9–12; Figs. 3–7 (pp. 40–44); Supp. Files 7–8 (pp. 44–91)**
**Planned methods of analysis**	**15**	**Bayesian random-effects network and model-based dose – response meta-analyses were fitted, accounting jointly for multi-arm trials and comparing candidate dose functions.**	**Main pp. 9–12; Supp. File 7 (pp. 44–61) and File 8 (pp. 64–91)**
**Assessment of Inconsistency**	**16**	**Global inconsistency was assessed by comparing consistency and unrelated mean effects models; local inconsistency was assessed by node splitting.**	**Main pp. 9–10, 15–16 and 18–19; Supp. S7.4–S7.5 (pp. 50–54) and S8.2 (pp. 66–73)**
**Risk of bias across studies**	**17**	**Comparison-adjusted funnel plots and Egger regression explored small-study effects in**	**Main pp. 9 and 22; Supp. S7.8 (pp.**

Section/Topic	Item	Checklist Item	Reported on Page
		networks with at least 10 studies.	62–63)
Additional analyses	18	Prespecified subgroup and sensitivity analyses examined intervention length, age, assessment type, assumed correlation, risk of bias and large studies.	Main pp. 9–12 and 20–22; Supp. S7.6–S7.7 (pp. 55–61) and S8.8–S8.9 (pp. 82–91)
RESULTS†			
Study selection	19	The searches identified 12,147 records; 186 full texts were assessed and 36 reports were included (Fig. 1).	Main pp. 12–13; Fig. 1 (p. 38)
Presentation of network structure	20	Network plots for finger dexterity and upper-limb function are presented in Fig. 2.	Main p. 39 (Fig. 2)
Summary of network geometry	21	Both connected networks comprised seven active interventions plus usual care, with 12 and 10 direct comparisons, respectively.	Main p. 13; Fig. 2 (p. 39)
Study characteristics	22	The 36 reports included 1,575 participants; study, intervention, outcome and dose characteristics are summarised in Table 1 and Supplementary File 5.	Main pp. 13–14; Table 1 (pp. 35–37); Supp. pp. 29–33
Risk of bias within studies	23	Risk of Bias 2 classified 21 reports as low risk, 10 as having some concerns and 5 as high risk.	Main p. 14; Supp. pp. 42–43
Results of individual studies	24	Arm-level study characteristics, exercise prescriptions, outcome data and derived doses are reported in Supplementary File 5.	Supp. pp. 29–33
Synthesis of results	25	Relative effects, rankings, dose – response estimates and CINeMA certainty assessments are reported for both outcomes.	Main pp. 14–20; Figs. 3–7 (pp. 40–44); Supp. Files 7–8 (pp. 44–91)
Exploration for inconsistency	26	Conventional networks showed no clear global or local inconsistency; local inconsistency was identified for selected modality-specific dose comparisons.	Main pp. 15–16 and 18–19; Supp. S7.4–S7.5 (pp. 50–54) and S8.2 (pp. 66–73)
Risk of bias across studies	27	Funnel plots and Egger regression showed no clear small-study effects, although power was	Main p. 22; Supp. S7.8 (pp. 62–63)

Section/Topic	Item	Checklist Item	Reported on Page
		limited.	
Results of additional analyses	28	Subgroup and sensitivity analyses were exploratory; the principal findings were generally robust.	Main pp. 20–22; Supp. S7.6–S7.7 (pp. 55–61) and S8.8–S8.9 (pp. 82–91)
DISCUSSION			
Summary of evidence	29	Aerobic exercise and task-oriented finger-dexterity training improved finger dexterity; mind – body exercise may improve upper-limb function. Dose – response patterns differed by outcome.	Main p. 22
Limitations	30	Limitations included sparse comparisons, few studies for some dose curves, heterogeneity, estimated rather than completed dose, and boundary-limited dose ranges.	Main p. 25
Conclusions	31	Exercise effects were outcome specific; current evidence supports modality selection and dose-ranging research but not an optimal dose or uniform prescription.	Main p. 26
FUNDING			
Funding	32	Funded by the Sports Culture Research Base of the National Sports Administration Bureau at Hainan Normal University and the Hainan Province Philosophy and Social Science Planning Project (HNSK(ZC) 25-224).	Main p. 27

Supplementary File 2: Literature Search Strategy

Information		
Search date: June 20, 2026<br>*Databases searched*: PubMed/MEDLINE、Embase、Cochrane Central Register of Controlled Trials （CENTRAL） 、Web of Science、Scopus、SPORTDiscus		
#	Searches	Results
PubMed		
#1	"Parkinson Disease"[Mesh]	95,468
#2	(((((((((Idiopathic Parkinson Disease[tiab]) OR (Idiopathic Parkinson's Disease[tiab])) OR (Lewy Body Parkinson Disease[tiab])) OR (Lewy Body Parkinson's Disease[tiab])) OR (Paralysis Agitans[tiab])) OR (Parkinson Disease, Idiopathic[tiab])) OR (Parkinson's Disease[tiab])) OR (Parkinson's Disease, Idiopathic[tiab])) OR (Parkinson's Disease, Lewy Body[tiab])) OR (Primary Parkinsonism[tiab])) OR (Parkinsonism, Primary[tiab])	134,879
#3	#1 OR #2	155,282
#4	"Exercise"[Mesh]	289,219
#5	((((((((((((((((((((((((Exercises[tiab]) OR (Exercise, Physical[tiab])) OR (Exercises, Physical[tiab])) OR (Physical Exercise[tiab])) OR (Physical Exercises[tiab])) OR (Exercise, Isometric[tiab])) OR (Exercises, Isometric[tiab])) OR (Isometric Exercises[tiab])) OR (Isometric Exercise[tiab])) OR (Exercise, Aerobic[tiab])) OR (Aerobic Exercise[tiab])) OR (Aerobic Exercises[tiab])) OR (Exercises, Aerobic[tiab])) OR (Exercise Training[tiab])) OR (Exercise Trainings[tiab])) OR (Training, Exercise[tiab])) OR (Trainings, Exercise[tiab])) OR (Physical Activity[tiab])) OR (Activities, Physical[tiab])) OR (Activity, Physical[tiab])) OR (Physical Activities[tiab])) OR (Active Breaks[tiab])) OR (Activity Breaks[tiab])) OR (Acute Exercise[tiab])) OR (Acute Exercises[tiab])) OR (Exercise, Acute[tiab])) OR (Exercises, Acute[tiab])	374,402
#6	#4 OR #5	519,753
#7	"Mind-Body Therapies"[Mesh]	52,299
#8	(((((Mind Body Therapies[tiab]) OR (Mind-Body Therapy[tiab])) OR (Therapies, Mind-Body[tiab])) OR (Therapy, Mind-Body[tiab])) OR (Mind-Body Medicine[tiab])) OR (Mind Body Medicine[tiab])	1,171
#9	#7 OR #8	52,952
#10	"Dancing"[Mesh]	4,088
#11	(((((((((((((((Dance[tiab]) OR (Ballet[tiab])) OR (Jazz Dance[tiab])) OR (Dance, Jazz[tiab])) OR (Tap Dance[tiab])) OR (Dance, Tap[tiab])) OR (Modern Dance[tiab])) OR (Dance, Modern[tiab])) OR (Hip-Hop Dance[tiab])) OR (Dance, Hip-Hop[tiab])) OR (Hip Hop Dance[tiab])) OR (Line Dancing[tiab])) OR (Dancing, Line[tiab])) OR	8,644

	(Salsa Dancing[tiab])) OR (Dancing, Salsa[tiab])) OR (Square Dance[tiab])) OR (Dance, Square[tiab])	
#12	#10 OR #11	9,645
#13	"Occupational Therapy"[Mesh]	16,547
#14	((((Occupational Therapies[tiab]) OR (Therapies, Occupational[tiab])) OR (Ergotherapy[tiab])) OR (Ergotherapies[tiab])) OR (Therapy, Occupational[tiab])	824
#15	#13 OR #14	17,185
#16	"Physical Therapy Modalities"[Mesh]	203,843
#17	((((((((((((((((Modalities, Physical Therapy[tiab]) OR (Modality, Physical Therapy[tiab])) OR (Physical Therapy Modality[tiab])) OR (Physical Therapy Techniques[tiab])) OR (Physical Therapy Technique[tiab])) OR (Techniques, Physical Therapy[tiab])) OR (Physiotherapy (Techniques)[tiab])) OR (Physiotherapies (Techniques)[tiab])) OR (Neurological Physiotherapy[tiab])) OR (Physiotherapy, Neurological[tiab])) OR (Neurophysiotherapy[tiab])) OR (Group Physiotherapy[tiab])) OR (Group Physiotherapies[tiab])) OR (Physiotherapies, Group[tiab])) OR (Physiotherapy, Group[tiab])) OR (Physical Therapy[tiab])) OR (Physical Therapies[tiab])) OR (Therapy, Physical[tiab])	209,969
#18	#16 OR #17	272,332
#19	"Virtual Reality"[Mesh]	10,937
#20	((((((((((Reality, Virtual[tiab]) OR (Virtual Reality, Educational[tiab])) OR (Educational Virtual Realities[tiab])) OR (Educational Virtual Reality[tiab])) OR (Reality, Educational Virtual[tiab])) OR (Virtual Realities, Educational[tiab])) OR (Virtual Reality, Instructional[tiab])) OR (Instructional Virtual Realities[tiab])) OR (Instructional Virtual Reality[tiab])) OR (Realities, Instructional Virtual[tiab])) OR (Reality, Instructional Virtual[tiab])) OR (Virtual Realities, Instructional[tiab])	5,478
#21	#19 OR #20	14,386
#22	"Video Games"[Mesh]	9,338
#23	((((((((Games, Video[tiab]) OR (Game, Video[tiab])) OR (Video Game[tiab])) OR (Videogame[tiab])) OR (Videogames[tiab])) OR (Computer Games[tiab])) OR (Computer Game[tiab])) OR (Game, Computer[tiab])) OR (Games, Computer[tiab])	6,194
#24	#22 OR #23	12,598
#25	"Robotics"[Mesh]	54,935
#26	((((((((((((((((((Remote Operations (Robotics)[tiab]) OR (Operation, Remote (Robotics)[tiab])) OR (Operations, Remote (Robotics)[tiab])) OR (Remote Operation (Robotics)[tiab])) OR (Telerobotics[tiab])) OR (Soft Robotics[tiab])) OR (Robotic, Soft[tiab])) OR (Soft Robotic[tiab])) OR (Socially Assistive Robots[tiab])) OR (Assistive Robot, Socially[tiab])) OR (Robot, Socially Assistive[tiab])) OR (Socially Assistive Robot[tiab])) OR (Social Robots[tiab])) OR (Robot, Social[tiab])) OR (Social Robot[tiab])) OR (Humanoid Robots[tiab])) OR (Humanoid Robot[tiab])) OR (Robot, Humanoid[tiab])) OR (Companion Robots[tiab])) OR (Companion Robot[tiab])) OR	8,401

	(Robot, Companion[tiab])	
#27	#25 OR #26	60,283
#28	"Therapy, Computer-Assisted"[Mesh]	51,114
#29	((((((((((((Therapy, Computer Assisted[tiab]) OR (Computer-Assisted Protocol-Directed Therapy[tiab])) OR (Computer-Assisted Protocol-Directed Therapies[tiab])) OR (Computer Assisted Protocol Directed Therapy[tiab])) OR (Protocol-Directed Therapies, Computer-Assisted[tiab])) OR (Therapies, Computer-Assisted Protocol-Directed[tiab])) OR (Therapy, Computer-Assisted Protocol-Directed[tiab])) OR (Therapy, Computer Assisted Protocol Directed[tiab])) OR (Protocol-Directed Therapy, Computer-Assisted[tiab])) OR (Protocol Directed Therapy, Computer Assisted[tiab])) OR (Computer-Assisted Therapy[tiab])) OR (Computer-Assisted Therapies[tiab])) OR (Computer Assisted Therapy[tiab])) OR (Therapies, Computer-Assisted[tiab])	11,195
#30	#28 OR #29	61,445
#31	#6 OR #9 OR #12 OR #15 OR #18 OR #21 OR #24 OR #27 OR #30	908,037
#32	((((((randomized controlled trial[pt]) OR (controlled clinical trial[pt])) OR (randomized[tiab])) OR (randomised[tiab])) OR (randomly[tiab])) OR (trial[tiab])) OR (placebo[tiab])	1,794,203
#33	#3 AND #31 AND #32	1,666

Cochrane		
#1	MeSH descriptor: [Parkinson Disease] explode all trees	6,685
#2	(‘Parkinson Disease, Idiopathic’ OR ‘Idiopathic Parkinson Disease’ OR ‘Parkinsonism, Primary’ OR ‘Idiopathic Parkinson's Disease’ OR ‘Primary Parkinsonism’ OR ‘Parkinson's Disease, Lewy Body’ OR ‘Parkinson's Disease’ OR ‘Lewy Body Parkinson's Disease’ OR ‘Paralysis Agitans’ OR ‘Parkinson's Disease, Idiopathic’ OR ‘Lewy Body Parkinson Disease’):ti,ab,kw	15,328
#3	#1 OR #2	15,328
#4	MeSH descriptor: [Exercise] explode all trees	43,817
#5	(‘Exercises’ OR ‘Exercise, Physical’ OR ‘Physical Exercises’ OR ‘Physical Exercise’ OR ‘Exercises, Physical’ OR ‘Physical Activity’ OR ‘Physical Activities’ OR ‘Activity, Physical’ OR ‘Activities, Physical’ OR ‘Trainings, Exercise’ OR ‘Training, Exercise’ OR ‘Exercise Training’ OR ‘Exercise Trainings’ OR ‘Exercises, Isometric’ OR ‘Exercise, Isometric’ OR ‘Isometric Exercises’ OR ‘Isometric Exercise’ OR ‘Aerobic Exercises’ OR ‘Aerobic Exercise’ OR ‘Exercise, Aerobic’ OR ‘Exercises, Aerobic’ OR ‘Acute Exercises’ OR ‘Acute Exercise’ OR ‘Exercises, Acute’ OR ‘Exercise, Acute’ OR ‘Activity Breaks’ OR ‘Active Breaks’):ti,ab,kw	173,657
#6	#4 OR #5	184,461
#7	MeSH descriptor: [Mind-Body Therapies] explode all trees	10,792

Cochrane		
#8	('Mind Body Therapies' OR 'Mind-Body Therapy' OR 'Therapy, Mind-Body' OR 'Therapies, Mind-Body' OR 'Mind-Body Medicine' OR 'Mind Body Medicine'):ti,ab,kw	1,471
#9	#7 OR #8	11,752
#10	MeSH descriptor: [Dancing] explode all trees	344
#11	('Salsa Dancing' OR 'Dancing, Salsa' OR 'Line Dancing' OR 'Dancing, Line' OR 'Hip-Hop Dance' OR 'Dance, Hip-Hop' OR 'Hip Hop Dance' OR 'Ballet' OR 'Dance' OR 'Square Dance' OR 'Dance, Square' OR 'Dance, Modern' OR 'Modern Dance' OR 'Jazz Dance' OR 'Dance, Jazz' OR 'Dance, Tap' OR 'Tap Dance'):ti,ab,kw	1,756
#12	#10 OR #11	1,798
#13	MeSH descriptor: [Occupational Therapy] explode all trees	1,144
#14	('Therapy, Occupational' OR 'Ergotherapies' OR 'Therapies, Occupational' OR 'Ergotherapy' OR 'Occupational Therapies'):ti,ab,kw	8,039
#15	#13 OR #14	8,039
#16	MeSH descriptor: [Physical Therapy Modalities] explode all trees	45,458
#17	('Neurophysiotherapy' OR 'Neurological Physiotherapy' OR 'Physiotherapy, Neurological' OR 'Therapy, Physical' OR 'Physical Therapies' OR 'Physical Therapy' OR 'Group Physiotherapy' OR 'Physiotherapy, Group' OR 'Physiotherapies, Group' OR 'Group Physiotherapies' OR 'Techniques, Physical Therapy' OR 'Physiotherapy (Techniques)' OR 'Physical Therapy Technique' OR 'Modality, Physical Therapy' OR 'Modalities, Physical Therapy' OR 'Physiotherapies (Techniques)' OR 'Physical Therapy Techniques' OR 'Physical Therapy Modality'):ti,ab,kw	91,960
#18	#16 OR #17	120,427
#19	MeSH descriptor: [Virtual Reality] explode all trees	1,916
#20	('Reality, Educational Virtual' OR 'Instructional Virtual Reality' OR 'Reality, Instructional Virtual' OR 'Virtual Realities, Instructional' OR 'Educational Virtual Reality' OR 'Instructional Virtual Realities' OR 'Virtual Reality, Educational' OR 'Educational Virtual Realities' OR 'Virtual Reality, Instructional' OR 'Realities, Instructional Virtual' OR 'Virtual Realities, Educational' OR 'Reality, Virtual'):ti,ab,kw	10,551
#21	#19 OR #20	10,576
#22	MeSH descriptor: [Video Games] explode all trees	1,527
#23	('Games, Video' OR 'Video Game' OR 'Videogames' OR 'Videogame' OR 'Game, Video' OR 'Games, Computer' OR 'Computer Games' OR 'Game, Computer' OR 'Computer Game'):ti,ab,kw	4,766
#24	#22 OR #23	4,835
#25	MeSH descriptor: [Robotics] explode all trees	2,360
#26	('Socially Assistive Robot' OR 'Robot, Social' OR 'Socially Assistive Robots' OR	590

Cochrane		
	'Social Robot' OR 'Social Robots' OR 'Robot, Socially Assistive' OR 'Assistive Robot, Socially' OR 'Remote Operations (Robotics)' OR 'Operations, Remote (Robotics)' OR 'Remote Operation (Robotics)' OR 'Operation, Remote (Robotics)' OR 'Soft Robotic' OR 'Soft Robotics' OR 'Robotic, Soft' OR 'Robot, Companion' OR 'Companion Robots' OR 'Companion Robot' OR 'Humanoid Robot' OR 'Humanoid Robots' OR 'Robot, Humanoid' OR 'Telerobotics'):ti,ab,kw	
#27	#25 OR #26	2,794
#28	MeSH descriptor: [Therapy, Computer-Assisted] explode all trees	3,353
#29	('Protocol-Directed Therapies, Computer-Assisted' OR 'Therapy, Computer-Assisted Protocol-Directed' OR 'Computer-Assisted Therapy' OR 'Therapies, Computer-Assisted' OR 'Therapy, Computer Assisted Protocol Directed' OR 'Computer-Assisted Protocol-Directed Therapies' OR 'Protocol-Directed Therapy, Computer-Assisted' OR 'Computer Assisted Protocol Directed Therapy' OR 'Computer-Assisted Protocol-Directed Therapy' OR 'Therapy, Computer Assisted' OR 'Computer-Assisted Therapies' OR 'Protocol Directed Therapy, Computer Assisted' OR 'Computer Assisted Therapy' OR 'Therapies, Computer-Assisted Protocol-Directed'):ti,ab,kw	10,539
#30	#28 OR #29	11,653
#31	#6 OR #9 OR #12 OR #15 OR #18 OR #21 OR #24 OR #27 OR #30	214,374
#32	('randomized controlled trial' OR 'randomized' OR 'placebo'):ti,ab,kw	1,461,057
#33	#3 AND #31 AND #32	1,880

Embase		
#1	'parkinson disease'/exp	245,866
#2	'idiopathic parkinsonism':ti,ab,kw OR 'lewy bodies of parkinson disease':ti,ab,kw OR 'lewy bodies of parkinson`s disease':ti,ab,kw OR 'lewy bodies of parkinsons disease':ti,ab,kw OR 'lewy body parkinson disease':ti,ab,kw OR 'lewy body parkinson`s disease':ti,ab,kw OR 'lewy body parkinsons disease':ti,ab,kw OR 'paralysis agitans':ti,ab,kw OR 'parkinson dementia complex':ti,ab,kw OR 'parkinson`s disease':ti,ab,kw OR 'parkinsons disease':ti,ab,kw OR 'primary parkinsonism':ti,ab,kw OR 'parkinson disease':ti,ab,kw	220,626
#3	#1 OR #2	277,075
#4	'exercise'/exp	589,783
#5	'biometric exercise':ti,ab,kw OR 'effort':ti,ab,kw OR 'exercise capacity':ti,ab,kw OR 'exercise performance':ti,ab,kw OR 'exercise training':ti,ab,kw OR 'exertion':ti,ab,kw OR 'fitness training':ti,ab,kw OR 'fitness workout':ti,ab,kw OR 'physical conditioning, human':ti,ab,kw OR 'physical effort':ti,ab,kw OR 'physical exercise':ti,ab,kw OR	812,315

Embase		
	'physical exertion':ti,ab,kw OR 'physical work-out':ti,ab,kw OR 'physical workout':ti,ab,kw OR 'exercise':ti,ab,kw	
#6	#4 OR #5	1,013,526
#7	'mind body therapy'/exp	95,714
#8	'mind-body therapy':ti,ab,kw OR 'mind body therapies':ti,ab,kw OR 'mind-body therapies':ti,ab,kw OR 'mind body medicine':ti,ab,kw OR 'mind-body medicine':ti,ab,kw OR 'tai chi':ti,ab,kw OR 'taiji':ti,ab,kw OR 'qigong':ti,ab,kw OR 'yoga':ti,ab,kw	21,020
#9	#7 OR #8	112,914
#10	'dancing'/exp	9,077
#11	'dance':ti,ab,kw OR 'dancer':ti,ab,kw OR 'dancing':ti,ab,kw	13,543
#12	#10 OR #11	15,477
#13	'occupational therapy'/exp	35,143
#14	'ergotherapy':ti,ab,kw OR 'occupation therapy':ti,ab,kw OR 'paediatric occupational therapy':ti,ab,kw OR 'pediatric occupational therapy':ti,ab,kw OR 'therapy, occupational':ti,ab,kw OR 'occupational therapy':ti,ab,kw	25,690
#15	#13 OR #14	41,314
#16	'physiotherapy'/exp	151,606
#17	'physical therapy':ti,ab,kw OR 'physical therapy (speciality)':ti,ab,kw OR 'physical therapy (specialty)':ti,ab,kw OR 'physical therapy modalities':ti,ab,kw OR 'physical therapy service':ti,ab,kw OR 'physical therapy speciality':ti,ab,kw OR 'physical therapy specialty':ti,ab,kw OR 'physical therapy techniques':ti,ab,kw OR 'physical treatment':ti,ab,kw OR 'physio therapy':ti,ab,kw OR 'physiotherapy department':ti,ab,kw OR 'therapy, physical':ti,ab,kw OR 'physiotherapy':ti,ab,kw	115,103
#18	#16 OR #17	184,494
#19	'virtual reality'/exp	43,122
#20	'virtual reality':ti,ab,kw	37,168
#21	#19 OR #20	50,776
#22	'video game'/exp	10,474
#23	'computer game':ti,ab,kw OR 'computergame':ti,ab,kw OR 'television game':ti,ab,kw OR 'tv games':ti,ab,kw OR 'video games':ti,ab,kw OR 'videogame':ti,ab,kw OR 'videogames':ti,ab,kw OR 'video game':ti,ab,kw	10,339
#24	#22 OR #23	15,121
#25	'robotics'/exp	54,331
#26	'robotics':ti,ab,kw	23,257
#27	#25 OR #26	63,198
#28	'computer assisted therapy'/exp	15,475
#29	'computer-assisted therapy':ti,ab,kw OR 'therapy, computer-assisted':ti,ab,kw OR 'computer assisted therapy':ti,ab,kw	229

Embase		
#30	#28 OR #29	15,595
#31	#6 OR #9 OR #12 OR #15 OR #18 OR #21 OR #24 OR #27 OR #30	1,414,342
#32	(‘randomized controlled trial’ OR ‘randomized’ OR ‘placebo’):ti,ab,kw	1,487,362
#33	#3 AND #31 AND #32	2,082

Web of Science		
#1	TS=(‘Parkinson Disease’ OR ‘Parkinson Disease, Idiopathic’ OR ‘Idiopathic Parkinson Disease’ OR ‘Parkinsonism, Primary’ OR ‘Idiopathic Parkinson's Disease’ OR ‘Primary Parkinsonism’ OR ‘Parkinson's Disease, Lewy Body’ OR ‘Parkinson's Disease’ OR ‘Lewy Body Parkinson's Disease’ OR ‘Paralysis Agitans’ OR ‘Parkinson's Disease, Idiopathic’ OR ‘Lewy Body Parkinson Disease’)	227,246
#2	TS=(‘Exercises’ OR ‘Exercise, Physical’ OR ‘Physical Exercises’ OR ‘Physical Exercise’ OR ‘Exercises, Physical’ OR ‘Physical Activity’ OR ‘Physical Activities’ OR ‘Activity, Physical’ OR ‘Activities, Physical’ OR ‘Trainings, Exercise’ OR ‘Training, Exercise’ OR ‘Exercise Training’ OR ‘Exercise Trainings’ OR ‘Exercises, Isometric’ OR ‘Exercise, Isometric’ OR ‘Isometric Exercises’ OR ‘Isometric Exercise’ OR ‘Aerobic Exercises’ OR ‘Aerobic Exercise’ OR ‘Exercise, Aerobic’ OR ‘Exercises, Aerobic’ OR ‘Acute Exercises’ OR ‘Acute Exercise’ OR ‘Exercises, Acute’ OR ‘Exercise, Acute’ OR ‘Activity Breaks’ OR ‘Active Breaks’)	1,987,523
#3	TS=(‘Mind Body Therapies’ OR ‘Mind-Body Therapy’ OR ‘Therapy, Mind-Body’ OR ‘Therapies, Mind-Body’ OR ‘Mind-Body Medicine’ OR ‘Mind Body Medicine’)	14,777
#4	TS=(‘Dancing’ OR ‘Salsa Dancing’ OR ‘Dancing, Salsa’ OR ‘Line Dancing’ OR ‘Dancing, Line’ OR ‘Hip-Hop Dance’ OR ‘Dance, Hip-Hop’ OR ‘Hip Hop Dance’ OR ‘Ballet’ OR ‘Dance’ OR ‘Square Dance’ OR ‘Dance, Square’ OR ‘Dance, Modern’ OR ‘Modern Dance’ OR ‘Jazz Dance’ OR ‘Dance, Jazz’ OR ‘Dance, Tap’ OR ‘Tap Dance’)	81,444
#5	TS=(‘Occupational Therapy’ OR ‘Therapy, Occupational’ OR ‘Ergotherapies’ OR ‘Therapies, Occupational’ OR ‘Ergotherapy’ OR ‘Occupational Therapies’)	113,906
#6	TS=(‘Physical Therapy Modalities’ OR ‘Neurophysiotherapy’ OR ‘Neurological Physiotherapy’ OR ‘Physiotherapy, Neurological’ OR ‘Therapy, Physical’ OR ‘Physical Therapies’ OR ‘Physical Therapy’ OR ‘Group Physiotherapy’ OR ‘Physiotherapy, Group’ OR ‘Physiotherapies, Group’ OR ‘Group Physiotherapies’ OR ‘Techniques, Physical Therapy’ OR ‘Physiotherapy (Techniques)’ OR ‘Physical Therapy Technique’ OR ‘Modality, Physical Therapy’ OR ‘Modalities, Physical Therapy’ OR ‘Physiotherapies (Techniques)’ OR ‘Physical Therapy Techniques’ OR ‘Physical Therapy Modality’)	753,814
#7	TS=(‘Virtual Reality’ OR ‘Reality, Educational Virtual’ OR ‘Instructional Virtual	120,243

Web of Science		
	Reality' OR 'Reality, Instructional Virtual' OR 'Virtual Realities, Instructional' OR 'Educational Virtual Reality' OR 'Instructional Virtual Realities' OR 'Virtual Reality, Educational' OR 'Educational Virtual Realities' OR 'Virtual Reality, Instructional' OR 'Realities, Instructional Virtual' OR 'Virtual Realities, Educational' OR 'Reality, Virtual')	
#8	TS=('Video Games' OR 'Games, Video' OR 'Video Game' OR 'Videogames' OR 'Videogame' OR 'Game, Video' OR 'Games, Computer' OR 'Computer Games' OR 'Game, Computer' OR 'Computer Game')	95,951
#9	TS=('Robotics' OR 'Socially Assistive Robot' OR 'Robot, Social' OR 'Socially Assistive Robots' OR 'Social Robot' OR 'Social Robots' OR 'Robot, Socially Assistive' OR 'Assistive Robot, Socially' OR 'Remote Operations (Robotics)' OR 'Operations, Remote (Robotics)' OR 'Remote Operation (Robotics)' OR 'Operation, Remote (Robotics)' OR 'Soft Robotic' OR 'Soft Robotics' OR 'Robotic, Soft' OR 'Robot, Companion' OR 'Companion Robots' OR 'Companion Robot' OR 'Humanoid Robot' OR 'Humanoid Robots' OR 'Robot, Humanoid' OR 'Telerobotics')	282,443
#10	TS=('Therapy, Computer-Assisted' OR 'Protocol-Directed Therapies, Computer-Assisted' OR 'Therapy, Computer-Assisted Protocol-Directed' OR 'Computer-Assisted Therapy' OR 'Therapies, Computer-Assisted' OR 'Therapy, Computer Assisted Protocol Directed' OR 'Computer-Assisted Protocol-Directed Therapies' OR 'Protocol-Directed Therapy, Computer-Assisted' OR 'Computer Assisted Protocol Directed Therapy' OR 'Computer-Assisted Protocol-Directed Therapy' OR 'Therapy, Computer Assisted' OR 'Computer-Assisted Therapies' OR 'Protocol Directed Therapy, Computer Assisted' OR 'Computer Assisted Therapy' OR 'Therapies, Computer-Assisted Protocol-Directed')	113,158
#11	#2 OR #3 OR #4 OR #5 OR #6 OR #7 OR #8 OR #9 OR #10	3,079,994
#12	TS=("randomized controlled trial")	274,648
#13	#1 AND #11 AND #12	859

Scopus		
#1	TITLE-ABS-KEY (Parkinson* OR parkinsonism OR "Parkinson disease" OR "Parkinson's disease" OR "idiopathic Parkinson disease" OR "idiopathic Parkinson's disease" OR "Lewy Body Parkinson disease" OR "Lewy Body Parkinson's disease" OR "primary parkinsonism" OR "paralysis agitans")	270,930
#2	TITLE-ABS-KEY (exercise OR exercises OR exercising OR "physical exercise" OR "physical exercises" OR "exercise training" OR "physical activity" OR "physical activities" OR "aerobic exercise" OR "aerobic exercises" OR "isometric exercise" OR "isometric exercises" OR "acute	3,239,614

Scopus		
	exercise" OR "acute exercises" OR "active break" OR "active breaks" OR training)	
#3	TITLE-ABS-KEY ("mind-body therapy" OR "mind body therapy" OR "mind-body therapies" OR "mind body therapies" OR "mind-body medicine" OR "mind body medicine" OR "tai chi" OR taiji OR qigong OR yoga)	28,467
#4	TITLE-ABS-KEY (dance OR dancing OR ballet OR "jazz dance" OR "tap dance" OR "modern dance" OR "hip-hop dance" OR "hip hop dance" OR "line dancing" OR "salsa dancing" OR "square dance" OR tango)	67,019
#5	TITLE-ABS-KEY ("occupational therapy" OR "occupational therapies" OR ergotherapy OR ergotherapies)	48,492
#6	TITLE-ABS-KEY (physiotherapy OR physiotherapies OR "physical therapy" OR "physical therapies" OR "physical therapy modality" OR "physical therapy modalities" OR "physical therapy technique" OR "physical therapy techniques" OR "neurological physiotherapy" OR neurophysiotherapy OR "group physiotherapy")	168,933
#7	TITLE-ABS-KEY ("virtual reality" OR "virtual realities" OR "educational virtual reality" OR "educational virtual realities" OR "instructional virtual reality" OR "instructional virtual realities" OR VR)	257,115
#8	TITLE-ABS-KEY ("video game" OR "video games" OR videogame OR videogames OR "computer game" OR "computer games" OR exergame OR exergames OR Wii OR Kinect)	81,763
#9	TITLE-ABS-KEY (robotics OR robot OR robots OR robotic OR "robot-assisted" OR " robot assisted" OR telerobotics OR "soft robotics" OR "soft robotic" OR "socially assistive robot" OR "socially assistive robots" OR "social robot" OR "social robots" OR "humanoid robot" OR "humanoid robots" OR "companion robot" OR "companion robots")	837,119
#10	TITLE-ABS-KEY ("computer-assisted therapy" OR "computer assisted therapy" OR "computer-assisted therapies" OR "computer assisted therapies" OR "computer-assisted" OR "computer assisted" OR "technology-assisted" OR "technology assisted" OR "computer-assisted protocol-directed therapy" OR "computer assisted protocol directed therapy" OR telerehabilitation OR "remote rehabilitation")	1,321,327
#11	#2 OR #3 OR #4 OR #5 OR #6 OR #7 OR #8 OR #9 OR #10	5,719,780
#12	TITLE-ABS-KEY (random OR randomized OR randomised OR randomly OR trial O R trials OR "randomized controlled trial" OR "randomised controlled trial" OR "controlled clinical trial" OR "clinical trial")	5,787,152
#13	#1 AND #11 AND #12	5,302

SPORTDiscus		
#1	(TI (Parkinson* OR parkinsonism OR "paralysis agitans") OR AB (Parkinson* OR parkinsonism OR "paralysis agitans") OR SU (Parkinson* OR parkinsonism OR "paralysis agitans"))	4,363
#2	(TI (exercise* OR "physical exercise*" OR "physical activit*" OR "exercise training" OR "aerobic exercise*" OR "isometric exercise*" OR "resistance training" OR "strength training" OR "motor training" OR "task-oriented training" OR "task oriented training" OR rehabilitation) OR AB (exercise* OR "physical exercise*" OR "physical activit*" OR "exercise training" OR "aerobic exercise*" OR "isometric exercise*" OR "resistance training" OR "strength training" OR "motor training" OR "task-oriented training" OR "task oriented training" OR rehabilitation) OR SU (exercise* OR "physical exercise*" OR "physical activit*" OR "exercise training" OR "aerobic exercise*" OR "isometric exercise*" OR "resistance training" OR "strength training" OR "motor training" OR "task-oriented training" OR "task oriented training" OR rehabilitation))	339,932
#3	(TI ("mind-body" OR "mind body" OR "mind-body therap*" OR "mind body therap*" OR "mind-body exercise*" OR "mind body exercise*" OR tai chi OR taiji OR qigong OR baduanjin OR yoga OR pilates) OR AB ("mind-body" OR "mind body" OR "mind-body therap*" OR "mind body therap*" OR "mind-body exercise*" OR "mind body exercise*" OR tai chi OR taiji OR qigong OR baduanjin OR yoga OR pilates) OR SU ("mind-body" OR "mind body" OR "mind-body therap*" OR "mind body therap*" OR "mind-body exercise*" OR "mind body exercise*" OR tai chi OR taiji OR qigong OR baduanjin OR yoga OR pilates))	17,578
#4	(TI (dance OR dancing OR "dance therapy" OR ballet OR tango OR "jazz dance" OR "tap dance" OR "modern dance" OR "hip-hop" OR "hip hop" OR "line dancing" OR salsa OR "square dance") OR AB (dance OR dancing OR "dance therapy" OR ballet OR tango OR "jazz dance" OR "tap dance" OR "modern dance" OR "hip-hop" OR "hip hop" OR "line dancing" OR salsa OR "square dance") OR SU (dance OR dancing OR "dance therapy" OR ballet OR tango OR "jazz dance" OR "tap dance" OR "modern dance" OR "hip-hop" OR "hip hop" OR "line dancing" OR salsa OR "square dance"))	18,169
#5	(TI ("occupational therapy" OR "occupational therap*" OR ergotherapy OR ergotherapies) OR AB ("occupational therapy" OR "occupational therap*" OR ergotherapy OR ergotherapies) OR SU ("occupational therapy" OR "occupational therap*" OR ergotherapy OR	4,725

SPORTDiscus		
	ergotherapies))	
#6	(TI ("physical therapy" OR "physical therap*" OR physiotherapy OR physiotherap* OR "physical therapy modalit*" OR "physical therapy technique*" OR neurophysiotherapy OR "neurological physiotherapy" OR rehabilitation)<br>OR AB ("physical therapy" OR "physical therap*" OR physiotherapy OR physiotherap* OR "physical therapy modalit*" OR "physical therapy technique*" OR neurophysiotherapy OR "neurological physiotherapy" OR rehabilitation)<br>OR SU ("physical therapy" OR "physical therap*" OR physiotherapy OR physiotherap* OR "physical therapy modalit*" OR "physical therapy technique*" OR neurophysiotherapy OR "neurological physiotherapy" OR rehabilitation))	87,978
#7	(TI ("virtual reality" OR "virtual realit*" OR VR OR "immersive virtual reality" OR "non-immersive virtual reality" OR "augmented reality")<br>OR AB ("virtual reality" OR "virtual realit*" OR VR OR "immersive virtual reality" OR "non-immersive virtual reality" OR "augmented reality")<br>OR SU ("virtual reality" OR "virtual realit*" OR VR OR "immersive virtual reality" OR "non-immersive virtual reality" OR "augmented reality"))	3,187
#8	(TI ("video game*" OR videogame* OR "computer game*" OR exergam* OR "active video game*" OR "interactive game*" OR "serious game*" OR gaming)<br>OR AB ("video game*" OR videogame* OR "computer game*" OR exergam* OR "active video game*" OR "interactive game*" OR "serious game*" OR gaming)<br>OR SU ("video game*" OR videogame* OR "computer game*" OR exergam* OR "active video game*" OR "interactive game*" OR "serious game*" OR gaming))	5,840
#9	(TI (robot* OR robotics OR "robot-assisted" OR "robotic-assisted" OR "assistive robot*" OR telerobotics OR "soft robotics" OR "humanoid robot*" OR "social robot*" OR "companion robot*")<br>OR AB (robot* OR robotics OR "robot-assisted" OR "robotic-assisted" OR "assistive robot*" OR telerobotics OR "soft robotics" OR "humanoid robot*" OR "social robot*" OR "companion robot*")<br>OR SU (robot* OR robotics OR "robot-assisted" OR "robotic-assisted" OR "assistive robot*" OR telerobotics OR "soft robotics" OR "humanoid robot*" OR "social robot*" OR "companion robot*"))	3,112
#10	(TI ("computer-assisted therap*" OR "computer assisted therap*" OR "computer-assisted training" OR "computer assisted training" OR "computer-based therap*" OR "computer based therap*" OR "computer-based training" OR "computer based training" OR "digital therap*" OR "digital training" OR telerehabilitation OR "tele-rehabilitation")<br>OR AB ("computer-assisted therap*" OR "computer assisted therap*" OR "computer-assisted training" OR "computer assisted training" OR "computer-based	701

SPORTDiscus		
	therap*" OR "computer based therap*" OR "computer-based training" OR "computer based training" OR "digital therap*" OR "digital training" OR telerehabilitation OR "tele-rehabilitation")<br>OR SU ("computer-assisted therap*" OR "computer assisted therap*" OR "computer-assisted training" OR "computer assisted training" OR "computer-based therap*" OR "computer based therap*" OR "computer-based training" OR "computer based training" OR "digital therap*" OR "digital training" OR telerehabilitation OR "tele-rehabilitation"))	
#11	#2 OR #3 OR #4 OR #5 OR #6 OR #7 OR #8 OR #9 OR #10	316,514
#12	(TI (random* OR trial* OR RCT OR placebo* OR "controlled trial*" OR "clinical trial*" OR "randomized controlled trial*" OR "randomised controlled trial*" OR "controlled clinical trial*")<br>OR AB (random* OR trial* OR RCT OR placebo* OR "controlled trial*" OR "clinical trial*" OR "randomized controlled trial*" OR "randomised controlled trial*" OR "controlled clinical trial*")<br>OR SU (random* OR trial* OR RCT OR placebo* OR "controlled trial*" OR "clinical trial*" OR "randomized controlled trial*" OR "randomised controlled trial*" OR "controlled clinical trial*"))	136,079
#13	#1 AND #11 AND #12	358

Supplementary File 3: Detailed eligibility criteria and theory-based definitions of intervention nodes

Eligibility criteria and intervention-node definitions were specified before quantitative synthesis. The criteria below distinguish eligibility for the conventional network meta-analysis from the additional data requirements for the dose–response analysis. Intervention nodes were constructed to maximise clinical and mechanistic similarity within nodes and minimise overlap between nodes, consistent with Cochrane guidance for network meta-analysis[1,2].

S3.1 Detailed eligibility criteria

Studies were eligible when all population, intervention, comparator, outcome and study-design criteria were satisfied. Dose-reporting requirements affected eligibility for the dose–response analysis but did not determine eligibility for the conventional intervention-type network.

Supplementary Table 3.1. Detailed eligibility criteria and prespecified decision rules.

Domain	Inclusion criterion	Exclusion criterion or decision rule
Population	Adults with a clinical diagnosis of Parkinson's disease.	Studies without participants with Parkinson's disease; mixed neurological populations when data for the Parkinson's disease subgroup could not be extracted separately.
Intervention	A structured exercise or rehabilitation intervention assignable to one of the prespecified intervention nodes: UC, CULR, TOFDT, TAULHT, RT, MCT, MBE or AET.	Interventions that could not be assigned reproducibly to a prespecified node; mixed interventions when the independent contribution of the target exercise component could not be distinguished.
Comparator	Usual care or no structured exercise; another eligible exercise or rehabilitation intervention; or a different dose or prescription of an eligible exercise intervention.	Comparators outside the eligible intervention set or comparators for which the relevant treatment contrast could not be isolated.
Outcome	At least one eligible upper-limb or hand outcome, including finger dexterity, hand function, upper-limb motor performance or a related functional measure, with data that could be extracted or converted to an effect estimate.	No eligible upper-limb or hand outcome; outcome data insufficient to estimate an effect size after reasonable attempts at derivation or author contact.
Study design	Randomised controlled trial.	Non-randomised or observational study, single-arm study, protocol, review, editorial or commentary, or case report.
Dose–response subset	Arm-level information sufficient to estimate weekly exercise dose, including exercise type, session duration, weekly frequency and intensity or an adequate intensity proxy.	Trials with insufficient dose information remained eligible for the conventional intervention-type network meta-analysis but were excluded from the dose–response analysis.

Domain	Inclusion criterion	Exclusion criterion or decision rule
Language and report status	No language restriction. Full reports and other reports with sufficient methodological and numerical information were eligible.	Conference abstracts or other abbreviated reports were excluded when eligibility, intervention classification or effect estimation could not be established from the available information.
Multiple reports of one trial	All reports from the same trial were linked and treated as one study. The most complete report for the target outcome and primary post-intervention time point was used, with supplementary information drawn from companion reports when appropriate.	Duplicate participant data were not entered more than once.

S3.2 Theoretical basis and principles for intervention-node construction

Nodes were defined by the intervention's dominant therapeutic component and proposed mechanism of action rather than by the terminology used by individual trial authors. The classification framework incorporated the WHO International Classification of Functioning, Disability and Health, motor-learning and experience-dependent neuroplasticity principles, augmented-feedback theory, progressive-overload and cardiorespiratory-conditioning principles, the WHO multicomponent physical-activity framework, and the meditative-movement or mind–body integration framework[3–9].

Primary therapeutic target. The principal intended target was identified as general upper-limb function, task-specific finger dexterity, technology-mediated motor practice, muscular strength or power, cardiorespiratory fitness, integrated multidomain function, or mind–body regulation.

Defining training mechanism. Classification considered the active training mechanism, including nonspecific therapeutic exercise, task-specific practice, augmented feedback and adaptive practice, progressive resistance, sustained aerobic loading, integration of multiple co-primary exercise domains, or coordinated movement, breathing and attentional focus.

Dominant-component principle. A single-component or clearly dominant intervention was assigned to the node representing its principal active ingredient. A programme was assigned to MCT only when two or more distinct exercise domains were intentionally delivered as co-primary components and no single component clearly defined the programme.

Separation of intervention type from dose. Frequency, intensity, session duration, programme duration and progression did not create separate intervention-type nodes. These characteristics were retained for dose estimation and effect-modifier analyses.

Outcome-independent assignment. Node assignment was based on the intervention protocol and was not changed according to the direction, magnitude or statistical significance of the reported outcomes.

S3.3 Operational definitions of intervention nodes

Supplementary Table 3.3. Theory-based operational definitions, representative exercise modalities and classification boundaries for intervention nodes.

Intervention node	Recognised theoretical framework	Operational definition	Representative exercise modalities
Usual care / no structured exercise (UC)	Reference condition; absence of a planned, progressive exercise stimulus.	Routine medical or nursing care, continuation of usual activities, education, a wait-list condition, or no additional intervention, without a prescribed and repeated structured exercise or rehabilitation programme.	Usual activities; routine clinical care; education-only control; wait-list; no additional exercise.
Conventional upper-limb rehabilitation (CULR)	WHO ICF; impairment-oriented and activity-oriented therapeutic rehabilitation[3,8].	Conventional upper-limb therapeutic exercise targeting mobility, range of motion, flexibility, general coordination, sensorimotor control, reaching, grasping or basic functional use, without another defining training mechanism dominating the programme.	Range-of-motion and stretching exercises; general reaching and grasping; coordination exercises; sensory-motor training; conventional physical or occupational therapy; basic activities-of-daily-living practice.
Task-oriented finger dexterity training (TOFDT)	Motor-learning theory; specificity of practice; experience-dependent neuroplasticity[4,8].	Repeated, goal-directed practice of finger, hand or fine-motor tasks that closely resemble the target skill, with progression in speed, accuracy, coordination, complexity or task difficulty. Fine-motor skill acquisition is the primary therapeutic target.	Finger-sequencing and tapping drills; pegboard practice; handwriting; buttoning; coin handling; object manipulation; precision grip; timed manual dexterity tasks.
Technology-assisted upper-limb/hand training (TAULHT)	Motor-learning theory; augmented-feedback framework; repetitive adaptive practice; experience-dependent neuroplasticity[4,5,8].	Upper-limb or hand training substantially mediated by interactive technology that provides real-time or summary feedback, task adaptation, movement assistance, resistance, performance scoring or high-repetition practice. Technology is a defining therapeutic delivery mechanism.	Virtual-reality training; exergaming; robotic or electromechanical upper-limb practice; motion-tracking games; sensor-based biofeedback; interactive haptic, visual or auditory feedback systems.
Resistance training (RT)	Progressive-overload principle; specificity of muscular adaptation[6,8].	Repeated voluntary muscle contractions against external or body-mass resistance, with resistance prescribed and usually progressed to increase muscular strength, power or endurance. Muscular loading is the dominant training stimulus.	Free weights; resistance machines; elastic bands; weighted functional tasks; body-weight resistance; isokinetic training; concentric, eccentric or power-oriented resistance exercise.
Multicomponent training (MCT)	WHO multicomponent physical-activity framework; systems-oriented functional training[6,8].	An intentionally integrated programme containing at least two distinct co-primary exercise domains—such as aerobic, resistance, balance, flexibility, coordination or functional task training—with no single component clearly defining the intervention.	Aerobic–strength–balance circuits; combined endurance and resistance programmes; functional circuit training; integrated mobility, balance, coordination and strengthening programmes.
Mind–body exercise (MBE)	Meditative-movement and mind–body integration framework; attentional and sensorimotor regulation[7,9].	Structured exercise that explicitly integrates controlled movement or posture with breathing, attentional focus, body awareness, relaxation or mental regulation. Coordinated mind–body practice is a defining component, rather than an incidental feature.	Tai Chi; Qigong; Baduanjin; yoga; Pilates; other structured meditative-movement practices.
Aerobic exercise training (AET)	Cardiorespiratory-conditioning, specificity and progressive-overload principles[6,8].	Continuous or interval-based rhythmic, dynamic exercise involving large muscle groups, primarily prescribed to elevate heart rate and oxygen consumption and improve cardiorespiratory endurance. Aerobic conditioning is the dominant training goal.	Brisk walking; treadmill walking; jogging or running; cycling; swimming; rowing; elliptical training; aerobic dance; arm-crank ergometry.

Supplementary File 4: Conversion of reported summary statistics and estimation of change-score standard deviations

S4.1 Conversion of reported summary statistics to means and standard deviations

Supplementary Table 4.1. Original summary statistics and conversion formulas used to derive means and standard deviations.

Study	Outcome	Intervention group		Control group		Formulae	Data format
		Baseline	Post-intervention	Baseline	Post-intervention		
Raciti 2022	Upper-limb function	48 (45.25–51)	52 (50.25–54)	56 (50–57.5)	57 (52.26–59)	Mean ≈ Median SD ≈ (Q3 − Q1) / 1.35	median (IQR)
Çetin 2024	Finger dexterity	28.6 (26.0–33.6)	26.0 (23.3–31.0)	30.1 (27.1–33.8)	28.6 (26.4–32.6)	Mean ≈ Median SD ≈ (Q3 − Q1) / 1.35	median (IQR)
	Upper-limb function	115.0 (94.6–143.5)	102.7 (88.7–128.0)	122.5 (101.2–149.0)	118.7 (94.8–141.7)	Mean ≈ Median SD ≈ (Q3 − Q1) / 1.35	median (IQR)
van der Kolk 2019	Finger dexterity	19.5 (0.9)	18.8 (0.7)	19.6 (0.9)	19.4 (0.7)	SD=SE×√n	mean (SE)
Martinazzo 2025	Upper-limb function	27.3 (3.1)	31.9 (3)	24.2 (3)	24.2 (2.8)	SD=SE×√n	mean (SE)

Note: All conversions of medians and interquartile ranges to means and standard deviations, and of SEs/SEMs to standard deviations, were performed strictly in accordance with the formulas provided in the Cochrane Handbook for Systematic Reviews of Interventions[10].

S4.2 Estimation of change-score standard deviations

Supplementary Table 4.2A. Outcome-specific correlation coefficients used in the primary analysis.

Outcome	Correlation coefficient (R)
Finger dexterity	0.840
Upper-limb function	0.741

Supplementary Table 4.2B. Study-level data used to derive r for finger dexterity.

Study	Group	Total (n)	Baseline SD	Final SD	Reported SD of change	Derived r	n used to derive r
Choi 2022	intervention	7	37.68	22.41	15.27	1.000	7
Choi 2022	control	7	24.54	22.09	2.45	1.000	7
Zare 2022	intervention	17	12.06	9.67	4.17	0.950	17

Study	Group	Total (n)	Baseline SD	Final SD	Reported SD of change	Derived r	n used to derive r
Zare 2022	control	16	6.11	2.03	4.23	0.950	16
Vorasoot 2019	intervention	23	38.53	34.02	27.17	0.726	23
Vorasoot 2019	control	23	56.19	48.88	16.75	0.959	23
Vanbellingen 2017	intervention	48	7.09	9.23	4.20	0.900	48
Vanbellingen 2017	control	44	7.81	12.95	6.83	0.900	44
van der Kolk 2019	intervention	61	7.26	5.64	5.64	0.644	61
Nackaerts 2017	intervention	18	8.72	26.60	22.59	0.589	18
Nackaerts 2017	control	20	12.51	6.61	9.57	0.657	20
Demirgöz Demir 2025	intervention	15	10.50	9.40	3.30	0.951	15
Demirgöz Demir 2025	control	15	14.20	13.50	2.40	0.986	15
Heinzle 2025	intervention	28	8.74	4.92	4.81	0.901	28
Heinzle 2025	control	29	7.55	2.85	5.13	0.902	29
Caglar 2005	intervention	15	16.70	11.10	7.86	0.918	15
Chen 2023	intervention	15	3.58	3.05	1.19	0.948	15
Chen 2023	control	14	2.41	2.27	0.97	0.916	14

Supplementary Table 4.2C. Study-level data used to derive r for upper-limb function.

Study	Group	Total (n)	Baseline SD	Final SD	Reported SD of change	Derived r	n used to derive r
Cabrera-Martos 2018	intervention	25	0.39	7.42	7.48	-0.128	50
Choi 2022	intervention	7	21.80	19.02	2.78	1.000	7
Choi 2022	control	7	11.92	11.58	0.34	1.000	7
Mishra 2022	intervention	3	18.77	22.35	10.11	0.893	3
Mishra 2022	control	3	36.17	33.47	6.08	0.988	3
Zare 2022	intervention	17	4.54	5.39	3.24	0.800	17
Zare 2022	control	16	2.91	2.45	1.75	0.800	16
Vanbellingen 2017	intervention	48	8.75	11.11	5.00	0.900	48
Vanbellingen 2017	control	44	10.79	12.42	5.43	0.900	44
Heinzle 2025	intervention	28	15.59	20.24	5.85	0.980	28
Heinzle 2025	control	29	9.36	10.59	2.34	0.980	29

Supplementary File 5: Exercise dose estimation, MET assignment, dose node mapping, and arm-level data preparation for dose–response analyses

S5.1 Definition and calculation of weekly exercise dose

Exercise dose was derived at the study-arm level from the prescribed intervention schedule and expressed as metabolic equivalent of task minutes per week (MET·min/week). For a single-component intervention, weekly dose was calculated as follows:

Weekly exercise dose (MET·min/week) = assigned MET × session duration (min/session) × intervention frequency (sessions/week)

For multicomponent or interval interventions, component-specific doses were calculated separately and summed as $\Sigma(\text{MET}_i \times \text{duration}_i \times \text{frequency}_i)$. When an intervention contained components of different intensities within the same session, the equivalent session MET was calculated as $\Sigma(\text{MET}_i \times \text{duration}_i)/\Sigma\text{duration}_i$. Warm-up and cool-down activities were included only when their activity type and duration were identifiable and formed part of the analysed session duration. Active exercise comparators were assigned doses from their own prescriptions. Usual care, no intervention, or care without prescribed structured exercise was assigned 0 MET·min/week.

The primary analysis used prescribed rather than adherence-adjusted dose because achieved session-level exposure was not reported consistently across trials. Within-session stages were time weighted when sufficient detail was available. If a prescription varied across intervention phases, phase-specific weekly doses were averaged over the reported intervention weeks. Calculations retained the available numerical precision; rounding for presentation did not alter dose-node assignment.

S5.2 Hierarchical assignment of MET values

A standard MET was defined as 3.5 mL O_2 $\cdot\text{kg}^{-1}\cdot\text{min}^{-1}$. MET values were assigned using a prespecified hierarchy. Directly reported physiological or workload information took precedence over activity-based imputation; otherwise, the 2024 Adult Compendium of Physical Activities was used as the primary crosswalk, supplemented by American College of Sports Medicine metabolic equations when the required parameters were available[11,12]. The hierarchy and corresponding assignment rules are summarised in Supplementary Table 5.2.

Supplementary Table 5.2. Hierarchical rules for study-arm–level MET assignment and derivation.

MET-derivation tier	Available information	Assignment rule
Tier A	Directly reported MET or VO_2; sufficient speed, grade, power, and body-mass data; or the prespecified zero-dose definition.	Use the reported value or an ACSM metabolic equation; convert oxygen uptake as MET = VO_2/3.5 when required; assign 0 MET for usual care or no prescribed structured exercise.
Tier B	Exercise mode, intensity, and training structure correspond to a specific or closely matching activity in	Use the MET value of the closest applicable Compendium activity.

MET-derivation tier	Available information	Assignment rule
	the 2024 Adult Compendium.	
Tier C	No exact entry is available for the rehabilitation task, virtual reality or robotic exercise, interval programme, or multicomponent training.	Use closest-activity analogy, interpolation between adjacent entries, or time weighting of reported components or stages.

Exercise intensity was classified as low (<3.0 MET), moderate (3.0–5.9 MET), or vigorous (≥6.0 MET). Heart rate, percentage of heart-rate reserve, percentage of maximal heart rate, Borg rating of perceived exertion, and percentage of one-repetition maximum were used to assess the plausibility of the assigned intensity but were not treated as directly reported MET values[12–15]. The MET-derivation tiers describe the directness of the conversion pathway and do not represent trial quality, risk of bias, or certainty of evidence.

S5.3 Handling of exercise and rehabilitation modalities

For aerobic exercise, directly reported MET, oxygen uptake, or calculable workload was preferred; otherwise, the closest walking, treadmill, or stationary-cycling entry was used. Resistance exercise was matched to the corresponding resistance-training entry, with repetitions, percentage of one-repetition maximum, or perceived exertion used to verify intensity. Mind–body exercise was matched according to the specific form, including tai chi, qigong, yoga, or Pilates. Hand training, conventional upper-limb rehabilitation, virtual reality, active video gaming, and robotic training were mapped according to the body region involved, degree of active movement, task demands, and training composition; the device name alone was not used to determine MET.

No Parkinson disease-specific compendium provides continuous MET values for all included exercise and rehabilitation modalities. General activity compendia and metabolic equations were therefore used for numerical standardisation, whereas Parkinson disease-specific physiological or accelerometer evidence informed plausibility checks where relevant[14,15].

S5.4 Mapping exact weekly doses to prespecified dose nodes

Outcome-specific dose nodes were defined before model estimation to maintain network connectivity while reflecting the observed distribution of exact weekly doses. Exact doses were assigned to the nearest node using the midpoint between adjacent nodes as the boundary. The prespecified nodes and midpoint boundaries are reported in Supplementary Table 5.4.

Supplementary Table 5.4. Prespecified outcome-specific weekly exercise-dose nodes and midpoint boundaries.

Outcome	Prespecified dose nodes (MET·min/week)	Midpoint boundaries (MET·min/week)
Finger dexterity	0, 180, 450, 700, 1,600	90, 315, 575, 1,150
Upper-limb function	0, 180, 450, 700, 1,575	90, 315, 575, 1,137.5

Intervals were right closed at each midpoint; for example, a finger-dexterity dose >90 and ≤315 MET·min/week was assigned to the 180-MET·min/week node. The exact weekly dose was retained

alongside the assigned node for description and audit; the assigned node representative value, rather than the exact weekly dose, was used as the primary dose–response model input.

S5.5 Traceability and interpretation

Supplementary Tables 5.6A and 5.6B report, for each eligible study arm, the assigned MET, session duration, weekly frequency, exact weekly dose, assigned dose node, and outcome data; the assigned node representative value was used as the primary model input. Supplementary Tables 5.7A and 5.7B provide the corresponding exercise prescription, authoritative tool, activity entry, MET-derivation method, and assigned MET value. Weekly doses were recalculated and checked against the prespecified node boundaries before analysis.

MET values derived through analogy, interpolation, or component weighting should be interpreted as standardised exposure estimates rather than directly measured energy expenditure. Consequently, a model-derived within-range maximum effect, near-maximum interval, or dose node should not be interpreted as an absolute physiological threshold or a clinical exercise prescription.

S5.6 Arm-level dose and outcome data for dose–response analyses

Supplementary Table 5.6A. Arm-level exercise dose, dose-node assignment, and outcome data for finger dexterity.

Study	Study arm	Exercise-type node	Assigned MET	Exercise intensity	Intervention frequency (sessions/week)	Session duration (min/session)	Exact weekly dose (MET·min/week)	Assigned dose node (MET·min/week)	Mean-change	y	se	Measurement tool	lower better
Allen 2017	Intervention	TAULHT	3.5	Moderate	3	45	472.5	450	0.20	-0.196	0.996	9HPT	yes
	Control	UC	0	NA	0	0	0	0	0.50	-0.489	0.941	9HPT	yes
Cabrera-Martos 2018	Intervention	TOFDT	3	Moderate	2	45	270	180	2.10	2.067	0.235	PPT	no
	Control	CULR	3	Moderate	2	45	270	180	-0.89	-0.876	0.396	PPT	no
Choi 2022	Intervention	TOFDT	3.5	Moderate	5	40	700	700	-11.90	11.140	5.403	9HPT	yes
	Control	CULR	2.3	Low	5	40	460	450	-2.63	2.462	0.867	9HPT	yes
Fernández-González 2019	Intervention	TAULHT	3	Moderate	2	30	180	180	4.33	4.173	1.226	PPT	no
	Control	CULR	3	Moderate	2	30	180	180	1.00	0.964	0.491	PPT	no
Hashemi 2022	Intervention	TAULHT	3	Moderate	3	90	810	700	-2.33	2.267	0.916	9HPT	yes
	Control	CULR	3	Moderate	3	90	810	700	-3.98	3.872	1.532	9HPT	yes
Horin 2019	Intervention	TAULHT	3	Moderate	7	30	630	700	-0.42	0.411	0.761	9HPT	yes
	Control	UC	0	NA	0	0	0	0	-0.48	0.470	0.513	9HPT	yes
Park 2021	Intervention	TOFDT	3.8	Moderate	3	50	570	450	-4.04	3.729	2.495	9HPT	yes
	Control	UC	0	NA	0	0	0	0	0.69	-0.637	1.235	9HPT	yes
Taghizadeh 2017	Intervention	TOFDT	3	Moderate	5	120	1800	1600	1.60	1.568	0.382	PPT	no
	Control	UC	0	NA	0	0	0	0	-0.15	-0.147	0.396	PPT	no
Zare 2022	Intervention	TOFDT	3	Moderate	5	120	1800	1600	-12.36	12.059	0.987	9HPT	yes
	Control	RT	3.5	Moderate	5	90	1575	1600	-11.35	11.073	1.032	9HPT	yes
Vorasoot 2019	Intervention	TOFDT	1.3	Low	7	45	409.5	450	-29.13	28.631	5.568	HW time	yes
	Control	UC	0	NA	0	0	0	0	5.17	-5.081	3.433	HW time	yes
Vanbellingen 2017	Intervention	TOFDT	2	Low	5	30	300	180	-2.11	2.092	0.601	9HPT	yes
	Control	RT	3.5	Moderate	5	30	525	450	0.58	-0.575	1.021	9HPT	yes
Raciti 2022	Intervention	TAULHT	3	Moderate	6	90	1620	1600	-8.10	7.821	2.301	9HPT	yes
	Control	CULR	2.9	Low	6	90	1566	1600	-3.70	3.572	1.192	9HPT	yes
Collett 2017	Intervention	MCT	4.25	Moderate	2	60	510	450	0.70	-0.694	0.445	9HPT	yes
	Control	TOFDT	2	Low	2	60	240	180	-1.20	1.190	0.494	9HPT	yes
van der Kolk 2019	Intervention	AET	6	Vigorous	3	30	540	450	-0.40	0.398	0.718	Pegboard test	yes
	Control	CULR	2.3	Low	3	30	207	180	-0.04	0.040	0.701	Pegboard test	yes

Study	Study arm	Exercise-type node	Assigned MET	Exercise intensity	Intervention frequency (sessions/week)	Session duration (min/session)	Exact weekly dose (MET·min/week)	Assigned dose node (MET·min/week)	Mean-change	y	se	Measurement tool	lower better
van der Kolk 2017	Intervention	AET	4.74	Moderate	3	30	426.6	450	0.60	-0.586	0.371	Pegboard test	yes
	Control	UC	0	NA	0	0	0	0	0.90	-0.879	0.681	Pegboard test	yes
Çetin 2024	Intervention	TAULHT	3.5	Moderate	3	50	525	450	-2.63	2.519	1.128	9HPT	yes
	Control	CULR	3	Moderate	3	50	450	450	-1.13	1.082	0.958	9HPT	yes
Nackaerts 2017	Intervention	TOFDT	1.3	Low	5	30	195	180	10.35	-10.133	5.213	Normalized jerk	yes
	Control	CULR	2.3	Low	5	30	345	450	-5.32	5.208	2.095	Normalized jerk	yes
Kadkhodaie 2020	Intervention	RT	3.5	Moderate	3	40	420	450	-45.00	43.200	18.470	RHT amplitude	yes
	Control	UC	0	NA	0	0	0	0	-0.50	0.480	24.350	RHT amplitude	yes
Duncan 2012	Intervention	MBE	3.5	Moderate	2	60	420	450	-3.40	3.349	1.103	9HPT	yes
	Control	UC	0	NA	0	0	0	0	0.70	-0.689	1.178	9HPT	yes
Robles-García 2016	Intervention	TAULHT	1.5	Low	3	30	135	180	2.20	2.071	0.640	FT amplitude	no
	Control	TOFDT	1.5	Low	3	30	135	180	1.60	1.506	0.757	FT amplitude	no
Marusiak 2019	Intervention	AET	3.44	Moderate	3	40	412.8	450	-1.10	1.054	0.086	UPDRS-23	yes
	Control	UC	0	NA	0	0	0	0	0.60	-0.575	0.148	UPDRS-23	yes
Schaeffer 2019	Intervention	TOFDT	2	Low	1	60	120	180	-3.50	3.430	1.149	Pegboard test	yes
	Control	UC	0	NA	0	0	0	0	-5.40	5.293	2.712	Pegboard test	yes
Demirgöz Demir 2025	Intervention	TAULHT	2.88	Low	5	120	1728	1600	-6.60	6.422	0.829	9HPT	yes
	Control	CULR	3	Moderate	5	90	1350	1600	-4.20	4.086	0.603	9HPT	yes
Heinzle 2025	Intervention	TAULHT	2.8	Low	4	30	336	450	-2.24	2.209	0.897	9HPT	yes
	Control	CULR	2.8	Low	4	30	336	450	-2.69	2.653	0.940	9HPT	yes
Raciti 2025	Intervention	TAULHT	2.3	Low	6	90	1242	1600	-2.40	2.352	2.304	9HPT	yes
	Control	CULR	2.3	Low	6	90	1242	1600	-4.10	4.019	0.732	9HPT	yes
Li 2024	Intervention	MBE	3	Moderate	5	30	450	450	2.24	2.208	0.861	PPT	no
	Control	UC	0	NA	0	0	0	0	-0.02	-0.020	0.678	PPT	no
Caglar 2005	Intervention	MCT	3.8	Moderate	7	60	1596	1600	-9.00	8.757	1.975	9HPT	yes
	Control	UC	0	NA	0	0	0	0	0.47	-0.457	1.269	9HPT	yes
Chen 2023	Intervention	TOFDT	3	Moderate	1	120	360	450	1.02	0.991	0.299	PPT	no
	Control	UC	0	NA	0	0	0	0	-0.34	-0.330	0.252	PPT	no
Eldemir 2023	Intervention	TOFDT	3.8	Moderate	3	60	684	700	-6.85	6.665	0.628	9HPT	yes
	Control	MCT	3.8	Moderate	3	60	684	700	0.05	-0.049	0.997	9HPT	yes

Study	Study arm	Exercise-type node	Assigned MET	Exercise intensity	Intervention frequency (sessions/week)	Session duration (min/session)	Exact weekly dose (MET·min/week)	Assigned dose node (MET·min/week)	Mean-change	y	se	Measurement tool	lower better
Moraes 2024	Intervention	MCT	4.3	Moderate	2	60	516	450	-0.04	0.040	0.013	FT-LH-EA	yes
	Control	TOFDT	1.5	Low	2	60	180	180	-0.01	0.010	0.008	FT-LH-EA	yes

Supplementary Table 5.6B. Arm-level exercise dose, dose-node assignment, and outcome data for upper-limb function.

Study	Study arm	Exercise-type node	Assigned MET	Exercise intensity	Intervention frequency (sessions/week)	Session duration (min/session)	Exact weekly dose (MET·min/week)	Assigned dose node (MET·min/week)	Mean-change	y	se	Measurement tool	lower better
Allen 2017	Intervention	TAULHT	3.5	Moderate	3	45	472.5	450	0.70	0.685	1.696	BBT	no
	Control	UC	0	NA	0	0	0	0	-0.90	-0.881	1.373	BBT	no
Cabrera-Martos 2018	Intervention	TOFDT	3	Moderate	2	45	270	180	17.36	17.087	1.473	GAS	no
	Control	CULR	3	Moderate	2	45	270	180	4.03	3.967	1.266	GAS	no
Choi 2022	Intervention	TOFDT	3.5	Moderate	5	40	700	700	-8.57	8.023	0.984	ADL-Q	yes
	Control	CULR	2.3	Low	5	40	460	450	-2.57	2.406	0.120	ADL-Q	yes
Fernández-González 2019	Intervention	TAULHT	3	Moderate	2	30	180	180	4.00	3.855	3.792	BBT	no
	Control	CULR	3	Moderate	2	30	180	180	6.00	5.783	3.005	BBT	no
Hashemi 2022	Intervention	TAULHT	3	Moderate	3	90	810	700	3.60	3.503	1.756	BBT	no
	Control	CULR	3	Moderate	3	90	810	700	4.26	4.145	1.456	BBT	no
Mishra 2022	Intervention	TOFDT	3	Moderate	3	50	450	450	-26.33	21.064	4.670	JTHFT	yes
	Control	CULR	3	Moderate	3	50	450	450	-14.00	11.200	2.808	JTHFT	yes
Taghizadeh 2017	Intervention	TOFDT	3	Moderate	5	120	1800	1575	7.70	7.547	1.715	BBT	no
	Control	UC	0	NA	0	0	0	0	-1.85	-1.813	2.277	BBT	no
Zare 2022	Intervention	TOFDT	3	Moderate	5	120	1800	1575	-4.95	4.829	0.767	JTHFT	yes
	Control	RT	3.5	Moderate	5	90	1575	1575	-3.16	3.083	0.427	JTHFT	yes
Vanbellingen 2017	Intervention	TOFDT	2	Low	5	30	300	180	-3.22	3.193	0.716	DextQ-24	yes
	Control	RT	3.5	Moderate	5	30	525	450	-0.71	0.704	0.812	DextQ-24	yes
Raciti 2022	Intervention	TAULHT	3	Moderate	6	90	1620	1575	4.00	3.862	0.793	FMA-UE	no
	Control	CULR	2.9	Low	6	90	1566	1575	1.59	1.535	1.457	FMA-UE	no
Çetin 2024	Intervention	TAULHT	3.5	Moderate	3	50	525	450	-7.27	6.963	0.990	MMDT turning test	yes
	Control	CULR	3	Moderate	3	50	450	450	-4.43	4.243	1.536	MMDT turning test	yes
Lorek 2025	Intervention	AET	3.3	Moderate	3	40	396	450	-1.00	0.971	0.324	MBS-AUE	yes
	Control	UC	0	NA	0	0	0	0	-0.06	0.058	0.407	MBS-AUE	yes

Study	Study arm	Exercise-type node	Assigned MET	Exercise intensity	Intervention frequency (sessions/week)	Session duration (min/session)	Exact weekly dose (MET·min/week)	Assigned dose node (MET·min/week)	Mean-change	y	se	Measurement tool	lower better
Marusiak 2019	Intervention	AET	3.44	Moderate	3	40	412.8	450	-1.80	1.724	0.246	DLMF-UPDRS 8–11	yes
	Control	UC	0	NA	0	0	0	0	-0.30	0.287	0.349	DLMF-UPDRS 8–11	yes
Schaeffer 2019	Intervention	TOFDT	2	Low	1	60	120	180	-1.90	1.862	0.562	MDS-UPDRS Part II	yes
	Control	UC	0	NA	0	0	0	0	-0.30	0.294	0.591	MDS-UPDRS Part II	yes
Demirgöz Demir 2025	Intervention	TAULHT	2.88	Low	5	120	1728	1575	-21.00	20.432	3.407	DASH	yes
	Control	CULR	3	Moderate	5	90	1350	1575	-13.50	13.135	2.338	DASH	yes
Heinzle 2025	Intervention	TAULHT	2.8	Low	4	30	336	450	3.83	3.778	1.090	BBT	no
	Control	CULR	2.8	Low	4	30	336	450	3.67	3.620	0.429	BBT	no
Raciti 2025	Intervention	TAULHT	2.3	Low	6	90	1242	1575	2.95	2.891	0.452	FMA-UE hand	no
	Control	CULR	2.3	Low	6	90	1242	1575	1.10	1.078	0.292	FMA-UE hand	no
Pazzaglia 2020	Intervention	TAULHT	3	Moderate	3	40	360	450	-8.00	7.877	2.352	DASH	yes
	Control	MCT	3	Moderate	3	40	360	450	-5.20	5.120	2.392	DASH	yes
Moraes 2024	Intervention	MCT	4.3	Moderate	2	60	516	450	-10.00	9.889	7.929	FT-LH-EA	yes
	Control	TOFDT	1.5	Low	2	60	180	180	-5.00	4.945	6.834	FT-LH-EA	yes
Eldemir 2023	Intervention	TOFDT	3.8	Moderate	3	60	684	700	-15.66	15.237	2.756	JHFT-DS	yes
	Control	MCT	3.8	Moderate	3	60	684	700	-1.82	1.771	2.829	JHFT-DS	yes
Leal 2019	Intervention	RT	3.5	Moderate	2	40	280	180	4.52	4.454	0.962	HGS	no
	Control	UC	0	NA	0	0	0	0	-1.69	-1.666	0.732	HGS	no
Zhang 2026	Intervention	MBE	3	Moderate	4	60	720	700	12.29	11.958	0.268	HGS	no
	Control	UC	0	NA	0	0	0	0	-3.06	-2.977	0.263	HGS	no
Martinazzo 2025	Intervention	MBE	3	Moderate	2	60	360	450	4.60	4.434	2.118	HGS	no
	Control	UC	0	NA	0	0	0	0	0.00	0.000	2.020	HGS	no

Note for Supplementary Tables 5.6A and 5.6B: Each row represents one study arm; blank cells in the "Study" column indicate additional arms of the study named immediately above. Exact weekly dose (MET·min/week) was calculated as assigned MET × session duration × weekly frequency, and dose-node assignment followed Supplementary Table 5.4. Usual care or no prescribed structured exercise was coded as 0 across dose fields, with exercise intensity recorded as not applicable. Intensity was classified as low (<3.0 MET), moderate (3.0–5.9 MET), or vigorous (≥6.0 MET).

"Mean-change" denotes the post-intervention minus baseline arm-level change; study-reported changes were preferred and harmonised to this convention. A negative Mean-change indicates improvement when "Lower better" = yes, and a positive value when "Lower better" = no. Outcome direction was harmonised as $y = J \times s \times$ Mean-change, where J is Hedges' small-sample correction and $s = -1$ for lower-better outcomes and +1 otherwise; positive y indicates within-arm improvement. The standard error was calculated as $se = J \times SD\_change/\sqrt{n}$. The y and se values are original-scale model inputs; the dose–response model used the study-specific pooled change-score SD (standsd) to standardise treatment effects. Reported SD_change values were used when available; otherwise, $SD\_change = \sqrt{(SD\_baseline^2 + SD\_post^2 - 2R \times SD\_baseline \times SD\_post)}$, with R = 0.84 for finger dexterity and 0.741 for upper-limb function. The derivation of these correlations is reported in Supplementary File 4. Mean-change is displayed to two decimal places and y and se to three; analyses used unrounded values.

Abbreviations: AET, aerobic exercise training; CULR, conventional upper-limb rehabilitation; MBE, mind–body exercise; MCT, multicomponent training; MET, metabolic equivalent of task; NA, not applicable; RT, resistance training; SD, standard deviation; TAULHT, technology-assisted upper-limb or hand training; TOFDT, task-oriented finger dexterity training; UC, usual care or no structured exercise.

S5.7 Study-arm-level exercise prescriptions and MET derivations

Supplementary Table 5.7A. Arm-level exercise prescriptions and MET assignments in studies reporting finger dexterity outcomes.

Study	Study arm	Exercise-type node	Reported intensity or prescription (condensed)	Authoritative tool	Activity entry	MET derivation	Assigned MET
Allen 2017	Intervention	TAULHT	Upper-limb exergaming controlled by finger or unilateral arm movements; 3 sessions/week for 12 weeks.	2024 Adult Compendium of Physical Activities	Codes 22200 (3.0 MET, upper-body active video game) and 22240 (4.0 MET, total-body active video game)	Midpoint: (3.0 + 4.0)/2.	3.5
	Control	UC	Usual care and normal activities; no structured exercise prescribed.	Trial protocol; prespecified zero-dose rule	No structured exercise/usual care (0 MET)	No structured exercise; zero-dose rule.	0.0
Cabrera-Martos 2018	Intervention	TOFDT	Patient-specific, goal-oriented upper-limb functional training at home; 45 min/session, 2 sessions/week for 4 weeks.	2024 Adult Compendium of Physical Activities	Codes 02056 and 22200 (general therapeutic task or active upper-body game, 3.0 MET)	Closest-activity crosswalk.	3.0
	Control	CULR	Standardized home-based range-of-motion, dexterity, and stretching exercises; 45 min/session, 2 sessions/week for 4 weeks.	2024 Adult Compendium of Physical Activities	Codes 02115 and 02056 (light upper-body and general therapeutic exercise), composite ≈3.0 MET	Closest-activity crosswalk.	3.0
Choi 2022	Intervention	TOFDT	Conventional occupational therapy (30 min) plus task-oriented LSVT-BIG training (40 min); 5 sessions/week for 4 weeks.	2024 Adult Compendium of Physical Activities	Codes 02064 and 02022 (general home exercise or moderate calisthenics, 3.8 MET)	Composite-activity crosswalk; standardized per coding rule.	3.5
	Control	CULR	Conventional occupational therapy (30 min) plus relaxation and stretching (40 min); 5 sessions/week for 4 weeks.	2024 Adult Compendium of Physical Activities	Code 02101 (mild stretching, 2.3 MET)	Direct activity-code match.	2.3
Fernández-González 2019	Intervention	TAULHT	Leap Motion–based upper-limb serious games involving reaching, grasping, pinching, and forearm rotation; 30 min/session, 2 sessions/week for 6 weeks.	2024 Adult Compendium of Physical Activities	Code 22200 (active video game using upper body, light-to-moderate, 3.0 MET)	Direct activity-code match.	3.0
	Control	CULR	Conventional upper-limb physiotherapy with mobility, strengthening, stretching, and functional-task practice; session intensity not otherwise quantified.	2024 Adult Compendium of Physical Activities	Codes 02115 and 02056 (light upper-body and general therapeutic exercise), composite ≈3.0 MET	Closest-activity crosswalk.	3.0
Hashemi 2022	Intervention	TAULHT	Kinect-based upper-limb virtual-reality training; 24 sessions (3/week), with supervised and unsupervised protocols reported.	2024 Adult Compendium of Physical Activities	Code 22200 (active video game using upper body, light-to-moderate, 3.0 MET)	Direct activity-code match.	3.0
	Control	CULR	Conventional rehabilitation; 90 min/session, 3 sessions/week.	2024 Adult Compendium of Physical Activities	Codes 02115 and 02056 (light upper-body and general therapeutic exercise), composite ≈3.0 MET	Closest-activity crosswalk.	3.0

Study	Study arm	Exercise-type node	Reported intensity or prescription (condensed)	Authoritative tool	Activity entry	MET derivation	Assigned MET
Horin 2019	Intervention	TAULHT	App-based hand/dexterity exercises, including digital peg, pinch, and stylus tasks; approximately 30 min/day for 12 weeks.	2024 Adult Compendium of Physical Activities	Code 22200 (active video game using upper body, light-to-moderate, 3.0 MET)	Direct activity-code match.	3.0
	Control	UC	Normal daily activities; no structured exercise prescribed.	Trial protocol; prespecified zero-dose rule	No structured exercise/usual care (0 MET)	No structured exercise; zero-dose rule.	0.0
Park 2021	Intervention	TOFDT	Seated dual-task drumming with rhythmic cueing and task switching; 50 min/session, 3 sessions/week for 12 weeks.	2024 Adult Compendium of Physical Activities	Code 10040 (drums, sitting, 3.8 MET)	Direct activity-code match.	3.8
	Control	UC	Usual community rehabilitation without music or cognitive training; no dose-coded structured exercise.	Trial protocol; prespecified zero-dose rule	No structured exercise/usual care (0 MET)	No structured exercise; zero-dose rule.	0.0
Taghizadeh 2017	Intervention	TOFDT	Sensory–motor hand training plus usual rehabilitation; 10 sessions (5/week) over 2 weeks.	2024 Adult Compendium of Physical Activities	Codes 02056 and 22200 (general therapeutic task or active upper-body game, 3.0 MET)	Closest-activity crosswalk.	3.0
	Control	UC	Usual rehabilitation without a separately prescribed structured exercise dose.	Trial protocol; prespecified zero-dose rule	No structured exercise/usual care (0 MET)	No structured exercise; zero-dose rule.	0.0
Zare 2022	Intervention	TOFDT	Activity-based functional training for 6 weeks; session intensity not otherwise quantified.	2024 Adult Compendium of Physical Activities	Codes 02056 and 22200 (general therapeutic task or active upper-body game, 3.0 MET)	Closest-activity crosswalk.	3.0
	Control	RT	Progressive strengthening using a power web and elastic resistance; approximately 90 min/day for 6 weeks.	2024 Adult Compendium of Physical Activities	Code 02054 (resistance training, multiple exercises, 8–15 reps, 3.5 MET)	Direct activity-code match.	3.5
Vorasoot 2019	Intervention	TOFDT	Home handwriting practice using a structured workbook; 5 pages/day for 4 weeks.	2024 Adult Compendium of Physical Activities	Code 09040 (sitting: writing, desk work, typing, 1.3 MET)	Direct activity-code match.	1.3
	Control	UC	No-treatment control; no structured exercise prescribed.	Trial protocol; prespecified zero-dose rule	No structured exercise/usual care (0 MET)	No structured exercise; zero-dose rule.	0.0
Vanbellingen 2017	Intervention	TOFDT	High-intensity, task-specific home hand-dexterity practice; 30 min/session, 5 sessions/week for 4 weeks.	2024 Adult Compendium of Physical Activities	Codes 09040 and 05090 (seated writing plus light object manipulation), weighted to 2.0 MET	Composite-activity crosswalk.	2.0
	Control	RT	TheraBand resistance exercise; 30 min/session, 5 sessions/week for 4 weeks.	2024 Adult Compendium of Physical Activities	Code 02054 (resistance training, multiple exercises, 8–15 reps, 3.5 MET)	Direct activity-code match.	3.5
Raciti 2022	Intervention	TAULHT	ArmeoSpring robot-assisted upper-limb therapy; approximately 45 min/session, 6 sessions/week for 8 weeks.	2024 Adult Compendium of Physical Activities	Code 22200 (active video game using upper body, light-to-moderate, 3.0 MET)	Closest-activity crosswalk.	3.0
	Control	CULR	Conventional physiotherapy comprising assisted movement, facilitation, proprioceptive work, reaching, and grasping; 6 sessions/week.	2024 Adult Compendium of Physical Activities	Codes 02115 and 02056 (light upper-body and general therapeutic exercise), composite ≈3.0 MET	Component-weighted activity crosswalk.	2.9

Study	Study arm	Exercise-type node	Reported intensity or prescription (condensed)	Authoritative tool	Activity entry	MET derivation	Assigned MET
Collett 2017	Intervention	MCT	Community exercise combining 30 min aerobic work at 55–85% age-predicted HRmax and 30 min resistance training; 2 sessions/week for 6 months.	2024 Adult Compendium of Physical Activities	30-min aerobic component (5.0 MET crosswalk) + Code 02054 resistance training (3.5 MET)	Time-weighted mean: [(5.0 × 30) + (3.5 × 30)]/60.	4.25
	Control	TOFDT	Home handwriting practice supported by a manual and monthly visits; physiological intensity not reported.	2024 Adult Compendium of Physical Activities	Codes 09040 and 05090 (seated writing plus light object manipulation), weighted to 2.0 MET	Composite-activity crosswalk.	2.0
van der Kolk 2019	Intervention	AET	Home-based stationary cycling prescribed by heart-rate reserve; 30–45 min/session, 3 sessions/week for 6 months.	ACSM metabolic/intensity framework; 2024 Adult Compendium	Code 01220 (stationary cycling, 90–100 W, moderate-to-vigorous, 6.0 MET) with HRR validation	Direct activity-code match.	6.0
	Control	CULR	Home stretching; 30–45 min/session, 3 sessions/week for 6 months.	ACSM metabolic/intensity framework; 2024 Adult Compendium	Code 02101 (mild stretching, 2.3 MET)	Direct activity-code match.	2.3
van der Kolk 2017	Intervention	AET	Home stationary cycling at 60–80% heart-rate reserve; approximately 30 min/session, 3 sessions/week for 24 weeks.	ACSM metabolic/intensity framework; 2024 Adult Compendium	Moderate stationary-cycling entries (HRR validated)	HRR-guided interpolation.	4.74
	Control	UC	No activity intervention; no structured exercise prescribed.	Trial protocol; prespecified zero-dose rule	No structured exercise/usual care (0 MET)	No structured exercise; zero-dose rule.	0.0
Çetin 2024	Intervention	TAULHT	Supervised exergame-based physiotherapy; 60 min/session, 3 sessions/week for 8 weeks.	2024 Adult Compendium of Physical Activities	Codes 22200 (3.0 MET, upper-body active video game) and 22240 (4.0 MET, total-body active video game)	Midpoint: (3.0 + 4.0)/2.	3.5
	Control	CULR	Dose-matched conventional physiotherapy exercise; 60 min/session, 3 sessions/week for 8 weeks.	2024 Adult Compendium of Physical Activities	Codes 02115 and 02056 (light upper-body and general therapeutic exercise), composite ≈3.0 MET	Closest-activity crosswalk.	3.0
Nackaerts 2017	Intervention	TOFDT	Intensive handwriting-amplitude practice; 30 min/day, 5 days/week for 6 weeks.	2024 Adult Compendium of Physical Activities	Code 09040 (sitting: writing, desk work, typing, 1.3 MET)	Direct activity-code match.	1.3
	Control	CULR	Time-matched stretching and relaxation; 30 min/day, 5 days/week for 6 weeks.	2024 Adult Compendium of Physical Activities	Code 02101 (mild stretching, 2.3 MET)	Direct activity-code match.	2.3
Kadkhodaie 2020	Intervention	RT	Upper-limb eccentric resistance training progressing from Borg RPE 10–11 to 12–14 (approximately 45–70% 1RM); 35–45 min, 3 days/week for 6 weeks.	ACSM metabolic/intensity framework; 2024 Adult Compendium	Code 02054 (multiple resistance exercises, 8–15 reps, 3.5 MET); Borg/%1RM used for validation	Direct activity-code match.	3.5
	Control	UC	Habitual physical activity maintained without additional training for 6 weeks.	Trial protocol; prespecified zero-dose rule	No structured exercise/usual care (0 MET)	No structured exercise; zero-dose rule.	0.0
Duncan 2012	Intervention	MBE	Community-based Argentine tango; 60 min/session, 2 sessions/week for 12 months; described as low-intensity aerobic activity.	2024 Adult Compendium of Physical Activities	Dancing crosswalk: Code 03040 (slow ballroom, 3.0 MET) and Code 03060 (moderate dance, 3.5 MET)	Closest-activity crosswalk.	3.5
	Control	UC	No-intervention control; usual lifestyle maintained.	Trial protocol; prespecified zero-dose rule	No structured exercise/usual care (0 MET)	No structured exercise; zero-dose rule.	0.0

Study	Study arm	Exercise-type node	Reported intensity or prescription (condensed)	Authoritative tool	Activity entry	MET derivation	Assigned MET
Robles-García 2016	Intervention	TAULHT	Virtual-reality action-observation training based on dominant-hand finger tapping; 25–35 min/session, 3 sessions/week for 4 weeks.	2024 Adult Compendium of Physical Activities	Code 22120 (video game, handheld controller, light effort, 1.5 MET)	Closest-activity crosswalk.	1.5
	Control	TOFDT	Active dominant-hand finger-tapping practice without action observation; 4-week programme.	2024 Adult Compendium of Physical Activities	Codes 09060/22120 (seated writing or light hand-controlled task, 1.5 MET)	Closest-activity crosswalk.	1.5
Marusiak 2019	Intervention	AET	Stationary-cycle interval training: 10-min warm-up, 40-min moderate intervals, and 10-min cool-down; 3 sessions/week for 8 weeks.	ACSM metabolic/intensity framework; 2024 Adult Compendium	Stationary-cycling entries for alternating interval phases	Interval-weighted mean: Σ(METfast × tfast + METslow × tslow)/Σt.	3.44
	Control	UC	Usual care/physiotherapy without moderate-to-vigorous aerobic training.	Trial protocol; prespecified zero-dose rule	No structured exercise/usual care (0 MET)	No structured exercise; zero-dose rule.	0.0
Schaeffer 2019	Intervention	TOFDT	Occupational therapy; 60 min/session, 1 session/week for 10 weeks.	2024 Adult Compendium of Physical Activities	Codes 09040 and 05090 (seated writing plus light object manipulation), weighted to 2.0 MET	Composite-activity crosswalk.	2.0
	Control	UC	No-intervention control; no structured exercise prescribed.	Trial protocol; prespecified zero-dose rule	No structured exercise/usual care (0 MET)	No structured exercise; zero-dose rule.	0.0
Demirgöz Demir 2025	Intervention	TAULHT	Conventional rehabilitation (90 min) plus Armeo Power robot-assisted upper-limb exercise (30 min); 5 days/week for 4 weeks.	2024 Adult Compendium of Physical Activities	90-min conventional rehabilitation (3.0 MET crosswalk) + 30-min robot-assisted upper-limb exercise (2.5 MET light guided exercise crosswalk)	Time-weighted mean: [(3.0 × 90) + (2.5 × 30)]/120.	2.88
	Control	CULR	Conventional rehabilitation with stretching, strengthening, activities of daily living, balance, coordination, and gait work; 90 min/day.	2024 Adult Compendium of Physical Activities	Codes 02115 and 02056 (light upper-body and general therapeutic exercise), composite ≈3.0 MET	Closest-activity crosswalk.	3.0
Heinzle 2025	Intervention	TAULHT	Immersive virtual-reality upper-limb training targeting range, coordination, selective finger movement, grasp, and daily tasks; 30 min, 4 times/week for 4 weeks.	2024 Adult Compendium of Physical Activities	Code 02115 (upper-body exercise, arm ergometer, light, 2.8 MET)	Closest-activity crosswalk.	2.8
	Control	CULR	Dose-matched conventional upper-limb mobility, strength, coordination, and functional-task training; 30 min, 4 times/week for 4 weeks.	2024 Adult Compendium of Physical Activities	Code 02115 (upper-body exercise, light, 2.8 MET)	Closest-activity crosswalk.	2.8
Raciti 2025	Intervention	TAULHT	AMADEO robot-assisted hand/finger exercise with feedback and virtual-reality tasks; 45 min/arm, 6 days/week for 8 weeks.	2024 Adult Compendium of Physical Activities	Codes 22160 and 02225 (light video-game or robot-assisted activity, 2.3 MET)	Closest-activity crosswalk.	2.3
	Control	CULR	Dose-matched conventional upper-limb physiotherapy with assisted movement, facilitation, reaching, and grasping; 45 min/arm, 6 days/week for 8 weeks.	2024 Adult Compendium of Physical Activities	Code 02101 (mild stretching, 2.3 MET)	Closest-activity crosswalk.	2.3

Study	Study arm	Exercise-type node	Reported intensity or prescription (condensed)	Authoritative tool	Activity entry	MET derivation	Assigned MET
Li 2024	Intervention	MBE	Baduanjin: 5-min warm-up, 30-min main practice, and 5-min cool-down; 40 min/session, 5 sessions/week for 4 weeks.	2024 Adult Compendium of Physical Activities	Code 15670 (tai chi/qigong, 3.3 MET) + Code 02101 (mild stretching, 2.3 MET)	Time-weighted mean: $[(3.3 \times 30) + (2.3 \times 10)]/40$.	3.0
	Control	UC	Habitual daily physical activity maintained; no structured exercise prescribed.	Trial protocol; prespecified zero-dose rule	No structured exercise/usual care (0 MET)	No structured exercise; zero-dose rule.	0.0
Caglar 2005	Intervention	MCT	Individually designed home exercise programme; approximately 60 min/session; component intensity not directly quantified.	2024 Adult Compendium of Physical Activities	Codes 02064, 02101 and/or 02054 according to component mix	Time-weighted mean: $\Sigma(MET_i \times t_i)/\Sigma t$.	3.8
	Control	UC	No prescribed home exercise programme.	Trial protocol; prespecified zero-dose rule	No structured exercise/usual care (0 MET)	No structured exercise; zero-dose rule.	0.0
Chen 2023	Intervention	TOFDT	Seated archery programme with approximately 15 min warm-up/stretching within a 2-h session; 1 session/week for 12 weeks.	2024 Adult Compendium of Physical Activities	Code 15010 (archery, 4.3 MET) + Code 02101 (stretching, 2.3 MET) + seated/light recovery crosswalk (1.5 MET)	Course-component mean: $\Sigma(MET_i \times t_i)/120$ min.	3.0
	Control	UC	Standard care with education on upper-limb/finger stretching; no structured exercise dose prescribed.	Trial protocol; prespecified zero-dose rule	No structured exercise/usual care (0 MET)	No structured exercise; zero-dose rule.	0.0
Eldemir 2023	Intervention	TOFDT	Videoconference-delivered upper-limb task circuit (15 stations, 3 min/task) plus home mobility training; Borg exertion was monitored.	ACSM metabolic/intensity framework; 2024 Adult Compendium	Code 02064 (home exercise, general, 3.8 MET) / Code 02022 (moderate calisthenics, 3.8 MET)	Closest-activity crosswalk.	3.8
	Control	MCT	Home balance, gait, and mobility exercises supported by a manual and telephone follow-up; Borg exertion was monitored.	ACSM metabolic/intensity framework; 2024 Adult Compendium	Code 02064 (home exercise, general, 3.8 MET) / Code 02022 (moderate calisthenics, 3.8 MET)	Closest-activity crosswalk.	3.8
Moraes 2024	Intervention	MCT	Global exercise combining 30 min aerobic work at 55–85% age-predicted HRmax and 30 min resistance training; 48 sessions over 24 weeks.	2024 Adult Compendium of Physical Activities	30-min aerobic component (5.0 MET crosswalk) + Code 02054 resistance training (3.5 MET)	Time-weighted mean: $[(5.0 \times 30) + (3.5 \times 30)]/60$; rounded.	4.3
	Control	TOFDT	Home handwriting training with hand warm-up and dominant-hand writing practice; 6-month programme.	2024 Adult Compendium of Physical Activities	Codes 09060/22120 (seated writing or light hand-controlled task, 1.5 MET)	Closest-activity crosswalk.	1.5

Supplementary Table 5.7B. Arm-level exercise prescriptions and MET assignments in studies reporting upper-limb function outcomes.

Study	Study arm	Exercise-type node	Reported intensity or prescription (condensed)	Authoritative tool	Activity entry	MET derivation	Assigned MET
Allen 2017	Intervention	TAULHT	Upper-limb exergaming controlled by finger or unilateral arm movements; 3 sessions/week for 12 weeks; MET not reported.	2024 Adult Compendium of Physical Activities	Codes 22200 (3.0 MET, upper-body active video game) and 22240 (4.0 MET, total-body active video game)	Midpoint: $(3.0 + 4.0)/2$.	3.5
	Control	UC	Usual care and normal activities; no structured exercise prescribed.	Trial protocol; prespecified zero-dose rule	No structured exercise/usual care (0 MET)	No structured exercise; zero-dose rule.	0.0

Study	Study arm	Exercise-type node	Reported intensity or prescription (condensed)	Authoritative tool	Activity entry	MET derivation	Assigned MET
Cabrera-Martos 2018	Intervention	TOFDT	Patient-specific, goal-oriented upper-limb functional training at home; 45 min/session, 2 sessions/week for 4 weeks.	2024 Adult Compendium of Physical Activities	Codes 02056 and 22200 (general therapeutic task or active upper-body game, 3.0 MET)	Closest-activity crosswalk.	3.0
	Control	CULR	Standardized home-based range-of-motion, dexterity, and stretching exercises; 45 min/session, 2 sessions/week for 4 weeks.	2024 Adult Compendium of Physical Activities	Codes 02115 and 02056 (light upper-body and general therapeutic exercise), composite ≈3.0 MET	Closest-activity crosswalk.	3.0
Choi 2022	Intervention	TOFDT	Conventional occupational therapy (30 min) plus task-oriented LSVT-BIG training (40 min); 5 sessions/week for 4 weeks.	2024 Adult Compendium of Physical Activities	Codes 02064 and 02022 (general home exercise or moderate calisthenics, 3.8 MET)	Composite-activity crosswalk; standardized per coding rule.	3.5
	Control	CULR	Conventional occupational therapy (30 min) plus relaxation and stretching (40 min); 5 sessions/week for 4 weeks.	2024 Adult Compendium of Physical Activities	Code 02101 (mild stretching, 2.3 MET)	Direct activity-code match.	2.3
Fernández-González 2019	Intervention	TAULHT	Leap Motion–based upper-limb serious games involving reaching, grasping, pinching, and forearm rotation; 30 min/session, 2 sessions/week for 6 weeks.	2024 Adult Compendium of Physical Activities	Code 22200 (active video game using upper body, light-to-moderate, 3.0 MET)	Direct activity-code match.	3.0
	Control	CULR	Conventional upper-limb physiotherapy with mobility, strengthening, stretching, and functional-task practice; session intensity not otherwise quantified.	2024 Adult Compendium of Physical Activities	Codes 02115 and 02056 (light upper-body and general therapeutic exercise), composite ≈3.0 MET	Closest-activity crosswalk.	3.0
Hashemi 2022	Intervention	TAULHT	Kinect-based upper-limb virtual-reality training; 24 sessions (3/week), with supervised and unsupervised protocols reported.	2024 Adult Compendium of Physical Activities	Code 22200 (active video game using upper body, light-to-moderate, 3.0 MET)	Direct activity-code match.	3.0
	Control	CULR	Conventional rehabilitation; 90 min/session, 3 sessions/week.	2024 Adult Compendium of Physical Activities	Codes 02115 and 02056 (light upper-body and general therapeutic exercise), composite ≈3.0 MET	Closest-activity crosswalk.	3.0
Mishra 2022	Intervention	TOFDT	Conventional upper-limb flexibility and strengthening plus motor-imagery training; 3 sessions/week for 3 weeks.	2024 Adult Compendium of Physical Activities	Codes 02056 and 22200 (general therapeutic task or active upper-body game, 3.0 MET)	Closest-activity crosswalk.	3.0
	Control	CULR	Conventional upper-limb flexibility, strengthening, and relaxation; 3 sessions/week for 3 weeks.	2024 Adult Compendium of Physical Activities	Codes 02115 and 02056 (light upper-body and general therapeutic exercise), composite ≈3.0 MET	Closest-activity crosswalk.	3.0
Taghizadeh 2017	Intervention	TOFDT	Sensory–motor hand training plus usual rehabilitation; 10 sessions (5/week) over 2 weeks.	2024 Adult Compendium of Physical Activities	Codes 02056 and 22200 (general therapeutic task or active upper-body game, 3.0 MET)	Closest-activity crosswalk.	3.0
	Control	UC	Usual rehabilitation without a separately prescribed structured exercise dose.	Trial protocol; prespecified zero-dose rule	No structured exercise/usual care (0 MET)	No structured exercise; zero-dose rule.	0.0

Study	Study arm	Exercise-type node	Reported intensity or prescription (condensed)	Authoritative tool	Activity entry	MET derivation	Assigned MET
Zare 2022	Intervention	TOFDT	Activity-based functional training for 6 weeks; session intensity not otherwise quantified.	2024 Adult Compendium of Physical Activities	Codes 02056 and 22200 (general therapeutic task or active upper-body game, 3.0 MET)	Closest-activity crosswalk.	3.0
	Control	RT	Progressive strengthening using a power web and elastic resistance; approximately 90 min/day for 6 weeks.	2024 Adult Compendium of Physical Activities	Code 02054 (resistance training, multiple exercises, 8–15 reps, 3.5 MET)	Direct activity-code match.	3.5
Vanbellingen 2017	Intervention	TOFDT	High-intensity, task-specific home hand-dexterity practice; 30 min/session, 5 sessions/week for 4 weeks.	2024 Adult Compendium of Physical Activities	Codes 09040 and 05090 (seated writing plus light object manipulation), weighted to 2.0 MET	Composite-activity crosswalk.	2.0
	Control	RT	TheraBand resistance exercise; 30 min/session, 5 sessions/week for 4 weeks.	2024 Adult Compendium of Physical Activities	Code 02054 (resistance training, multiple exercises, 8–15 reps, 3.5 MET)	Direct activity-code match.	3.5
Raciti 2022	Intervention	TAULHT	ArmeoSpring robot-assisted upper-limb therapy; approximately 45 min/session, 6 sessions/week for 8 weeks.	2024 Adult Compendium of Physical Activities	Code 22200 (active video game using upper body, light-to-moderate, 3.0 MET)	Closest-activity crosswalk.	3.0
	Control	CULR	Conventional physiotherapy comprising assisted movement, facilitation, proprioceptive work, reaching, and grasping; 6 sessions/week.	2024 Adult Compendium of Physical Activities	Codes 02115 and 02056 (light upper-body and general therapeutic exercise), composite ≈3.0 MET	Component-weighted activity crosswalk.	2.9
Çetin 2024	Intervention	TAULHT	Supervised exergame-based physiotherapy; 60 min/session, 3 sessions/week for 8 weeks.	2024 Adult Compendium of Physical Activities	Codes 22200 (3.0 MET, upper-body active video game) and 22240 (4.0 MET, total-body active video game)	Midpoint: (3.0 + 4.0)/2.	3.5
	Control	CULR	Dose-matched conventional physiotherapy exercise; 60 min/session, 3 sessions/week for 8 weeks.	2024 Adult Compendium of Physical Activities	Codes 02115 and 02056 (light upper-body and general therapeutic exercise), composite ≈3.0 MET	Closest-activity crosswalk.	3.0
Lorek 2025	Intervention	AET	Moderate-intensity aerobic interval cycling with alternating cadence phases; heart rate, cadence, power, and perceived exertion were monitored for 12 weeks.	ACSM metabolic/intensity framework; 2024 Adult Compendium	Stationary-cycling entries for alternating interval phases	Interval-weighted mean: $\Sigma(MET_{fast} \times t_{fast} + MET_{slow} \times t_{slow})/\Sigma t$.	3.3
	Control	UC	Non-training control; no structured exercise prescribed.	Trial protocol; prespecified zero-dose rule	No structured exercise/usual care (0 MET)	No structured exercise; zero-dose rule.	0.0
Marusiak 2019	Intervention	AET	Stationary-cycle interval training: 10-min warm-up, 40-min moderate intervals, and 10-min cool-down; 3 sessions/week for 8 weeks.	ACSM metabolic/intensity framework; 2024 Adult Compendium	Stationary-cycling entries for alternating interval phases	Interval-weighted mean: $\Sigma(MET_{fast} \times t_{fast} + MET_{slow} \times t_{slow})/\Sigma t$.	3.44
	Control	UC	Usual care/physiotherapy without moderate-to-vigorous aerobic training.	Trial protocol; prespecified zero-dose rule	No structured exercise/usual care (0 MET)	No structured exercise; zero-dose rule.	0.0
Schaeffer 2019	Intervention	TOFDT	Occupational therapy; 60 min/session, 1 session/week for 10 weeks.	2024 Adult Compendium of Physical Activities	Codes 09040 and 05090 (seated writing plus light object manipulation), weighted to 2.0 MET	Composite-activity crosswalk.	2.0
	Control	UC	No-intervention control; no structured exercise prescribed.	Trial protocol; prespecified zero-dose rule	No structured exercise/usual care (0 MET)	No structured exercise; zero-dose rule.	0.0

Study	Study arm	Exercise-type node	Reported intensity or prescription (condensed)	Authoritative tool	Activity entry	MET derivation	Assigned MET
Demirgöz Demir 2025	Intervention	TAULHT	Conventional rehabilitation (90 min) plus Armeo Power robot-assisted upper-limb exercise (30 min); 5 days/week for 4 weeks.	2024 Adult Compendium of Physical Activities	90-min conventional rehabilitation (3.0 MET crosswalk) + 30-min robot-assisted upper-limb exercise (2.5 MET light guided exercise crosswalk)	Time-weighted mean: $[(3.0 \times 90) + (2.5 \times 30)]/120$.	2.88
	Control	CULR	Conventional rehabilitation with stretching, strengthening, activities of daily living, balance, coordination, and gait work; 90 min/day.	2024 Adult Compendium of Physical Activities	Codes 02115 and 02056 (light upper-body and general therapeutic exercise), composite ≈3.0 MET	Closest-activity crosswalk.	3.0
Heinzle 2025	Intervention	TAULHT	Immersive virtual-reality upper-limb training targeting range, coordination, selective finger movement, grasp, and daily tasks; 30 min, 4 times/week for 4 weeks.	2024 Adult Compendium of Physical Activities	Code 02115 (upper-body exercise, arm ergometer, light, 2.8 MET)	Closest-activity crosswalk.	2.8
	Control	CULR	Dose-matched conventional upper-limb mobility, strength, coordination, and functional-task training; 30 min, 4 times/week for 4 weeks.	2024 Adult Compendium of Physical Activities	Code 02115 (upper-body exercise, light, 2.8 MET)	Closest-activity crosswalk.	2.8
Raciti 2025	Intervention	TAULHT	AMADEO robot-assisted hand/finger exercise with feedback and virtual-reality tasks; 45 min/arm, 6 days/week for 8 weeks.	2024 Adult Compendium of Physical Activities	Codes 22160 and 02225 (light video-game or robot-assisted activity, 2.3 MET)	Closest-activity crosswalk.	2.3
	Control	CULR	Dose-matched conventional upper-limb physiotherapy with assisted movement, facilitation, reaching, and grasping; 45 min/arm, 6 days/week for 8 weeks.	2024 Adult Compendium of Physical Activities	Code 02101 (mild stretching, 2.3 MET)	Closest-activity crosswalk.	2.3
Pazzaglia 2020	Intervention	TAULHT	Virtual-reality rehabilitation; 40 min/session, 3 sessions/week for 6 weeks.	2024 Adult Compendium of Physical Activities	Code 22200 (active video game using upper body, light-to-moderate, 3.0 MET)	Direct activity-code match.	3.0
	Control	MCT	Conventional multicomponent rehabilitation; 40 min/session, 3 sessions/week for 6 weeks.	2024 Adult Compendium of Physical Activities	Codes 02064, 02101 and/or 02054 according to component mix	Time-weighted mean: $\Sigma(MET_i \times t_i)/\Sigma t$.	3.0
Moraes 2024	Intervention	MCT	Global exercise combining 30 min aerobic work at 55–85% age-predicted HRmax and 30 min resistance training; 48 sessions over 24 weeks.	2024 Adult Compendium of Physical Activities	30-min aerobic component (5.0 MET crosswalk) + Code 02054 resistance training (3.5 MET)	Time-weighted mean: $[(5.0 \times 30) + (3.5 \times 30)]/60$; rounded.	4.3
	Control	TOFDT	Home handwriting training with hand warm-up and dominant-hand writing practice; 6-month programme.	2024 Adult Compendium of Physical Activities	Codes 09060/22120 (seated writing or light hand-controlled task, 1.5 MET)	Closest-activity crosswalk.	1.5
Eldemir 2023	Intervention	TOFDT	Videoconference-delivered upper-limb task circuit (15 stations, 3 min/task) plus home mobility training; Borg exertion was monitored.	ACSM metabolic/intensity framework; 2024 Adult Compendium	Code 02064 (home exercise, general, 3.8 MET) / Code 02022 (moderate calisthenics, 3.8 MET)	Closest-activity crosswalk.	3.8
	Control	MCT	Home balance, gait, and mobility exercises supported by a manual and telephone follow-up; Borg exertion was monitored.	ACSM metabolic/intensity framework; 2024 Adult Compendium	Code 02064 (home exercise, general, 3.8 MET) / Code 02022 (moderate calisthenics, 3.8 MET)	Closest-activity crosswalk.	3.8

Study	Study arm	Exercise-type node	Reported intensity or prescription (condensed)	Authoritative tool	Activity entry	MET derivation	Assigned MET
Leal 2019	Intervention	RT	Supervised multiexercise resistance training (2 sets of 8–12 repetitions); 30–40 min/session, 2 sessions/week for 24 weeks.	ACSM metabolic/intensity framework; 2024 Adult Compendium	Code 02054 (resistance training, multiple exercises, 8–15 reps, 3.5 MET)	Direct activity-code match.	3.5
	Control	UC	Usual medication only; no resistance training prescribed.	Trial protocol; prespecified zero-dose rule	No structured exercise/usual care (0 MET)	No structured exercise; zero-dose rule.	0.0
Zhang 2026	Intervention	MBE	Tai Chi programme comprising a 45-min main component and 15-min warm-up/cool-down; 14-week intervention.	2024 Adult Compendium of Physical Activities	Code 15670 (tai chi/qigong, 3.3 MET) + Code 02101 (mild stretching/warm-up, 2.3 MET)	Time-weighted mean: [(3.3 × 45) + (2.3 × 15)]/60.	3.0
	Control	UC	Control condition; no structured exercise prescription identified for the dose-coded comparator.	Trial protocol; prespecified zero-dose rule	No structured exercise/usual care (0 MET)	No structured exercise; zero-dose rule.	0.0
Martinazzo 2025	Intervention	MBE	Progressive mat Pilates; 60 min/session for 12 weeks.	2024 Adult Compendium of Physical Activities	Code 02105 (Pilates, general, 2.8 MET)	Compendium value standardized per coding rule.	3.0
	Control	UC	Control condition; no structured exercise prescription identified for the dose-coded comparator.	Trial protocol; prespecified zero-dose rule	No structured exercise/usual care (0 MET)	No structured exercise; zero-dose rule.	0.0

Note for Supplementary Tables 5.7A and 5.7B: Each row represents one study arm; blank cells in the "Study" column indicate additional arms of the study named immediately above. Because eligibility was outcome specific, the same study arm may appear in both tables and should not be counted as a separate trial. "Reported intensity or prescription (condensed)" summarises the intervention information used for MET assignment; "Authoritative tool" and "Activity entry" identify the source and corresponding activity code; and "MET derivation" records direct matching, crosswalking, interpolation, or time weighting according to Supplementary Table 5.2. Assigned MET is the final value used to calculate exact weekly dose in Supplementary Tables 5.6A and 5.6B. For time-weighted calculations, MET_i and t_i denote the MET value and duration of component i, respectively; "fast" and "slow" indicate the corresponding interval phases.

Abbreviations: 1RM, one-repetition maximum; ACSM, American College of Sports Medicine; AET, aerobic exercise training; CULR, conventional upper-limb rehabilitation; HRmax, maximum heart rate; HRR, heart-rate reserve; LSVT BIG, Lee Silverman Voice Treatment BIG; MBE, mind–body exercise; MCT, multicomponent training; MET, metabolic equivalent of task; RPE, rating of perceived exertion; RT, resistance training; TAULHT, technology-assisted upper-limb or hand training; TOFDT, task-oriented finger dexterity training; UC, usual care or no structured exercise.

Supplementary File 6: Risk-of-bias assessment

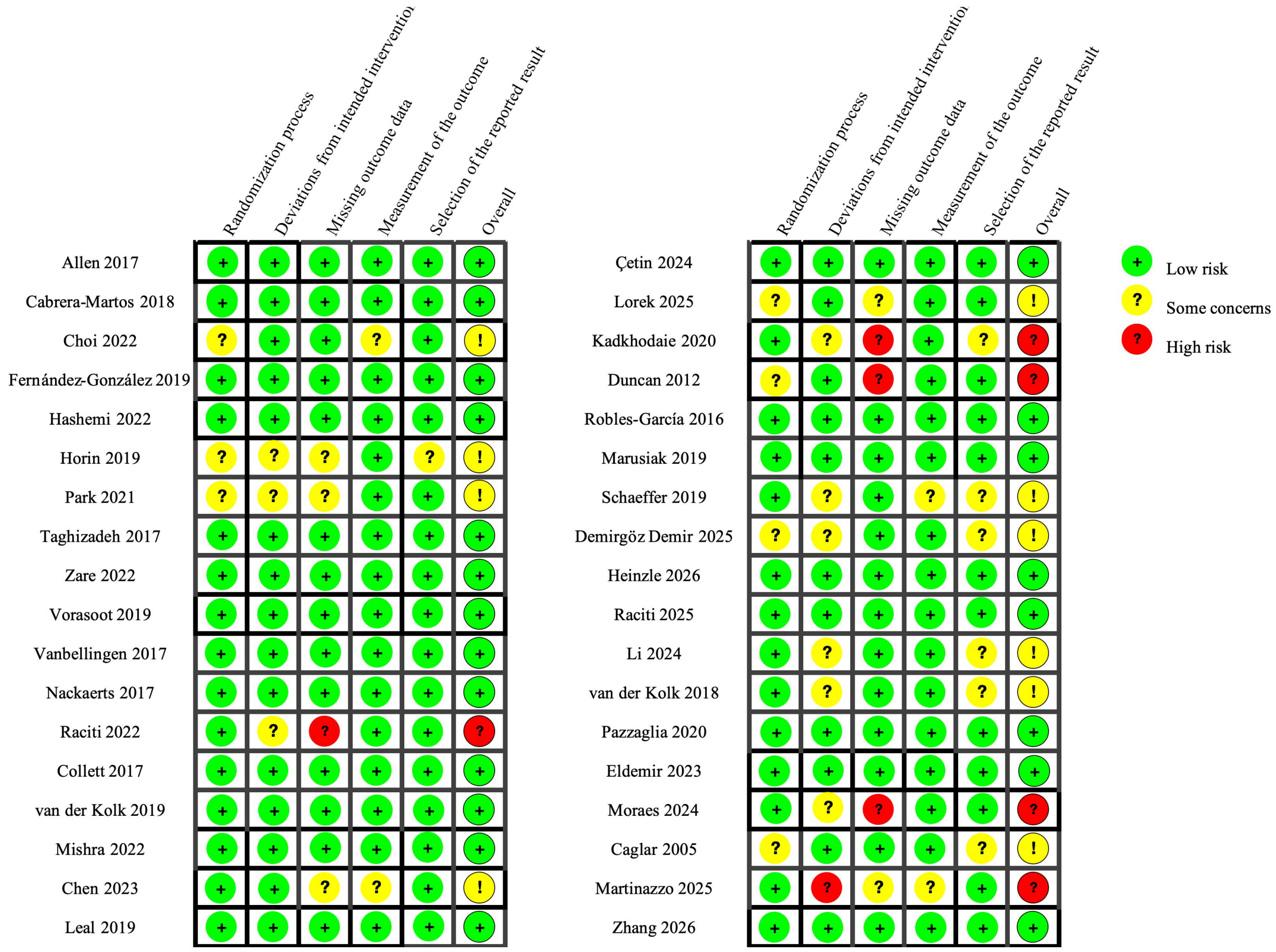

Supplementary Fig. 6A. RoB 2 quality assessment of studies.

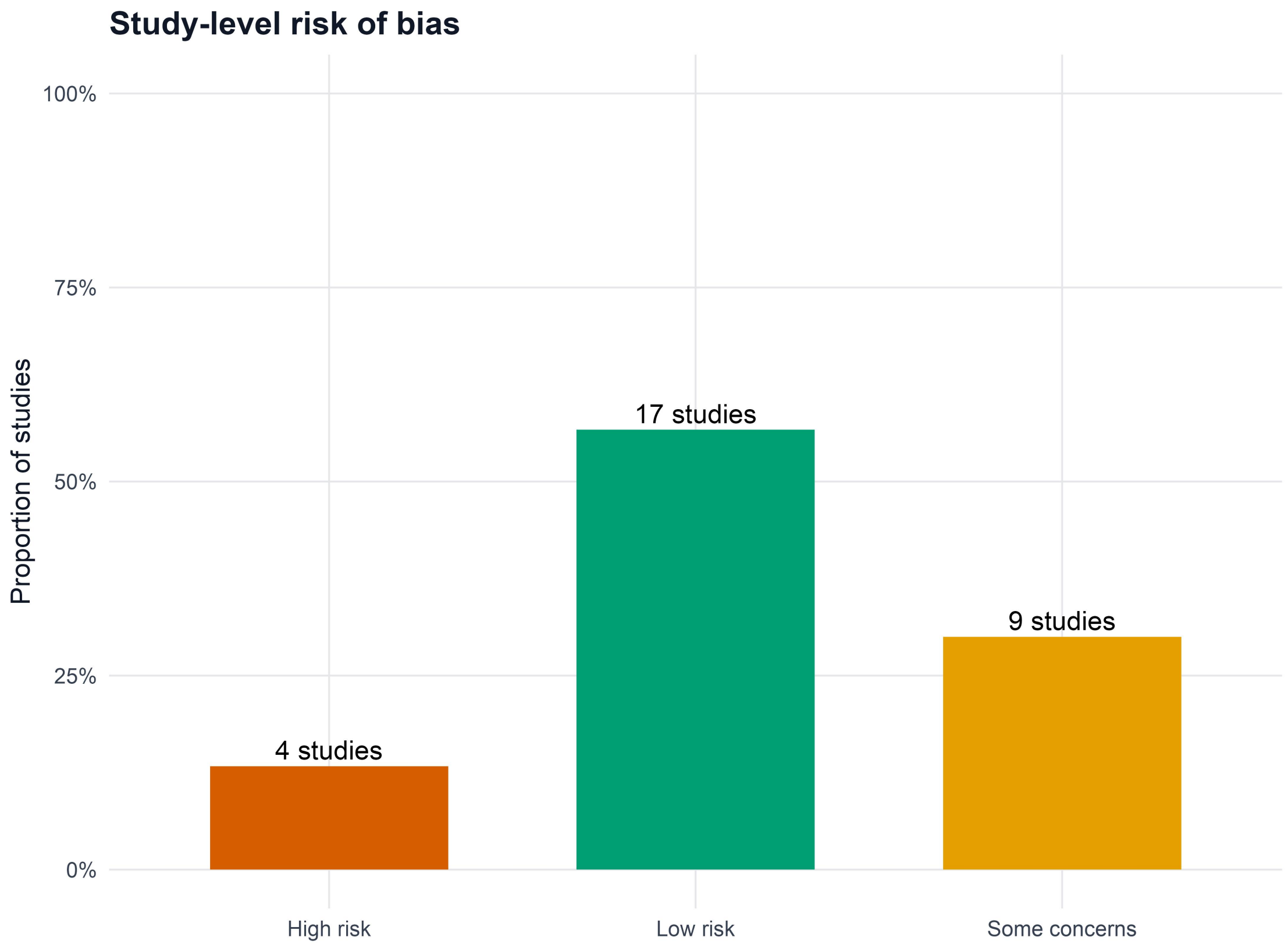

Supplementary Fig. 6B. Distribution of overall risk-of-bias judgements across studies reporting finger dexterity outcomes.

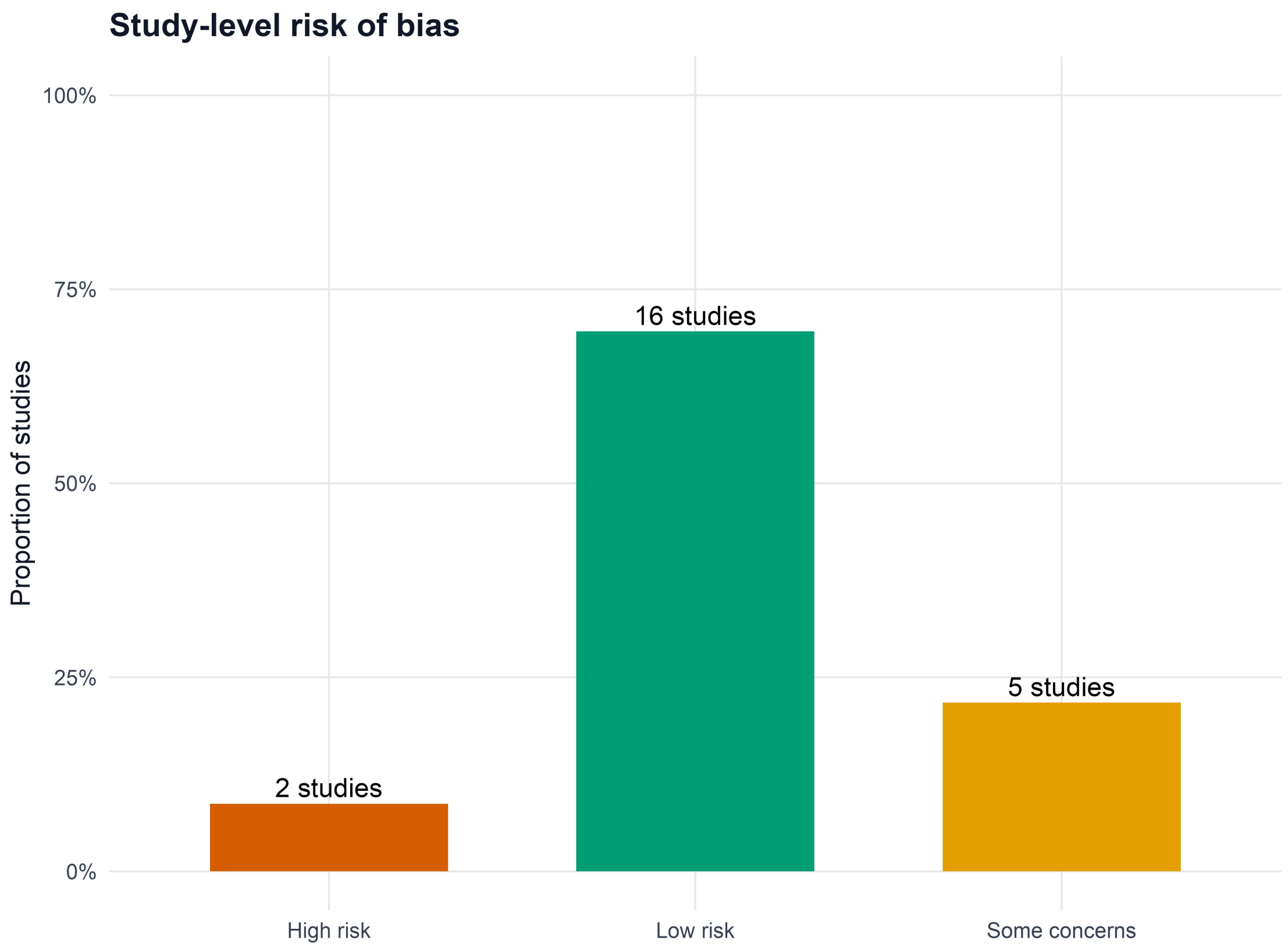

Supplementary Fig. 6C. Distribution of overall risk-of-bias judgements across studies reporting upper-limb function outcomes.

Supplementary File 7: Conventional Bayesian network meta-analysis

S7.1 Relative effects versus usual care and posterior intervention rankings

Supplementary Table 7.1A. Relative effects versus usual care and posterior intervention rankings for finger dexterity.

Exercise modality	Posterior median Hedges' g versus UC	95% CrI	P(g > 0)	SUCRA	Mean rank	Rank
AET	1.460	0.260–2.685	0.991	0.876	1.87	1
CULR	0.478	-0.468–1.438	0.845	0.380	5.34	7
MBE	0.601	-0.790–2.000	0.809	0.477	4.66	5
MCT	0.536	-0.603–1.669	0.831	0.431	4.98	6
RT	0.656	-0.605–1.912	0.852	0.501	4.49	4
TAULHT	0.657	-0.290–1.607	0.916	0.513	4.41	3
TOFDT	0.956	0.233–1.671	0.994	0.713	3.01	2
UC	0.000	0.000–0.000	—	0.109	7.24	8

Note: Effects are reported as posterior median Hedges' g values with 95% credible intervals (CrIs) for each exercise modality versus usual care (UC); positive values favour the exercise modality. P(g > 0) denotes the posterior probability of benefit relative to UC and is not applicable to UC itself. Rankings include all interventions in the network. Higher SUCRA values and lower mean ranks indicate more favourable rankings; rank 1 denotes the highest-ranked intervention. AET, aerobic exercise; CULR, conventional upper-limb rehabilitation; MBE, mind–body exercise; MCT, multicomponent training; RT, resistance training; SUCRA, surface under the cumulative ranking curve; TAULHT, technology-assisted upper-limb or hand training; TOFDT, task-oriented finger dexterity training.

Supplementary Table 7.1B. Relative effects versus usual care and posterior intervention rankings for upper-limb function.

Exercise modality	Posterior median Hedges' g versus UC	95% CrI	P(g > 0)	SUCRA	Mean rank	Rank
AET	0.963	-1.593–3.513	0.769	0.551	4.14	4
CULR	-0.252	-2.614–2.106	0.419	0.228	6.41	8
MBE	6.897	4.284–9.426	>0.999	1.000	1	1
MCT	0.185	-2.620–2.972	0.553	0.372	5.39	6
RT	1.035	-1.383–3.479	0.799	0.590	3.87	2
TAULHT	0.169	-2.178–2.521	0.559	0.376	5.37	5
TOFDT	0.854	-1.066–2.812	0.809	0.584	3.91	3
UC	0.000	0.000–0.000	—	0.299	5.91	7

Note: Effects are reported as posterior median Hedges' g values with 95% credible intervals (CrIs) for each exercise modality versus usual care (UC); positive values favour the exercise modality. P(g > 0) denotes the posterior probability of benefit relative to UC and is not applicable to UC itself. Rankings include all interventions in the network. Higher SUCRA values and lower mean ranks indicate more favourable rankings; rank 1 denotes the highest-ranked intervention. AET, aerobic exercise; CULR, conventional upper-limb rehabilitation; MBE, mind–body exercise; MCT, multicomponent training; RT,

resistance training; SUCRA, surface under the cumulative ranking curve; TAULHT, technology-assisted upper-limb or hand training; TOFDT, task-oriented finger dexterity training.

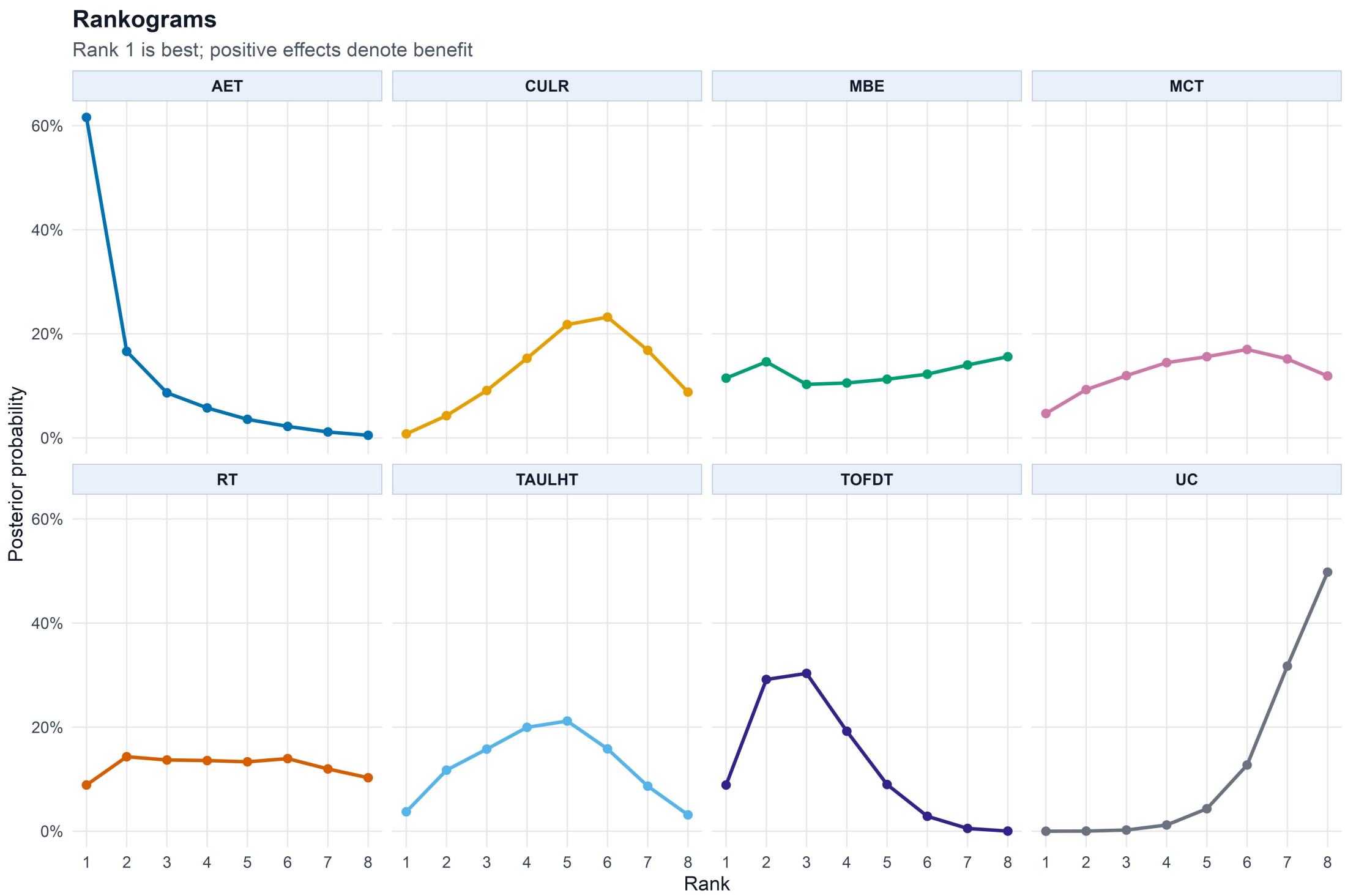

Supplementary Fig. 7.1A. Posterior rank distributions of exercise modalities for finger dexterity in the conventional Bayesian network meta-analysis.

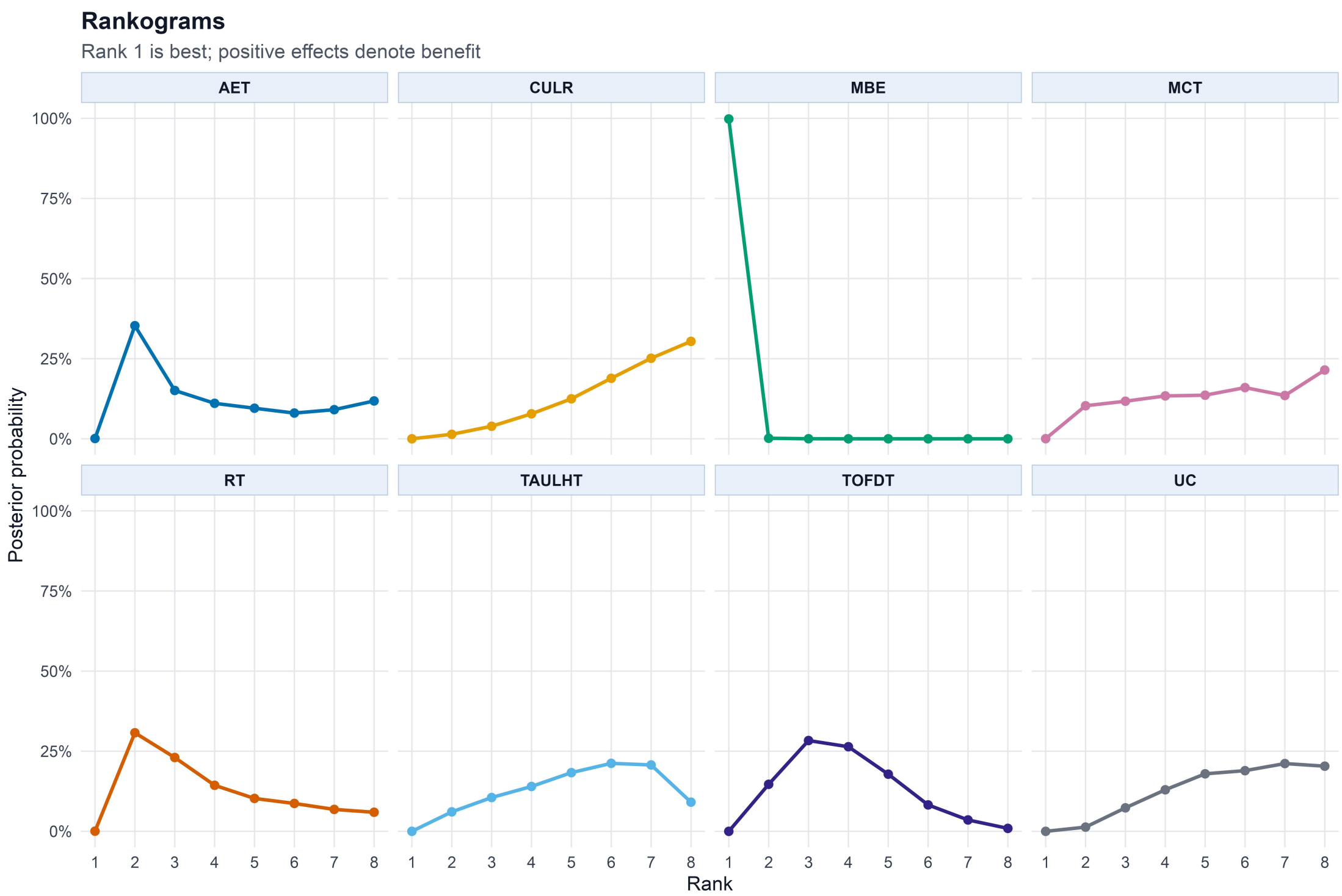

Supplementary Fig. 7.1B. Posterior rank distributions of exercise modalities for upper-limb function in the conventional Bayesian network meta-analysis.

S7.2 CINeMA assessment of certainty of evidence for pairwise intervention comparisons

Supplementary Table 7.2A. Certainty of evidence for pairwise intervention comparisons for finger dexterity.

Comparison	Number of studies	Within-study bias	Reporting bias	Indirectness	Imprecision	Heterogeneity	Incoherence	Confidence rating
CULR vs AET	1	No concerns	No concerns	Some concerns	Major concerns	Major concerns	No concerns	Very low
MBE vs AET	0	Some concerns	No concerns	No concerns	No concerns	Major concerns	Some concerns	Very low
MCT vs AET	0	Some concerns	No concerns	Some concerns	Some concerns	No concerns	No concerns	Very low
RT vs AET	0	No concerns	No concerns	Some concerns	No concerns	Some concerns	No concerns	Low
TAULHT vs AET	0	No concerns	No concerns	No concerns	Some concerns	No concerns	No concerns	Moderate
TOFDT vs AET	0	No concerns	No concerns	No concerns	No concerns	No concerns	Some concerns	Moderate
UC vs AET	2	No concerns	No concerns	No concerns	No concerns	No concerns	No concerns	High
MBE vs CULR	0	Some concerns	No concerns	No concerns	Some concerns	Some concerns	No concerns	Very low
MCT vs CULR	0	Some concerns	No concerns	No concerns	No concerns	No concerns	Some concerns	Low
RT vs CULR	0	No concerns	No concerns	Some concerns	Major concerns	No concerns	No concerns	Very low
TAULHT vs CULR	7	No concerns	No concerns	Some concerns	No concerns	Some concerns	No concerns	Low
TOFDT vs CULR	3	No concerns	No concerns	Some concerns	No concerns	Some concerns	No concerns	Low
UC vs CULR	0	No concerns	No concerns	No concerns	No concerns	Some concerns	Some concerns	Low
MCT vs MBE	0	No concerns	No concerns	No concerns	Major concerns	Some concerns	No concerns	Very low
RT vs MBE	0	Some concerns	No concerns	No concerns	No concerns	No concerns	Some concerns	Low
TAULHT vs MBE	0	No concerns	No concerns	No concerns	No concerns	No concerns	Some concerns	Moderate
TOFDT vs MBE	0	No concerns	No concerns	No concerns	Some concerns	No concerns	Some concerns	Low
UC vs MBE	2	No concerns	No concerns	Some concerns	No concerns	No concerns	No concerns	Moderate
RT vs MCT	0	Some concerns	No concerns	Major concerns	Some concerns	Some concerns	No concerns	Very low
TAULHT vs MCT	0	Some concerns	No concerns	No concerns	Some concerns	No concerns	No concerns	Low
TOFDT vs MCT	3	Some concerns	No concerns	No concerns	Some concerns	No concerns	No concerns	Low
UC vs MCT	1	Some concerns	No concerns	No concerns	Some concerns	No concerns	No concerns	Low
TAULHT vs RT	0	No concerns	No concerns	No concerns	No concerns	Some concerns	Some concerns	Low

Comparison	Number of studies	Within-study bias	Reporting bias	Indirectness	Imprecision	Heterogeneity	Incoherence	Confidence rating
TOFDT vs RT	2	No concerns	No concerns	No concerns	No concerns	No concerns	Some concerns	Moderate
UC vs RT	1	Some concerns	No concerns	No concerns	No concerns	Some concerns	No concerns	Low
TOFDT vs TAULHT	1	No concerns	No concerns	Some concerns	No concerns	Some concerns	No concerns	Low
UC vs TAULHT	2	No concerns	No concerns	Some concerns	No concerns	No concerns	No concerns	Moderate
UC vs TOFDT	5	No concerns	No concerns	No concerns	No concerns	No concerns	No concerns	High

Supplementary Table 7.2B. Certainty of evidence for pairwise intervention comparisons for upper-limb function.

Comparison	Number of studies	Within-study bias	Reporting bias	Indirectness	Imprecision	Heterogeneity	Incoherence	Confidence rating
CULR vs AET	0	No concerns	No concerns	Major concerns	No concerns	No concerns	No concerns	Low
MBE vs AET	0	Some concerns	No concerns	Some concerns	No concerns	No concerns	Some concerns	Very low
MCT vs AET	0	Some concerns	No concerns	No concerns	No concerns	Some concerns	No concerns	Low
RT vs AET	0	Some concerns	No concerns	No concerns	No concerns	Some concerns	No concerns	Low
TAULHT vs AET	0	No concerns	No concerns	Some concerns	Some concerns	No concerns	No concerns	Low
TOFDT vs AET	0	No concerns	No concerns	No concerns	No concerns	Some concerns	Some concerns	Low
UC vs AET	2	No concerns	No concerns	Some concerns	No concerns	No concerns	Some concerns	Low
MBE vs CULR	0	No concerns	No concerns	Major concerns	No concerns	No concerns	Some concerns	Very low
MCT vs CULR	0	Some concerns	No concerns	No concerns	Some concerns	No concerns	No concerns	Low
RT vs CULR	0	No concerns	No concerns	No concerns	No concerns	Some concerns	Some concerns	Low
TAULHT vs CULR	7	No concerns	No concerns	No concerns	Some concerns	Some concerns	No concerns	Low
TOFDT vs CULR	3	No concerns	No concerns	No concerns	Some concerns	Some concerns	No concerns	Low
UC vs CULR	0	No concerns	No concerns	Some concerns	Some concerns	Some concerns	No concerns	Very low
MCT vs MBE	0	No concerns	No concerns	No concerns	Some concerns	No concerns	Some concerns	Low
RT vs MBE	0	Some concerns	No concerns	No concerns	No concerns	No concerns	No concerns	Moderate
TAULHT vs MBE	0	No concerns	No concerns	No concerns	No concerns	No concerns	Some concerns	Moderate
TOFDT vs MBE	0	No concerns	No concerns	No concerns	No concerns	No concerns	No concerns	High
UC vs MBE	2	No concerns	No concerns	No concerns	Some concerns	Some concerns	No concerns	Low

Comparison	Number of studies	Within-study bias	Reporting bias	Indirectness	Imprecision	Heterogeneity	Incoherence	Confidence rating
RT vs MCT	0	Some concerns	No concerns	No concerns	Some concerns	No concerns	Some concerns	Very low
TAULHT vs MCT	1	No concerns	No concerns	Some concerns	No concerns	Some concerns	No concerns	Low
TOFDT vs MCT	2	Major concerns	No concerns	No concerns	Some concerns	Major concerns	No concerns	Very low
UC vs MCT	0	Some concerns	No concerns	Major concerns	No concerns	No concerns	Some concerns	Very low
TAULHT vs RT	0	Some concerns	No concerns	Some concerns	Major concerns	No concerns	No concerns	Very low
TOFDT vs RT	2	No concerns	No concerns	Some concerns	Some concerns	No concerns	No concerns	Low
UC vs RT	1	No concerns	No concerns	Some concerns	No concerns	Some concerns	No concerns	Low
TOFDT vs TAULHT	0	No concerns	No concerns	Some concerns	No concerns	No concerns	Some concerns	Low
UC vs TAULHT	1	No concerns	No concerns	No concerns	Some concerns	Some concerns	No concerns	Low
UC vs TOFDT	2	Some concerns	No concerns	No concerns	No concerns	No concerns	No concerns	Moderate

Note: Certainty of evidence was assessed using CINeMA across the six domains shown and rated as high, moderate, low, or very low. “Number of studies” denotes direct head-to-head studies; zero indicates that the comparison was informed exclusively by indirect evidence. CINeMA, Confidence in Network Meta-Analysis; AET, aerobic exercise; CULR, conventional upper-limb rehabilitation; MBE, mind–body exercise; MCT, multicomponent training; RT, resistance training; TAULHT, technology-assisted upper-limb or hand training; TOFDT, task-oriented finger dexterity training; UC, usual care or no structured exercise.

S7.3 Posterior between-study heterogeneity

Supplementary Table 7.3. Posterior estimates of between-study heterogeneity (τ) for finger dexterity and upper-limb function.

Outcome	Between-study heterogeneity, posterior median τ	95% CrI
Finger dexterity	0.931	0.673–1.341
Upper-limb function	1.901	1.620–1.996

Note: Note: CrI, credible interval. τ denotes the posterior median between-study standard deviation estimated under the random-effects consistency model and is expressed on the Hedges' g (standardized mean difference) scale. The 95% CrI reflects posterior uncertainty around τ; larger τ values indicate greater between-study heterogeneity.

S7.4 Global inconsistency, model-fit, and convergence assessment

Supplementary Table 7.4A. Assessment of global inconsistency in conventional Bayesian network meta-analyses.

Outcome	Model	DIC	Residual deviance	Effective parameters (pD)	ΔDIC versus consistency model	Convergence status
Finger dexterity	Consistency random-effects model	117.603	60.304	57.299	0.000	Converged
	UME random-effects model	118.354	60.255	58.099	0.752	Converged
Upper-limb function	Consistency random-effects model	90.196	46.774	45.422	0.000	Converged
	UME random-effects model	92.339	46.822	45.516	0.143	Converged

Note: Δ DIC was calculated as $DIC_{UME} - DIC_{consistency}$; negative values indicate improved fit of the UME model. All models achieved satisfactory convergence. The UME model did not materially improve model fit for either outcome, providing no clear indication of global inconsistency; however, this should not be interpreted as proof of consistency. DIC, deviance information criterion; pD, effective number of parameters; UME, unrelated mean effects.

Supplementary Table 7.4B. Model comparison and convergence assessment for conventional Bayesian network meta-analyses of finger dexterity and upper-limb function.

Outcome	Model	DIC	Residual deviance	τ (95% CrI)	Maximum PSRF	Minimum ESS	Convergence	Primary model
Finger dexterity	Consistency model, random effects	117.60	60.30	0.93 (0.67–1.34)	1.0002	21,759.1	Converged	Yes
	Consistency model, fixed effect	256.09	218.97	—	1.0003	15,935.2	Converged	No
	Unrelated mean effects model, random effects	118.35	60.26	0.96 (0.67–1.45)	1.0001	28,791.8	Converged	No
Upper-limb function	Consistency model, random effects	92.20	46.77	1.90 (1.62–2.00)	1.0001	34,588.0	Converged	Yes
	Consistency model, fixed effect	762.48	732.32	—	1.0009	20,314.3	Converged	No
	Unrelated mean effects model, random effects	92.34	46.82	1.91 (1.65–2.00)	1.0001	35,019.6	Converged	No

Abbreviations: CrI, credible interval; DIC, deviance information criterion; ESS, effective sample size; PSRF, potential scale reduction factor. τ denotes the posterior between-study standard deviation. The random-effects consistency model was prespecified as the primary model. Lower DIC values indicate better relative model fit. A dash indicates that the parameter was not applicable to the fixed-effect model. All models achieved convergence.

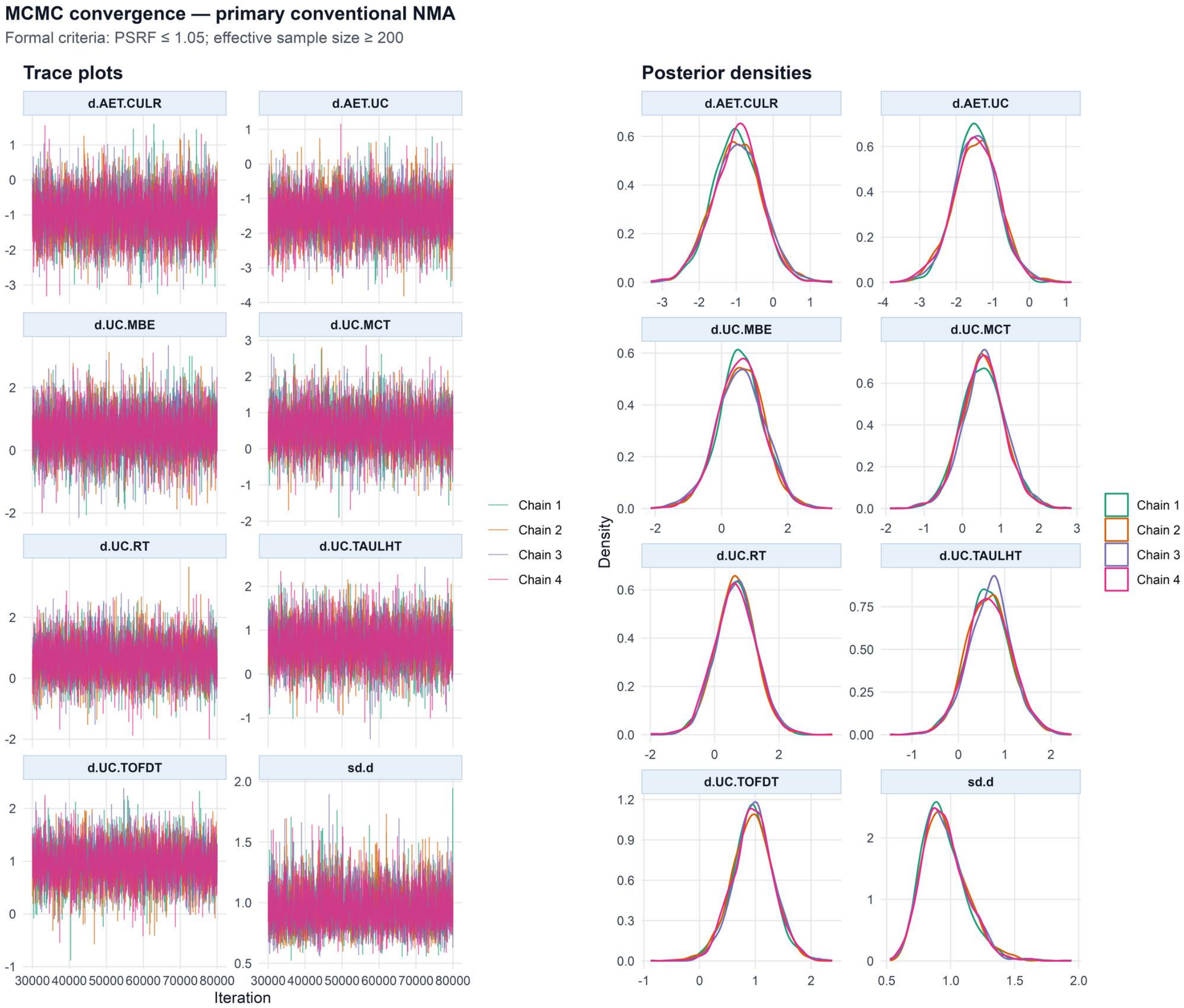

Supplementary Fig. 7.4A. Markov chain Monte Carlo convergence diagnostics for the conventional Bayesian network meta-analysis of finger dexterity.

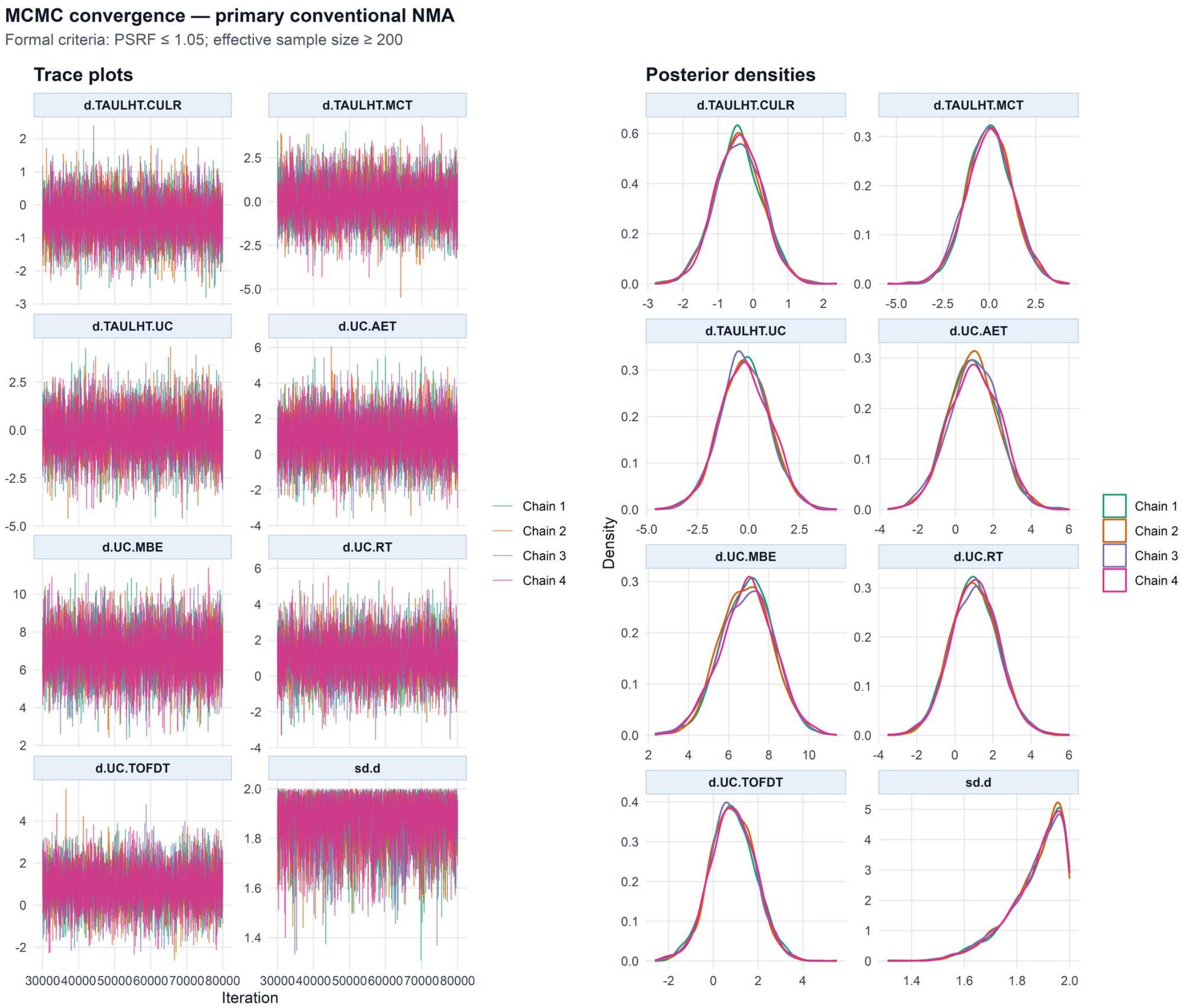

Supplementary Fig. 7.4B. Markov chain Monte Carlo convergence diagnostics for the conventional Bayesian network meta-analysis of upper-limb function.

S7.5 Local inconsistency assessed using node splitting

Supplementary Table 7.5A. Node-splitting estimates and local inconsistency tests for finger dexterity.

Comparison	Direct estimate (95% CrI)	Indirect estimate (95% CrI)	Consistency-model estimate (95% CrI)	P for inconsistency
CULR vs AET	−0.062 (−1.905–1.800)	−1.802 (−3.554–−0.056)	−0.979 (−2.288–0.316)	0.171
UC vs AET	−2.000 (−3.410–−0.576)	−0.257 (−2.399–1.883)	−1.457 (−2.665–−0.262)	0.175
TAULHT vs CULR	0.291 (−0.480–1.066)	−0.350 (−1.971–1.305)	0.176 (−0.518–0.872)	0.479
TOFDT vs CULR	0.553 (−0.668–1.749)	0.367 (−1.038–1.723)	0.470 (−0.416–1.350)	0.832
TOFDT vs MCT	0.677 (−0.458–1.817)	−0.506 (−2.608–1.612)	0.427 (−0.595–1.434)	0.323
UC vs MCT	−1.334 (−3.286–0.679)	−0.159 (−1.531–1.213)	−0.532 (−1.660–0.615)	0.325
TOFDT vs RT	0.251 (−1.160–1.693)	0.403 (−1.832–2.626)	0.289 (−0.895–1.462)	0.913
UC vs RT	−0.561 (−2.661–1.535)	−0.714 (−2.344–0.906)	−0.663 (−1.914–0.587)	0.906
TOFDT vs TAULHT	−0.260 (−2.382–1.827)	0.439 (−0.619–1.496)	0.297 (−0.637–1.223)	0.551
UC vs TAULHT	−0.022 (−1.408–1.370)	−1.187 (−2.467–0.090)	−0.652 (−1.607–0.286)	0.216
UC vs TOFDT	−0.859 (−1.779–0.077)	−1.110 (−2.299–0.088)	−0.951 (−1.669–−0.226)	0.725

Supplementary Table 7.5B. Node-splitting estimates and local inconsistency tests for upper-limb function.

Comparison	Direct estimate (95% CrI)	Indirect estimate (95% CrI)	Consistency-model estimate (95% CrI)	P for inconsistency
TAULHT vs CULR	0.383 (−1.031–1.775)	0.688 (−3.078–4.446)	0.429 (−0.880–1.723)	0.880
TOFDT vs CULR	1.162 (−1.009–3.304)	0.878 (−2.438–4.192)	1.131 (−0.712–2.956)	0.891
TAULHT vs MCT	0.204 (−3.326–3.697)	−0.239 (−3.603–3.136)	−0.029 (−2.463–2.419)	0.856
TOFDT vs MCT	0.505 (−2.020–3.061)	0.982 (−3.140–5.077)	0.676 (−1.568–2.875)	0.848
TOFDT vs RT	−0.079 (−2.643–2.490)	−0.391 (−4.447–3.707)	−0.172 (−2.394–2.044)	0.898
UC vs RT	−1.202 (−4.653–2.265)	−0.861 (−4.186–2.500)	−1.035 (−3.475–1.411)	0.894
UC vs TAULHT	−0.191 (−3.686–3.284)	−0.205 (−3.381–2.906)	−0.158 (−2.469–2.182)	0.992
UC vs TOFDT	−0.751 (−3.275–1.810)	−1.016 (−3.980–1.953)	−0.858 (−2.814–1.081)	0.901

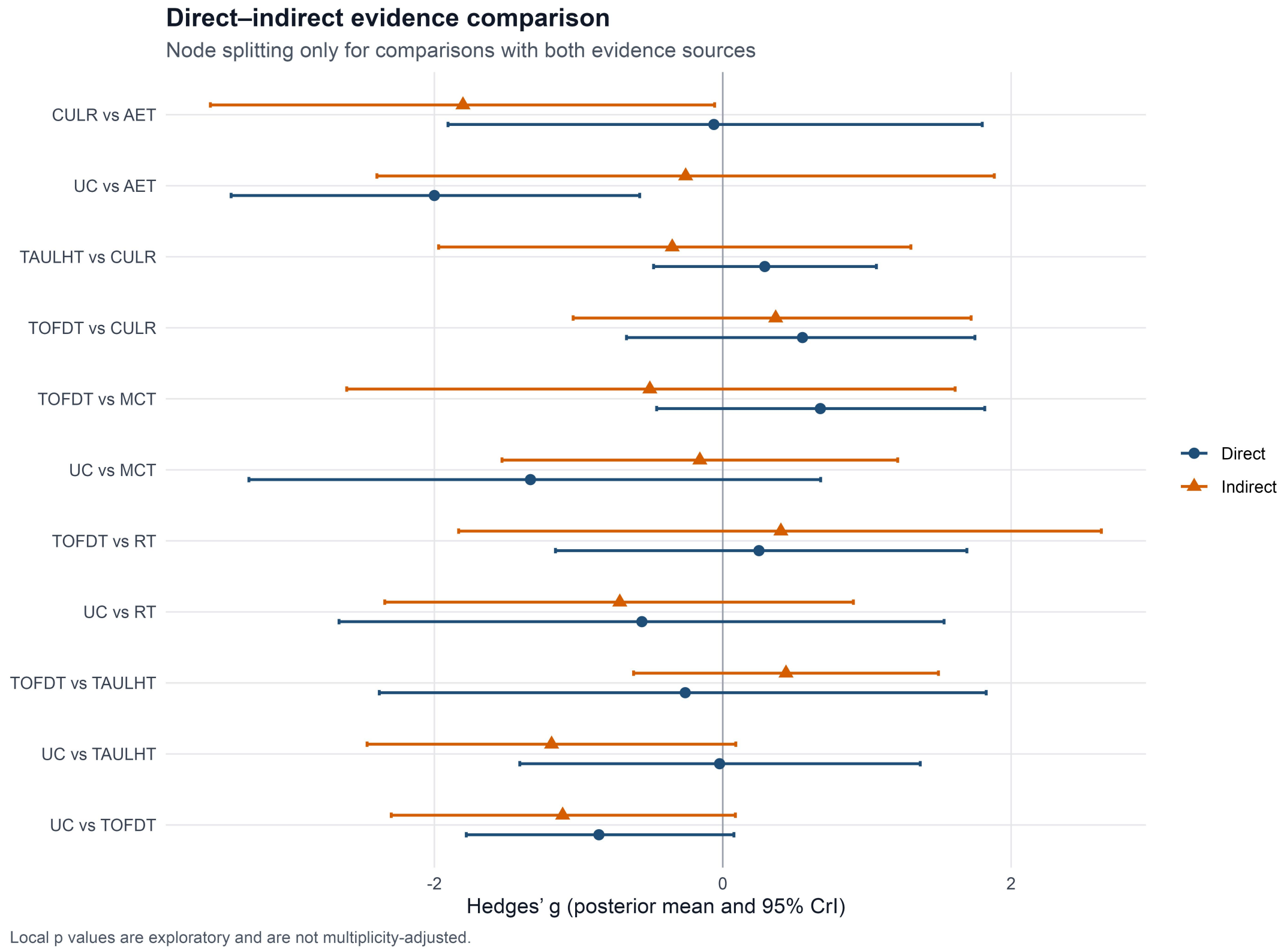

Supplementary Fig. 7.5A. Node-splitting comparison of direct and indirect evidence for finger dexterity.

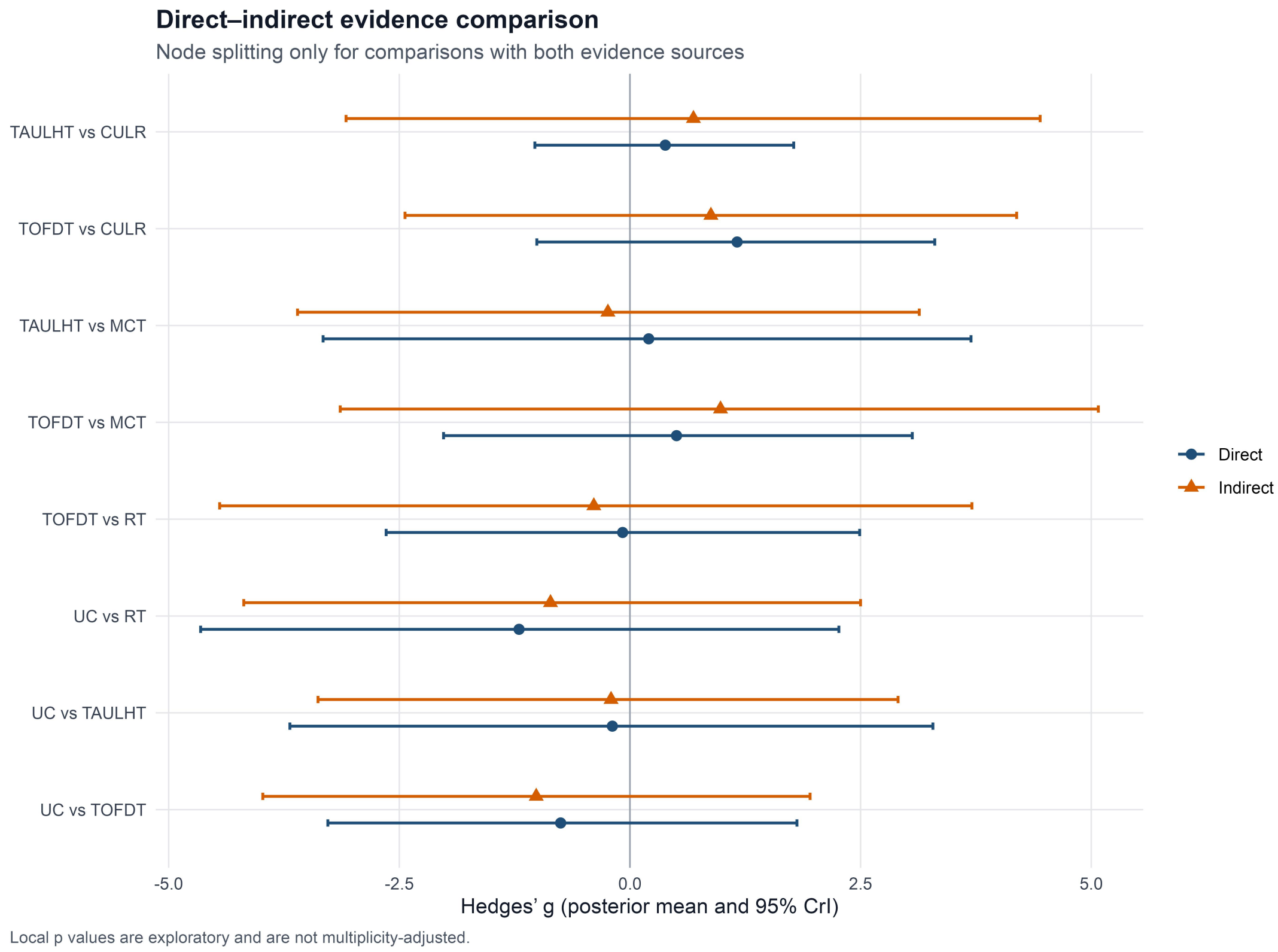

Supplementary Fig. 7.5B. Node-splitting comparison of direct and indirect evidence for upper-limb function.

S7.6 Exploratory subgroup and treatment-by-subgroup interaction analyses

Supplementary Table 7.6A. Subgroup-specific heterogeneity and treatment rankings for finger dexterity.

Subgroup	Studies (arms)	Tau, median (95% CrI)	Highest-SUCRA treatment	SUCRA
Intervention duration				
<8 weeks	15 (30)	0.744 (0.423–1.414)	TAULHT	0.799
≥8 weeks	15 (30)	1.172 (0.723–1.861)	AET	0.821
Mean age				
<65 years	13 (26)	0.581 (0.190–1.399)	CULR	0.712
≥65 years	17 (34)	0.477 (0.213–0.958)	AET	0.999
Outcome assessment type				
Pegboard-based	24 (48)	0.540 (0.315–0.889)	TOFDT	0.922
Non-pegboard	6 (12)	0.913 (0.048–1.936)	AET	0.919

Note: Numbers of studies and treatment arms are reported as studies (arms). τ represents the posterior between-study standard deviation from the random-effects model. The highest-ranked treatment was determined using SUCRA. Higher SUCRA values indicate a greater probability of a favorable ranking but do not establish treatment superiority. Rankings for AET in the ≥65-year and non-pegboard subgroups were each supported by a single study and should be interpreted cautiously. The non-pegboard network was sparse.

Supplementary Table 7.6B. Bayesian treatment-by-subgroup interaction estimates for finger dexterity.

Subgroup dimension	Contrast	Interaction β, median (95% CrI)	Pr(β>0)
Intervention duration	≥8 vs <8 weeks	−0.085 (−1.269 to 1.102)	0.443
Mean age	≥65 vs <65 years	0.992 (−0.033 to 2.025)	0.972
Outcome assessment type	Non-pegboard vs pegboard	1.343 (0.058 to 2.680)	0.980

Note: Interaction estimates are presented as posterior medians with 95% credible intervals. Positive coefficients indicate numerically greater treatment effects in the second category of each contrast. A shared interaction coefficient was assumed across all non-UC treatments. Independent subgroup estimates were not used to infer between-subgroup differences.

Supplementary Table 7.6C. Subgroup-specific heterogeneity and treatment rankings for upper-limb function.

Subgroup	Studies (arms)	Tau, median (95% CrI)	Highest-SUCRA treatment	SUCRA
Intervention duration				
<8 weeks	11 (22)	1.162 (0.559–1.910)	TOFDT	0.801
≥8 weeks	12 (24)	1.931 (1.697–1.997)	MBE	0.996
Mean age				
<65 years	10 (20)	1.935 (1.712–1.998)	MBE	0.983
≥65 years	13 (26)	0.690 (0.328–1.470)	TOFDT	0.892

Subgroup	Studies (arms)	Tau, median (95% CrI)	Highest-SUCRA treatment	SUCRA
Outcome assessment type				
Objective/clinician assessed	17 (34)	1.918 (1.664–1.997)	MBE	0.999
Patient/goal-reported	6 (12)	0.812 (0.054–1.911)	TOFDT	0.863

Note: Numbers of studies and treatment arms are reported as studies (arms). τ represents the posterior between-study standard deviation from the random-effects model. The highest-ranked treatment was determined using SUCRA. Higher SUCRA values indicate a greater probability of a favorable ranking but do not establish treatment superiority. Rankings for MBE in the ≥8-week, <65-year, and objective/clinician-assessed subgroups were each supported by two studies and should be interpreted cautiously. The <8-week and patient/goal-reported networks each had only one study directly providing UC anchoring and were considered sparse. Heterogeneity was high in the ≥8-week, <65-year, and objective/clinician-assessed subgroups.

Supplementary Table 7.6D. Bayesian treatment-by-subgroup interaction estimates for upper-limb function.

Subgroup dimension	Contrast	Interaction β, median (95% CrI)	Pr(β>0)
Intervention duration	≥8 vs <8 weeks	0.188 (−2.785 to 3.457)	0.556
Mean age	≥65 vs <65 years	−0.174 (−3.177 to 2.794)	0.447
Outcome assessment type	Patient/goal-reported vs objective/clinician assessed	−0.322 (−3.718 to 2.689)	0.409

Note: Interaction estimates are presented as posterior medians with 95% credible intervals. Positive coefficients indicate numerically greater treatment effects in the second category of each contrast. A shared interaction coefficient was assumed across all non-UC treatments. Independent subgroup estimates were not used to infer between-subgroup differences. All three 95% credible intervals included zero; therefore, no clear treatment-by-subgroup interaction was identified.

S7.7 Sensitivity analyses of relative treatment effects in the conventional Bayesian network meta-analysis

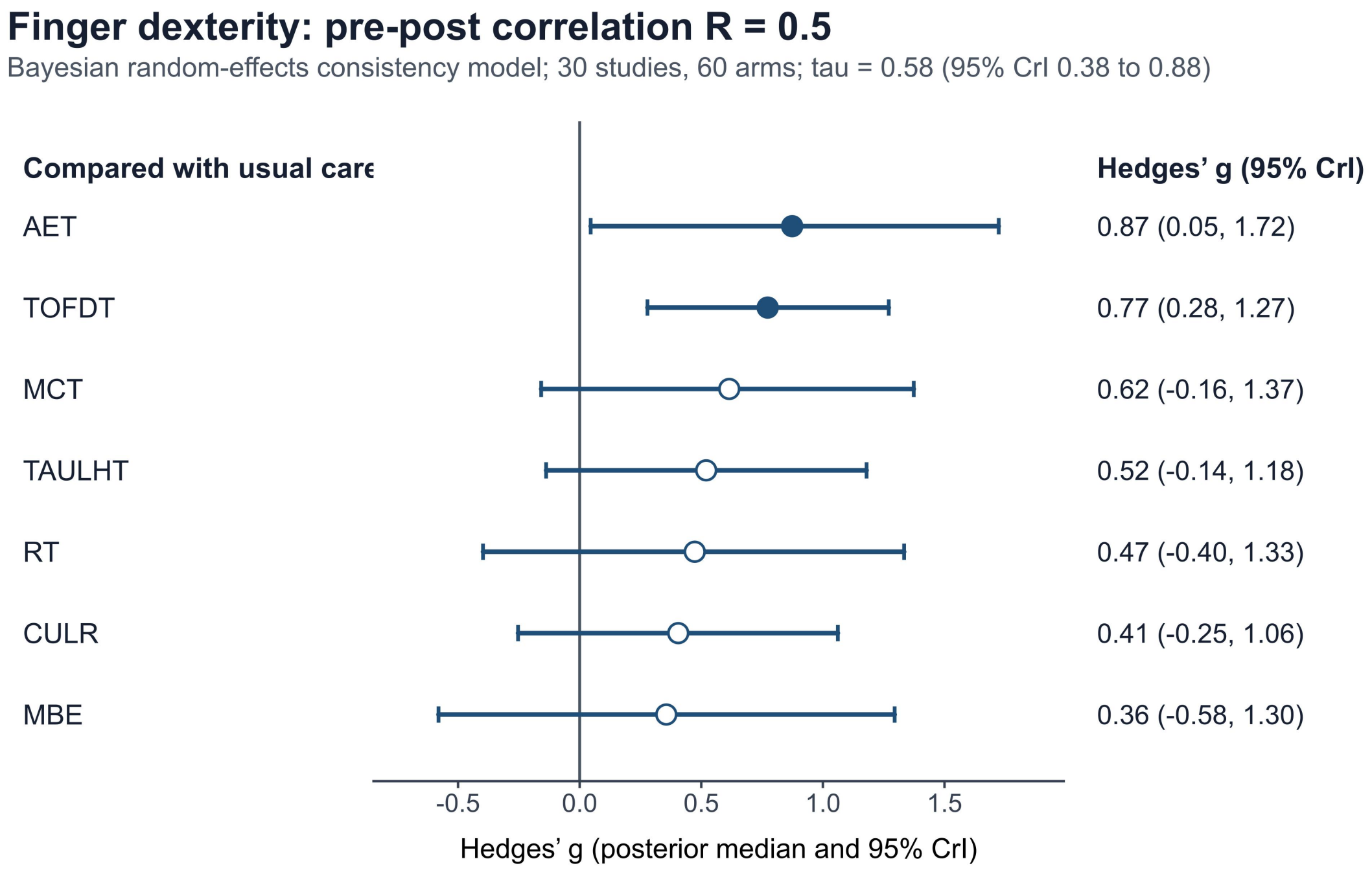

Supplementary Fig. 7.7A. Sensitivity analysis of relative effects versus usual care for finger dexterity assuming a pre–post correlation of R = 0.5.

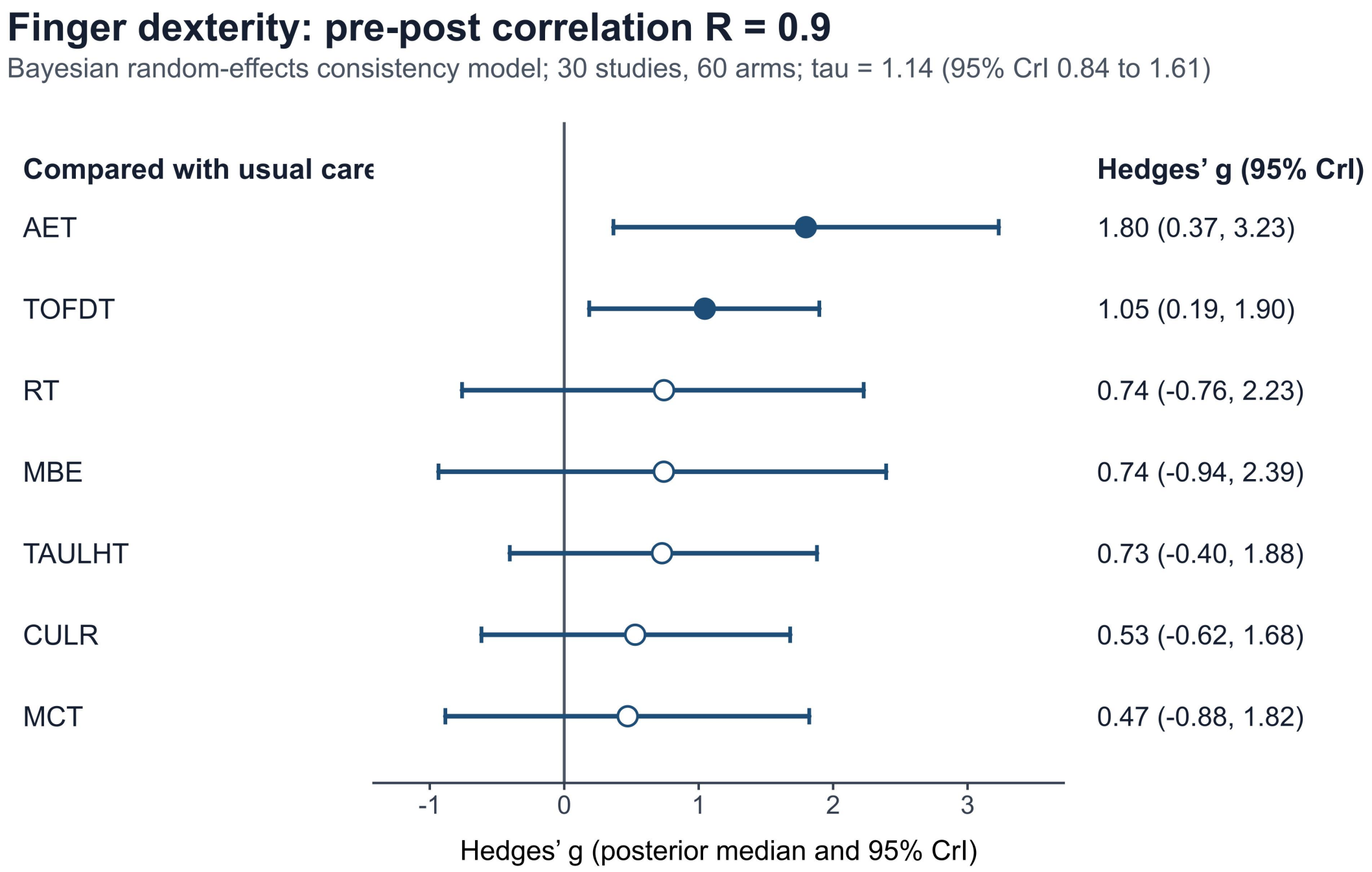

Supplementary Fig. 7.7B. Sensitivity analysis of relative effects versus usual care for finger dexterity assuming a pre–post correlation of R = 0.9.

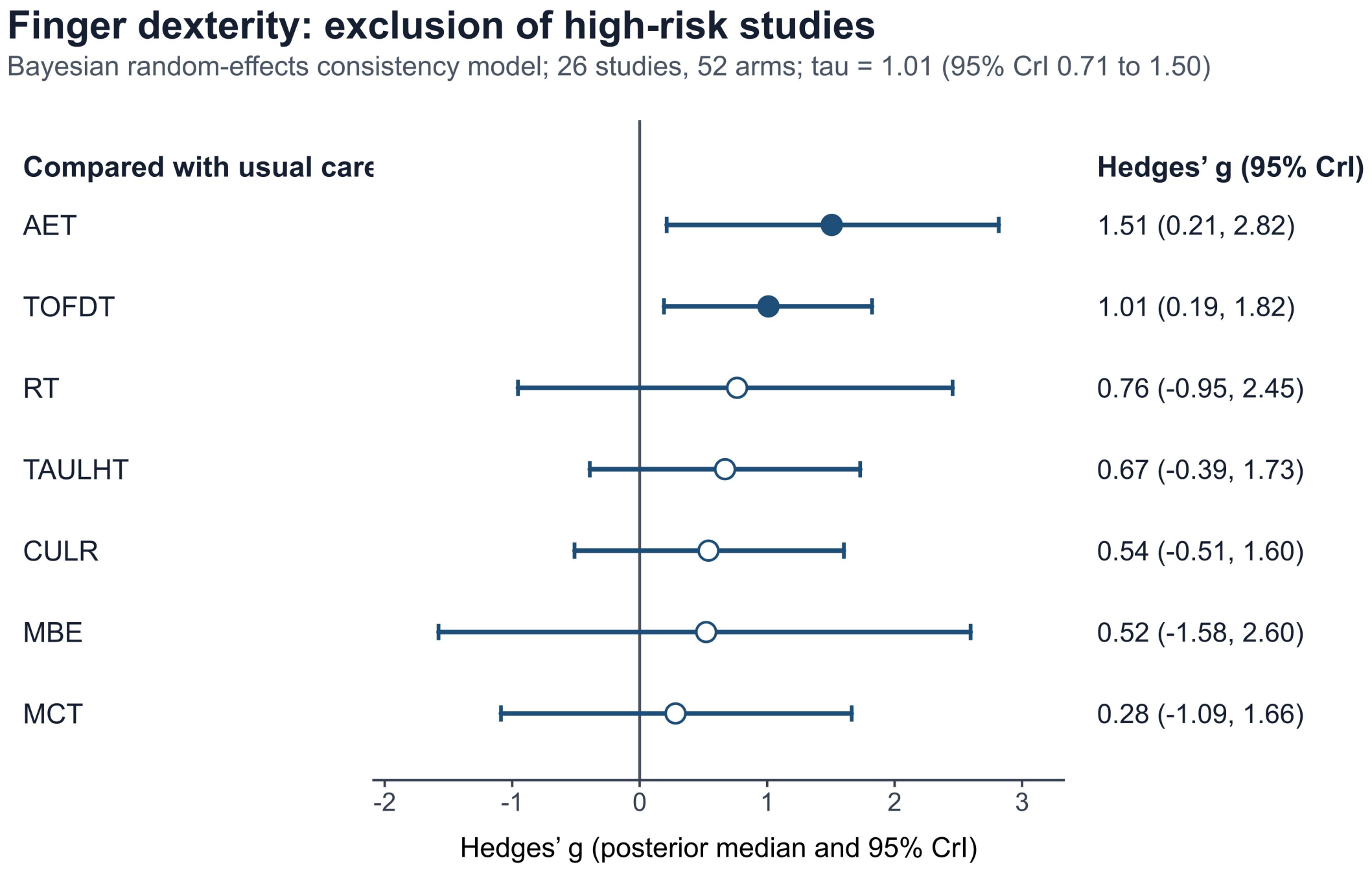

Supplementary Fig. 7.7C. Sensitivity analysis of relative effects versus usual care for finger dexterity after excluding studies at high risk of bias.

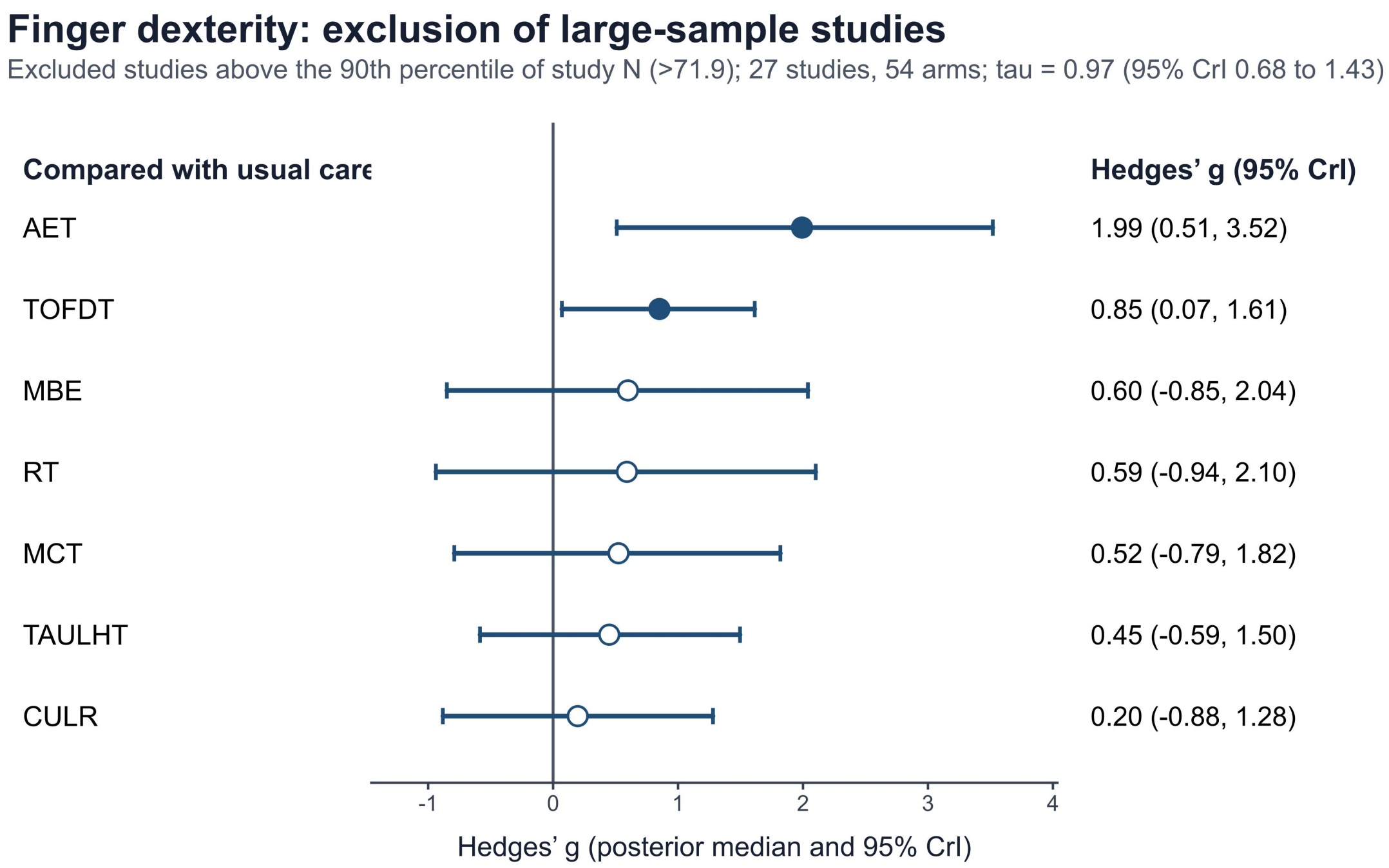

Supplementary Fig. 7.7D. Sensitivity analysis of relative effects versus usual care for finger dexterity after excluding studies with total sample sizes above the outcome-specific 90th percentile.

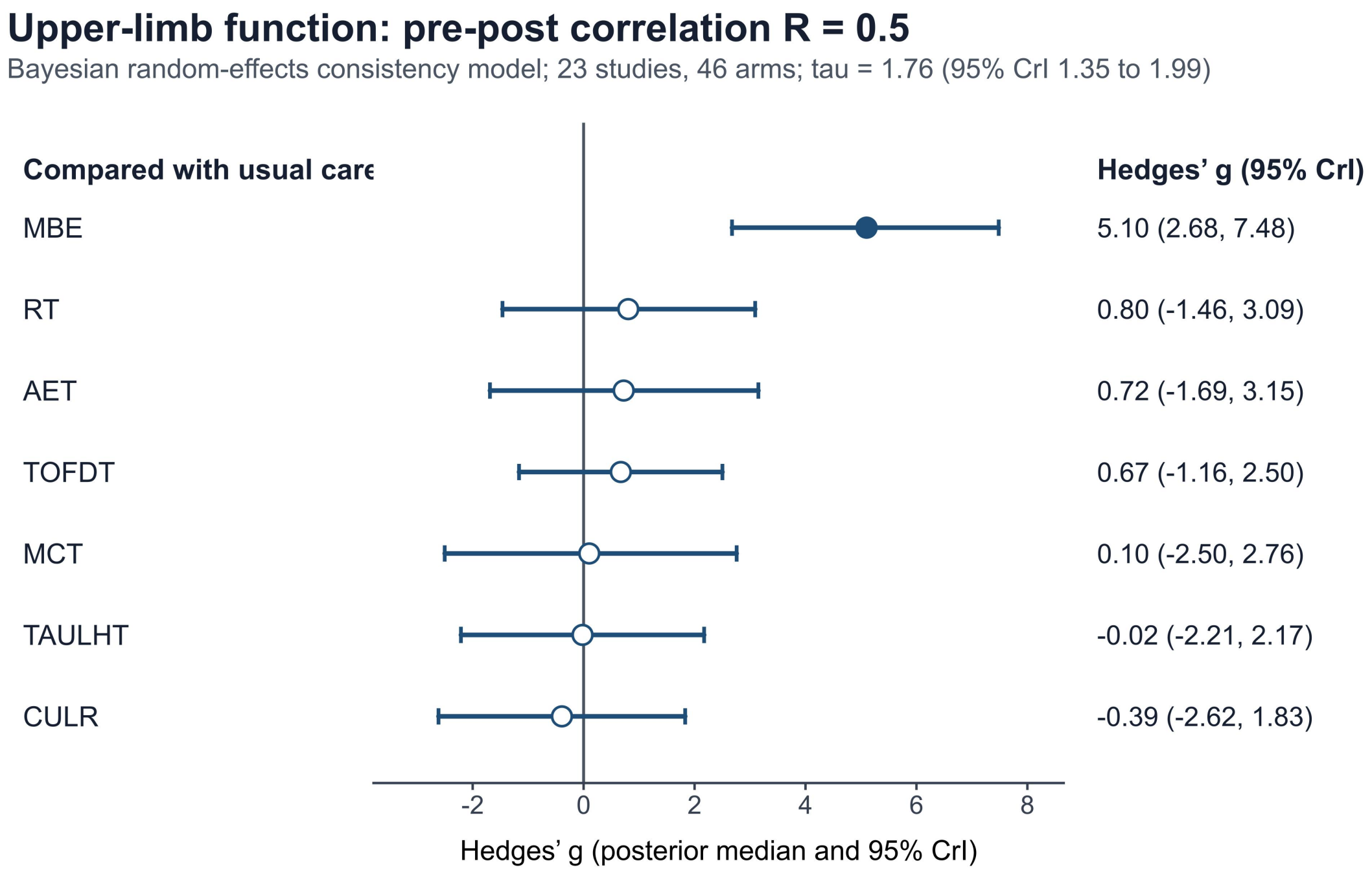

Supplementary Fig. 7.7E. Sensitivity analysis of relative effects versus usual care for upper-limb function assuming a pre–post correlation of R = 0.5.

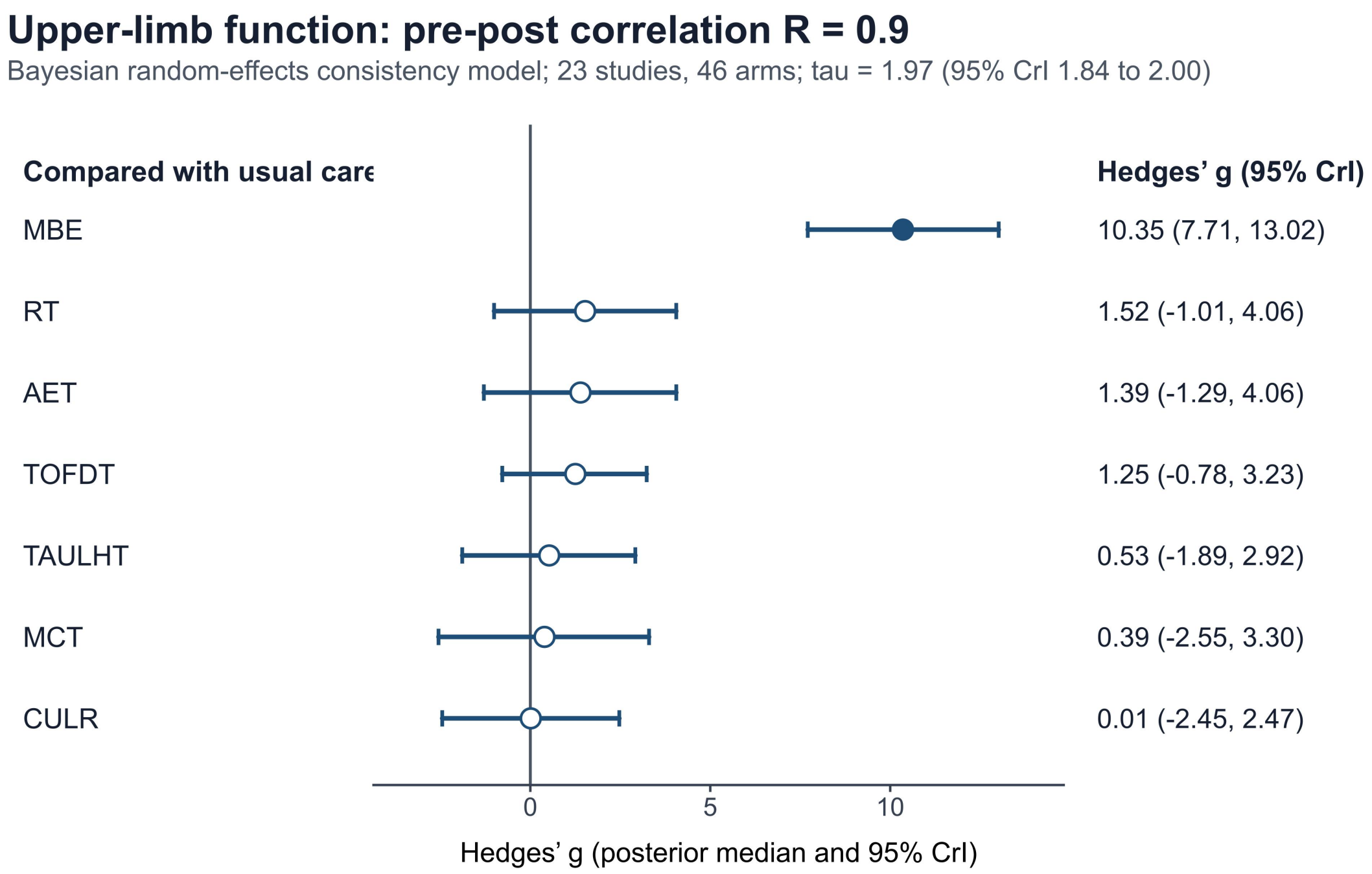

Supplementary Fig. 7.7F. Sensitivity analysis of relative effects versus usual care for upper-limb function assuming a pre–post correlation of R = 0.9.

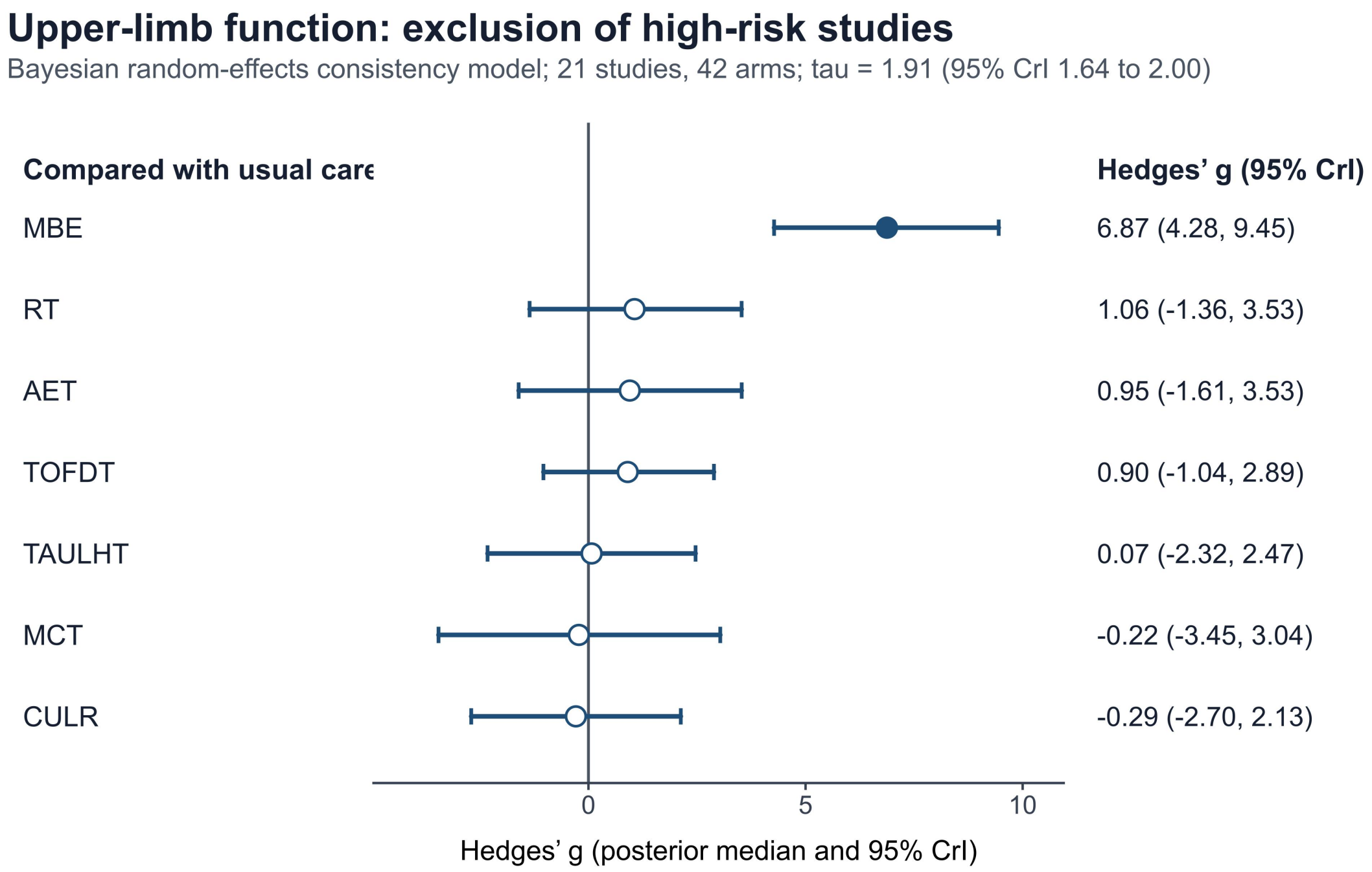

Supplementary Fig. 7.7G. Sensitivity analysis of relative effects versus usual care for upper-limb function after excluding studies at high risk of bias.

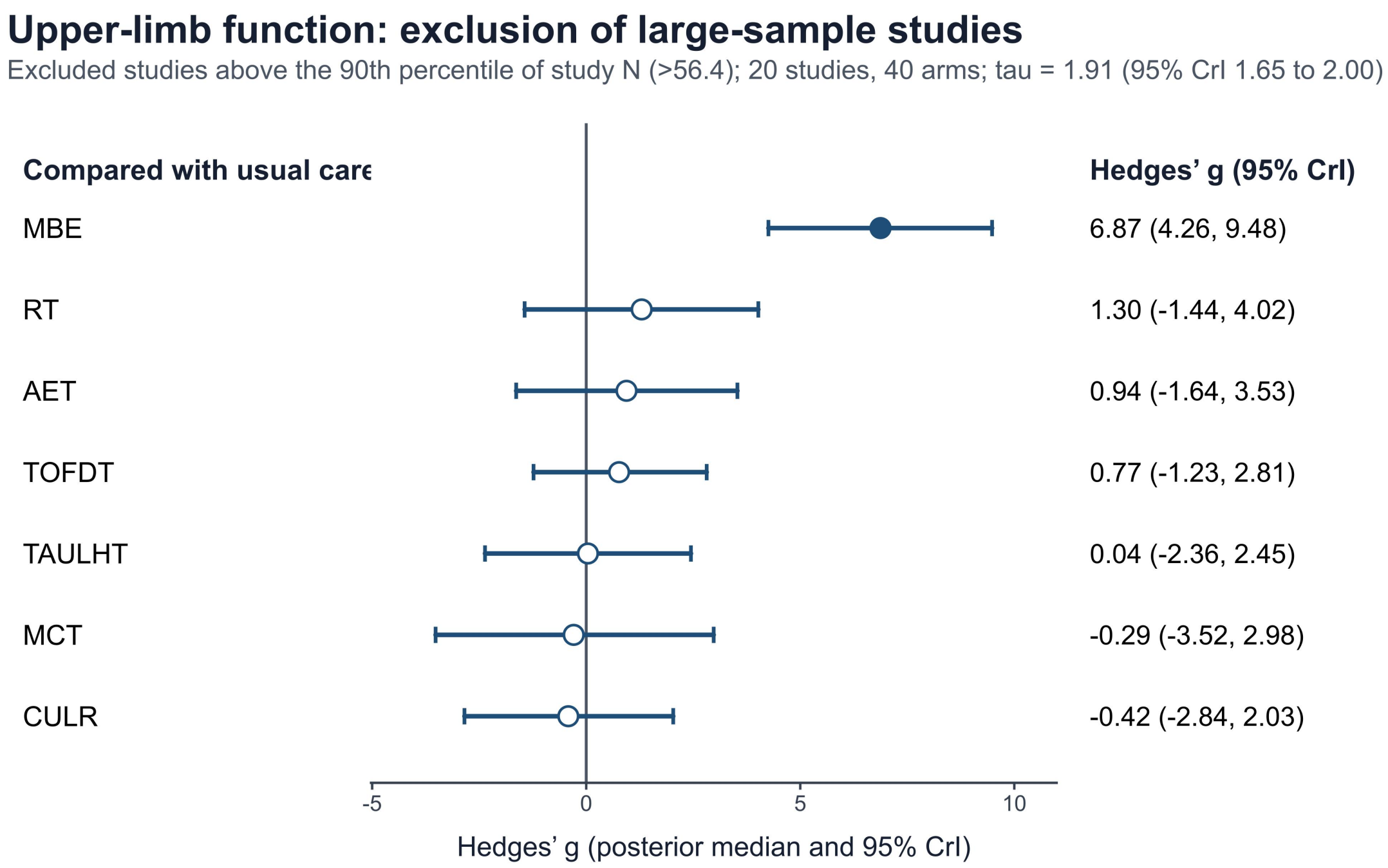

Supplementary Fig. 7.7H. Sensitivity analysis of relative effects versus usual care for upper-limb function after excluding studies with total sample sizes above the outcome-specific 90th percentile.

Note: Points represent posterior median Hedges' g values, and horizontal lines represent 95% credible intervals (CrIs), estimated using Bayesian random-effects consistency models with usual care (UC) as the reference. Positive values favour the listed exercise modality. Filled circles indicate that the 95% CrI

excludes zero, whereas open circles indicate that the 95% CrI includes zero. Panels A and E assume a pre–post correlation of R = 0.5, and panels B and F assume R = 0.9 when calculating change-score standard deviations. Panels C and G exclude studies at high risk of bias. Panels D and H exclude studies with total sample sizes above the outcome-specific 90th percentile. The numbers of studies and treatment arms and the posterior between-study standard deviation (τ) are reported within each panel. AET, aerobic exercise; CULR, conventional upper-limb rehabilitation; MBE, mind–body exercise; MCT, multicomponent training; RT, resistance training; TAULHT, technology-assisted upper-limb or hand training; TOFDT, task-oriented finger dexterity training; UC, usual care.

S7.8 Small-study effects and publication-bias diagnostics

Supplementary Table 7.8. Exploratory Egger regression for small-study effects.

Outcome	Egger coefficient	SE	P value
Finger dexterity	−1.278	1.670	0.450
Upper-limb function	−0.313	5.142	0.952

Note: Egger regression was based on comparison-adjusted effects and was considered exploratory. SE, standard error.

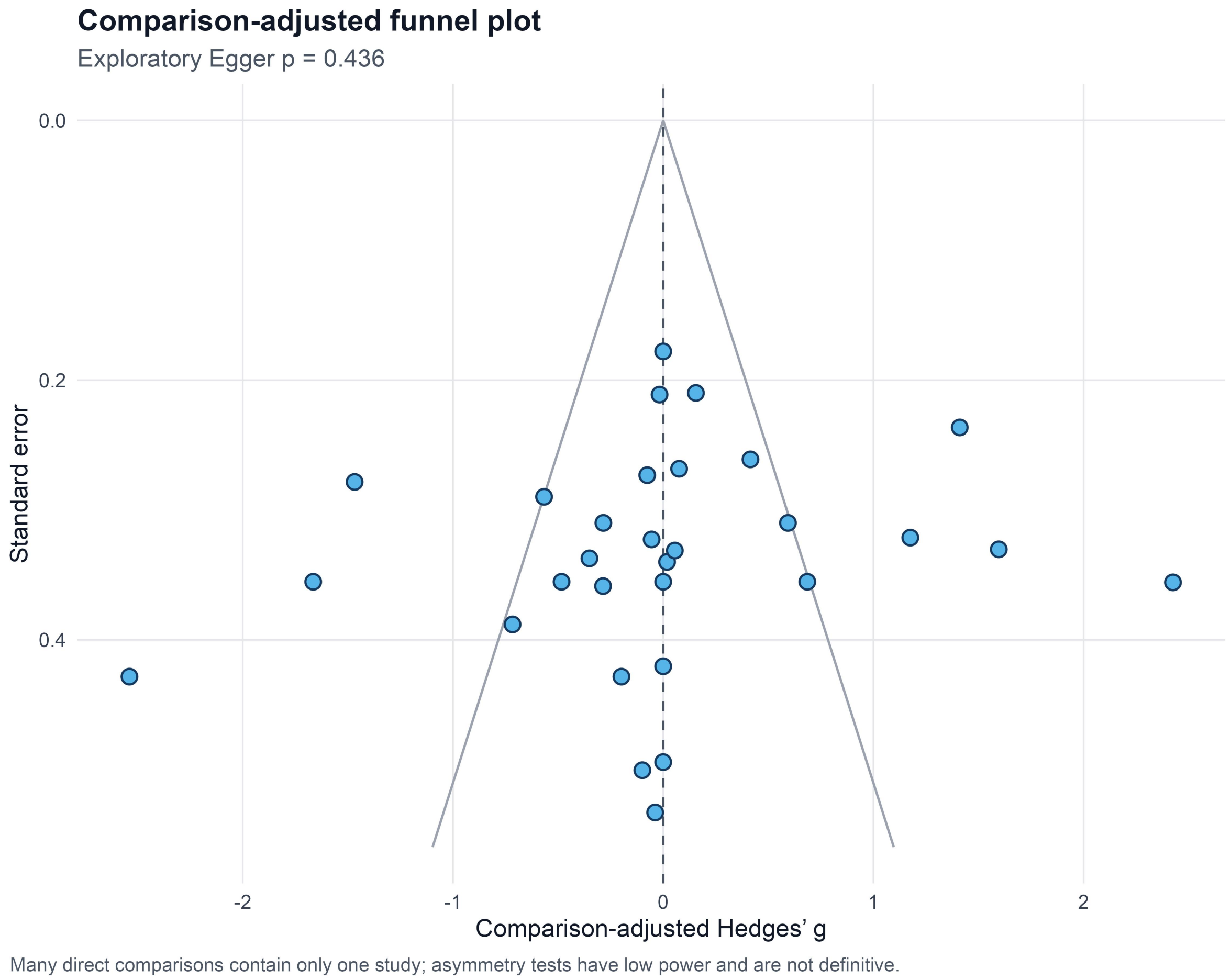

Supplementary Fig. 7.8A. Comparison-adjusted funnel plot for finger dexterity.

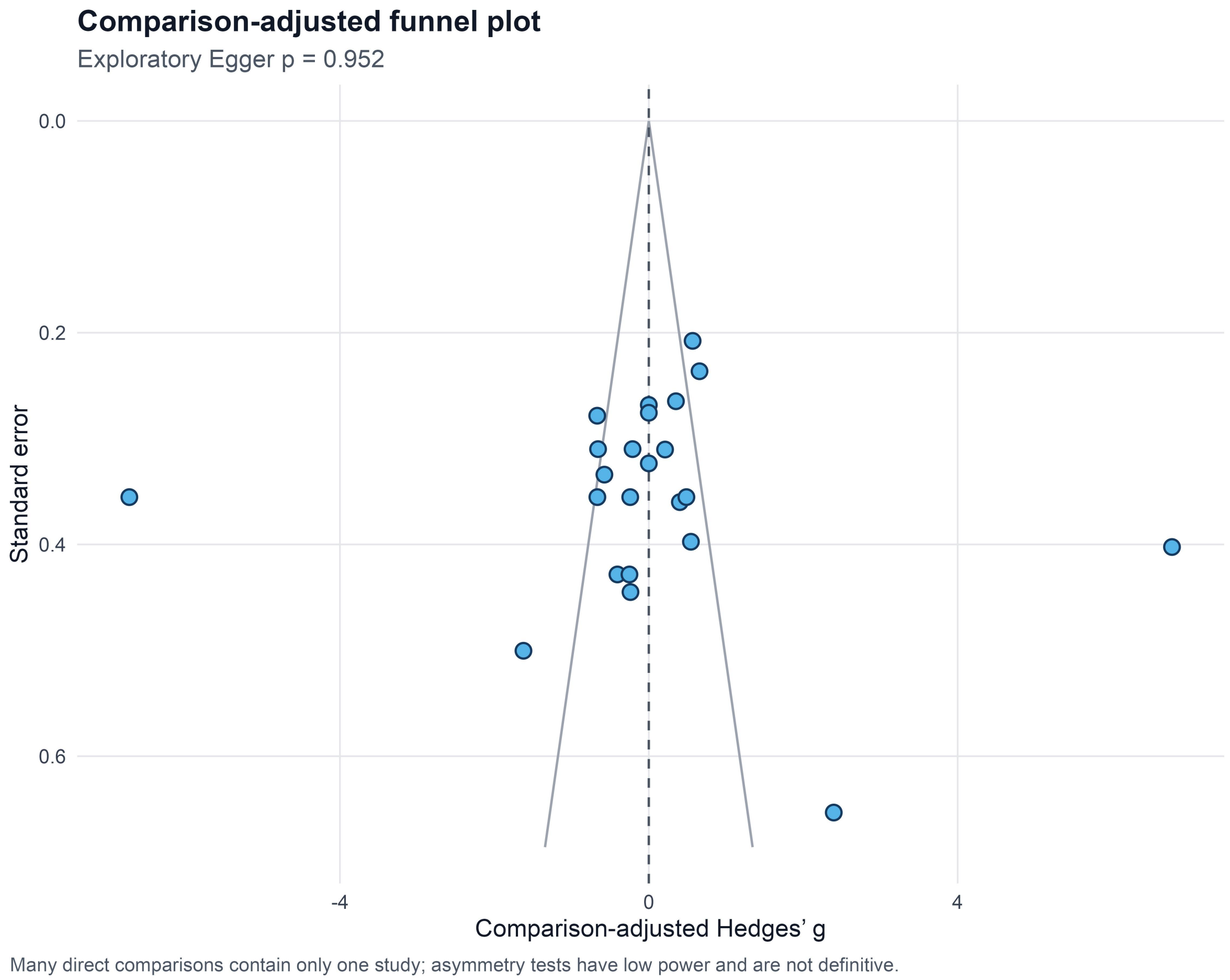

Supplementary Fig. 7.8B. Comparison-adjusted funnel plot for upper-limb function.

Supplementary File 8: Bayesian dose–response network meta-analysis

S8.1 Observed dose distribution and data support

Supplementary Table 8.1A. Observed dose support for finger dexterity.

Exercise category	No. of contributing studies	No. of active arms	No. of distinct observed doses	Observed dose range, MET-min/week	Total participants
Overall exercise	23	33	29	120–1,800	764
AET	3	3	3	412.8–540	91
CULR	11	11	11	180–1,566	225
MBE	2	2	2	420–450	53
MCT	4	4	4	510–1,596	109
RT	3	3	3	420–1,575	71
TAULHT	10	10	10	135–1,728	158
TOFDT	14	14	13	120–1,800	302

Note: Of 30 eligible studies, 23 contributed to the pooled overall exercise dose–response model; studies with identical overall dose nodes across arms contributed only to modality-specific analyses. Active-arm and participant counts exclude zero-dose UC arms and should not be summed across exercise categories. Distinct doses and ranges represent exact observed weekly doses and indicate data support rather than recommended doses; these exact values were mapped to prespecified nodes before the node representative values entered the primary model.

Supplementary Table 8.1B. Observed dose support for upper-limb function.

Exercise category	No. of contributing studies	No. of active arms	No. of distinct observed doses	Observed dose range, MET-min/week	Total participants
Overall exercise	15	22	20	120–1,800	418
AET	2	2	2	396–412.8	23
CULR	10	10	9	180–1,566	144
MBE	2	2	2	360–720	26
MCT	3	3	3	360–684	76
RT	3	3	3	280–1,575	87
TAULHT	9	9	9	180–1,728	158
TOFDT	9	9	8	120–1,800	191

Note: Of 23 eligible studies, 15 contributed to the pooled overall exercise dose–response model; studies with identical overall dose nodes across arms contributed only to modality-specific analyses. Active-arm and participant counts exclude zero-dose UC arms and should not be summed across exercise categories. Distinct doses and ranges represent exact observed weekly doses and indicate data support rather than recommended doses; these exact values were mapped to prespecified nodes before the node representative values entered the primary model.

Abbreviations for Tables 7.1A and 7.1B: AET, aerobic exercise; CULR, conventional upper-limb rehabilitation; MBE, mind–body exercise; MCT, multicomponent training; MET, metabolic equivalent of task; RT, resistance

training; TAULHT, technology-assisted upper-limb or hand training; TOFDT, task-oriented finger dexterity training; UC, usual care or no structured exercise.

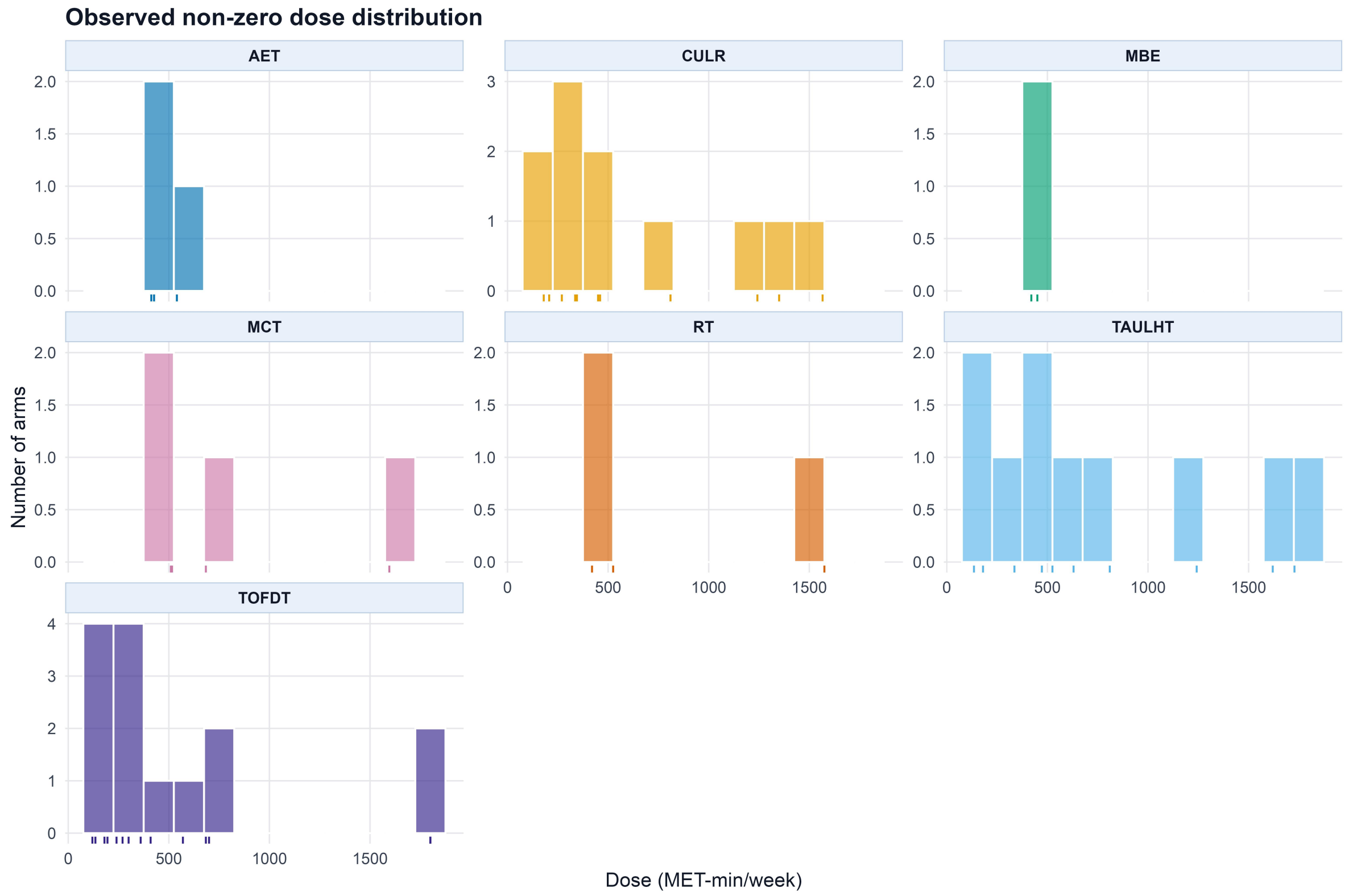

Supplementary Fig. 8.1A. Observed distribution of non-zero weekly exercise doses included in the Bayesian dose–response network meta-analysis for finger dexterity

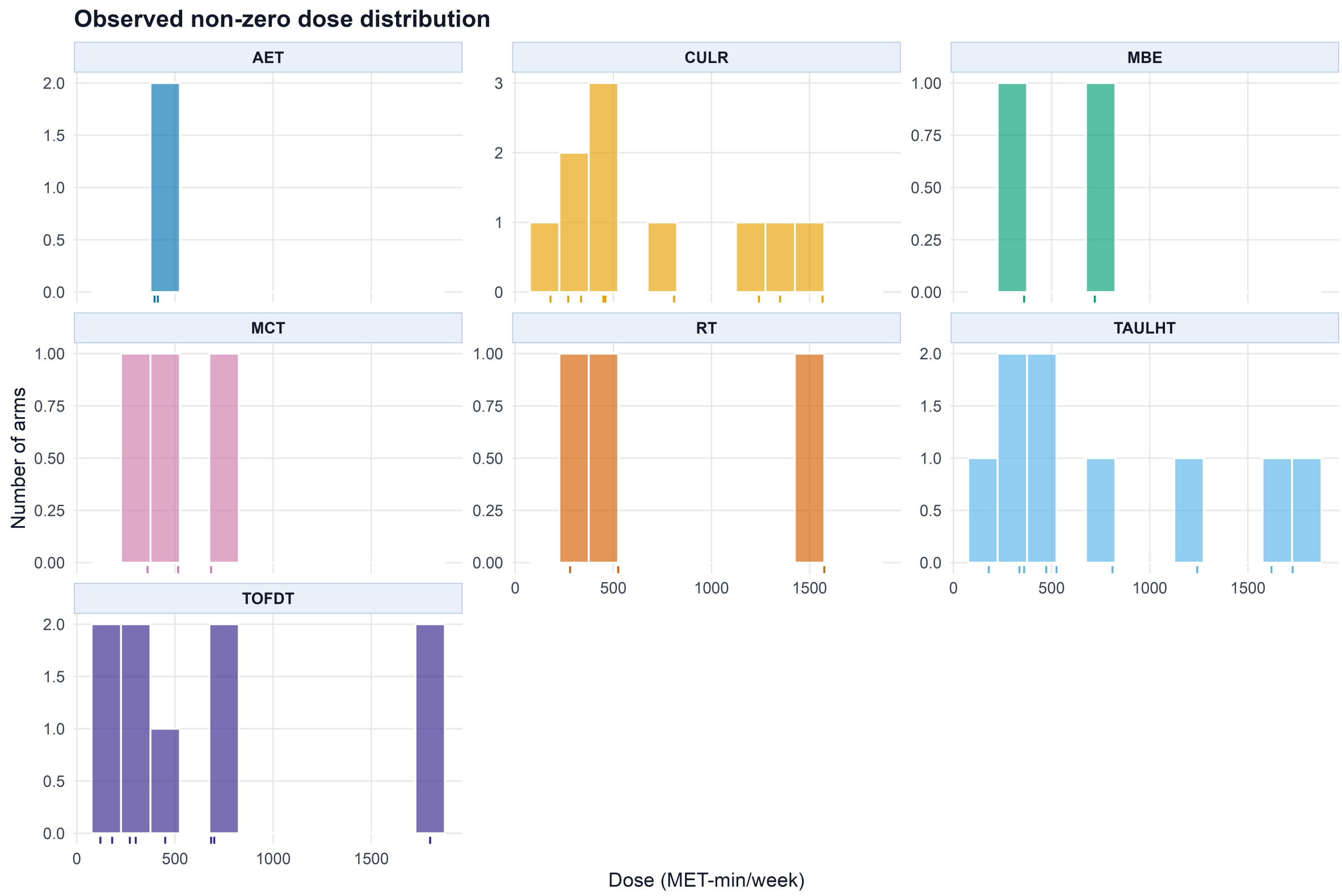

Supplementary Fig. 8.1B. Observed distribution of non-zero weekly exercise doses included in the Bayesian dose–response network meta-analysis for upper-limb function

S8.2 Dose–response model selection, fit, convergence, and consistency

Supplementary Table 8.2A. Candidate overall-exercise dose–response models for finger dexterity.

Dose function	Effects model	DIC	ΔDIC	Residual dev.	τ	Max PSRF	Min ESS	Primary
Linear	Random	38.48	0.26	47.38	0.865	1.001	4,500	Yes
Emax	Random	38.22	0.00	47.33	0.815	1.001	3,500	No
Exponential	Random	38.77	0.55	47.32	0.823	1.001	3,100	No
Log-linear	Random	39.15	0.93	47.28	0.831	1.002	2,400	No
Quadratic	Random	39.16	0.94	47.12	0.854	1.001	4,500	No
Natural cubic spline	Random	40.73	2.51	47.46	0.811	1.001	3,200	No
Natural cubic spline	Common	103.24	65.02	133.54	–	1.002	1,400	No
Emax	Common	118.43	80.21	148.99	–	1.004	1,200	No
Exponential	Common	125.83	87.61	159.64	–	1.001	3,200	No
Log-linear	Common	126.30	88.08	160.56	–	1.001	4,500	No
Quadratic	Common	130.23	92.01	162.68	–	1.001	2,600	No
Linear	Common	132.69	94.47	166.49	–	1.001	4,500	No

Supplementary Table 8.2B. Candidate modality-specific dose–response models for finger dexterity.

Dose function	Effects model	DIC	ΔDIC	Residual dev.	τ	Max PSRF	Min ESS	Primary
Linear	Random	210.35	0.42	60.24	0.955	1.003	890	Yes
Natural cubic spline	Random	209.93	0.00	60.40	0.924	1.003	870	No
Exponential	Random	210.38	0.45	60.48	0.908	1.002	2,000	No
Quadratic	Random	211.79	1.86	60.71	0.917	1.002	1,500	No
Emax	Random	212.55	2.62	60.95	0.917	1.003	1,400	No
Log-linear	Random	214.97	5.04	61.04	0.915	1.001	2,500	No
Natural cubic spline	Common	289.31	79.38	142.08	–	1.003	1,000	No
Quadratic	Common	317.11	107.17	180.72	–	1.002	1,900	No
Emax	Common	333.99	124.06	202.25	–	1.008	390	No
Exponential	Common	342.46	132.53	216.37	–	1.002	1500	No
Log-linear	Common	348.80	138.86	223.86	–	1.001	4,200	No
Linear	Common	366.20	156.27	239.32	–	1.003	780	No

Supplementary Table 8.2C. Candidate overall-exercise dose–response models for upper-limb function.

Dose function	Effects model	DIC	ΔDIC	Residual dev.	τ	Max PSRF	Min ESS	Primary
Exponential	Random	28.50	0.56	30.92	1.951	1.002	2,300	Yes
Emax	Random	27.94	0.00	30.94	1.953	1.001	3,700	No
Natural cubic spline	Random	28.31	0.37	30.82	1.949	1.002	2,100	No
Linear	Random	28.56	0.62	31.06	1.957	1.002	2,300	No
Log-linear	Random	29.43	1.49	30.90	1.954	1.001	4,500	No

Dose function	Effects model	DIC	ΔDIC	Residual dev.	τ	Max PSRF	Min ESS	Primary
Quadratic	Random	29.55	1.61	30.85	1.951	1.001	4,400	No
Natural cubic spline	Common	929.87	901.93	936.05	–	1.002	1,700	No
Quadratic	Common	1,150.98	1,123.03	1,167.61	–	1.003	1,100	No
Emax	Common	1,238.56	1,210.62	1,255.37	–	1.002	1,500	No
Exponential	Common	1,280.53	1,252.59	1,299.42	–	1.001	3,500	No
Log-linear	Common	1,326.54	1,298.59	1,344.63	–	1.001	4,500	No
Linear	Common	1,413.83	1,385.88	1,431.42	–	1.001	4,400	No

Supplementary Table 8.2D. Candidate modality-specific dose–response models for upper-limb function.

Dose function	Effects model	DIC	ΔDIC	Residual dev.	τ	Max PSRF	Min ESS	Primary
Linear	Random	188.14	0.58	46.62	1.884	1.002	1,500	Yes
Natural cubic spline	Random	187.56	0.00	46.56	1.848	1.003	980	No
Emax	Random	187.65	0.09	46.71	1.899	1.002	1,300	No
Exponential	Random	189.18	1.62	46.45	1.925	1.001	3,500	No
Quadratic	Random	190.11	2.56	46.25	1.815	1.002	2,100	No
Log-linear	Random	190.77	3.21	46.63	1.925	1.002	1,600	No
Natural cubic spline	Common	230.49	42.93	71.22	–	1.002	1,500	No
Quadratic	Common	359.10	171.54	181.36	–	1.001	2,900	No
Linear	Common	476.25	288.70	345.28	–	1.002	1,300	No
Log-linear	Common	538.77	351.22	397.57	–	1.002	1,600	No
Exponential	Common	555.26	367.70	413.77	–	1.001	4,500	No
Emax	Common	649.46	461.91	464.14	–	1.004	670	No

Note for Supplementary Tables 8.2A–D: All candidate models used the mapped dose-node representative values as the dose input. Predictions between node values are interpolations; predictions above the highest node but within the original exact-dose support range are limited extrapolations. Lower DIC values indicate better relative fit; ΔDIC was calculated relative to the lowest DIC among successfully fitted models.Primary models were selected among eligible random-effects models after convergence, residual-fit, and prediction-stability checks; models within ΔDIC ≤2 were considered equivalent, with the more parsimonious dose function preferred. PSRF ≤1.05 and ESS ≥200 indicated adequate convergence. τ denotes the posterior between-study standard deviation and is not applicable to common-effect models. DIC, deviance information criterion; ESS, effective sample size; PSRF, potential scale reduction factor.

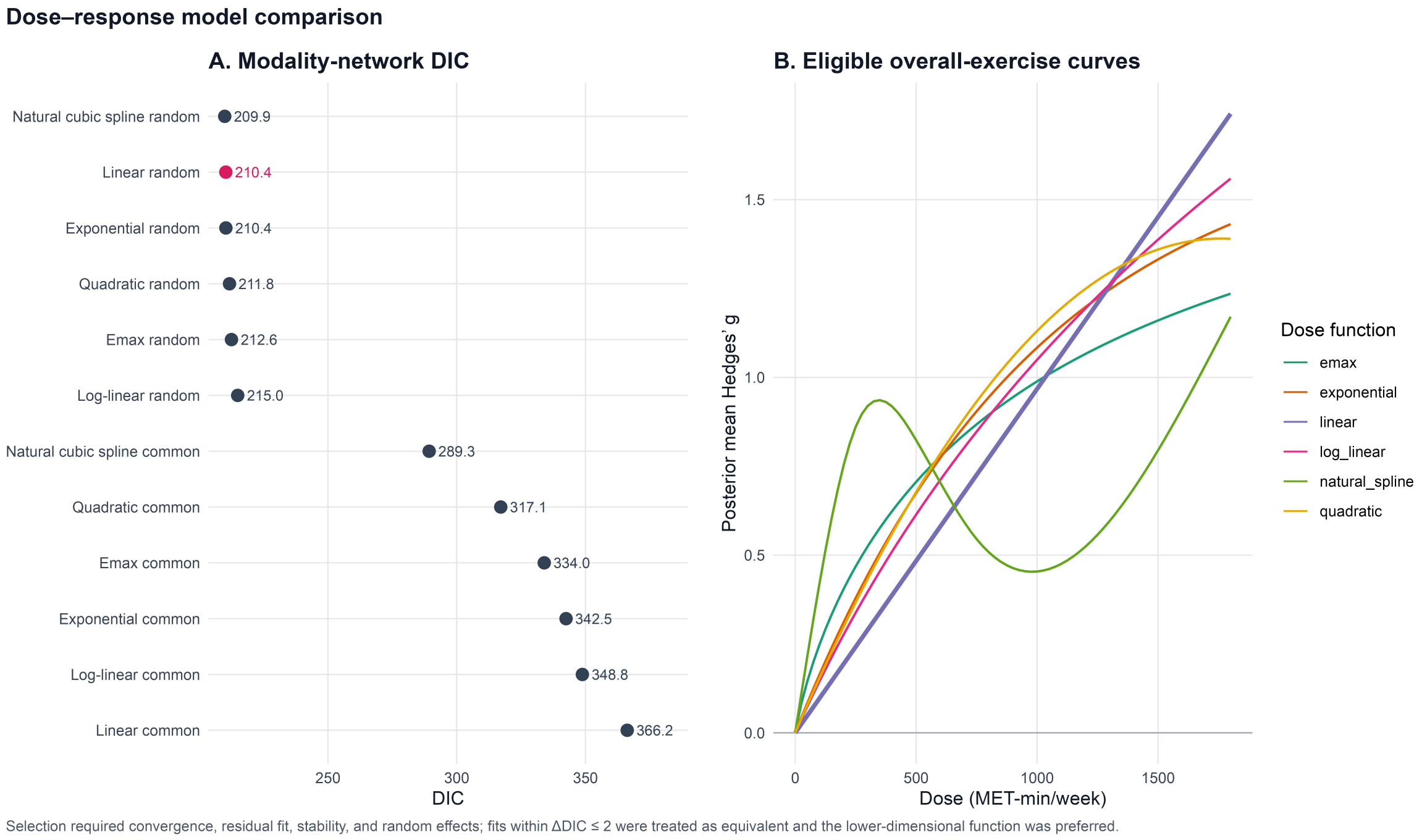

Supplementary Fig. 8.2A. Comparison of candidate modality-specific and overall-exercise dose–response models for finger dexterity.

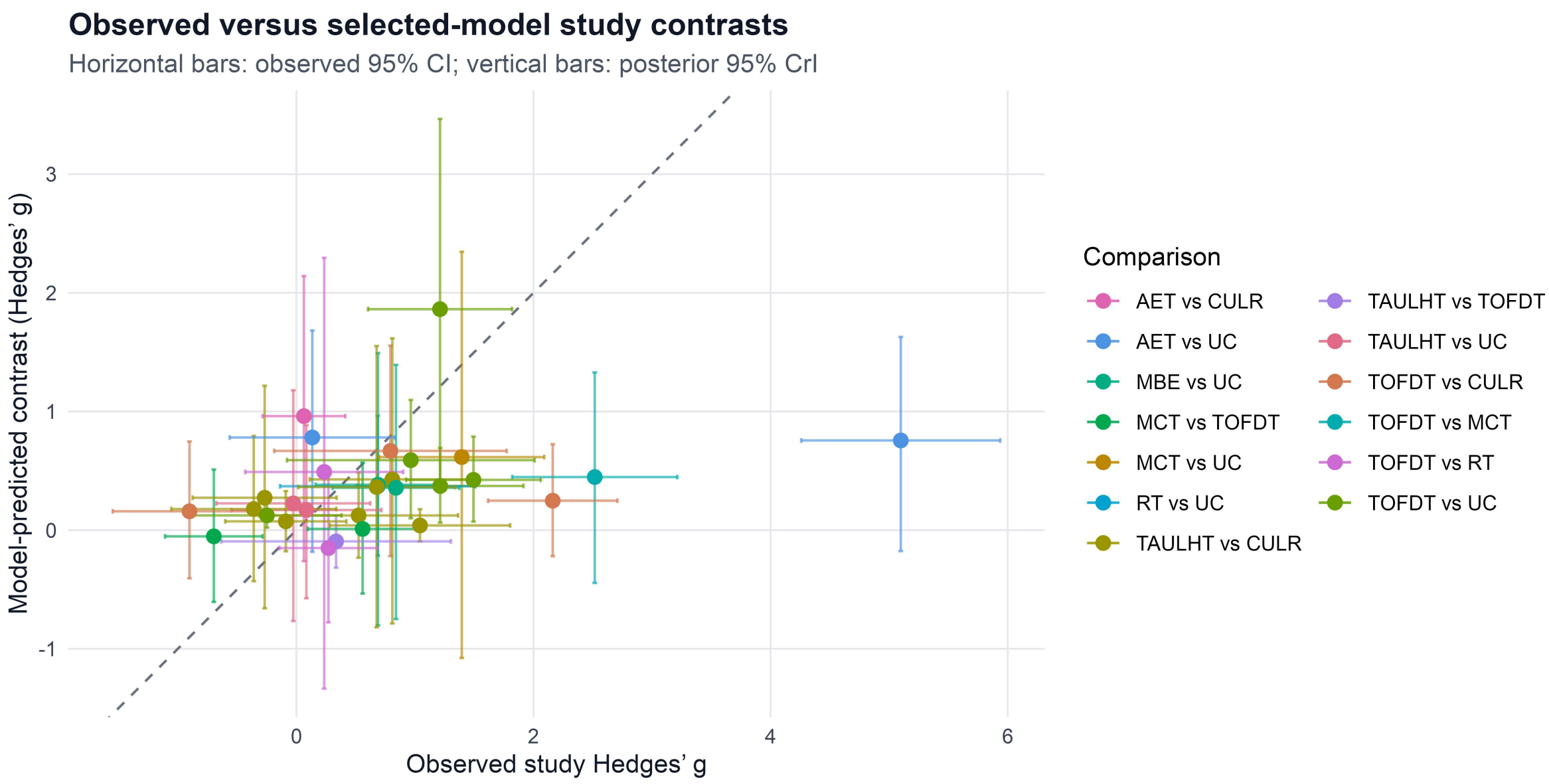

Supplementary Fig. 8.2B. Observed versus posterior-predicted contrast effects for finger dexterity.

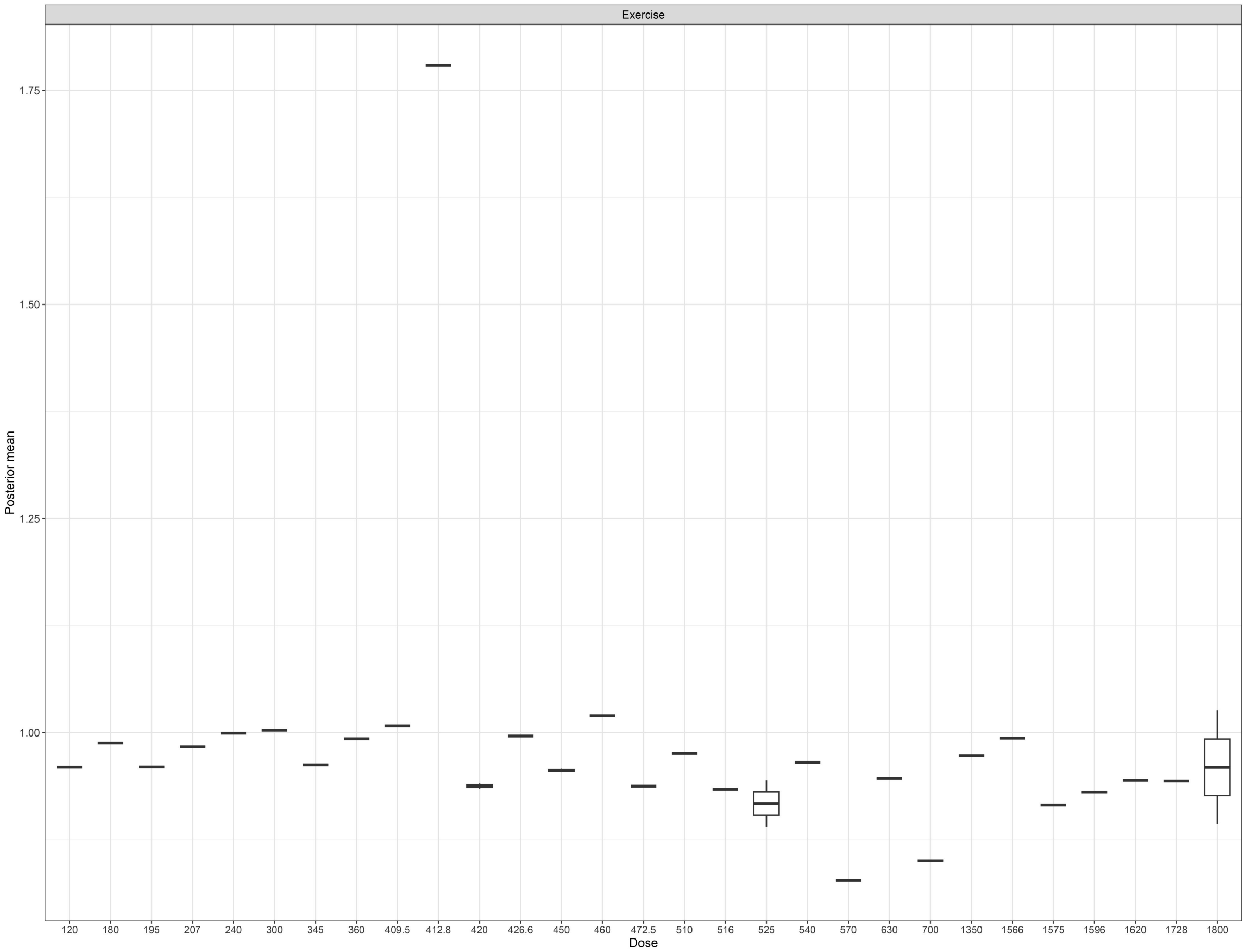

Supplementary Fig. 8.2C. Deviance plot for the overall-exercise dose–response model for finger dexterity.

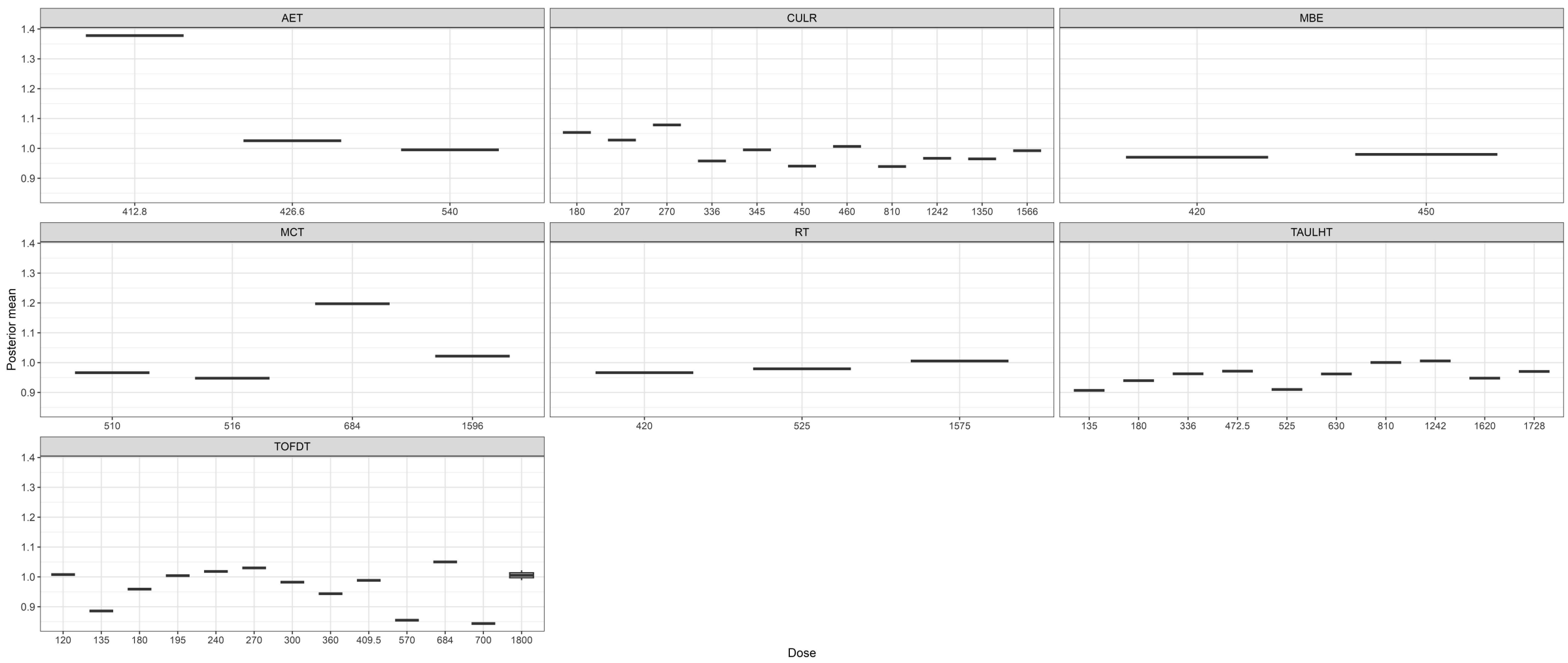

Supplementary Fig. 8.2D. Deviance plots for the modality-specific dose–response models for finger dexterity.

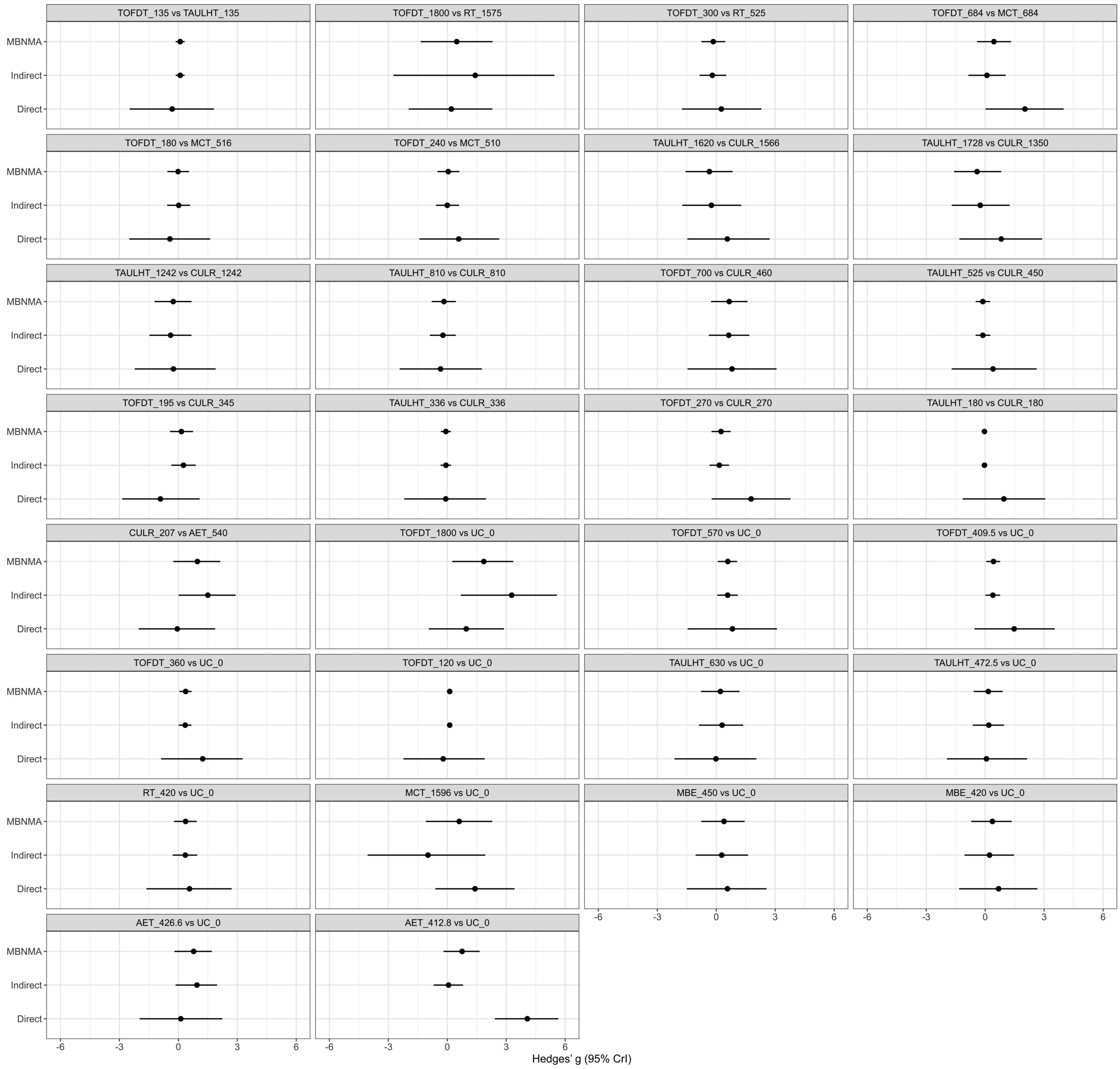

Supplementary Fig. 8.2E. Node-splitting analysis for finger dexterity: forest plots.

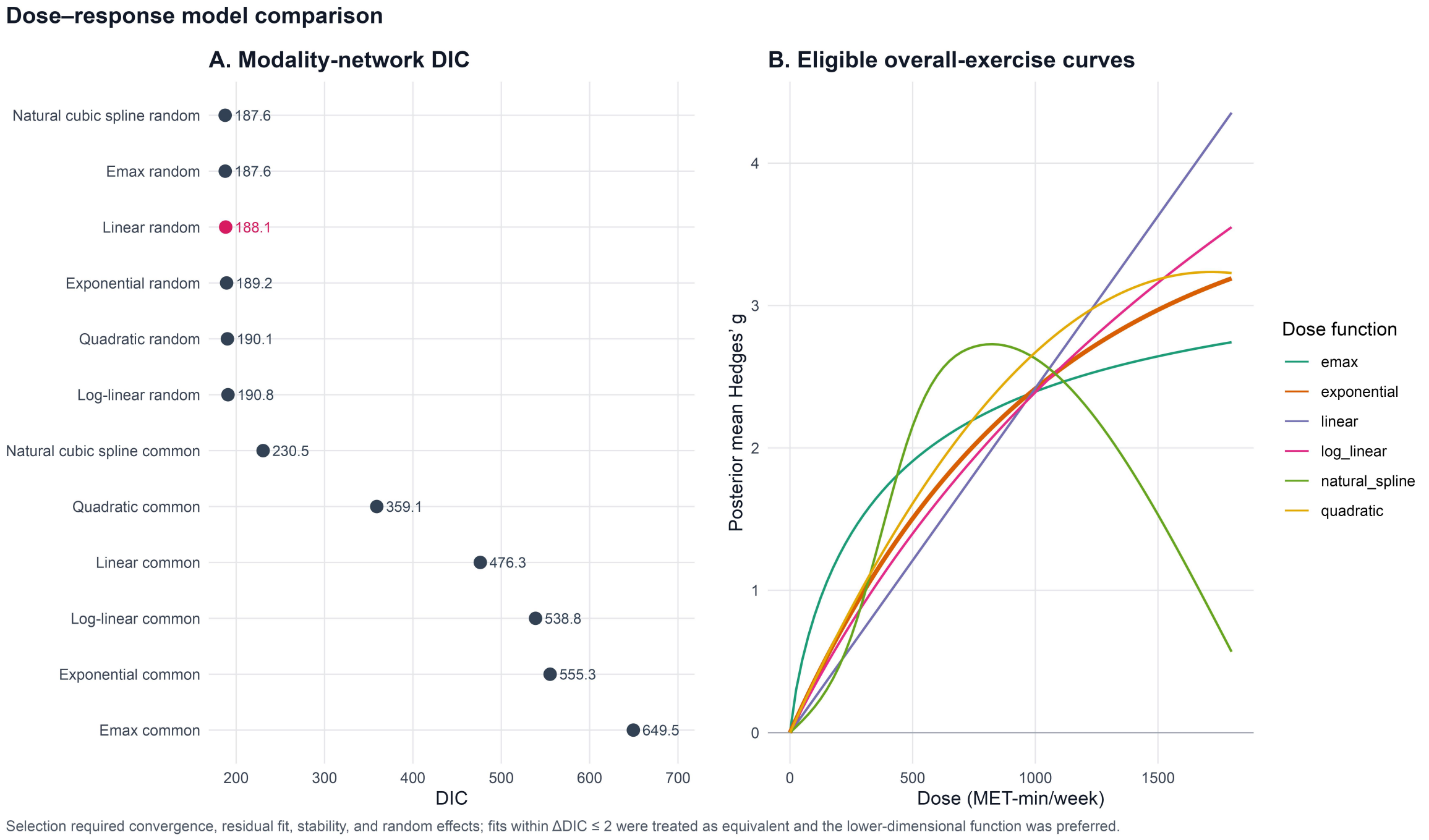

Supplementary Fig. 8.2F. Comparison of candidate modality-specific and overall-exercise dose–response models for upper-limb function.

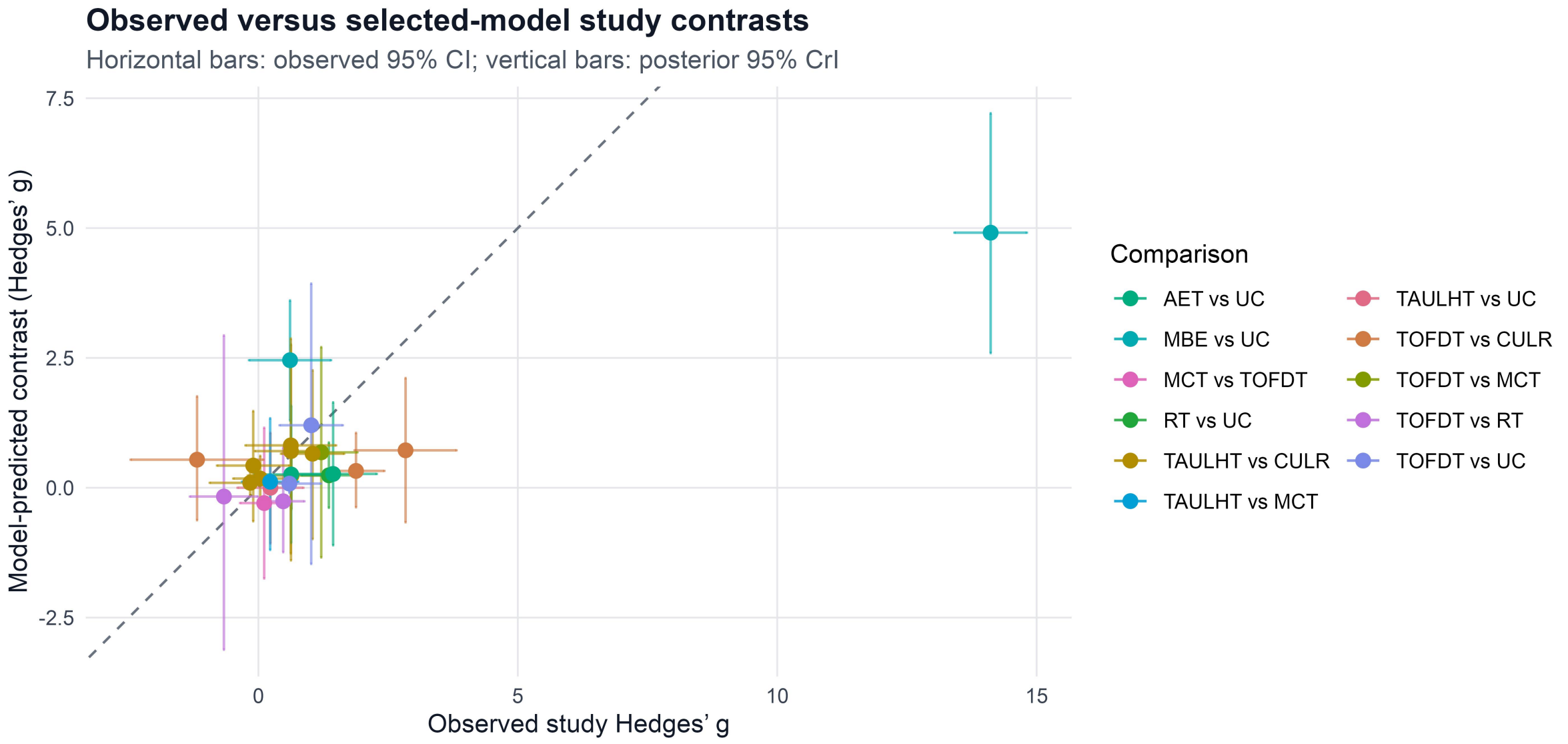

Supplementary Fig. 8.2G. Observed versus posterior-predicted contrast effects for upper-limb function.

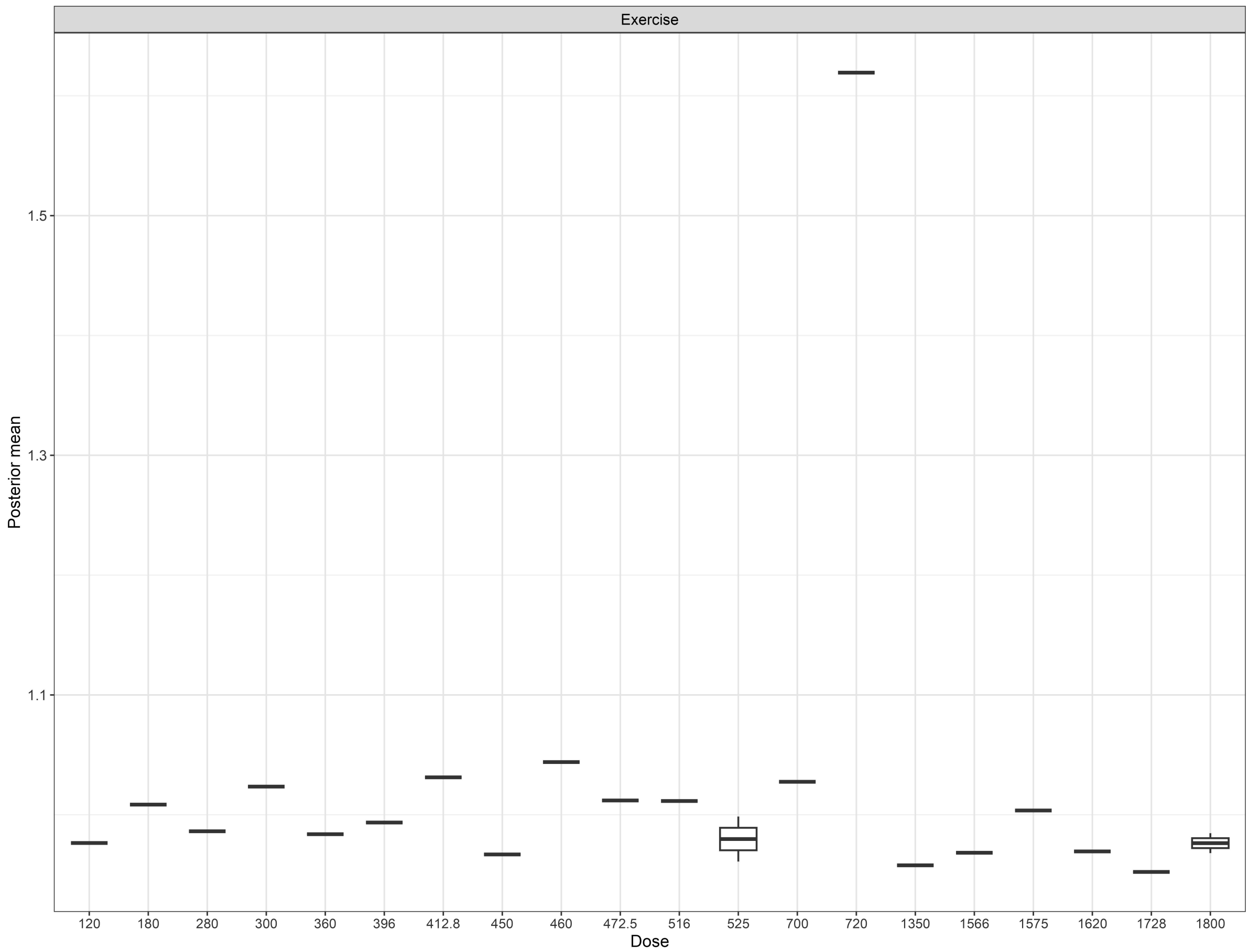

Supplementary Fig. 8.2H. Deviance plot for the overall-exercise dose–response model for upper-limb function.

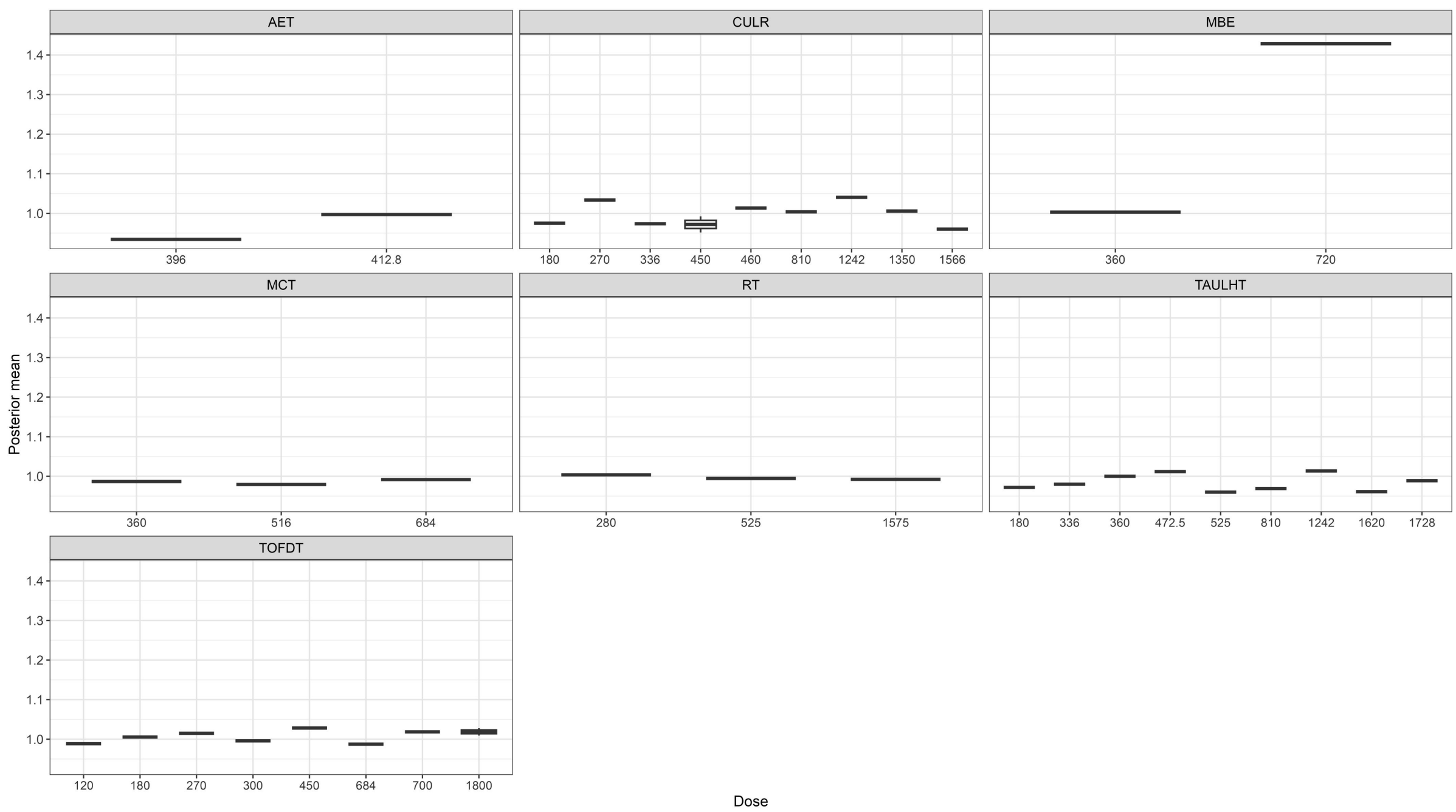

Supplementary Fig. 8.2I. Deviance plots for the modality-specific dose–response models for upper-limb function.

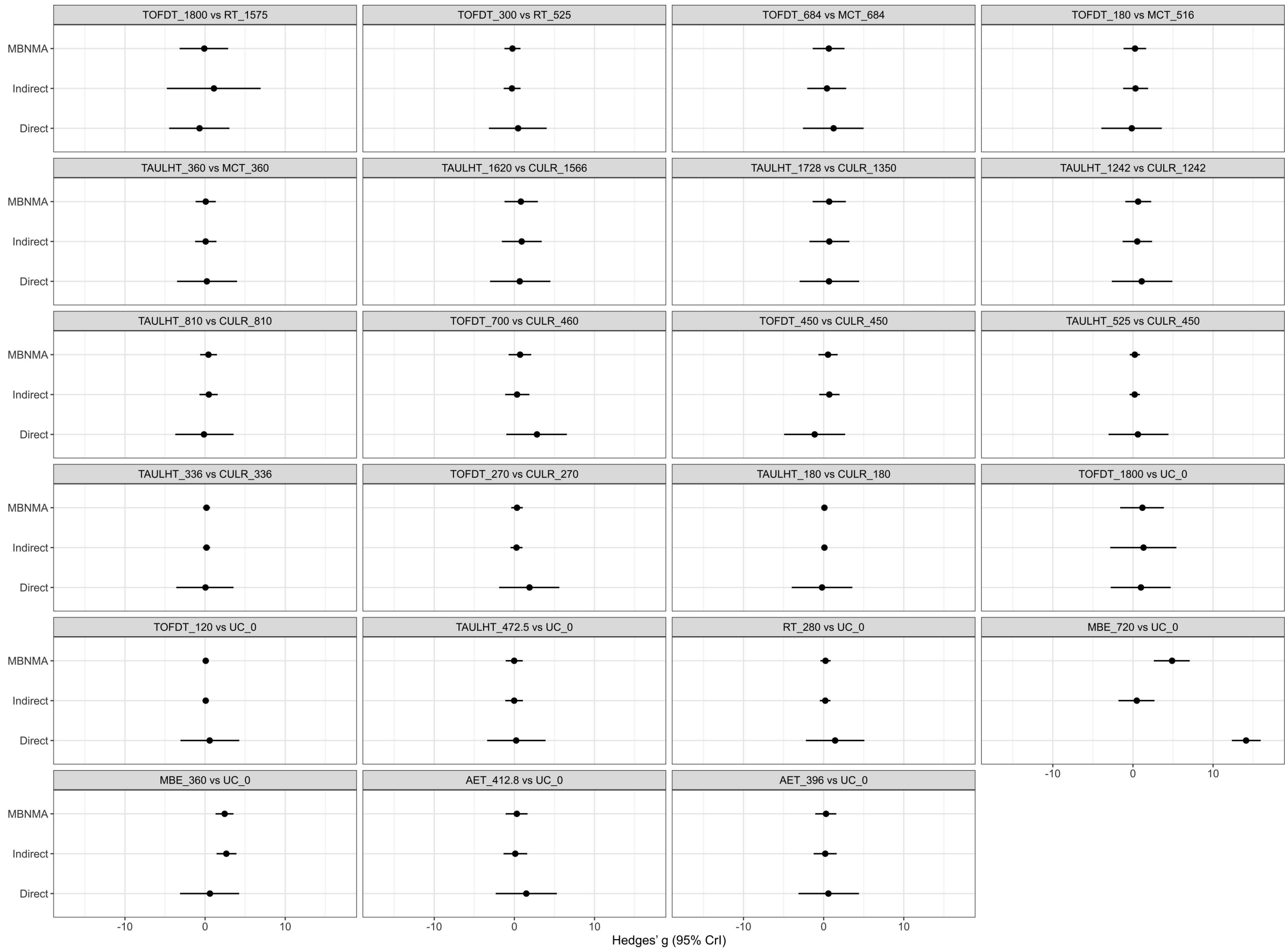

Supplementary Fig. 8.2J. Node-splitting analysis for upper-limb function: forest plots.

S8.3 Overall exercise dose–response associations

Supplementary Table 8.3A. Overall exercise dose–response model summary for upper-limb function and finger dexterity.

Outcome	Selected model	No. of studies	Distinct observed doses, n	Observed dose range, MET·min/week	Dose with largest predicted effect, MET·min/week	Near-maximum interval, MET·min/week	Boundary status
Finger dexterity	Linear random-effects	23	29	120–1,800	1,800	1,790–1,800	Maximum at upper boundary
Upper-limb function	Exponential random-effects	15	20	120–1,800	1,800	1,790–1,800	Maximum at upper boundary

Supplementary Table 8.3B. Predicted overall exercise effects at representative weekly doses for upper-limb function and finger dexterity.

Outcome	Evaluated dose, MET·min/week	Predicted Hedges' g, posterior median	95% CrI	P(g>0)
Finger dexterity	120	0.116	0.039–0.193	0.997
	600	0.578	0.195–0.965	0.997
	1,200	1.157	0.391–1.931	0.997
	1,800	1.735	0.586–2.896	0.997
Upper-limb function	120	0.432	0.165–0.700	>0.999
	600	1.722	0.658–2.795	>0.999
	1,200	2.667	1.020–4.329	>0.999
	1,800	3.186	1.218–5.171	>0.999

Note for Supplementary Tables 8.3A and 8.3B: The within-range maximum and near-maximum doses were constrained to the observed dose range; the near-maximum interval denotes doses with posterior mean effects within 0.01 Hedges' g of the largest predicted mean effect. "Maximum at upper boundary" indicates that the largest predicted effect occurred at the highest observed dose and should not be interpreted as an optimal prescription or a biological plateau. Effects are posterior median Hedges' g values with 95% credible intervals relative to the zero-dose reference (usual care or no structured exercise);positive values favour exercise, and $P(g > 0)$ denotes the posterior probability of benefit. CrI, credible interval; MET, metabolic equivalent of task.

S8.4 Modality-specific dose–response associations

Supplementary Table 8.4A. Modality-specific dose–response estimates for finger dexterity.

Exercise modality	No. of studies	Observed range	Mean-curve range maximum	Posterior range maximum (95% CrI)	Largest predicted effect (95% CrI)
AET	3	413–540	540	540 [413, 540]	0.99 [−0.23, 2.13]
CULR	11	180–1,566	1,566	1,566 [180, 1,566]	0.19 [−2.55, 2.79]
MBE	2	420–450	450	450 [420, 450]	0.38 [−0.80, 1.49]
MCT	4	510–1,596	1,596	1,596 [510, 1,596]	0.62 [−1.08, 2.35]
RT	3	420–1,575	1,575	1,575 [420, 1,575]	1.39 [−0.80, 3.62]
TAULHT	10	135–1,728	1,728	1,728 [135, 1,728]	0.62 [−2.10, 3.23]
TOFDT	14	120–1,800	1,800	1,800 [1,800, 1,800]	1.86 [0.32, 3.47]

Supplementary Table 8.4B. Modality-specific dose–response estimates for upper-limb function.

Exercise modality	No. of studies	Observed range	Mean-curve range maximum	Posterior range maximum (95% CrI)	Largest predicted effect (95% CrI)
AET	2	396–413	413	413 [396, 413]	0.27 [−1.11, 1.64]
CULR	10	180–1,566	180	180 [180, 1,566]	−0.09 [−0.54, 0.33]
MBE	2	360–720	720	720 [720, 720]	4.91 [2.60, 7.21]
MCT	3	360–684	360	360 [360, 684]	−0.13 [−1.16, 0.92]
RT	3	280–1,575	1,575	1575 [280, 1,575]	1.35 [−2.16, 4.89]
TAULHT	9	180–1,728	180	954 [180, 1,728]	0.00 [−0.41, 0.40]
TOFDT	9	120–1,800	1,800	1,800 [120, 1,800]	1.20 [−1.47, 3.93]

Note: Doses are expressed in MET-min/week, and effects are expressed as Hedges' g. The mean-curve range maximum denotes the dose associated with the largest posterior mean effect within the observed dose range. The posterior range maximum was determined separately for each posterior draw and is reported as the median [95% credible interval]; it should not be interpreted as an optimal dose. Largest predicted effects are posterior medians [95% credible intervals] evaluated at the mean-curve range-maximum dose. All dose searches were restricted to the observed dose range; boundary estimates should be interpreted cautiously. CrI, credible interval; MET, metabolic equivalent of task.

S8.5 Dose-specific treatment rankings

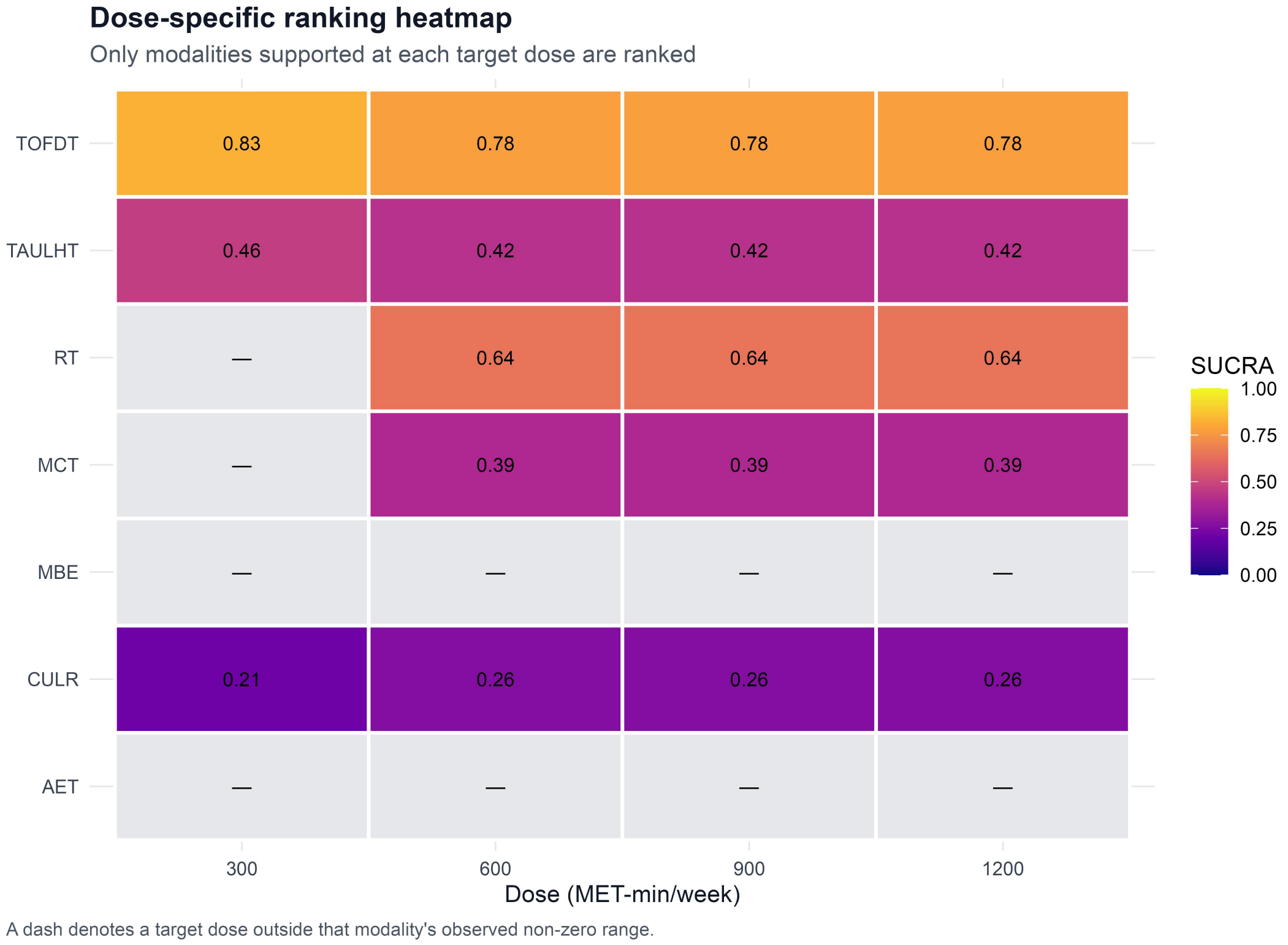

Supplementary Fig. 8.5A. Dose-specific ranking heatmap for finger dexterity.

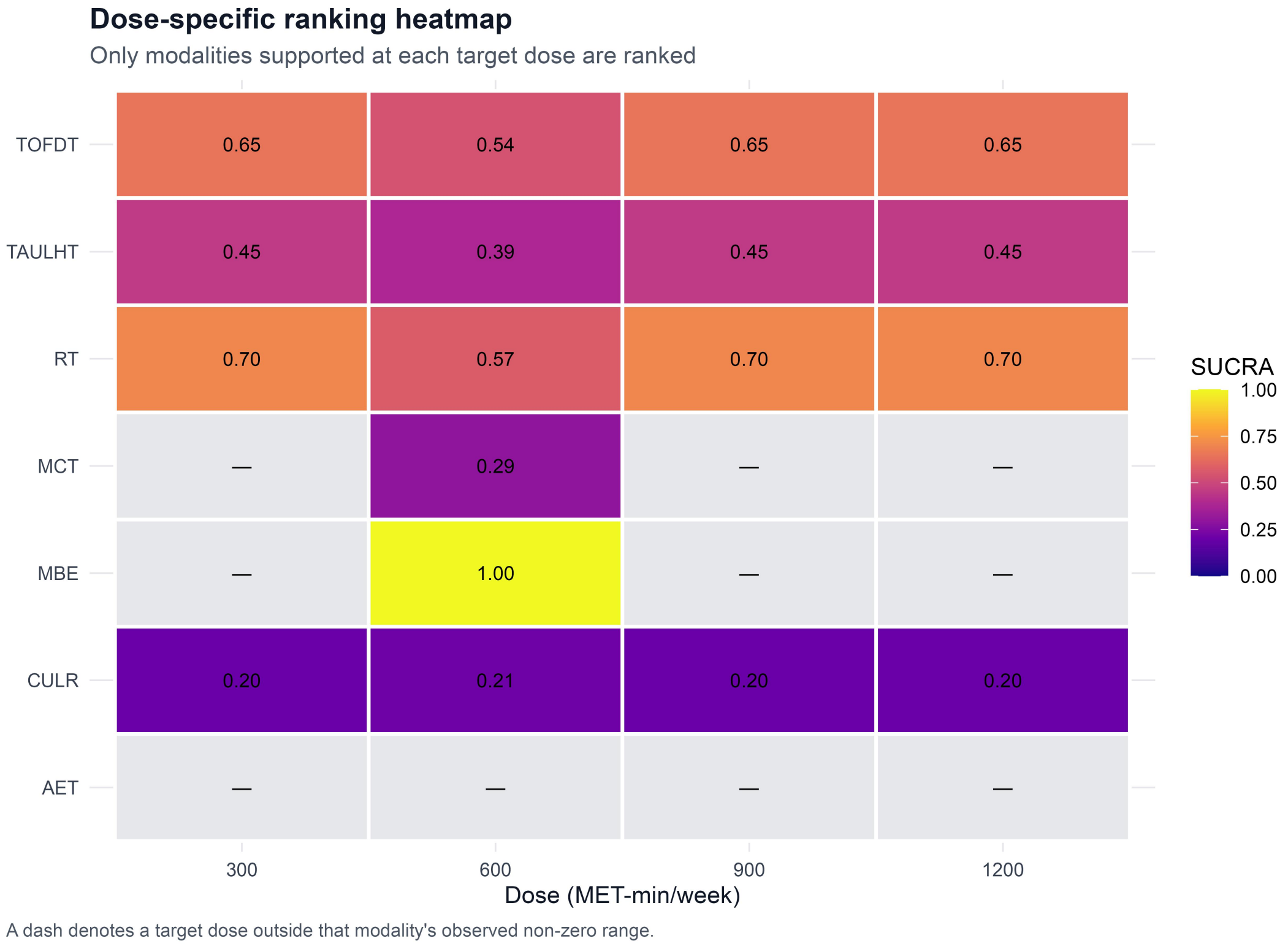

Supplementary Fig. 8.5B. Dose-specific ranking heatmap for upper-limb function.

Note: Heatmaps show the surface under the cumulative ranking curve (SUCRA) at representative weekly doses

for (A) finger dexterity and (B) upper-limb function. Rankings were restricted to modalities whose observed non-zero dose ranges included the target dose; dashes indicate unsupported doses. Because the eligible set of modalities varied across doses, SUCRA values should not be compared directly between dose columns. Rankings are descriptive and do not identify an optimal or recommended dose.

S8.6 CINeMA assessment of certainty of evidence in the Bayesian dose–response network meta-analysis

Supplementary Table 8.6A. Certainty of evidence for prespecified dose-specific comparisons in the Bayesian dose–response network meta-analysis for finger dexterity.

Comparison	Number of studies	Observed dose range (MET·min/week)	MBNMA effect, Hedges' g (95% CrI)	Within-study bias	Reporting bias	Indirectness	Imprecision	Heterogeneity	Incoherence	Confidence rating
Overall exercise 180 vs UC 0	23	120–1800	0.17 (0.06 to 0.29)	No concerns	No concerns	Some concerns	No concerns	Some concerns	No concerns	Low
CULR 180 vs UC 0	11	180–1566	0.02 (-0.29 to 0.32)	Some concerns	No concerns	No concerns	Some concerns	Some concerns	No concerns	Very low
TAULHT 180 vs UC 0	10	135–1728	0.06 (-0.22 to 0.34)	Some concerns	No concerns	Some concerns	No concerns	No concerns	Some concerns	Very low
TOFDT 180 vs UC 0	14	120–1800	0.19 (0.03 to 0.35)	No concerns	No concerns	No concerns	Some concerns	No concerns	No concerns	Moderate
Overall exercise 450 vs UC 0	23	120–1800	0.43 (0.15 to 0.72)	No concerns	No concerns	No concerns	No concerns	No concerns	Some concerns	Moderate
AET 450 vs UC 0	3	413–540	0.82 (-0.19 to 1.78)	No concerns	No concerns	No concerns	No concerns	Some concerns	No concerns	Moderate
CULR 450 vs UC 0	11	180–1566	0.05 (-0.73 to 0.80)	Some concerns	No concerns	No concerns	Major concerns	Some concerns	No concerns	Very low
MBE 450 vs UC 0	2	420–450	0.38 (-0.80 to 1.49)	Some concerns	No concerns	Some concerns	No concerns	Major concerns	No concerns	Very low
RT 450 vs UC 0	3	420–1575	0.40 (-0.23 to 1.03)	No concerns	No concerns	No concerns	Some concerns	No concerns	Some concerns	Low
TAULHT 450 vs UC 0	10	135–1728	0.16 (-0.55 to 0.84)	Some concerns	No concerns	No concerns	Some concerns	Major concerns	No concerns	Very low
TOFDT 450 vs UC 0	14	120–1800	0.47 (0.08 to 0.87)	No concerns	No concerns	Some concerns	Some concerns	No concerns	No concerns	Low
Overall exercise 700 vs UC 0	23	120–1800	0.67 (0.23 to 1.13)	Some concerns	No concerns	No concerns	No concerns	No concerns	No concerns	Moderate
CULR 700 vs UC 0	11	180–1566	0.09 (-1.14 to 1.25)	Some concerns	No concerns	No concerns	No concerns	Some concerns	Some concerns	Very low
MCT 700 vs UC 0	4	510–1596	0.27 (-0.47 to 1.03)	No concerns	No concerns	No concerns	Some concerns	Some concerns	Some concerns	Very low
RT 700 vs UC 0	3	420–1575	0.62 (-0.36 to 1.61)	Some concerns	No concerns	Some concerns	Some concerns	No concerns	No concerns	Very low
TAULHT 700 vs UC 0	10	135–1728	0.25 (-0.85 to 1.31)	No concerns	No concerns	No concerns	Some concerns	Some concerns	Some concerns	Very low
TOFDT 700 vs UC 0	14	120–1800	0.72 (0.12 to 1.35)	No concerns	No concerns	No concerns	No concerns	Some concerns	No concerns	Moderate
Overall exercise 1600 vs UC 0	23	120–1800	1.54 (0.52 to 2.57)	No concerns	No concerns	Some concerns	No concerns	No concerns	No concerns	Moderate
TAULHT 1600 vs UC 0	10	135–1728	0.57 (-1.94 to 2.99)	Some concerns	No concerns	No concerns	Some concerns	No concerns	Some concerns	Very low
TOFDT 1600 vs UC 0	14	120–1800	1.66 (0.28 to 3.08)	Some concerns	No concerns	No concerns	No concerns	Some concerns	Some concerns	Very low

Supplementary Table 8.6B. Certainty of evidence for prespecified dose-specific comparisons in the Bayesian dose–response network meta-analysis for upper-limb function.

Comparison	Number of studies	Observed dose range (MET·min/week)	MBNMA effect, Hedges' g (95% CrI)	Within-study bias	Reporting bias	Indirectness	Imprecision	Heterogeneity	Incoherence	Confidence rating
Overall exercise 180 vs UC 0	15	120–1800	0.63 (0.24 to 1.02)	No concerns	No concerns	Some concerns	No concerns	No concerns	No concerns	Moderate
CULR 180 vs UC 0	10	180–1566	-0.09 (-0.54 to 0.33)	Some concerns	No concerns	Some concerns	Major concerns	No concerns	Some concerns	Very low
TAULHT 180 vs UC 0	9	180–1728	0.00 (-0.41 to 0.40)	Some concerns	No concerns	No concerns	Some concerns	Some concerns	Some concerns	Very low
TOFDT 180 vs UC 0	9	120–1800	0.12 (-0.15 to 0.39)	Some concerns	No concerns	Some concerns	No concerns	Some concerns	No concerns	Very low
Overall exercise 450 vs UC 0	15	120–1800	1.38 (0.53 to 2.24)	No concerns	No concerns	No concerns	No concerns	Some concerns	No concerns	Moderate
CULR 450 vs UC 0	10	180–1566	-0.23 (-1.36 to 0.83)	Some concerns	No concerns	No concerns	Major concerns	No concerns	Some concerns	Very low
MBE 450 vs UC 0	2	360–720	3.07 (1.62 to 4.51)	No concerns	No concerns	Some concerns	No concerns	No concerns	No concerns	Moderate
MCT 450 vs UC 0	3	360–684	-0.16 (-1.45 to 1.16)	Some concerns	No concerns	No concerns	Some concerns	Some concerns	No concerns	Very low
RT 450 vs UC 0	3	280–1575	0.39 (-0.62 to 1.40)	No concerns	No concerns	Some concerns	No concerns	Some concerns	Some concerns	Very low
TAULHT 450 vs UC 0	9	180–1728	0.00 (-1.02 to 1.00)	Some concerns	No concerns	No concerns	Major concerns	Some concerns	No concerns	Very low
TOFDT 450 vs UC 0	9	120–1800	0.30 (-0.37 to 0.98)	Some concerns	No concerns	No concerns	No concerns	No concerns	Some concerns	Low
Overall exercise 700 vs UC 0	15	120–1800	1.92 (0.73 to 3.12)	Some concerns	No concerns	No concerns	No concerns	No concerns	No concerns	Moderate
CULR 700 vs UC 0	10	180–1566	-0.36 (-2.12 to 1.30)	No concerns	No concerns	No concerns	Some concerns	Major concerns	Some concerns	Very low
MBE 700 vs UC 0	2	360–720	4.78 (2.52 to 7.01)	No concerns	No concerns	Some concerns	No concerns	No concerns	No concerns	Moderate

Comparison	Number of studies	Observed dose range (MET·min/week)	MBNMA effect, Hedges' g (95% CrI)	Within-study bias	Reporting bias	Indirectness	Imprecision	Heterogeneity	Incoherence	Confidence rating
RT 700 vs UC 0	3	280–1575	0.60 (-0.96 to 2.17)	Some concerns	No concerns	No concerns	Some concerns	No concerns	Some concerns	Very low
TAULHT 700 vs UC 0	9	180–1728	0.00 (-1.59 to 1.56)	No concerns	No concerns	No concerns	Some concerns	Some concerns	Some concerns	Very low
TOFDT 700 vs UC 0	9	120–1800	0.47 (-0.57 to 1.53)	Some concerns	No concerns	No concerns	No concerns	Major concerns	No concerns	Very low
Overall exercise 1575 vs UC 0	15	120–1800	3.03 (1.16 to 4.91)	Some concerns	No concerns	No concerns	No concerns	Some concerns	No concerns	Low
RT 1575 vs UC 0	3	280–1575	1.35 (-2.16 to 4.89)	No concerns	No concerns	Some concerns	No concerns	Some concerns	No concerns	Low
TAULHT 1575 vs UC 0	9	180–1728	0.00 (-3.57 to 3.52)	Some concerns	No concerns	No concerns	Some concerns	Some concerns	Some concerns	Very low
TOFDT 1575 vs UC 0	9	120–1800	1.05 (-1.28 to 3.44)	Some concerns	No concerns	No concerns	No concerns	Some concerns	Some concerns	Very low

Note: Effects are posterior median Hedges' g estimates with 95% credible intervals (CrIs) relative to usual care (UC) at dose 0; positive values favour exercise. Prespecified dose anchors were 180, 450, 700, and 1,600 MET·min/week for finger dexterity and 180, 450, 700, and 1,575 MET·min/week for upper-limb function. "Number of studies" denotes studies contributing to the corresponding continuous dose–response function, not studies conducted at the exact target dose. Only estimates within the observed intervention-specific dose range were assessed. Certainty of evidence was assessed using CINeMA across the six domains shown and rated as high, moderate, low, or very low. Heterogeneity judgements incorporated the model-specific τ and approximate 95% prediction interval. The Hedges' g thresholds of −0.20 and +0.20 were exploratory and do not represent validated minimal clinically important differences or recommended doses. MBNMA, model-based network meta-analysis; CINeMA, Confidence in Network Meta-Analysis; CrI, credible interval; MET, metabolic equivalent of task; AET, aerobic exercise; CULR, conventional upper-limb rehabilitation; MBE, mind–body exercise; MCT, multicomponent training; RT, resistance training; TAULHT, technology-assisted upper-limb or hand training; TOFDT, task-oriented finger dexterity training; UC, usual care or no structured exercise.

S8.7 Exploratory dose thresholds and data-supported effective ranges

Supplementary Table 8.7. Exploratory Hedges' g threshold of 0.20 for threshold-attaining overall and modality-specific exercise curves.

Outcome	Curve	Minimum statistically supported dose, MET·min/week	Minimum dose reaching Hedges' g 0.20, MET·min/week	Predicted Hedges' g at threshold, posterior median (95% CrI)	P(g > 0)	P(g ≥ 0.20)	Threshold-supported dose range, MET·min/week
Finger dexterity	Overall exercise	120	207	0.200 (0.067–0.333)	0.997	0.496	207–1,800
	TOFDT	120	195	0.202 (0.034–0.375)	0.991	0.510	195–1,800
Upper-limb function	Overall exercise	120	120	0.432 (0.165–0.700)	>0.999	0.955	120–1,800
	MBE	360	360	2.456 (1.298–3.604)	>0.999	>0.999	360–720

Note: Only curves reaching the exploratory Hedges' g threshold of 0.20 threshold within their observed dose ranges are shown. The minimum statistically supported dose was the lowest data-supported dose for which the lower 95% CrI exceeded zero. The Hedges' g threshold of 0.20 was determined using the posterior mean, whereas effect estimates are reported as posterior medians with 95% CrIs. P(g > 0) and P(g ≥ 0.20) denote the posterior probabilities of benefit and of reaching the exploratory threshold, respectively. The remaining modality-specific curves did not meet both criteria within their observed dose ranges. A Hedges' g of 0.20 should not be interpreted as a validated minimal clinically important difference or a recommended exercise dose. CrI, credible interval; MBE, mind–body exercise; MET, metabolic equivalent of task; TOFDT, task-oriented finger dexterity training.

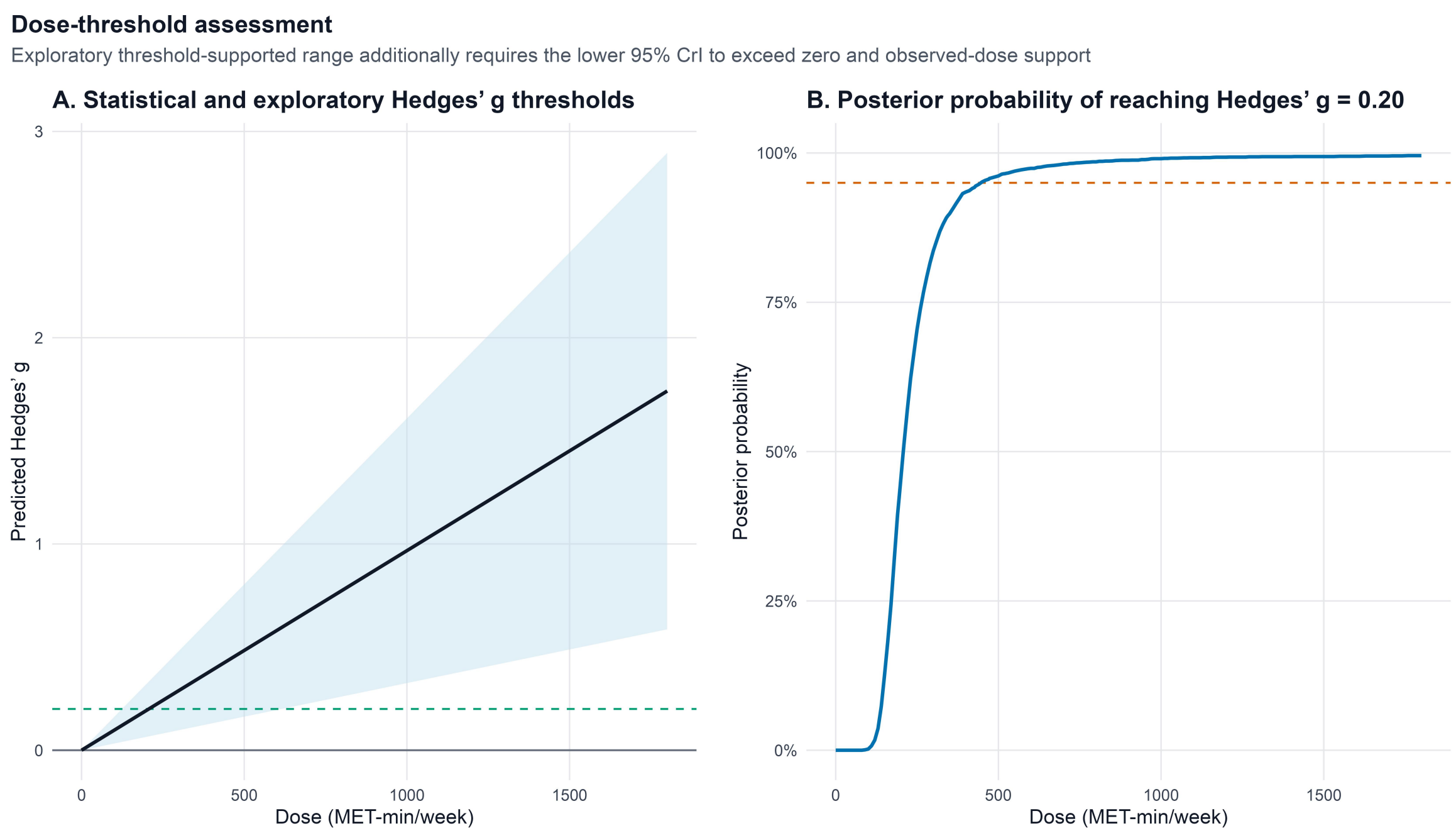

Supplementary Fig. 8.7A. Overall exercise dose-threshold assessment for finger dexterity. Solid line and shading in panel A show the posterior median Hedges' g and 95% CrI.

Horizontal lines mark no effect and the exploratory Hedges' g threshold of 0.20. Panel B shows Pr(g ≥ 0.20); the dashed line marks 0.95. Hedges' g threshold of 0.20 is not a validated MCID or recommended dose. CrI, credible interval; MCID, minimal clinically important difference.

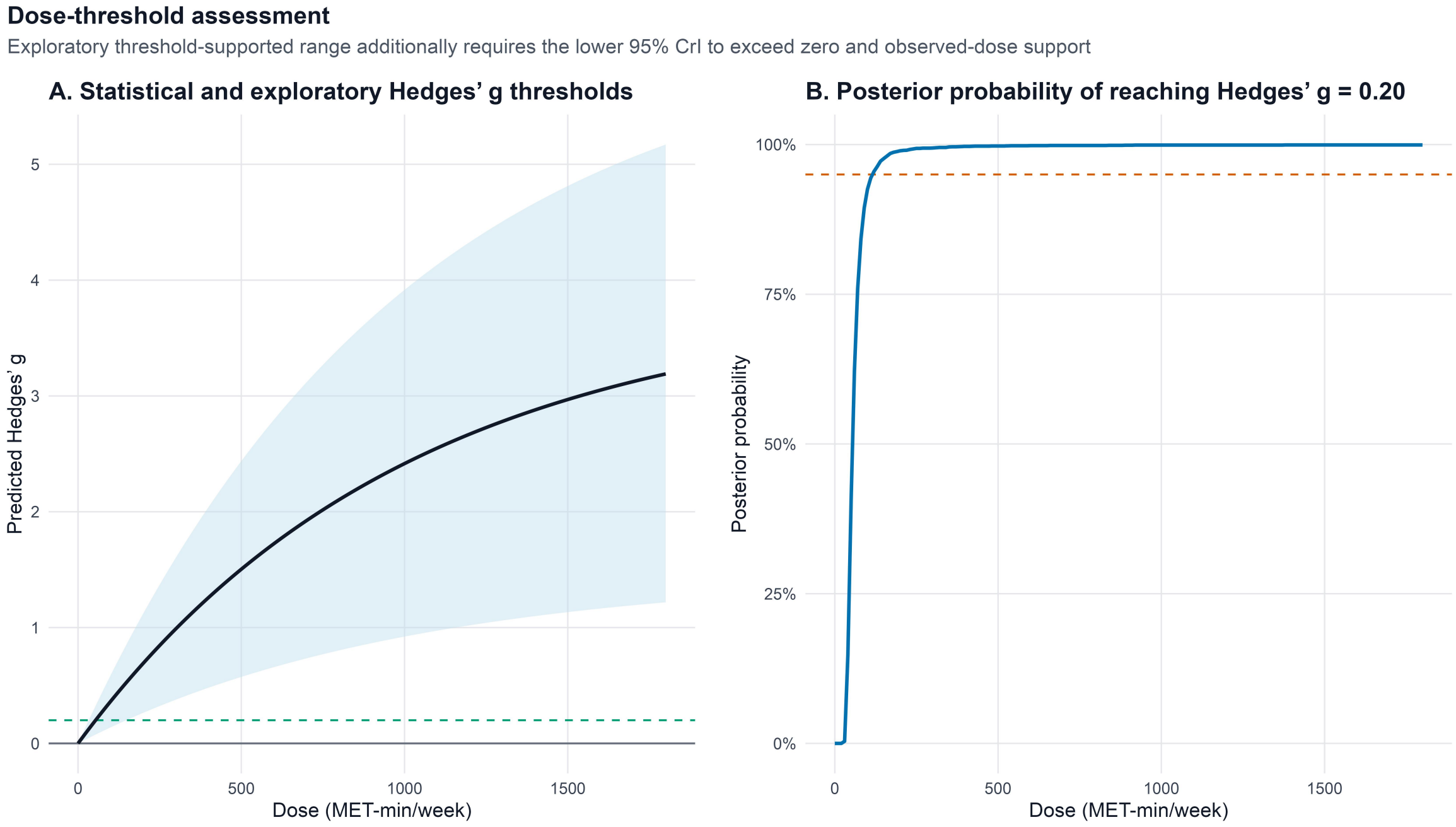

Supplementary Fig. 8.7B. Overall exercise dose-threshold assessment for upper-limb function. Solid line and shading in panel A show the posterior median Hedges' g and 95% CrI. Horizontal lines mark no effect and the exploratory Hedges' g threshold of 0.20. Panel B shows Pr(g ≥ 0.20); the dashed line marks 0.95. Hedges' g threshold of 0.20 is not a validated MCID or recommended dose. CrI, credible interval; MCID, minimal clinically important difference.

S8.8 Exploratory dose-by-subgroup interaction analyses

Supplementary Table 8.8A. Bayesian dose-by-subgroup interaction estimates for finger dexterity.

Subgroup dimension	Contrast	Studies, n (reference/comparison)	Common supported dose range, MET·min/week	Interaction estimate, β (95% CrI)	Pr (β>0)
Intervention duration	≥8 vs <8 weeks	10/13	195–1620	0.26 (−0.99 to 1.49)	0.666
Mean age	≥65 vs <65 years	10/13	180–1728	0.80 (−0.37 to 2.01)	0.914
Outcome assessment type	Non-pegboard vs pegboard	18/5	180–516	2.84 (0.99 to 4.48)	0.998

Note: Values are posterior medians (95% CrIs). Study counts are reported as reference/comparison subgroups. The common supported dose range represents the overlap in observed doses between subgroups. β denotes the comparison-minus-reference difference in the linear dose–response slope, expressed as Hedges' g per 1,000 MET·min/week; positive values indicate a greater dose-related benefit in the comparison subgroup. Pr(β > 0) is the posterior probability of a positive interaction. The assessment-type analysis is exploratory because the non-pegboard subgroup included only five studies and had a restricted common dose range. CrI, credible interval; MET, metabolic equivalent of task.

Supplementary Table 8.8B. Bayesian dose-by-subgroup interaction estimates for upper-limb function.

Subgroup dimension	Contrast	Studies, n (reference/comparison)	Common supported dose range, MET·min/week	Interaction estimate, β (95% CrI)	Pr (β>0)
Intervention duration	≥8 vs <8 weeks	5/10	300–1620	3.56 (0.42 to 6.66)	0.987
Mean age	≥65 vs <65 years	6/9	180–1728	−0.37 (−3.68 to 2.88)	0.416
Outcome assessment type	Patient/goal-reported vs objective/clinician-assessed	11/4	180–1728	0.15 (−3.71 to 3.93)	0.530

Note: Values are posterior medians (95% CrIs). Study counts are reported as reference/comparison subgroups. The common supported dose range represents the overlap in observed doses between subgroups. β denotes the comparison-minus-reference difference in the exponential dose–response amplitude parameter; positive values indicate a greater dose-related benefit in the comparison subgroup. Pr(β > 0) is the posterior probability of a positive interaction. All analyses are exploratory; estimates should be interpreted cautiously because several subgroups included only 4–6 studies and the posterior for the between-study SD (τ) approached the upper limit of the prespecified Uniform(0, 2) prior. CrI, credible interval; MET, metabolic equivalent of task.

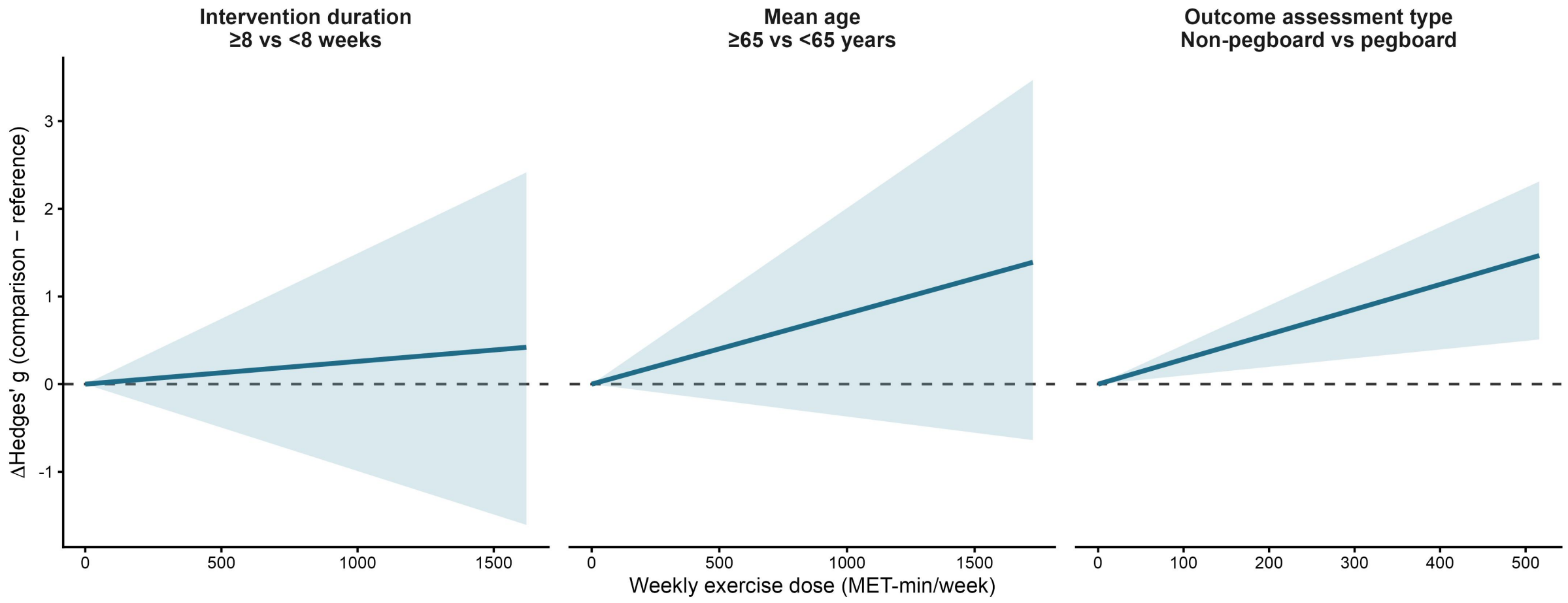

Supplementary Fig. 8.8A. Dose-by-subgroup interaction curves for finger dexterity. Solid lines and shading show posterior median comparison-minus-reference differences and 95% CrIs. Dashed lines denote no interaction; positive values indicate a greater dose-related benefit in the comparison subgroup. Curves are anchored at zero dose and extend to the upper limit of the common observed dose range; inference is restricted to the common non-zero dose range reported in Supplementary Table 8.8A. CrI, credible interval; MET, metabolic equivalent of task.

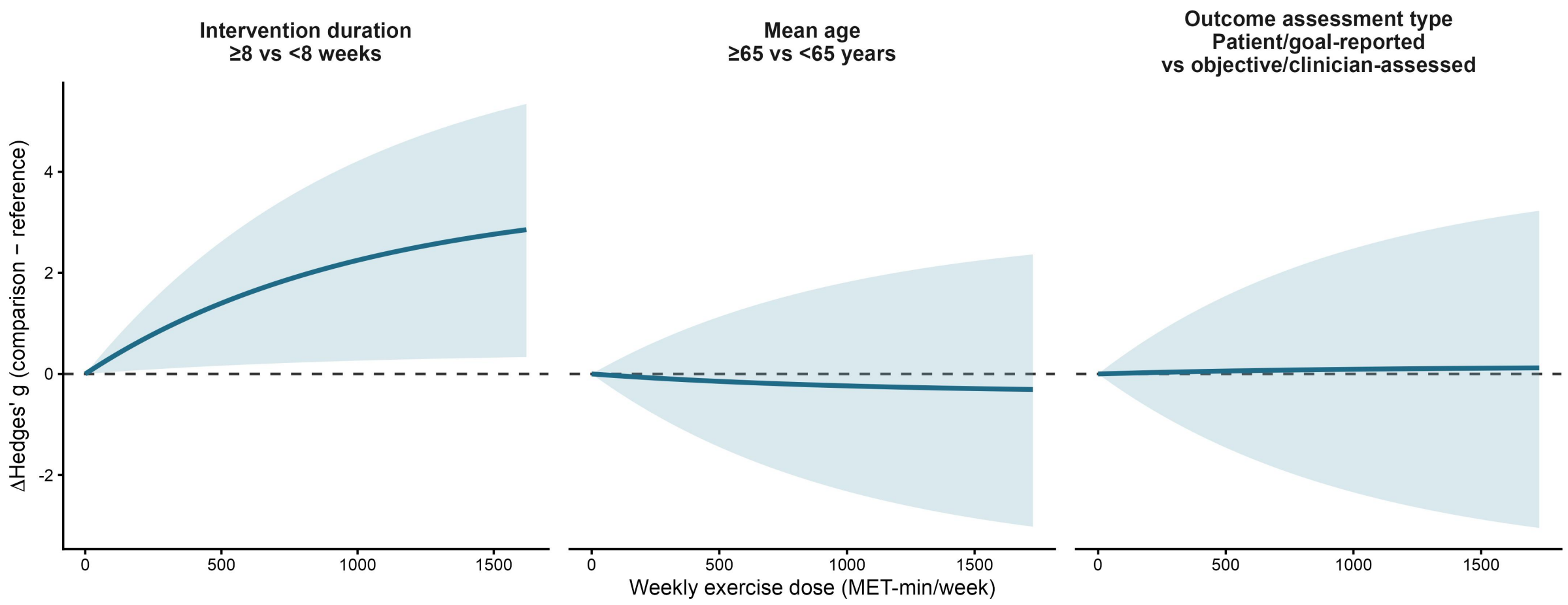

Supplementary Fig. 8.8B. Dose-by-subgroup interaction curves for upper-limb function. Solid lines and shading show posterior median comparison-minus-reference differences and 95% CrIs. Dashed lines denote no interaction; positive values indicate a greater dose-related benefit in the comparison subgroup. Curves are anchored at zero dose and extend to the upper limit of the common observed dose range; inference is restricted to the common non-zero dose range reported in Supplementary Table 8.8B. CrI, credible interval; MET, metabolic equivalent of task.

S8.9 Sensitivity analyses of the dose–response network meta-analysis

Supplementary Table 8.9A. Model fit and heterogeneity across sensitivity analyses of the dose–response network meta-analysis for finger dexterity and upper-limb function.

Outcome	Analysis level	Sensitivity analysis	Studies	DIC	Residual deviance	τ (95% CrI)
Finger dexterity	Overall exercise	Primary analysis	23	38.61	47.24	0.866 (0.598 to 1.294)
	Overall exercise	R = 0.50	23	38.05	47.33	0.551 (0.347 to 0.858)
	Overall exercise	R = 0.90	23	38.35	47.22	1.087 (0.775 to 1.596)
	Overall exercise	Excluding high-risk studies	19	31.15	38.93	0.975 (0.661 to 1.512)
	Overall exercise	Excluding large studies (>90th percentile)	20	37.18	40.90	0.921 (0.619 to 1.398)
	Exercise type-specific	Primary analysis	30	211.93	60.72	0.965 (0.694 to 1.365)
	Exercise type-specific	R = 0.50	30	250.63	60.41	0.618 (0.419 to 0.917)
	Exercise type-specific	R = 0.90	30	194.68	60.27	1.163 (0.870 to 1.634)
	Exercise type-specific	Excluding high-risk studies	26	182.70	52.24	1.053 (0.769 to 1.539)
	Exercise type-specific	Excluding large studies (>90th percentile)	27	193.43	54.19	0.956 (0.674 to 1.408)
Upper-limb function	Overall exercise	Primary analysis	15	27.84	30.82	1.951 (1.777 to 1.998)
	Overall exercise	R = 0.50	15	27.39	30.48	1.897 (1.595 to 1.996)
	Overall exercise	R = 0.90	15	32.92	32.38	1.982 (1.913 to 1.999)
	Overall exercise	Excluding high-risk studies	13	25.49	26.91	1.953 (1.785 to 1.998)
	Overall exercise	Excluding large studies (>90th percentile)	13	28.70	26.98	1.954 (1.792 to 1.999)
	Exercise type-specific	Primary analysis	23	189.06	46.51	1.887 (1.543 to 1.995)
	Exercise type-specific	R = 0.50	23	206.01	46.05	1.648 (1.137 to 1.982)
	Exercise type-specific	R = 0.90	23	164.10	47.47	1.966 (1.833 to 1.999)
	Exercise type-specific	Excluding high-risk studies	21	165.03	42.58	1.897 (1.590 to 1.996)
	Exercise type-specific	Excluding large studies (>90th percentile)	20	159.55	40.58	1.901 (1.578 to 1.996)

Note: All analyses used random-effects models with the prespecified Uniform(0, 2) prior for the between-study standard deviation (τ). Linear dose–response functions were used for finger dexterity at both analysis levels. For upper-limb function, the overall-exercise model was exponential, whereas the exercise type-specific model was linear. All models met the prespecified convergence criteria (Rhat ≤ 1.05 and ESS ≥ 200). R denotes the assumed pre–post correlation used to derive change-score standard deviations. Large studies were defined by an outcome-specific total sample size above the 90th percentile. Estimates of τ for upper-limb function approached the upper prior boundary and should be interpreted cautiously. CrI, credible interval; DIC, deviance information criterion; ESS, effective sample size.

Supplementary Table 8.9B. Overall exercise sensitivity estimates for finger dexterity.

Sensitivity analysis	Weekly dose (MET·min/week)	Hedges' g (95% CrI)	P(g > 0)
Primary analysis	120	0.115 (0.039 to 0.194)	0.998
Primary analysis	600	0.576 (0.195 to 0.968)	0.998
Primary analysis	1,200	1.152 (0.391 to 1.936)	0.998
Primary analysis	1,800	1.728 (0.586 to 2.904)	0.998
R = 0.50	120	0.088 (0.033 to 0.143)	0.999
R = 0.50	600	0.442 (0.167 to 0.717)	0.999
R = 0.50	1,200	0.884 (0.334 to 1.434)	0.999
R = 0.50	1,800	1.325 (0.501 to 2.151)	0.999
R = 0.90	120	0.131 (0.035 to 0.225)	0.995
R = 0.90	600	0.654 (0.177 to 1.124)	0.995
R = 0.90	1,200	1.308 (0.354 to 2.248)	0.995
R = 0.90	1,800	1.962 (0.530 to 3.372)	0.995
Excluding high-risk studies	120	0.111 (0.024 to 0.201)	0.992
Excluding high-risk studies	600	0.556 (0.120 to 1.004)	0.992
Excluding high-risk studies	1,200	1.111 (0.241 to 2.007)	0.992
Excluding high-risk studies	1,800	1.667 (0.361 to 3.011)	0.992
Excluding large studies (>90th percentile)	120	0.122 (0.038 to 0.203)	0.996
Excluding large studies (>90th percentile)	600	0.611 (0.192 to 1.016)	0.996
Excluding large studies (>90th percentile)	1,200	1.221 (0.383 to 2.033)	0.996
Excluding large studies (>90th percentile)	1,800	1.832 (0.575 to 3.049)	0.996

Note: Values are posterior medians (95% CrIs). Positive Hedges' g values favour exercise, and P(g > 0) denotes the posterior probability of benefit. All prespecified doses were within the observed overall-exercise dose range for the corresponding scenario. R denotes the assumed pre–post correlation used to derive change-score standard deviations. Large studies were defined by an outcome-specific total sample size above the 90th percentile. CrI, credible interval; MET, metabolic equivalent of task.

Supplementary Table 8.9C. Overall exercise sensitivity estimates for upper-limb function.

Sensitivity analysis	Weekly dose (MET·min/week)	Hedges' g (95% CrI)	P(g > 0)
Primary analysis	120	0.428 (0.165 to 0.703)	0.999
Primary analysis	600	1.708 (0.657 to 2.805)	0.999
Primary analysis	1,200	2.646 (1.017 to 4.345)	0.999
Primary analysis	1,800	3.161 (1.215 to 5.190)	0.999
R = 0.50	120	0.331 (0.066 to 0.596)	0.992

Sensitivity analysis	Weekly dose (MET·min/week)	Hedges' g (95% CrI)	P(g > 0)
R = 0.50	600	1.321 (0.264 to 2.379)	0.992
R = 0.50	1,200	2.046 (0.410 to 3.685)	0.992
R = 0.50	1,800	2.444 (0.489 to 4.402)	0.992
R = 0.90	120	0.643 (0.362 to 0.918)	1.000
R = 0.90	600	2.567 (1.444 to 3.663)	1.000
R = 0.90	1,200	3.976 (2.236 to 5.673)	1.000
R = 0.90	1,800	4.749 (2.671 to 6.776)	1.000
Excluding high-risk studies	120	0.445 (0.174 to 0.717)	0.999
Excluding high-risk studies	600	1.774 (0.694 to 2.862)	0.999
Excluding high-risk studies	1,200	2.747 (1.076 to 4.432)	0.999
Excluding high-risk studies	1,800	3.281 (1.285 to 5.294)	0.999
Excluding large studies (>90th percentile)	120	0.448 (0.168 to 0.726)	0.999
Excluding large studies (>90th percentile)	600	1.789 (0.672 to 2.898)	0.999
Excluding large studies (>90th percentile)	1,200	2.771 (1.040 to 4.489)	0.999
Excluding large studies (>90th percentile)	1,800	3.310 (1.242 to 5.362)	0.999

Note: Values are posterior medians (95% CrIs). Positive Hedges' g values favour exercise, and P(g > 0) denotes the posterior probability of benefit. All prespecified doses were within the observed overall-exercise dose range for the corresponding scenario. R denotes the assumed pre–post correlation used to derive change-score standard deviations. Large studies were defined by an outcome-specific total sample size above the 90th percentile. Estimates of τ approached the upper boundary of the Uniform(0, 2) prior and should be interpreted cautiously. CrI, credible interval; MET, metabolic equivalent of task.

Supplementary Table 8.9D. Exercise type-specific robustness for finger dexterity.

Exercise type	Sensitivity analysis	No. of supported doses compared	Maximum absolute difference in posterior median Hedges' g	Effect direction unchanged	95% CrI crossing status unchanged
CULR	R = 0.50	4	0.098	Yes	Yes
CULR	R = 0.90	4	0.062	Yes	Yes
CULR	Excluding high-risk studies	4	0.039	Yes	Yes
CULR	Excluding large studies	4	0.239	No	Yes
MCT	R = 0.50	3	0.163	Yes	Yes
MCT	R = 0.90	3	0.111	Yes	Yes
MCT	Excluding high-risk studies	3	0.099	Yes	Yes
MCT	Excluding large studies	3	0.106	Yes	Yes
RT	R = 0.50	3	0.293	Yes	Yes
RT	R = 0.90	3	0.137	Yes	Yes
RT	Excluding high-risk studies	3	0.051	Yes	Yes
RT	Excluding large studies	3	0.122	Yes	Yes
TAULHT	R = 0.50	4	0.026	Yes	Yes
TAULHT	R = 0.90	4	0.022	Yes	Yes
TAULHT	Excluding high-risk studies	4	0.038	Yes	Yes
TAULHT	Excluding large studies	4	0.211	Yes	Yes
TOFDT	R = 0.50	4	0.306	Yes	Yes
TOFDT	R = 0.90	4	0.125	Yes	Yes

Exercise type	Sensitivity analysis	No. of supported doses compared	Maximum absolute difference in posterior median Hedges' g	Effect direction unchanged	95% CrI crossing status unchanged
TOFDT	Excluding high-risk studies	4	0.014	Yes	Yes
TOFDT	Excluding large studies	4	0.009	Yes	Yes

Note: Each sensitivity scenario was compared with the primary analysis at prespecified doses supported by both scenarios. The maximum absolute difference is the largest absolute difference in posterior median Hedges' g across these doses. Effect direction and 95% CrI crossing status were classified relative to the primary analysis. R denotes the assumed pre–post correlation used to derive change-score standard deviations. Large studies were defined by an outcome-specific total sample size above the 90th percentile. AET and MBE were omitted because no prespecified report dose was supported by both the primary analysis and any sensitivity scenario. AET, aerobic exercise training; CrI, credible interval; CULR, conventional upper-limb rehabilitation; MBE, mind–body exercise; MCT, multicomponent training; RT, resistance training; TAULHT, technology-assisted upper-limb or hand training; TOFDT, task-oriented finger dexterity training.

Supplementary Table 8.9E. Exercise type-specific robustness for upper-limb function.

Exercise type	Sensitivity analysis	No. of supported doses compared	Maximum absolute difference in posterior median Hedges' g	Effect direction unchanged	95% CrI crossing status unchanged
CULR	R = 0.50	4	0.104	Yes	Yes
CULR	R = 0.90	4	0.003	Yes	Yes
CULR	Excluding high-risk studies	4	0.075	Yes	Yes
CULR	Excluding large studies	4	0.079	Yes	Yes
MBE	R = 0.50	1	0.591	Yes	Yes
MBE	R = 0.90	1	1.673	Yes	Yes
MBE	Excluding high-risk studies	1	0.024	Yes	Yes
MBE	Excluding large studies	1	0.043	Yes	Yes
MCT	R = 0.50	1	0.040	Yes	Yes
MCT	R = 0.90	1	0.024	Yes	Yes
MCT	Excluding high-risk studies	1	0.051	Yes	Yes
MCT	Excluding large studies	1	0.046	Yes	Yes
RT	R = 0.50	4	0.092	Yes	Yes
RT	R = 0.90	4	0.248	Yes	Yes
RT	Excluding high-risk studies	4	0.015	Yes	Yes
RT	Excluding large studies	4	0.117	Yes	Yes
TAULHT	R = 0.50	4	0.240	No	Yes
TAULHT	R = 0.90	4	0.174	Yes	Yes
TAULHT	Excluding high-risk studies	4	0.044	No	Yes
TAULHT	Excluding large studies	4	0.089	No	Yes
TOFDT	R = 0.50	4	0.079	Yes	Yes
TOFDT	R = 0.90	4	0.302	Yes	Yes
TOFDT	Excluding high-risk studies	4	0.026	Yes	Yes
TOFDT	Excluding large studies	4	0.050	Yes	Yes

Note: Each sensitivity scenario was compared with the primary analysis at prespecified doses supported by both scenarios. The maximum absolute difference is the largest absolute difference in posterior median Hedges' g across these doses. Effect direction and 95% CrI crossing status were classified relative to the primary analysis. R denotes the assumed pre–post correlation used to derive change-score standard deviations. Large studies were defined by an outcome-specific total sample size above the 90th percentile. AET was omitted because no prespecified report dose was supported by both the primary analysis and any sensitivity scenario. For MBE and MCT, robustness classifications were based on one supported dose and should not be interpreted as curve-wide agreement. Estimates of τ approached the upper boundary of the Uniform(0, 2) prior and should be interpreted cautiously. AET, aerobic exercise training; CrI, credible interval; CULR, conventional upper-limb rehabilitation; MBE, mind–body exercise; MCT, multicomponent training; RT, resistance training; TAULHT, technology-assisted upper-limb or hand training; TOFDT, task-oriented finger dexterity training.

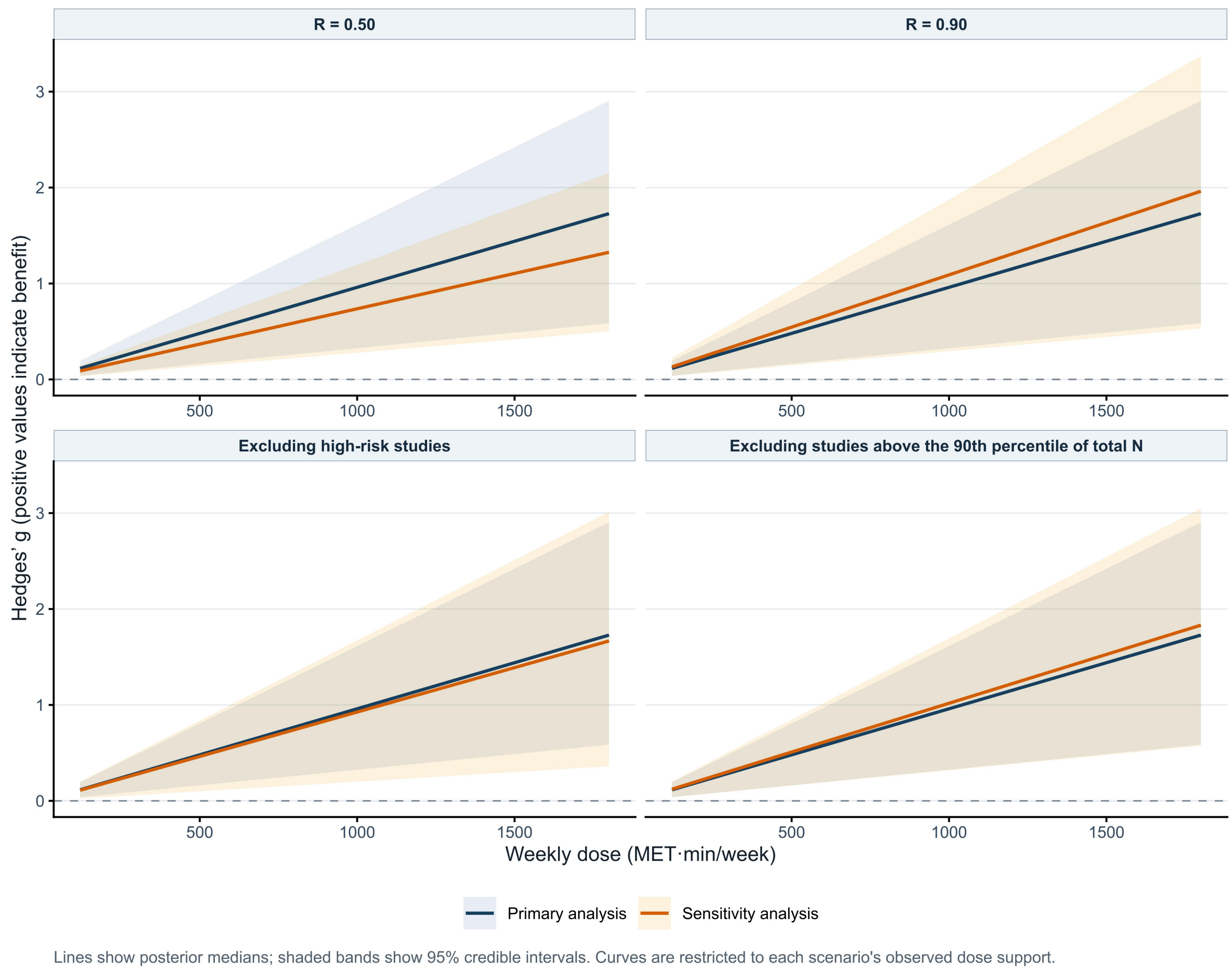

Supplementary Fig. 8.9A. Sensitivity of the overall exercise dose–response curve for finger dexterity. Each panel compares the primary analysis with one sensitivity analysis. Lines and bands show posterior medians and 95% CrIs within scenario-specific observed dose ranges. Positive Hedges' g values favour exercise. R denotes the assumed pre–post correlation. CrI, credible interval; MET, metabolic equivalent of task.

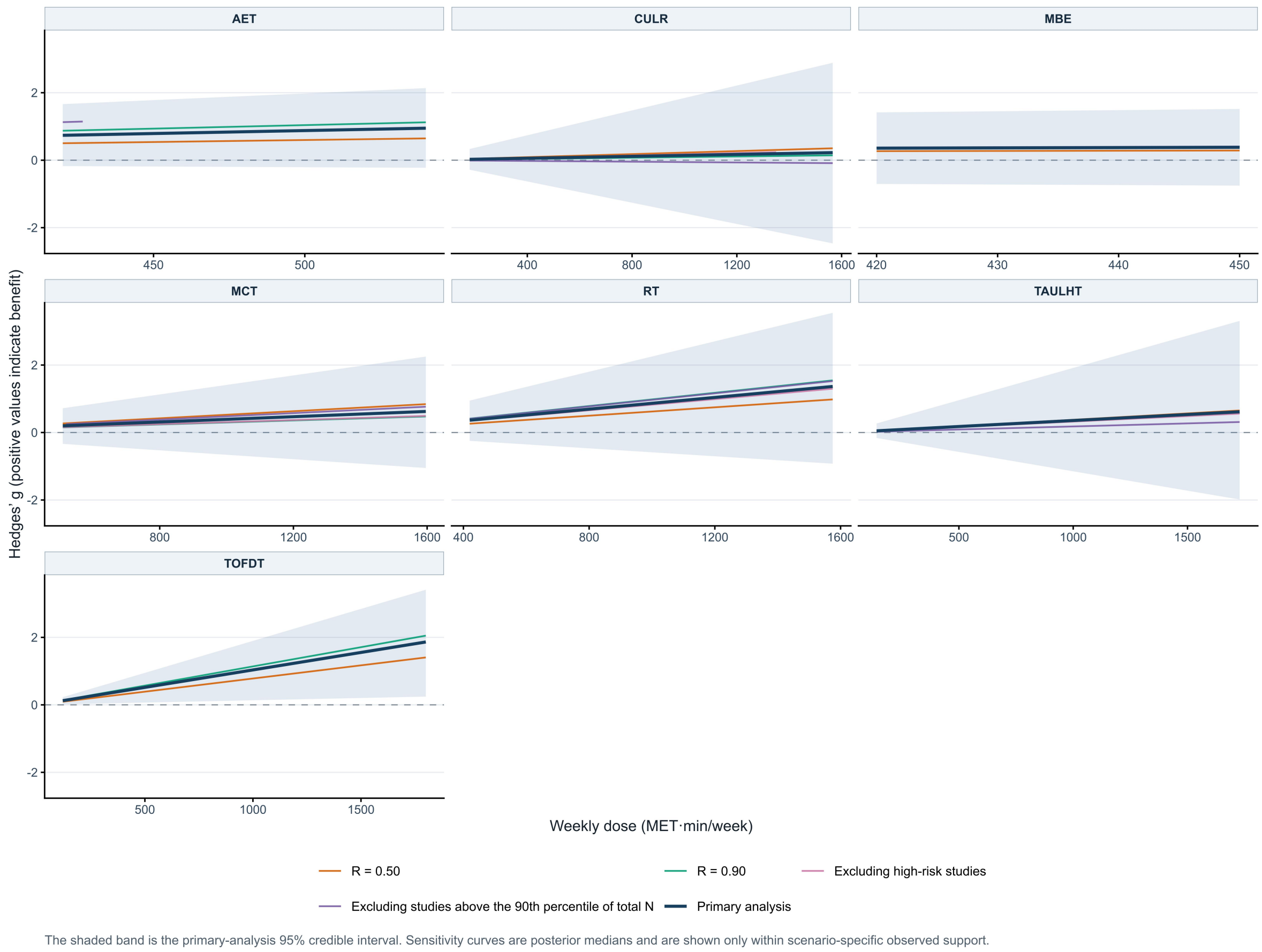

Supplementary Fig. 8.9B. Sensitivity of exercise type-specific dose–response curves for finger dexterity. The dark-blue line and band show the primary posterior median and 95% CrI; coloured lines show sensitivity-analysis medians within scenario-specific observed dose ranges. Gaps indicate unsupported doses. Positive Hedges' g values favour exercise. AET, aerobic exercise training; CrI, credible interval; CULR, conventional upper-limb rehabilitation; MBE, mind–body exercise; MCT, multicomponent training; MET, metabolic equivalent of task; RT, resistance training; TAULHT, technology-assisted upper-limb or hand training; TOFDT, task-oriented finger dexterity training.

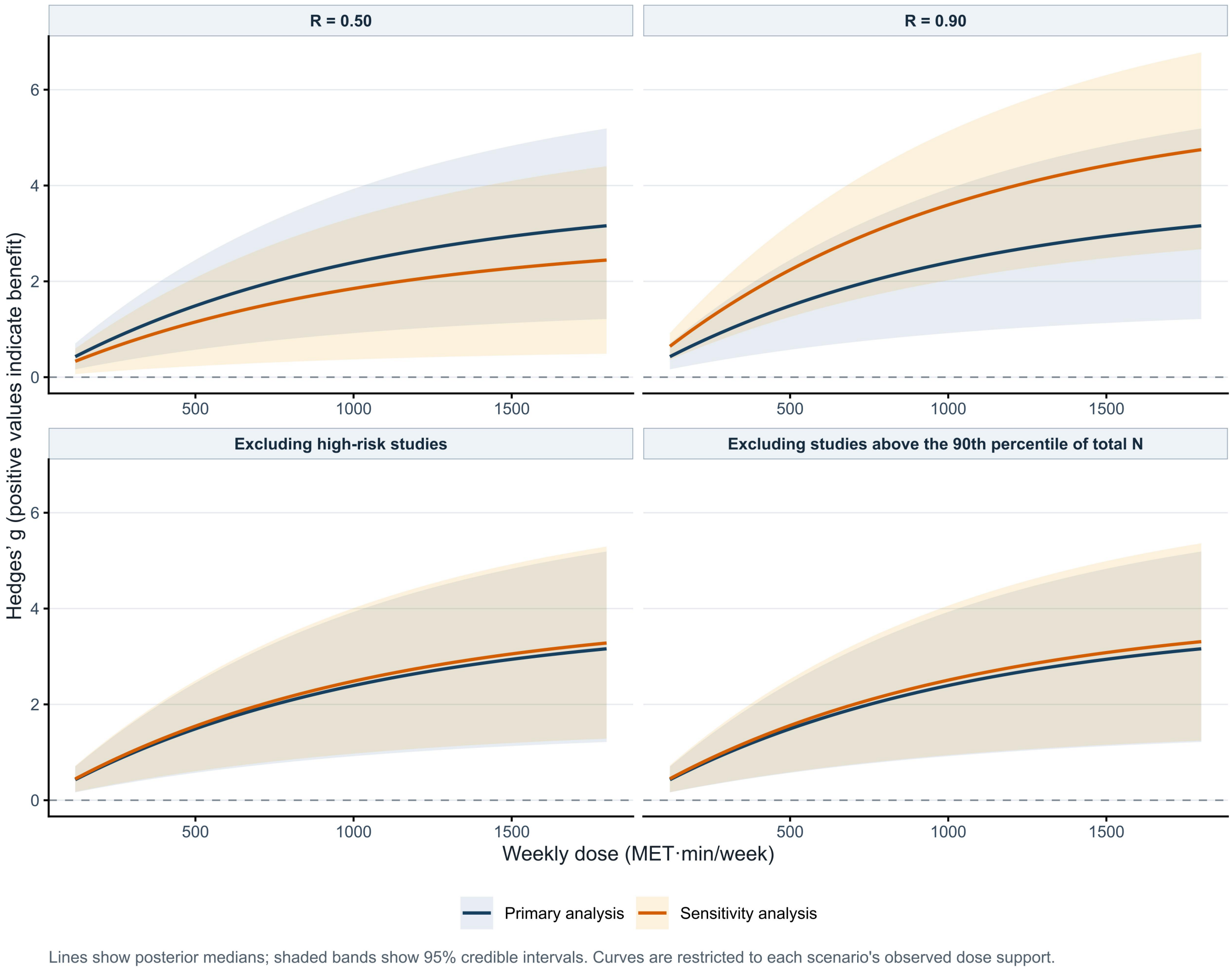

Supplementary Fig. 8.9C. Sensitivity of the overall exercise dose–response curve for upper-limb function. Each panel compares the primary analysis with one sensitivity analysis. Lines and bands show posterior medians and 95% CrIs within scenario-specific observed dose ranges. Positive Hedges' g values favour exercise. R denotes the assumed pre–post correlation. CrI, credible interval; MET, metabolic equivalent of task.

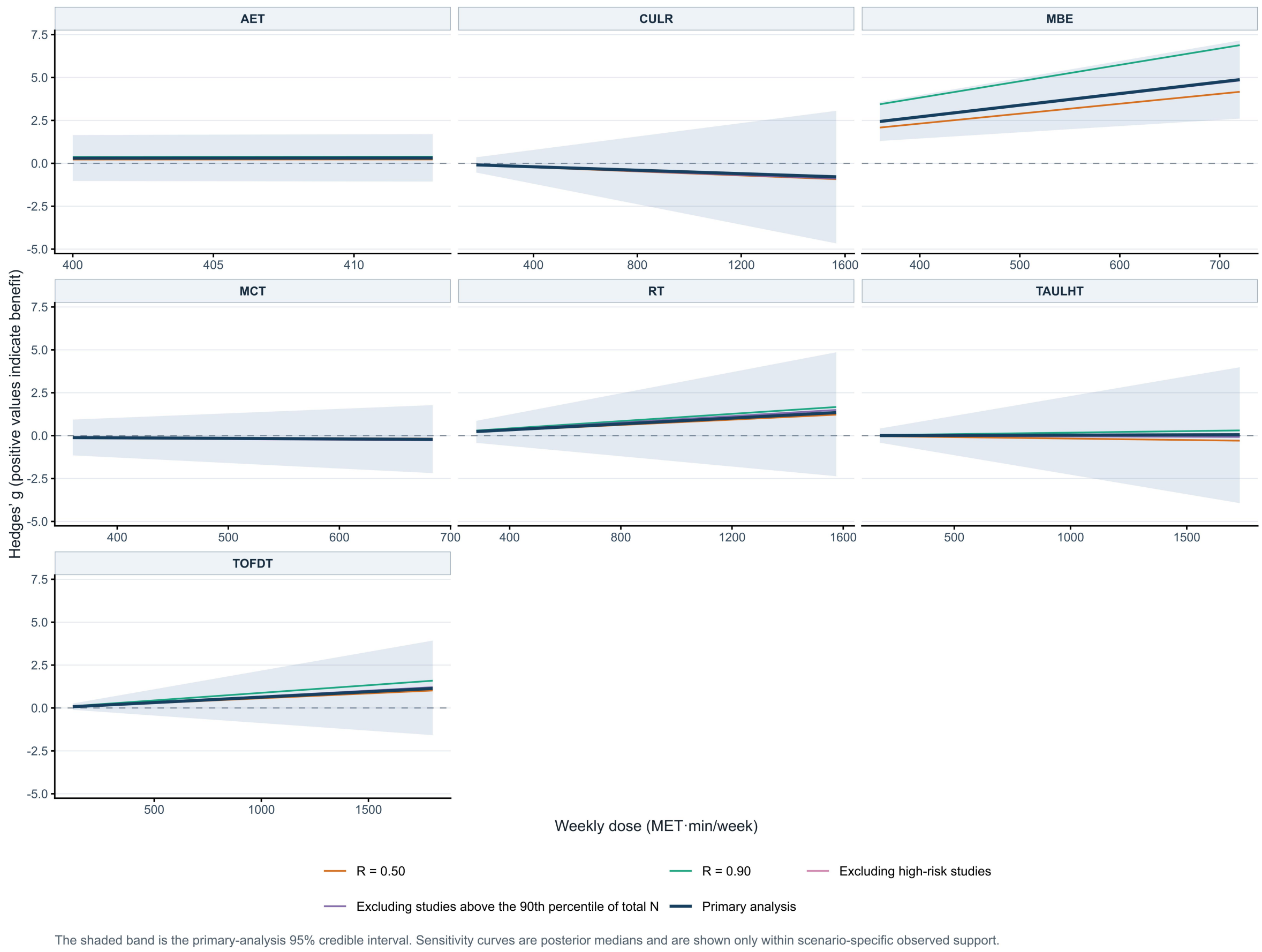

Supplementary Fig. 8.9D. Sensitivity of exercise type-specific dose–response curves for upper-limb function. The dark-blue line and band show the primary posterior median and 95% CrI; coloured lines show sensitivity-analysis medians within scenario-specific observed dose ranges. Gaps indicate unsupported doses. Positive Hedges' g values favour exercise. AET, aerobic exercise training; CrI, credible interval; CULR, conventional upper-limb rehabilitation; MBE, mind–body exercise; MCT, multicomponent training; MET, metabolic equivalent of task; RT, resistance training; TAULHT, technology-assisted upper-limb or hand training; TOFDT, task-oriented finger dexterity training.

References

[1] Chaimani A, Caldwell DM, Li T, Higgins JPT, Salanti G. Undertaking network meta-analyses. Cochrane Handb. Syst. Rev. Interv. Version 6.5, Cochrane; 2024.

[2] Page MJ, McKenzie JE, Bossuyt PM, Boutron I, Hoffmann TC, Mulrow CD, et al. The PRISMA 2020 statement: An updated guideline for reporting systematic reviews. BMJ 2021;372:n71. https://doi.org/10.1136/bmj.n71.

[3] World Health Organization. International Classification of Functioning, Disability and Health: ICF. Geneva: World Health Organization; 2001.

[4] Kleim JA, Jones TA. Principles of experience-dependent neural plasticity: Implications for rehabilitation after brain damage. J Speech Lang Hear Res JSLHR 2008;51:S225-239. https://doi.org/10.1044/1092-4388(2008/018).

[5] Sigrist R, Rauter G, Riener R, Wolf P. Augmented visual, auditory, haptic, and multimodal feedback in motor learning: A review. Psychon Bull Rev 2013;20:21–53. https://doi.org/10.3758/s13423-012-0333-8.

[6] Bull FC, Al-Ansari SS, Biddle S, Borodulin K, Buman MP, Cardon G, et al. World health organization 2020 guidelines on physical activity and sedentary behaviour. Br J Sports Med 2020;54:1451–62. https://doi.org/10.1136/bjsports-2020-102955.

[7] Larkey L, Jahnke R, Etnier J, Gonzalez J. Meditative movement as a category of exercise: Implications for research. J Phys Act Health 2009;6:230–8. https://doi.org/10.1123/jpah.6.2.230.

[8] Osborne JA, Botkin R, Colon-Semenza C, DeAngelis TR, Gallardo OG, Kosakowski H, et al. Physical therapist management of parkinson disease: A clinical practice guideline from the American physical therapy association. Phys Ther 2022;102:pzab302. https://doi.org/10.1093/ptj/pzab302.

[9] National Center for Complementary and Integrative Health. Mind and Body Practices. NCCIH n.d. https://www.nccih.nih.gov/health/mind-and-body-practices (accessed July 30, 2026).

[10] Julian P. T. Higgins, Tianjing Li, Jonathan J. Deeks. Chapter 6: Choosing effect measures and computing estimates of effect. Cochrane Handb. Syst. Rev. Interv. 6.5, Cochrane; 2024.

[11] Herrmann SD, Willis EA, Ainsworth BE, Barreira TV, Hastert M, Kracht CL, et al. 2024 adult compendium of physical activities: A third update of the energy costs of human activities. J Sport Health Sci 2024;13:6–12. https://doi.org/10.1016/j.jshs.2023.10.010.

[12] American College of Sports Medicine. ACSM's Guidelines for Exercise Testing and Prescription. 12th ed. Philadelphia, PA: Wolters Kluwer; 2025.

[13] Borg G. Borg's Perceived Exertion and Pain Scales. Champaign, IL: Human Kinetics; 1998.

[14] Jeng B, Cederberg KLJ, Lai B, Sasaki JE, Bamman MM, Motl RW. Accelerometer output and its association with energy expenditure in persons with mild-to-moderate parkinson's disease. PloS One 2020;15:e0242136. https://doi.org/10.1371/journal.pone.0242136.

[15] Jeng B, Cederberg KLJ, Lai B, Sasaki JE, Bamman MM, Motl RW. Wrist-based accelerometer cut-points for quantifying moderate-to-vigorous intensity physical activity in parkinson's disease. Gait Posture 2022;91:235–9. https://doi.org/10.1016/j.gaitpost.2021.10.027.
